# Systematic exploration of different E3 ubiquitin ligases: an approach towards potent and selective CDK6 degraders†
†Electronic supplementary information (ESI) available. See DOI: 10.1039/d0sc00167h


**DOI:** 10.1039/d0sc00167h

**Published:** 2020-03-04

**Authors:** Christian Steinebach, Yuen Lam Dora Ng, Izidor Sosič, Chih-Shia Lee, Sirui Chen, Stefanie Lindner, Lan Phuong Vu, Aleša Bricelj, Reza Haschemi, Marius Monschke, Elisabeth Steinwarz, Karl G. Wagner, Gerd Bendas, Ji Luo, Michael Gütschow, Jan Krönke

**Affiliations:** a Pharmaceutical Institute , Department of Pharmaceutical & Medicinal Chemistry , University of Bonn , An der Immenburg 4 , 53121 Bonn , Germany . Email: guetschow@uni-bonn.de; b Department of Internal Medicine III , University Hospital Ulm , Albert-Einstein-Allee 23 , 89081 Ulm , Germany . Email: jan.kroenke@uni-ulm.de; c Faculty of Pharmacy , University of Ljubljana , Aškerčeva cesta 7 , 1000 Ljubljana , Slovenia; d Laboratory of Cancer Biology and Genetics , Center for Cancer Research , National Cancer Institute , Bethesda , MD 20892 , USA; e Pharmaceutical Institute , Department of Pharmaceutical & Cell Biological Chemistry , University of Bonn , An der Immenburg 4 , 53121 Bonn , Germany; f Pharmaceutical Institute , Pharmaceutical Technology , University of Bonn , Gerhard-Domagk-Straße 3 , 53121 Bonn , Germany

## Abstract

Cyclin-dependent kinase 6 (CDK6) is an important regulator of the cell cycle. Together with CDK4, it phosphorylates and inactivates retinoblastoma (Rb) protein. In tumour cells, CDK6 is frequently upregulated and CDK4/6 kinase inhibitors like palbociclib possess high activity in breast cancer and other malignancies. Besides its crucial catalytic function, kinase-independent roles of CDK6 have been described. Therefore, targeted degradation of CDK6 may be advantageous over kinase inhibition. Proteolysis targeting chimeras (PROTACs) structurally based on the cereblon (CRBN) ligand thalidomide have recently been described to degrade the targets CDK4/6. However, CRBN-based PROTACs have several limitations including the remaining activity of immunomodulatory drugs (IMiDs) on Ikaros transcription factors as well as CRBN inactivation as a resistance mechanism in cancer. Here, we systematically explored the chemical space of CDK4/6 PROTACs by addressing different E3 ligases and connecting their respective small-molecule binders *via* various linkers to palbociclib. The spectrum of CDK6-specific PROTACs was extended to von Hippel Lindau (VHL) and cellular inhibitor of apoptosis protein 1 (cIAP1) that are essential for most cancer cells and therefore less likely to be inactivated. Our VHL-based PROTAC series included compounds that were either specific for CDK6 or exhibited dual activity against CDK4 and CDK6. IAP-based PROTACs caused a combined degradation of CDK4/6 and IAPs resulting in synergistic effects on cancer cell growth. Our new degraders showed potent and long-lasting degrading activity in human and mouse cells and inhibited proliferation of several leukemia, myeloma and breast cancer cell lines. In conclusion, we show that VHL- and IAP-based PROTACs are an attractive approach for targeted degradation of CDK4/6 in cancer.

## Abstract 2

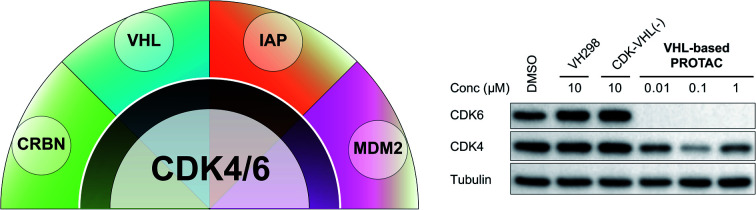
Cyclin-dependent kinase 6 (CDK6) is an important regulator of the cell cycle. Together with CDK4, it phosphorylates and inactivates retinoblastoma (Rb) protein.

## Introduction

**Fig. 1:**
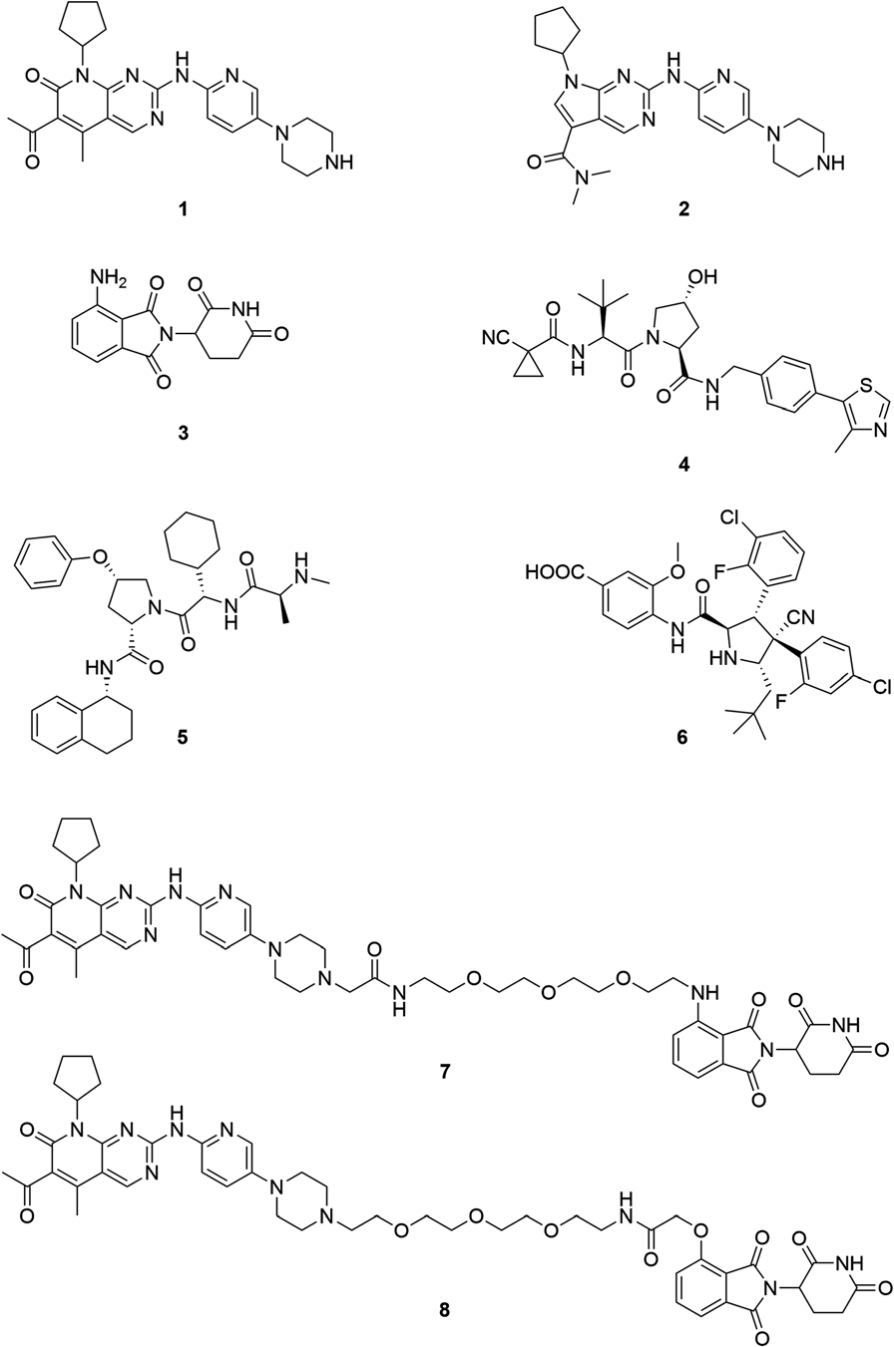
Chemical structures of the CDK4/6 inhibitors palbociclib (**1**) and ribociclib (**2**), selected E3 ligase ligands, *i.e.* the CRBN ligand pomalidomide (**3**), the VHL inhibitor VH298 (**4**), an IAP ligand (**5**), and the MDM2 antagonist idasanutlin (**6**), as well as published CDK6 selective PROTACs YKL-06-102 (**7**), and BSJ-03-123 (**8**).

Cyclin-dependent kinases 4 and 6 (CDK4/6) are key orchestrators of cell cycle regulation as they control the progression from G1- to S-phase of the cell cycle.^1^ Both kinases are catalytically inactive until associating with D-type cyclins, which leads to the phosphorylation of the tumour suppressor retinoblastoma (Rb). Dysregulation of the cell cycle, fuelled by up-regulated and over-activated CDKs, significantly contributes to uncontrolled cell proliferation, which is a fundamental hallmark of cancer.^2^ The dual CDK4/6 inhibitors (CDK4/6i) palbociclib (**1**) and ribociclib (**2**) (Fig. 1) have recently been approved by the FDA and EMA for the treatment of patients with advanced or metastatic breast cancer in combination with anti-hormone therapy.^3^ Several clinical trials for other malignant diseases are currently ongoing.^4^–^6^ Despite the high level of homology between CDK4 and CDK6, homologue-specific functions were identified in recent studies.^7^–^11^ Although tremendous efforts have been made to find new CDK4/6i,^12^,^13^ the current array of inhibitors failed to discriminate between both homologues.^14^,^15^ The majority of strategies to design CDK4/6i were focused on CDK4/6-cyclin D dimer activities without considering CDK interacting protein/kinase inhibitory protein (CIP/KIP) family proteins.^16^ Very recently, crystallographic studies demonstrated that p21^CIP^ and p27^KIP^ are assembly factors for active CDK4/6-cyclin D trimer complexes which are not sensitive to CDK4/6i.^17^ Furthermore, CDK6 has activities that are partially kinase-independent and not affected by kinase inhibitors.^18^ These functions indicate the particular role of CDK6 in tissue homeostasis and differentiation that is not shared with CDK4, thus rendering CDK6 an attractive anti-tumour target.^9^ Accordingly, CDK6 degradation strategies might outperform inhibitors and constitute one of the most promising approaches to expand CDK drug discovery.^14^,^15^,^19^,^20^

The field of proteolysis targeting chimeras (PROTACs) has made tremendous achievements and reached substantial milestones in the last years.^21^–^29^ Briefly, PROTACs are bifunctional small molecules, which comprise two linker-connected moieties that simultaneously bind a target protein and an E3 ubiquitin ligase. Frequently employed E3 ligase binders **3–6** which have successfully been utilized in PROTAC design are presented in Fig. 1. Once the PROTAC molecule has entered the cell, ternary complexes with the target can be formed, causing selective ubiquitination and degradation by the proteasome. In particular, the ability to knockdown kinases is an exciting scope of PROTACs and has received much attention.^19^,^30^–^32^

The vital significance of CDK4/6 in key cellular processes inspired researchers to apply the PROTAC technology for the development of novel kinase modulators. These approaches, including the one that is reported herein, utilized 2-aminopyrimidines, palbociclib or ribociclib, as the CDK4/6-addressing moiety.^33^–^37^ The corresponding molecular design culminated in the discovery of the prototypical PROTACs **7** and **8** (Fig. 1).^33^ The common feature of recently published CDK4/6 degraders (CDK4/6d) is the phthalimide ligand for the cullin-RING E3 ubiquitin ligase cereblon (CRL4^CRBN^). However, despite potent degradation of CDK6, these CRBN-based PROTACs have some limitations including off-target effects related to Ikaros transcription factors as well as a possible resistance of cancer cells to CRBN-based PROTACs through genetic *CRBN* inactivation.^38^ In general, gene expression levels of an E3 ligase component might affect the activity of the recruiting PROTAC, and tissue specificity may be guided by addressing different E3 ligases. Accordingly, to systematically explore the CDK4/6 degradation space, we designed palbociclib-based PROTACs for recruiting four different E3 ligases, *i.e.* CRL4^CRBN^, von Hippel Lindau (CRL2^VHL^), and two non-CRL ligases, *i.e.* cellular inhibitor of apoptosis protein 1 (cIAP1), and mouse double minute 2 homolog (MDM2).

## Results and discussion

### Chemistry

**Scheme 1:**
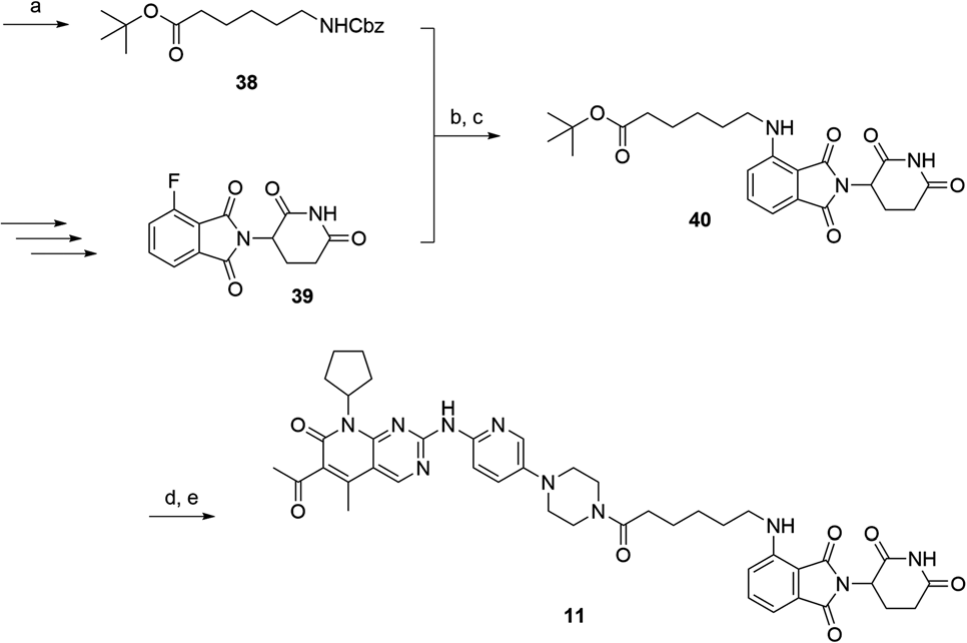
Synthesis of the CRBN-addressing CDK4/6 degrader **11**. Reagents and conditions: (a) *Z*-6-aminohexanoic acid, DCC, DMAP, *t*BuOH, CH_2_Cl_2_, rt, 16 h; (b) Pd/C, H_2_, EtOAc, 16 h, rt; (c) DIPEA, DMSO, 90 °C, 24 h; (d) TFA, CH_2_Cl_2_, 40 °C, 2 h; (e) HATU, DMSO, rt, 16 h.

The putative CRBN-addressing CDK4/6d (Table 1) were prepared by reacting 4-fluoro-thalidomide (**39**, Scheme 1) with ten different orthogonally protected amino to carboxylic acid (N-to-C) linkers, which have been elaborated by us.^39^,^40^ These building blocks include polyethylene glycol (PEG)- and other alkylidene-based linkers. Subsequent deprotection of the *tert*-butyl ester and HATU-mediated coupling to the piperazine handle of palbociclib yielded the envisaged CRBN-CDK PROTACs **11–20**. An exemplary synthesis of compound **11** is outlined in Scheme 1. Compound **21** (Table 1) represents a negative control bearing an *N*-methylated-pomalidomide moiety which is unable to bind to CRBN. Notably, the PROTAC structures have an acylated piperazine moiety, thus differing from previously reported CDK4/6d,^33^–^37^ all of which possess an alkylated piperazine with basic properties. On the contrary, our synthesis resulted in compounds with a tertiary amide which are not protonated at physiological pH value. This modification was expected to have significant effects on the activity and selectivity of the CDK4/6-addressing PROTACs.

**Table 1:** Activities of the CRBN-addressing CDK4/6d

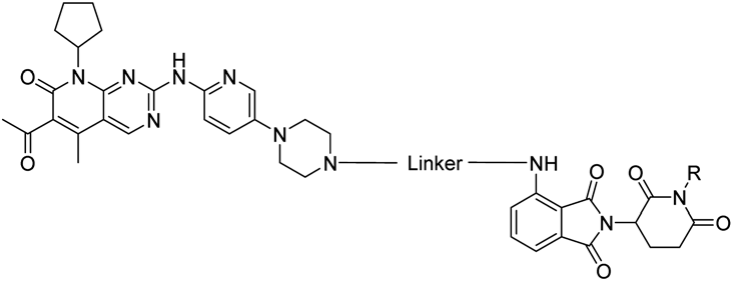							
Cmpd	Linker	R	elog *D*_7.4_^*a*^	*D*_CDK4_ ^*b*^	*D*_CDK6_ ^*b*^	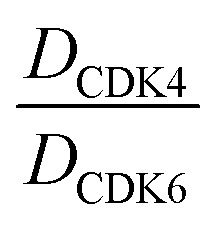 ^*c*^	*D*_IKZF1_ ^*d*^
				0.1 μM	0.1 μM	0.1 μM	0.1 μM
**3** (POM)	—	—	n.d^*e*^	91	91	n.d.	17
**8** (BSJ-03-123)	—	—	2.5	27	5.5	4.9	82
**11**	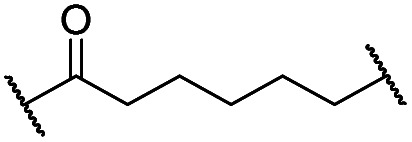	H	3.4	25	11	2.3	1.0
**12**	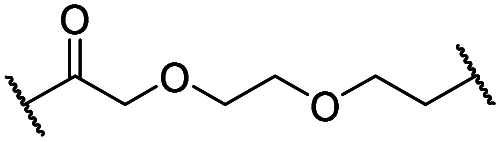	H	2.8	19	8.4	2.3	0.8
**13**	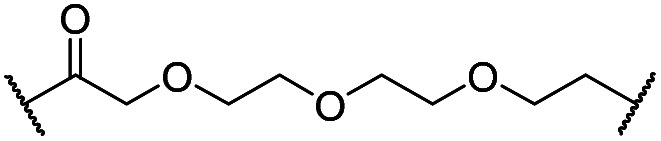	H	2.8	15	7.7	1.9	1.0
**14**	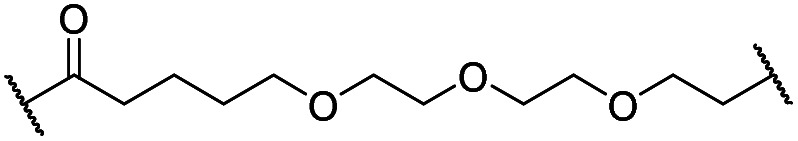	H	3.1	26	7.8	3.3	0.8
**15**	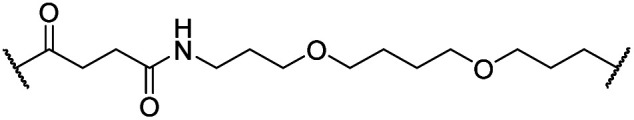	H	3.1	91	65	1.4	56
**16**	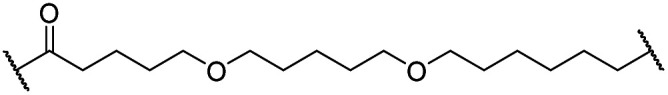	H	4.6	76	32	2.4	6.7
**17**	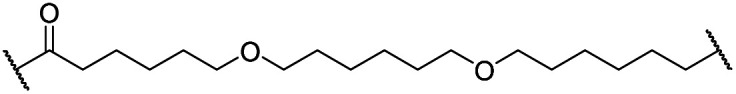	H	5.2	86	51	1.7	18
**18**	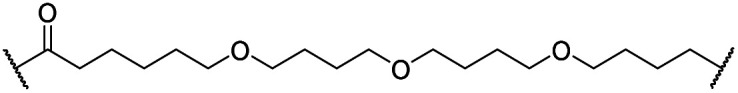	H	4.4	57	17	3.4	5.9
**19**	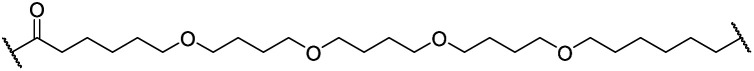	H	5.4	>95	51	n.d.	35
**20**	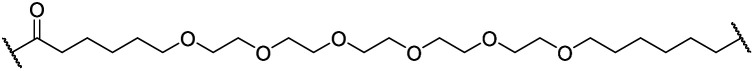	H	3.7	86	16	5.4	5.8
**21**	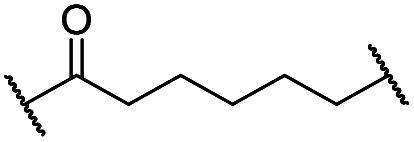	Me	3.6	95	>95	n.d.	93

^*a*^Experimental distribution coefficient at pH 7.4.

^*b*^CDK4 or CDK6 degradation indicated as remaining CDK4 or CDK6 levels after 16 h treatment of each compound at the indicated concentration. Percentage values are normalized to DMSO-treated MM.1S cells and the respective loading controls (100%). All of the data were the average of at least three independent experiments.

^*c*^Selectivity ratio for the degradation of CDK6 over CDK4.

^*d*^Neosubstrate degradation indicated as remaining IKZF1 levels, respectively.

^*e*^Not determined.

The synthesis of VHL-addressing CDK4/6d (Table 2) was accomplished by fusing the VHL ligand VH032 (ref. 41) (**52**, Scheme S1, ESI†) to different chloro to carboxylic acid (Cl-to-C) linkers which were in most cases synthesized by a BAIB/TEMPO-mediated oxidation of their corresponding primary alcohol precursors. The obtained chloro-linker-VHL ligand conjugates were subjected to a Finkelstein reaction and the *in situ* formed alkyl iodides led to the successful alkylation of palbociclib. Scheme S1 (ESI†) shows the preparation of **22** by the typical route to such PROTACs. The negative control compound **26** (Table 2) exhibits a reversed stereochemistry at C-4 of the hydroxyproline unit, a modification which abolishes binding to VHL.^42^,^43^ For the composition of compound **27**, the same PEG4 linker as in **24** was installed, but a methylated derivative of VH032, which possesses an enhanced binding affinity for VHL^43^ was used.

**Table 2:** Activities of the VHL-addressing CDK4/6d of the ‘amide’ and ‘phenoxy’ series

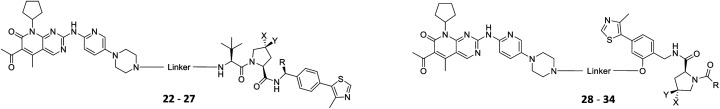									
Cmpd	Linker	R	X	Y	elog *D*_7.4_^*a*^	*D*_CDK4_ ^*b*^	*D*_CDK6_ ^*b*^		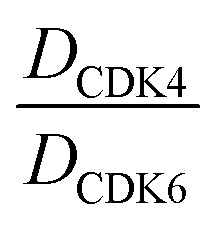 ^*c*^
						0.1 μM	0.1 μM	1 μM	0.1 μM
**4** (VH298)	—	—	—	—	n.d.^*d*^	>95	>95	n.d.	n.d.
**8** (BSJ-03-123)	—	—	—	—	2.5	27	5.5	2.1	4.9

**‘Amide’ subseries**									
**22**	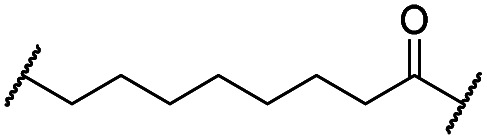	H	OH	H	3.3	12	2.9	4.1	4.1
**23**	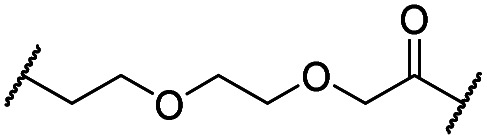		OH	H	2.7	61	8.2	n.d.	7.4
**24**	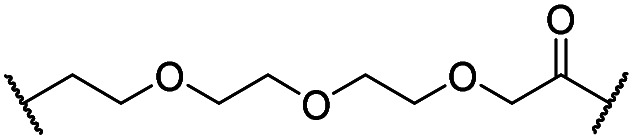		OH	H	3.5	80	14	n.d.	5.7
**25**	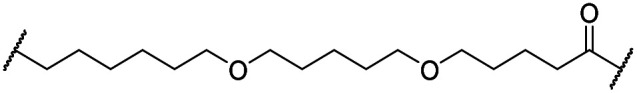		OH	H	4.8	>95	40	n.d.	n.d.
**26**	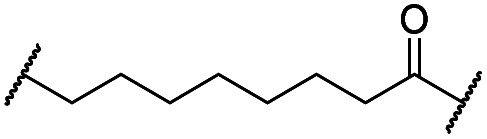		H	OH	3.2	>95	>95	n.d.	n.d.
**27** (CST620)	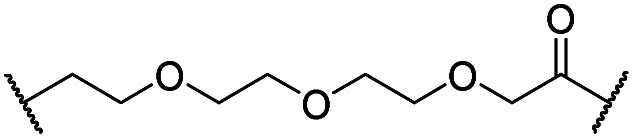	Me	OH	H	2.9	33	1.7	15	19

**‘Phenoxy’ subseries**									
**28**	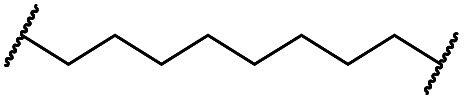	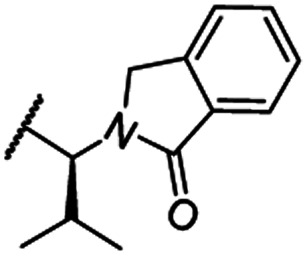	OH	H	4.5	>95	62	n.d.	n.d.
**29**	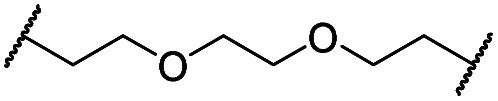		OH	H	3.2	>95	29	n.d.	n.d.
**30**	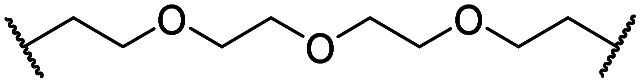		OH	H	3.1	94	69	n.d.	1.4
**31**	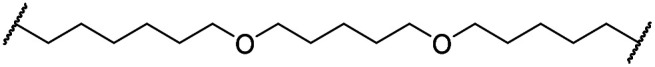		OH	H	5.3	>95	65	n.d.	n.d.
**32**	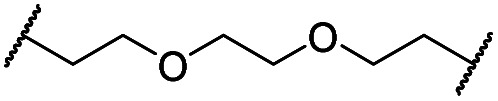		H	OH	3.0	82	>95	n.d.	n.d.
**33**	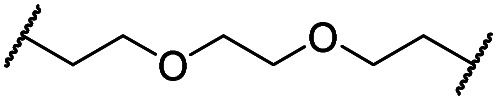	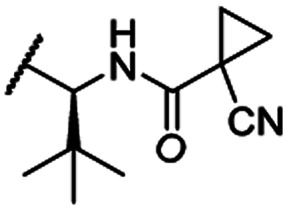	OH	H	3.0	65	1.4	n.d.	46
**34** (CST651)	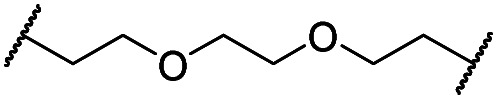	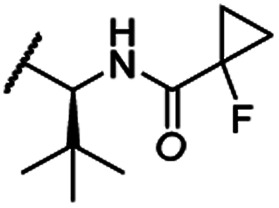	OH	H	3.1	44	1.4	8.5	31

^*a*^Experimental distribution coefficient at pH 7.4.

^*b*^CDK4 or CDK6 degradation indicated as remaining CDK4 or CDK6 levels after 16 h treatment of each compound at the indicated concentration. Percentage values are normalized to DMSO-treated MM.1S cells and the respective loading controls (100%). All of the data were the average of at least three independent experiments.

^*c*^Selectivity ratio for the degradation of CDK6 over CDK4.

^*d*^Not determined.

**Scheme 2:**
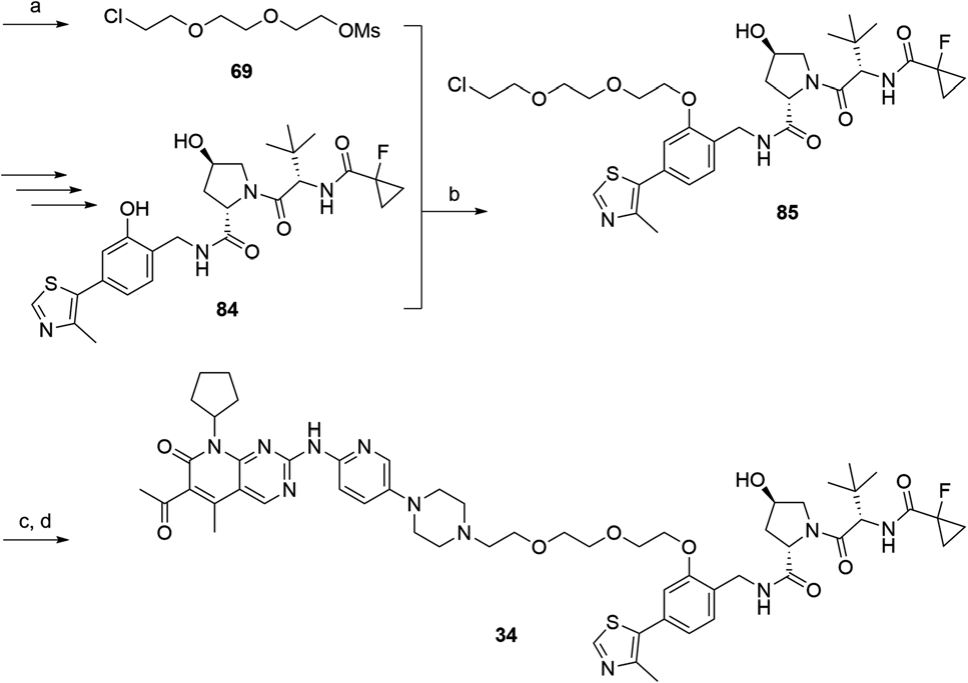
Synthesis of the VHL-addressing CDK4/6 degrader **34** with a different connection of the linkers. Reagents and conditions: (a) 2-[2-(2-chloroethoxy)ethoxy]ethanol, MsCl, Et_3_N, CH_2_Cl_2_, rt, 16 h; (b) (i) Cs_2_CO_3_, DMF, rt, 16 h; (ii) 60 °C, 3 h; (c) NaI, acetone, 60 °C, 48 h; (d) DIPEA, DMSO/DMF, 80 °C, 24 h.

In a second VHL-based series, a distinct functionalization site of the VHL ligand was chosen as an exit vector (Table 2 and Scheme 2).^44^,^45^ As reported, different points of attachment to the VHL ligand can result in two contrasting E3 ligase recruitment geometries and an isoform-selective degradation of two closely related proteins.^44^ To unambiguously investigate the impact of the linker attachment point on the target degradation, the same linkers as before were realized. Four different chloro to methane-sulfonate ester (Cl-to-OMs) linkers were coupled to the phenolic group of a VHL ligand (Table S6, ESI†) under mild conditions. The so obtained chloro-linker-VHL ligand conjugates were subjected to a similar reaction sequence as described above. For the assembly of the VHL non-binding diastereomer **32** (Table 2), hydroxyproline with reversed stereochemistry at C-4 was again incorporated.

To assess the degradability of CDK4/6 by further ubiquitin ligases, we thought to address the E3 ligases IAP and MDM2, both commonly hijacked for degrader design.^46^ For the former ligase, ligand **5**^47^ (Fig. 1) was selected, which was obtained in the course of an intensive structural evaluation of different IAP-based degraders.^48^ For MDM2, the highly potent and selective antagonist idasanutlin (**6**),^49^ was chosen, which has already been successfully incorporated into BRD4-targeting PROTACs.^50^ The resulting IAP- and MDM2- based degraders **35** and **37** are depicted in Tables 3 and S5 (ESI†), respectively. For both compounds, the same PEG4 linker as present in BSJ-03-123 (**8**), as well as in **13**, **24**, and **30** (Tables 1 and 2) was employed. The diastereomeric compound **36** was synthesized as a negative control for degrader **35**.

**Table 3:** Activities of the IAP-addressing CDK4/6 degrader

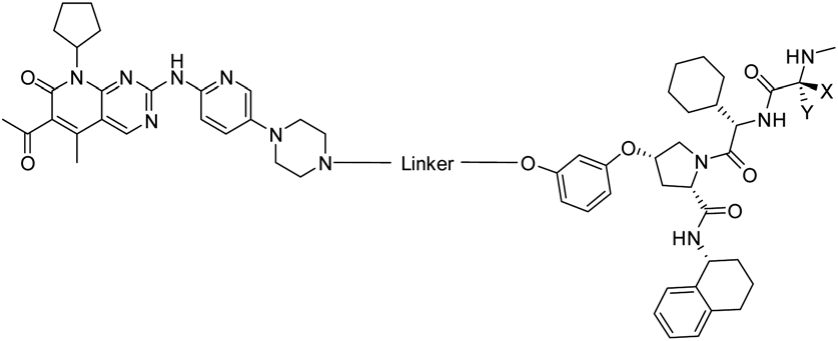							
Cmpd	Linker	X	Y	elog *D*_7.4_^*a*^	*D*_CDK4_ ^*b*^	*D*_CDK6_ ^*b*^	
					0.1 μM	0.1 μM	1 μM
**5**	—	—	—	n.d.^*c*^	n.d.	n.d.	n.d.
**8** (BSJ)	—	—	—	2.5	27	5.5	2.1
**35**	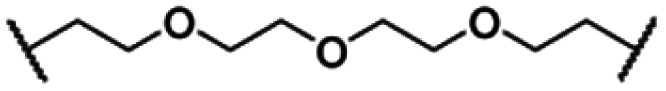	Me	H	4.3	77	75	18
**36**	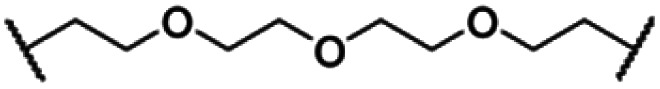	H	Me	4.5	>95	>95	n.d.

^*a*^Experimental distribution coefficient at pH 7.4.

^*b*^CDK4 or CDK6 degradation indicated as remaining CDK4 or CDK6 levels after 16 h treatment of each compound at the indicated concentration. Percentage values are normalized to DMSO-treated MM.1S cells and the respective loading controls (100%). All of the data were the average of at least three independent experiments.

^*c*^Not determined.

Previous reports on principles of PROTAC design highlighted the importance of different physiochemical properties to achieve successful degradation.^46^,^51^,^52^ In order to assess activity determining physicochemical properties, we calculated molecular descriptors, *i.e.* the molecular weight, the topological polar surface area (TPSA), the number of rotatable bonds (NRotB), as well as hydrogen bond donors (HBD) and acceptors (HBA) of all compounds (Tables S1–S4, ESI†). Furthermore, the distribution coefficients (log *D*) were determined experimentally (Tables 1–3 and S5, ESI†) as a measure for lipophilicity. To draw structure–degradation relationships, we calculated the degrader score (Deg_S)^46^ as an overall measure of CDK4/6d efficacy (Tables S1–S4, ESI†).

### Amide-connected CRBN-addressing PROTACs

We investigated the effects of pomalidomide-based compounds **11–20** on the protein levels of CDK4 and CDK6 by western blot analyses of the multiple myeloma (MM) cell line MM.1S. After treatment with the ten PROTACs at 0.1 μM for 16 hours, a reduction of CDK4/6 protein levels was observed in most cases (Fig. S1, ESI†). We determined the concentration of both kinases and CRBN, the latter in order to control the expression of the corresponding ligase. Since IMiDs, such as pomalidomide, cause the degradation of the lymphoid transcription factors Ikaros (IKZF1) and Aiolos (IKZF3) through the involvement of CRBN,^53^,^54^ this undesired effect was inspected. In order to compare the degrading efficacy, the western blotting data from replicates were quantified and the mean remaining protein concentrations are listed in Table 1. In principle, the degradation of CDK4/6 can be induced by PROTACs bearing an amide linkage between the piperazine of the palbociclib portion and the linker. Although the linkers of the CDK4/6d spanned 6 to 28 linear atoms, the effects of the linker lengths were less pronounced in comparison with the lipophilicity of the PROTACs. Representatives with log *D* values lower than 4 led to superior CDK6 degradation, compared to the more hydrophobic compounds **16–19**. The incorporation of an additional carboxamide moiety into the linker of **15** was disadvantageous. As further characteristics of CDK4/6d, the desired selectivity of CDK6 over CDK4 degradation and the unwanted influence on the IKZF1 concentration were determined. Some of our compounds reached the selectivity ratio of BSJ-03-123 (**8**), but most of them caused reduced levels of IKZF1. This finding was in line with other studies which utilized the pomalidomide substructure for ligase recruitment.^33^,^35^ For detailed mechanistic investigations, PROTAC **11** was selected and the methylated analogue **21** was synthesized as CRBN non-binding control compound. Next, we determined the concentration- and time-dependent activity of **11** (Fig. S2, ESI†). As shown by means of appropriate inhibitors, CDK4/6 degradation was governed by the ubiquitin–proteasome system (Fig. S3, ESI†). CRBN knockout and competition with **3** confirmed the involvement of the E3 ligase CRBN in the degradation of CDK4/6 by amide-connected, pomalidomide-derived CDK4/6d (Fig. S3, ESI†).

**Fig. 2:**
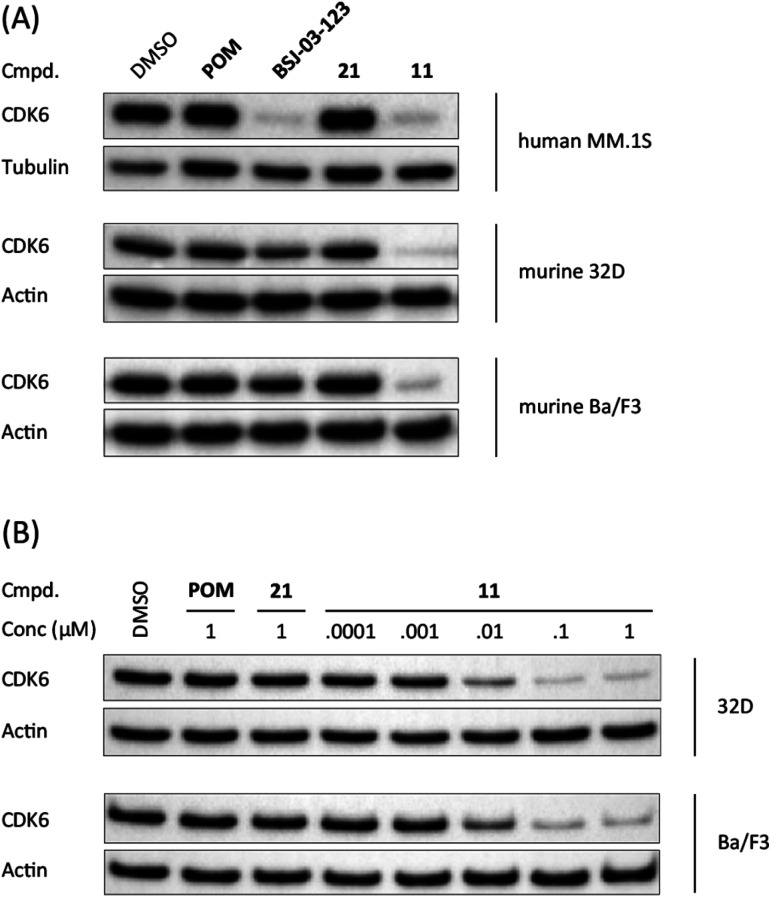
CDK6 degradation by CRBN-based degrader **11** is conserved in murine cell lines. (A) Human cell lines MM.1S and murine cell lines 32D and Ba/F3 were treated with vehicle, pomalidomide (POM), CDK6-selective degrader BSJ-03-123 (**8**), negative control **21**, or PROTAC **11** at 0.1 μM for 16 h; (B) CDK6-degradation is induced in a dose-dependent manner in murine cells. Murine cell lines 32D and Ba/F3 were treated with PROTAC **11** at indicated concentrations for 16 h.

When bound to IMiDs, human CRBN induces the recruitment of the neosubstrates IKZF1 and IKZF3 *via* a Val-388 interaction,^53^,^55^ leading to their subsequent proteasomal degradation. In mice, the single amino acid Val-388 is replaced by isoleucine (Ile-391), which renders murine CRBN inactive to degrade neosubstrates.^56^ The previously reported BRD4 degrader dBET1 was capable of binding to murine CRBN and performed as an active PROTAC in murine cells.^57^,^58^ To test whether our CRBN-based degrader **11** maintains CDK6 degradation across different species, we performed western blotting experiments with the murine myeloid 32D cell line and the murine pro-B-cell line Ba/F3 (Fig. 2A). PROTAC **11**, but not its chemically matched negative control **21**, induced strong CDK6 degradation in both 32D and Ba/F3 cells after treatment with 0.1 μM of these compounds. While the activity of BSJ-03-123 (**8**) was attenuated at this concentration, **11** mediated on-target effects at concentrations as low as 10 nM (Fig. 2B).

A further limitation for the (pre)clinical application of CRBN-based PROTACs is that CRBN is dispensable for most cancer cell lines^59^ and genetic inactivation of *CRBN* constitutes a resistance mechanism to IMiDs in multiple myeloma.^60^ For PROTACs hijacking E3 ligases such as VHL and cIAP1, which are more essential for cancer cells, as indicated by CRISPR-based knockout screens from the DepMap database (Fig. S4, ESI†),^59^ such a resistance mechanism is predicted to be unlikely.

### VHL-based PROTACs

In order to exploit a selection of different ligases for the targeted degradation of CDK4/6 and to expand the chemical space of CDK4/6d, we first envisaged recruiting the most essential E3 ligase, *i.e.* VHL. Despite the extraordinary importance of VHL in the PROTAC field, this E3 ligase has not yet been utilized for the successful development of CDK4/6 PROTACs and was even considered to be inappropriate.^37^ We followed a combinatorial approach towards VHL-based PROTACs by assembling four different linkers of various lengths and lipophilicities and two VHL ligands bearing different exit vectors for linker connection,^43^,^61^,^62^ which were ultimately incorporated into the final palbociclib-derived CDK4/6d. Noteworthy, all PROTACs of the first, ‘amide’ subseries (Tables 2 and S6, ESI†) had pronounced ability to suppress CDK4/6 protein levels. While **22** had high activity on both, CDK4 and CDK6, the introduction of a different linker in **23** and **24** shifted selectivity towards CDK6, similar to that of BSJ-03-123 (**8**) (Fig. S5, ESI†). These results indicate, for the first time, that palbociclib-derived CDK4/6d with both VHL- and CRBN-ligands share the preferred degradation of CDK6 over CDK4. Again, degrading activity was accompanied by moderate lipophilicity with log *D* values lower than 4. We selected one of the successful, PEG-containing compounds, *i.e.***24** whose linker corresponded to that of **8**, for further chemical modification (Table S6, ESI†). The tailored introduction of a methyl group in the VHL-addressing part,^43^ resulted in the optimized PROTAC **27** (Table 2) with respect to both, potency and selectivity. Extensive experiments with **27** addressed the concentration- and time-dependent depletion and the confirmation of the VHL ligase/proteasome-mediated protein degradation (Fig. S5 and S6, ESI†).

**Fig. 3:**
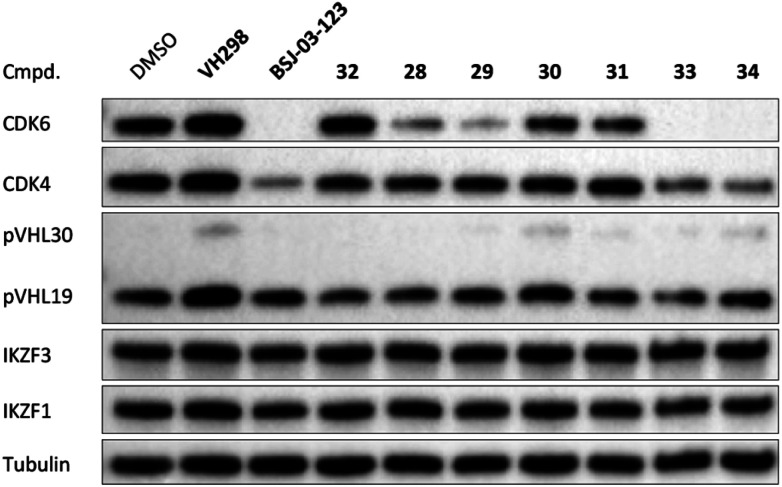
VHL-based PROTACs induce strong and selective CDK6 degradation. MM.1S cells were treated with 0.1 μM VH298 (**4**), the degrader BSJ-03-123 (**8**), negative control **32**, or PROTACs **28–31** and improved PROTACs **33–34** for 16 h.

The second ‘phenoxy’ subseries (Table 2) comprised CDK4/6d with different types of VHL ligands (Table S6, ESI†) and featured the analogous linker structures. Compounds **28–31** did not induce CDK4 depletion, but exclusively PROTAC **29** degraded CDK6 to more than 50% at a concentration of 0.1 μM. The activity of **29**, when compared with its close relative **30** was unexpected, also in the light of the same lipophilicity of both compounds (Table 2). In the subsequent optimization step, inspired by previous reports,^43^,^45^,^61^,^63^ the valine–isoindolinone moiety of **29** was replaced by *tert*-leucine acylated with a cyanocyclopropanecarbonyl (**33**) or fluoro-cyclopropanecarbonyl group (**34**). These two structural modifications led to CDK4/6d with outstanding properties, combining strong degrading potency with remarkable CDK6 selectivity, as evidenced by their selectivity indices of 46 and 31, respectively. Fig. 3 shows an exemplary western blot analysis of the CDK4 and 6 levels in MM.1S cells treated with the compounds of the ‘phenoxy’ subseries. Expectedly, VHL blockade by its ligand VH298 (**4**) did not affect CDK4/6 levels. The effective CDK6 depletion by the CRBN-based standard BSJ-03-123 (**8**) was not approached by our VHL-based PROTACs **28–31** but reached with **33** and **34**. Palbociclib and all palbociclib-based PROTACs resulted in a decrease in Rb phosphorylation regardless of their capabilities of degrading CDK4/6 (Fig. S7, ESI†). Inherent limitations of PROTAC approaches are off-target effects, which result from inevitable ligase modulation by the E3 binding component.^64^ As anticipated, none of our VHL-based CDK4/6d altered the levels of VHL, IKZF1 and IKZF3 (Fig. 3).

**Fig. 4:**
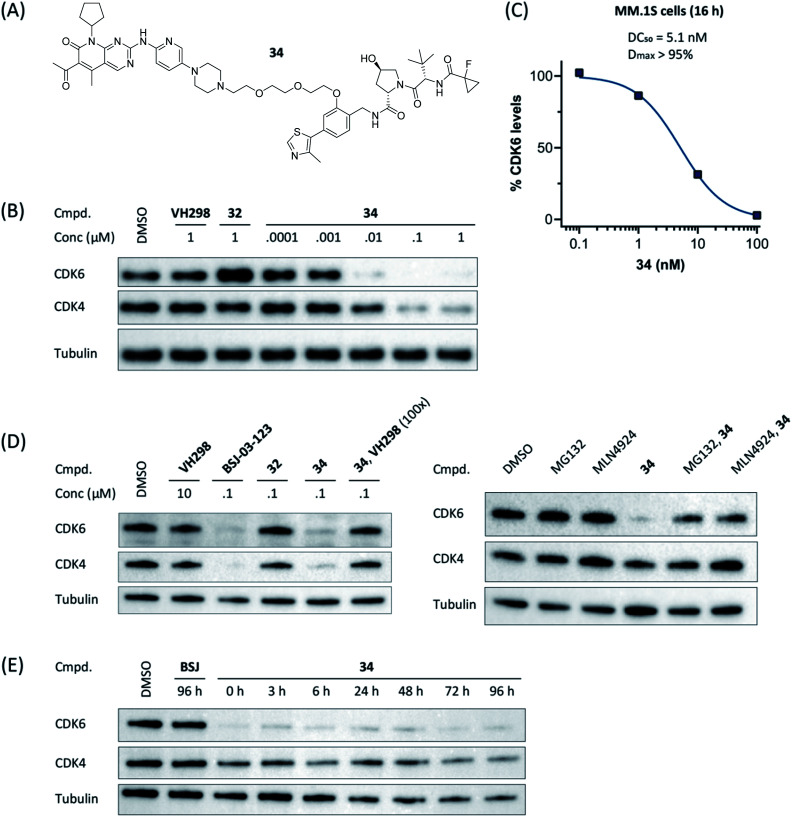
VHL-based PROTAC **34** of the ‘phenoxy’ subseries (A) induces CDK6-selective degradation in a dose-dependent manner (B). MM.1S cells were treated with 1 μM of the VHL ligand VH298, BSJ-03-123 (**8**), negative control **32**, or PROTAC **34** at the indicated concentrations for 16 h. (C) Quantification of (B) and calculation of the DC_50_-value. The value for 1 μM was excluded due to a slight hook effect. Mechanistic characterization of **34**; the activity is CRL2^VHL^- and proteasome-dependent (D). Co-treatment of **34** and VH298 (100-fold excess) abrogated CDK4/6 degradation. MG132 and MLN4924 prevented proteasomal degradation of CDK4/6. MM.1S cells were treated with MG132, MLN4924 or VH298 in the presence or absence of CDK4/6d **34** (0.1 μM). (E) Persisting effects of PROTAC **34** on CDK4/6 degradation after drug washout. MM.1S cells were treated with PROTACs at 0.1 μM for 16 h before washout with PBS (=0 h), then kept in plain media until indicated time points.

Compound **34** (Fig. 4A) was further characterized by profiling its concentration-dependent activity (Fig. 4B). In MM.1S cells, DC_50_ values of 5.1 nM (CDK6) or 20 nM (CDK4) after 16 h treatment and a maximum of CDK6 degradation of >95% at a concentration as low as 100 nM were achieved (Fig. 4C and S8, ESI†). As it was also carried out with selected PROTACs of other types, we performed competition and inhibition experiments with our lead **34**. Co-treatment of **34** and VH298 (**4**), competing with the PROTAC at the VHL binding site, diminished CDK6 degradation (Fig. 4D). To address the expected involvement of the ubiquitin–proteasome system, MM.1S cells were incubated either with MG132, a proteasome inhibitor, or MLN4924, a neddylation-activating enzyme inhibitor (Fig. 4D). In both cases, the PROTAC-induced degradation of CDK6 was prevented, clearly demonstrating that CDK6d **34** exploits the ubiquitin–proteasome pathway.

**Fig. 5:**
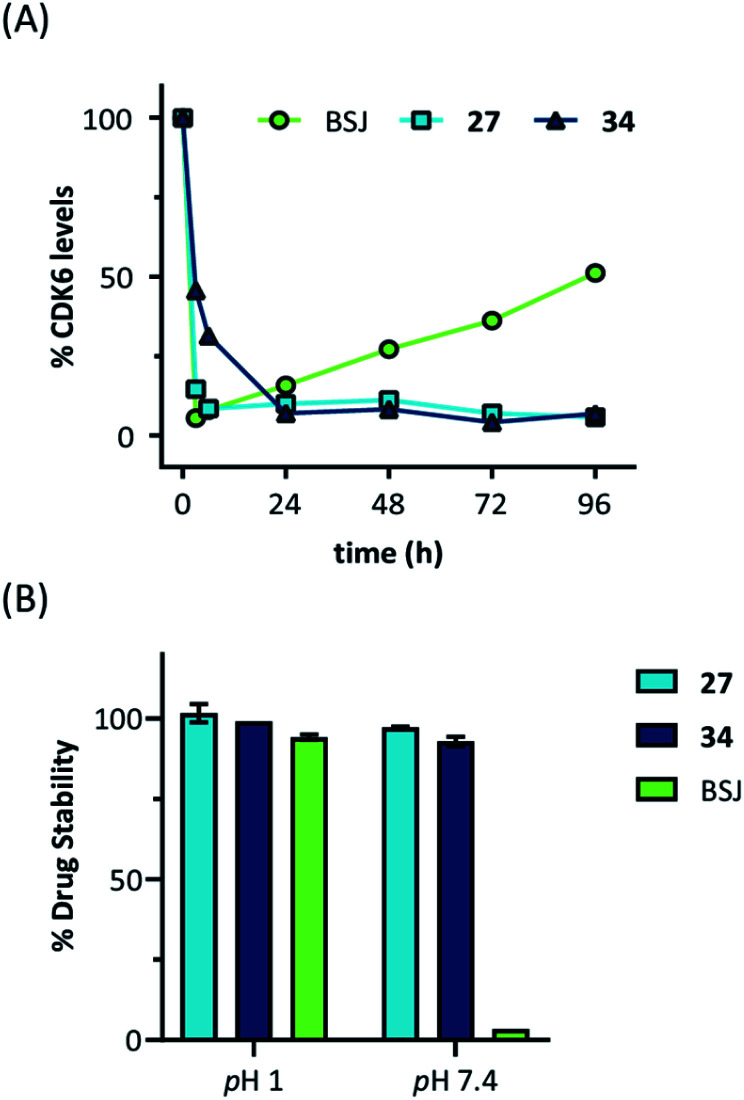
(A) Quantification of long-term treatment experiments (see Fig. S9, ESI†) and (B) drug stability data at pH 1 and pH 7.4. Acetonitrile solutions of the PROTACs were mixed with two different buffers and incubated for 24 hours at 37 °C. Subsequently, aliquots were analysed by LC/MS and normalized to acetonitrile solutions.

Next, we investigated the persistence of CDK6 degradation after single drug exposure. While CDK6 protein levels after treatment of MM.1S cells with BSJ-03-123 (**8**) began to recover after 24 h, our VHL-based PROTACs **27** and **34** achieved satisfactory CDK6 level suppression even after 96 h (Fig. S9, ESI†). The differences were even more pronounced when conducting a drug washout-step, showing that while BSJ-03-123 kept CDK6 protein below 50% for a maximum of 6 h, CDK6 degradation mediated by PROTACs **27** and **34** was more persistent and stable for up to 72 h (Fig. 4E and S10, ESI†). Similarly, washout experiments with a CRBN-based CDK6d referred to as pal-pom revealed that CDK4/6 levels were restored to their original values after 24 h.^34^ Since these degraders mainly differ in the ligase-binding part, we hypothesized that the deviations in the long-term experiments (Fig. 5A) are due to chemical inactivation of the CRBN ligand. We then tested compounds **27**, **34** (VHL-based) and BSJ-03-123 (**8**, CRBN-based) for susceptibility to hydrolysis. These PROTACs were incubated in two different buffers for 24 h at 37 °C and aliquots were analysed by LC/MS. While all three compounds were stable at pH 1, the CRBN-based PROTAC **8** showed pronounced decomposition at pH 7.4 (Fig. 5B) with masses of the main degradation peaks referring to metabolites with one or two water molecules incorporated. These LC/MS data are consistent with the known aqueous instability of thalidomide,^65^ suggesting that IMiD-type PROTACs are susceptible to hydrolytic inactivation under physiological pH value.

### Applicability in different cell lines

**Fig. 6:**
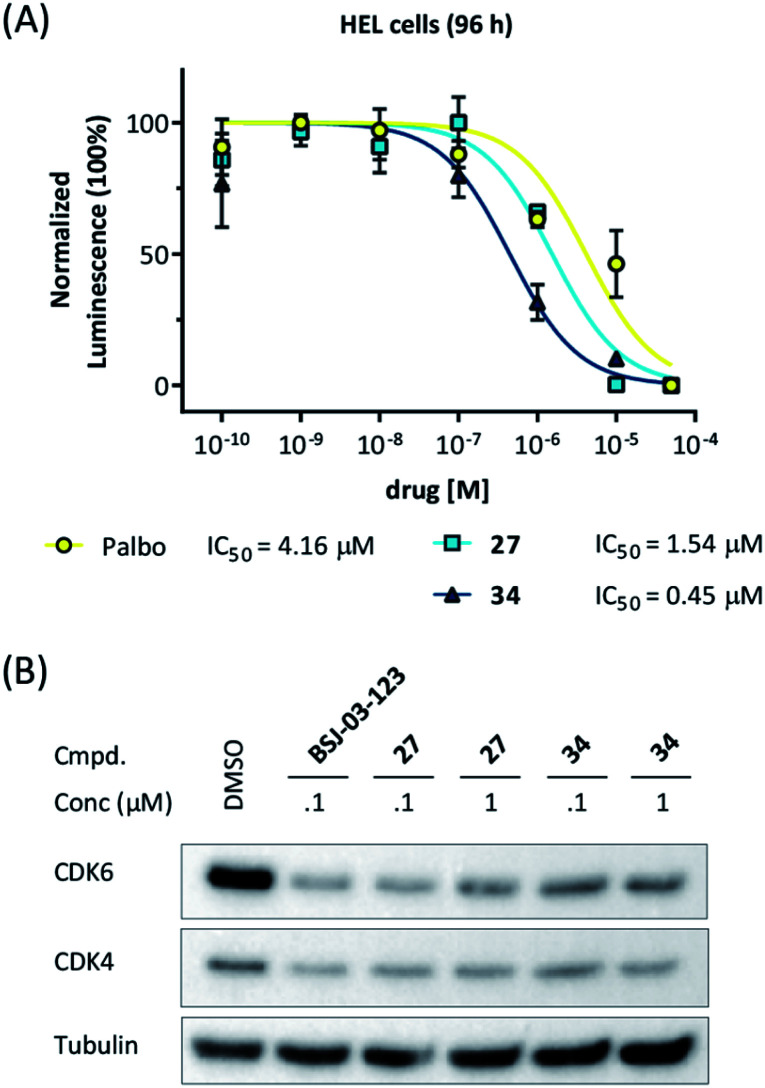
(A) CDK6d **27** and **34** show more pronounced effects on cell viability in the human HEL cells compared to palbociclib (palbo). For cell viability, cells were treated with palbo, **27**, or **34** for 96 h. (B) CDK6 degradation after treatment with PROTACs **27** and **34** for 16 h was confirmed by western blotting.

Our data obtained with the cell line MM.1S demonstrated that VHL-based compounds **27** and **34** were highly effective and selective CDK6 degraders. To further show their applicability, a panel of additional cell lines were treated with these CDK6d. We tested PROTACs **27** and **34** on various breast cancer, MM and leukemia cell lines and compared outcomes with effects caused by the CDK4/6i palbociclib. In the human MM cell lines MM.1S, LP-1, AMO-1, in the acute myeloid leukemia (AML) cell lines MOLM-13, HEL, KG-1, K562, and in the acute lymphoblastic leukemia (ALL) cell line Nalm-6, treatment with PROTACs **27** and **34** inhibited cell proliferation (Fig S11, ESI†). In the human AML cell line HEL, PROTACs **27** and **34** were even more potent than palbociclib (Fig. 6A). Western blotting confirmed the strong on-target activity of **34**. As human and murine E3 ligase VHL is not completely conserved between different species,^66^ we investigated whether the murine 32D and Ba/F3 cells constitute an appropriate model system for the effects of our VHL-based degraders. PROTAC **22** induced pronounced CDK6 degradation in both Ba/F3 and 32D cells at 1 μM, demonstrating activity in murine cells (Fig. S12, ESI†). These results confirm the applicability of our CRBN-and VHL-based PROTACs in mouse models, a prerequisite for future *in vivo* investigations of the anti-cancer effects of our CDK6d.

Palbociclib has been approved for treating patients with hormone receptor-positive and human epidermal growth factor receptor 2 negative advanced or metastatic breast cancer.^67^,^68^ To determine if the differential CDK4/6 selectivity renders palbociclib-based PROTACs differential therapeutic potential, we evaluated the impact of our PROTACs on the viability of breast cancer cell lines. MDA-MB-231 cells showed only moderate sensitivity to palbociclib with an IC_50_ of 0.26 μM, which is consistent with previous studies.^34^,^69^ The VHL-based CDK4/6 PROTACs **27** and **34** and their corresponding negative control compounds **26** and **32** had inhibitory activity comparable to palbociclib (Fig. S13A, ESI†), suggesting this impact to be governed mainly through CDK4/6 inhibition. This assumption was confirmed when investigating the compounds in the palbociclib-resistant breast cancer cell line, BT549 (Fig. S13B, ESI†). Next, we compared CRBN- and VHL-based PROTACs with respect to CDK4/6 degradation in MDA-MB-231 cells, revealing that BSJ-03-123 and **27** were more efficient than **34** (Fig. S13C, ESI†). In consistency with the observations in the MM.1S cells, our VHL-based PROTACs had stronger effects on CDK6 than CDK4.

After we had demonstrated that PROTAC **34** induced degradation of CDK6 without affecting MDA-MB-231 cell viability at the effective concentration of 0.1 μM, we investigated whether PROTAC-mediated CDK6 knockdown has additional effects on cell phenotypes. Cell migration is declined both in cells with shRNA-mediated CDK4/6 knockdown and CDK4/6 inhibition by palbociclib.^70^,^71^ We employed a wound-healing assay in order to analyse the impact of PROTAC **34** on cell migration, in comparison with palbociclib and the VHL ligand VH298 (Fig. S14, ESI†). Quantification of the wound closure revealed that both CDK6d **34** and CDK4/6i palbociclib significantly impaired cell migration and resulted in a reduction of wound healing by 29% and 17%, respectively. While CDK6d **34** performed slightly better than palbociclib, the monomeric VHL ligand VH298 (**4**) did not affect the ability of MDA-MB-231 cells to migrate.

### Extensibility to other ligases

**Fig. 7:**
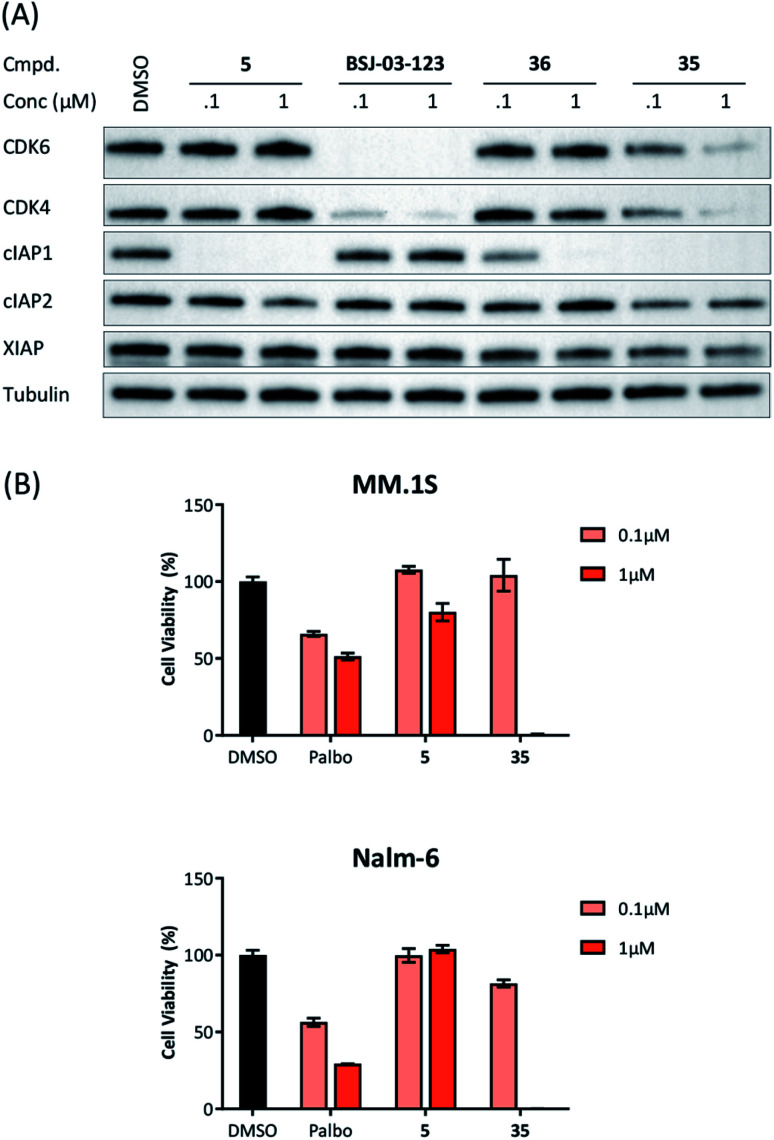
(A) IAP-based degrader **35** induces CDK4/6 degradation in a dose-dependent manner. MM.1S cells were treated with the IAP ligand **5**, the CDK6-selective degrader BSJ-03-123, negative control **36**, or degrader **35** at the indicated concentrations for 16 h. In addition to the target proteins CDK4/6, the expression levels of relevant members of the inhibitor of apoptosis (IAP) protein family were blotted; (B) IAP-based PROTAC **35** is more as potent as palbociclib (palbo) and ligand **5** on decreasing cell viability in the multiple myeloma cell line MM.1S and the acute lymphoblastic leukemia cell line Nalm-6. Cells were treated with palbo, **5**, or **35** for 96 h at 0.1 μM or 1 μM. All results were normalized to non-treated conditions and data represent mean ± SD of biological triplicates.

Recent work demonstrated that investigating different combinations of E3 and target recruiting elements is vitally important to tune degrader activity and selectivity.^72^ Furthermore, in regard to clinical applications of PROTACs in cancer, genetic inactivation of E3 ligase components displays a particular vulnerability to develop resistance.^38^,^60^ These issues provided us with a rationale for expanding the CDK4/6d toolbox to additional E3 ligases. To address the PROTACability of CDK4/6 *via* non-CRL ligases, the IAP-based degrader **35** and the MDM2-based PROTAC **37** were synthesized (Tables 3 and S5, ESI†). Compound **35**, but not its monovalent progenitor **5** (Fig. 1), was able to degrade CDK4 and CDK6 at concentrations as low as 0.1 μM and with particularly pronounced effects at 1 μM (Fig. 7A). In contrast to CRBN- and VHL-based PROTACs, CDK6 was not preferentially diminished when using the IAP-based degrader **35**. These results demonstrate that activity and selectivity can be modulated not only by tailored modifications in the target ligand and linker portion,^35^ but also by choosing different E3-recruiting elements.^73^ The degrader-induced knockdown of cIAP1 is a well-known phenomenon of IAP inhibitors and IAP-based heterobifunctional compounds^25^ and was also observed with our chimaeras. IAP inhibition has been shown to induce apoptosis in cancer cells and pursued as a treatment for cancer.^25^ Therefore, the induced degradation of the E3 ligase cIAP1 itself may contribute to the anti-tumour effects of IAP-based degraders. Consistently, cell viability after treatment with IAP-based degrader **35** was more strongly impaired in comparison to palbociclib and PROTACs from the VHL series, as well as the IAP ligand **5** (Fig. 7B). The negative control **36**, which contains a deactivated IAP ligand, failed to degrade CDK4 and CDK6 at a concentration of 1 μM (Fig. 7A).

The MDM2-based compound **37** was unable to induce CDK4/6 degradation at 0.1 and 1 μM (Fig. S15, ESI†). As this compound displayed extremely high lipophilicity (Table S5, ESI†) and did not induce stabilization of p53 and its downstream effector protein p21 (Fig. S15, ESI†), we concluded that poor cell permeability hampered cellular effects. Further research into appropriate palbociclib/linker/MDM2-ligand combinations to achieve acceptable physicochemical properties will be necessary. Interestingly, we observed that control treatment with the MDM2i idasanutlin displayed dose-dependent effects on CDK4 levels (Fig. S15, ESI†). The protein p21^CIP^ was characterized as a CDK inhibitor at high p21 protein levels.^74^ Although it has been discovered, that MDM2i synergistically work with CDK4/6i,^75^ direct effects on the CDK4/6 protein levels have not been described yet. To shed light on the underlying mechanism, we investigated if CDK4/6 degradation is directly proportional to p53/p21 levels. For this purpose, AMG232, currently the most potent inhibitor of the MDM2-p53 interaction, was used, as well as a newly synthesized putative MDM2 degrader **95** (Table S4, ESI†). Previous studies revealed that CRBN-based MDM2-degraders are highly effective in inducing activation of p53.^76^ Both the MDM2i and PROTAC **95** were able to stabilize p21 and diminished CDK4 levels in a dose-dependent manner, similar than idasanutlin did (Fig. S16, ESI†). In contrast, no significant changes in CDK4/6 abundance were observed in the p53-null CML cell line K562 implying CDK4/6 downregulation is a downstream effect of p53 activation (Fig. S17, ESI†). Given that any MDM2-based compound might possess additional biological activities by p53/p21 activation,^50^,^77^ CDK4/6 downregulation should be considered as a relevant off-target effect of heterobifunctional MDM2 degraders.

## Conclusions

**Fig. 8:**
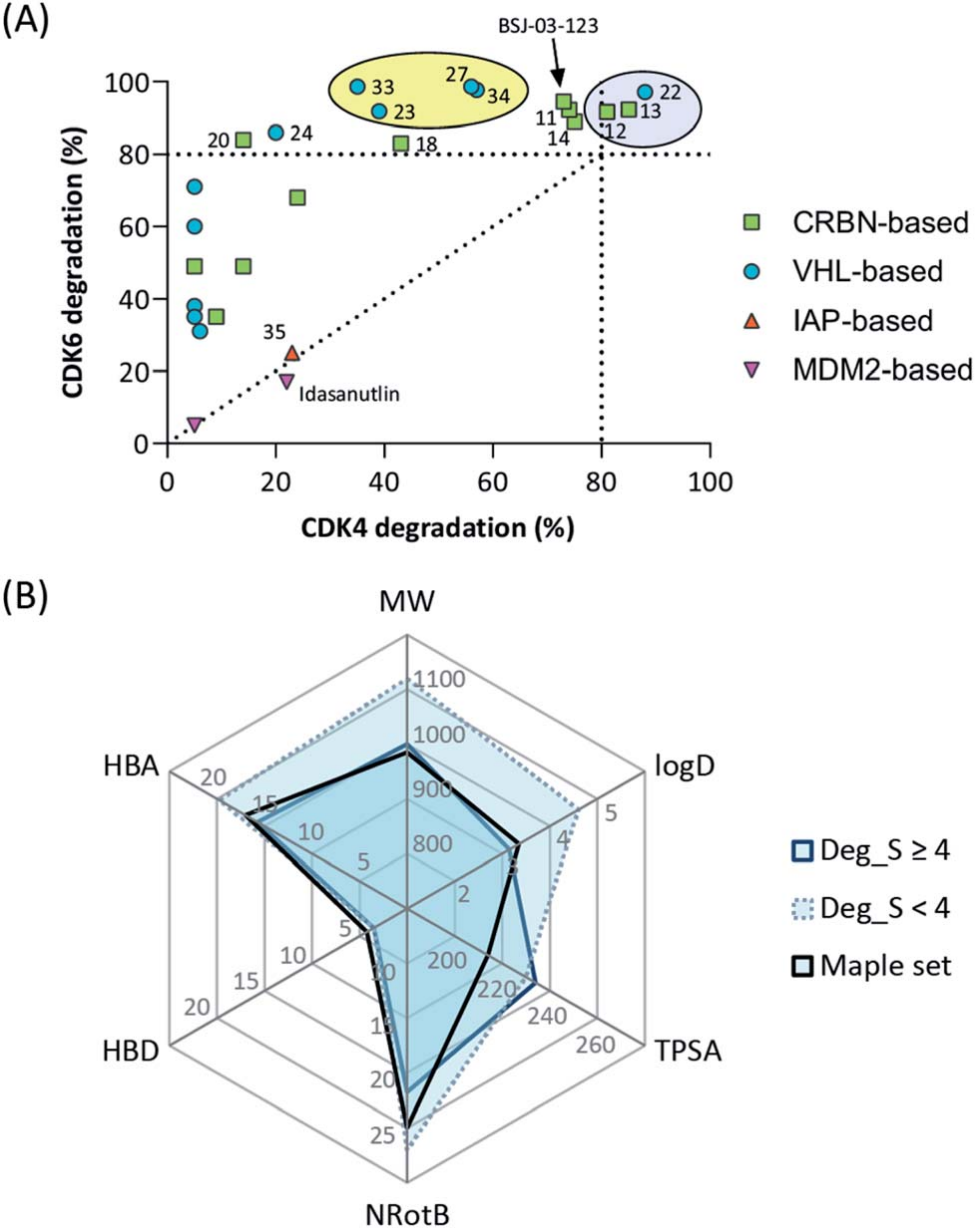
(A) Selectivity profile of CDK4/6d hijacking four different E3 ligases. (B) Radar plot of molecular descriptors of highly active and less active CDK6 degrading PROTACs (see Tables S1–S4, ESI†). Average values for the recently published analysis of more than 400 degraders (‘Maple set’) are given.

The work reported herein represents a thorough exploration of CDK4/6 degradation space through the consideration of four different E3 ligases for the assembly of final PROTAC molecules. PROTAC representatives utilizing three out of four ligases were capable of inducing tripartite binding as a prerequisite for proteasomal degradation of CDK4/6. We generated, for the first time, highly active VHL-based PROTACs with considerable CDK6 selectivity in a broad range of different human and murine cancer cells. We also discovered a highly active IAP-based degrader that induced the degradation of CDK4/6 as well as the IAPs themselves, which may facilitate killing cancer cells that require IAPs for survival. Furthermore, a new mechanism was unravelled by which MDM2 inhibitors and degraders lead to diminished CDK4 levels in a dose-dependent and p21-proportional manner. Our comprehensive set of PROTACs (Fig. 8A) may contribute to medicinal chemistry rules for successful degrader design. Out of the series of test compounds, three potent dual CDK4/6 PROTACs and four VHL-based PROTAC molecules with a desired CDK6 selectivity were identified. Different physicochemical properties and molecular descriptors were plotted in a radar chart to analyse multivariate data and unravel the activity-determining features of our CDK4/6 PROTACs (Fig. 8B). The identified lead compounds were further investigated in AML, ALL, breast cancer, and murine cells. Moreover, we demonstrated that CDK4/6d **34** can block CDK's kinase signalling and moonlighting functionalities at the same time. An interference with kinase-independent functions of CDK4/6 constitutes a particular opportunity of such degraders. The CDK6-selective PROTAC **34** (CST651) raises the intriguing possibility to assemble the mosaic of CDK6-specific functions *via* a PROTAC-mediated knockdown. Our degraders represent multipurpose tools to study CDK6 biology in even greater detail and may translate to new therapies in cancer.

## Conflicts of interest

There are no conflicts to declare.

## Supplementary Material

Supplementary information
