# Supplementary material for: Systematic exploration of different E3 ubiquitin ligases: an approach towards potent and selective CDK6 degraders
Source: Chem Sci. 2020 Mar 4;11(13):3474–86. doi: 10.1039/d0sc00167h (PMC7552917; doi:10.1039/d0sc00167h)
Supplement: Supplementary file 1 [file SC-011-D0SC00167H-s001.pdf]

Electronic Supplementary Material (ESI) for Chemical Science.
This journal is © The Royal Society of Chemistry 2020

Supplementary information

Systematic Exploration of Different E3 Ubiquitin Ligases: An Approach Towards Potent and Selective CDK6 Degraders

Christian Steinebach,[#,a] Yuen Lam Dora Ng,[#,b] Izidor Sosič,[c] Chih-Shia Lee,[d] Sirui Chen,[b] Stefanie Lindner,[b] Lan Phuong Vu,[a] Aleša Bricelj,[c] Reza Haschemi,[e] Marius Monschke,[f] Elisabeth Steinwarz,[e] Karl G. Wagner,[f] Gerd Bendas,[e] Ji Luo,[d] Michael Gütschow*,[a] and Jan Krönke*,[b]

[a.] Pharmaceutical Institute, Department of Pharmaceutical & Medicinal Chemistry, University of Bonn, An der Immenburg 4, 53121 Bonn, Germany.

[b.] Department of Internal Medicine III, University Hospital Ulm, Albert-Einstein-Allee 23, 89081 Ulm, Germany.

[c.] Faculty of Pharmacy, University of Ljubljana, Aškerčeva cesta 7, 1000 Ljubljana, Slovenia.

[d.] Laboratory of Cancer Biology and Genetics, Center for Cancer Research, National Cancer Institute, Bethesda, MD 20892, United States.

[e.] Pharmaceutical Institute, Department of Pharmaceutical & Cell Biological Chemistry, University of Bonn, An der Immenburg 4, 53121 Bonn, Germany.

[f.] Pharmaceutical Institute, Pharmaceutical Technology, University of Bonn, Gerhard-Domagk-Straße 3, 53121 Bonn, Germany.

[#]These authors contributed equally.

Table of Content

Supplementary Schemes, Tables and Figures....3

Supplementary Information: Biology.... 27

A. Cell Lines.... 27

B. CRISPR-mediated Knockout of CRBN and VHL.... 27

C. Immunoblotting.... 28

D. Cell Viability Assay.... 29

E. Cell Migration Assay.... 29

F. Statistical Analysis.... 29

Supplementary Information: Chemistry.... 30

G. General Remarks.... 32

H. Synthesis of CRBN-based PROTACs.... 33

J. Synthesis of VHL-based PROTACs....45

K. Synthesis of IAP-based degraders.... 77

L. Synthesis of MDM2-based PROTACs....85

M. Selected Analytical Spectra....90

Supplementary Information: Physical Chemistry.... 93

N. Molecular Descriptor Calculation.... 93

O. Physicochemical Measurements.... 93

References.... 95

Supplementary Schemes, Tables and Figures

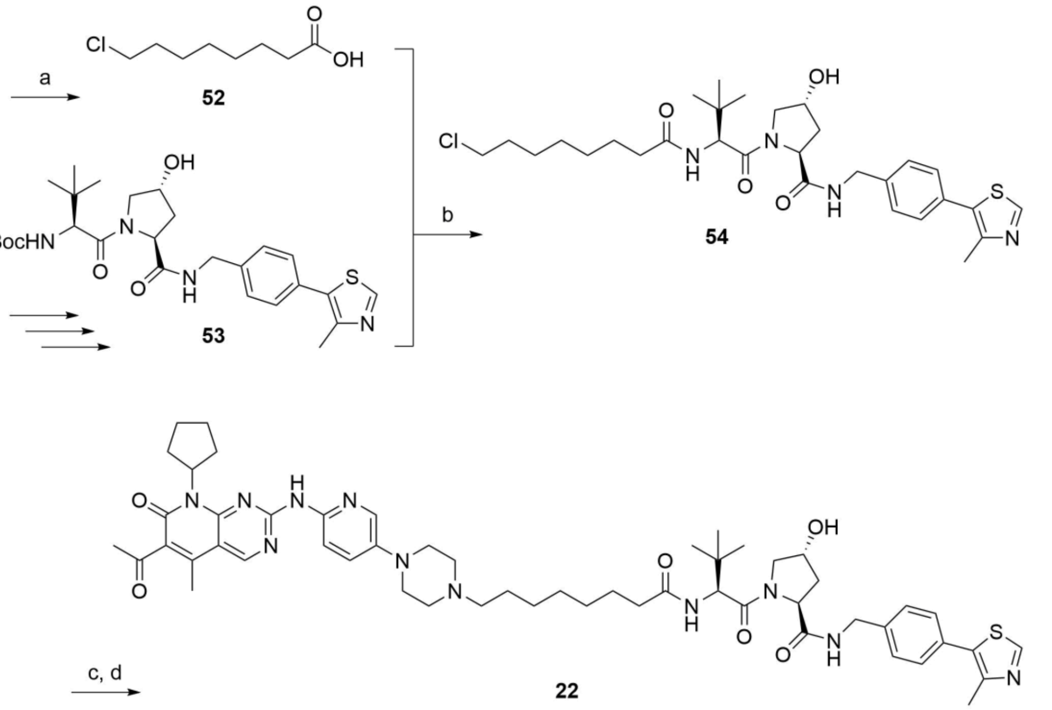

Scheme S1 Synthesis of the VHL-addressing CDK4/6 degrader **22**. Reagents and conditions: (a) 8-Chloro-1-octanol, BAIB/TEMPO, MeCN/H_2O, rt, 16 h; (b) HATU, DIPEA, DMF, rt, 16 h; (c) NaI, acetone, 60 °C, 48 h; (d) DIPEA, DMSO/DMF, 80 °C, 24 h.

Cmpd	Deg_S (CDK6)	MW [g/mol]	elogD	TPSA [A^2]	NRotB	HBD	HBA	Structure
**7**	5.7	936	2.7	247	21	4	17	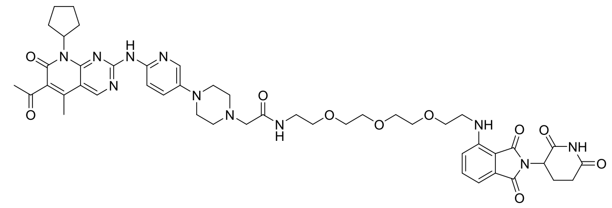
**8**	5.7	937	2.5	244	21	3	17	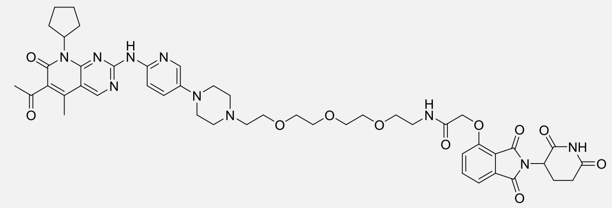
**11**	4.0	817	3.4	207	13	3	13	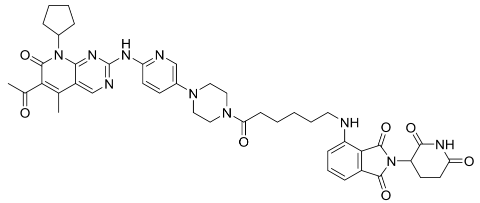
**12**	4.7	849	2.8	226	15	3	15	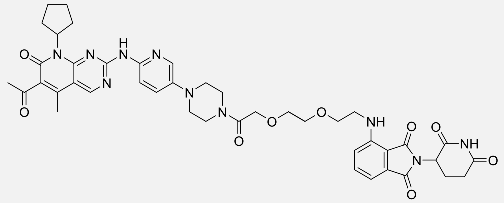

**13**	4.7	893	2.8	235	18	3	16	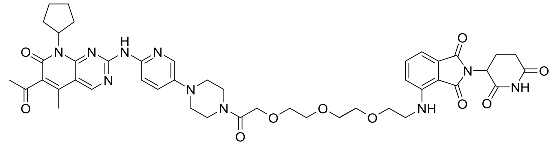
**14**	4.7	935	3.1	235	21	3	16	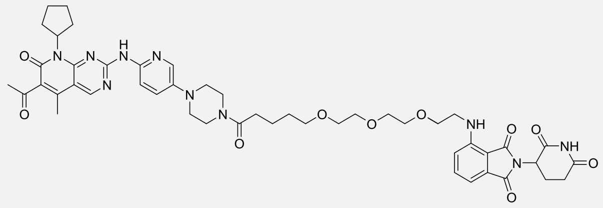
**15**	3.3	990	3.1	255	23	4	16	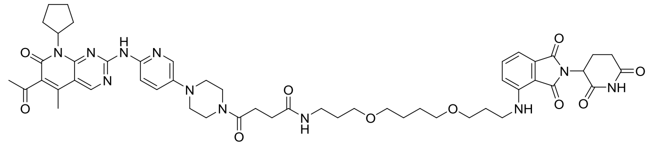
**16**	3.3	989	4.6	226	25	3	15	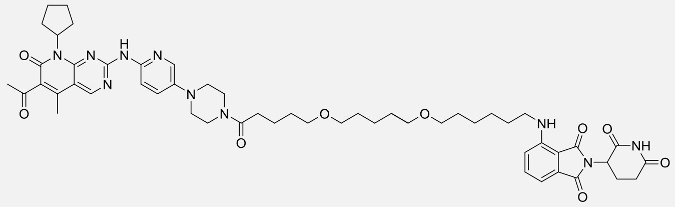
**17**	3.3	1017	5.2	226	27	3	15	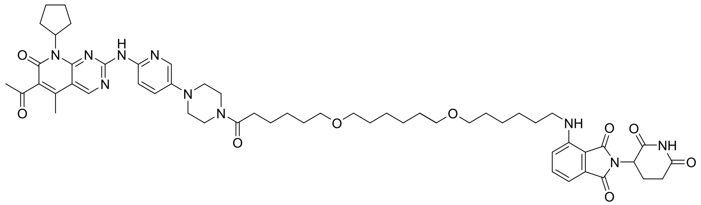

**18**	4.0	1033	4.4	235	28	3	16	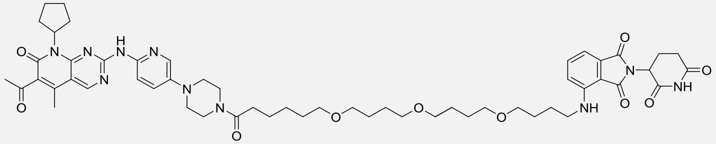
**19**	3.3	1133	5.4	244	35	3	17	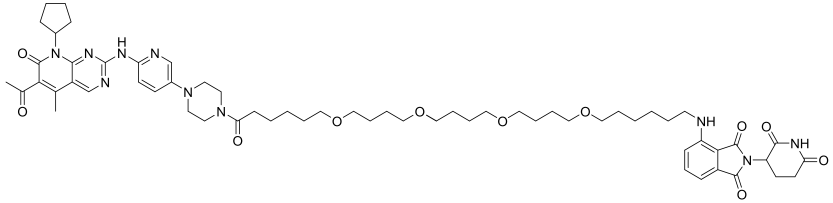
**20**	4.0	1137	3.7	263	35	3	19	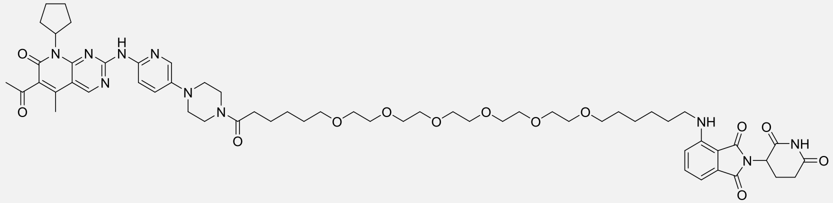
**21**	n.d.	831	3.6	198	13	2	13	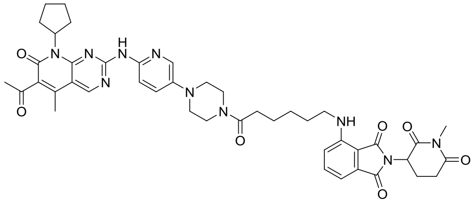

Table S1 Overview on synthesized CRBN-based CDK-PROTACs. Degrader score (Deg_S),[1] topological polar surface area (TPSA), number of rotatable bonds (NRotB), hydrogen bond donors (HBD), hydrogen bond acceptors (HBA)

Cmpd	Deg_S (CDK6)	MW [g/mol]	elogD	TPSA [A^2]	NRotB	HBD	HBA	Structure
**22**	4.7	1002	3.3	206	20	4	13	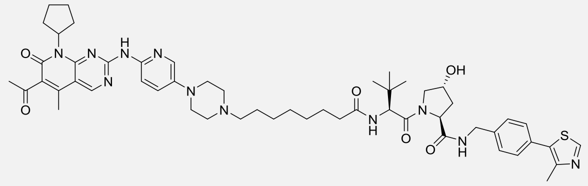
**23**	4.7	1006	2.7	225	20	4	15	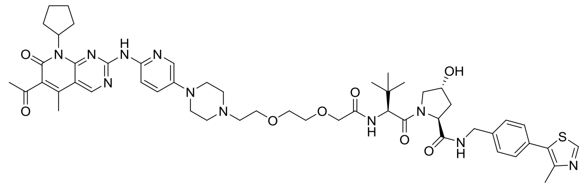
**24**	4.0	1050	3.5	234	23	4	16	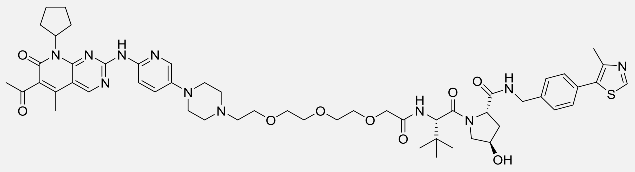
**25**	3.3	1147	4.8	225	30	4	15	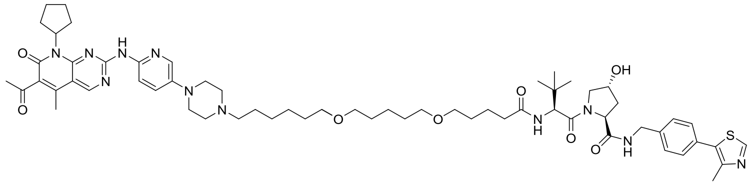

**26**	n.d.	1002	3.2	206	20	4	13	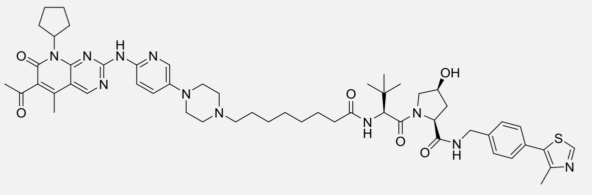
**27**	7.0	1064	2.9	234	23	4	16	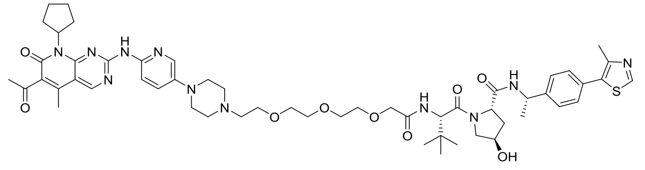
**28**	3.3	1106	4.5	207	22	3	14	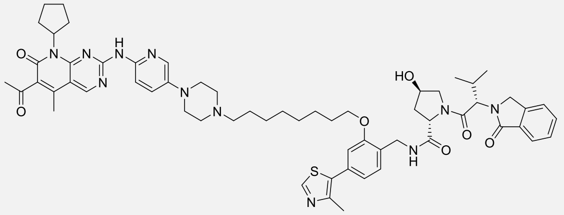
**29**	4.0	1110	3.2	225	22	3	16	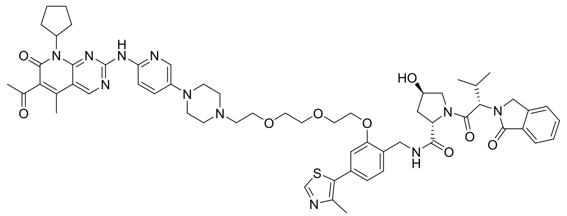
**30**	3.3	1154	3.1	234	25	3	17	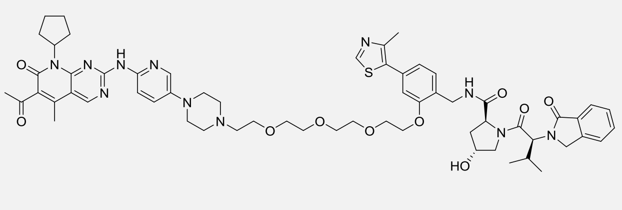

**31**	3.3	1251	5.3	225	32	3	16	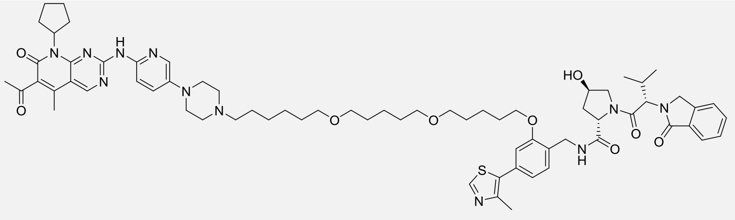
**32**	n.d.	1110	3.0	225	22	3	16	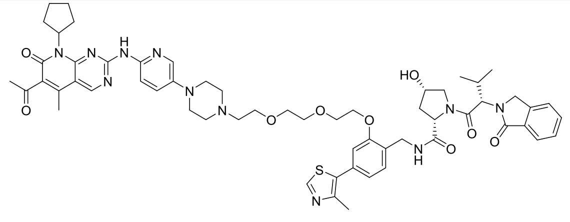
**33**	4.7	1101	3.0	258	23	4	17	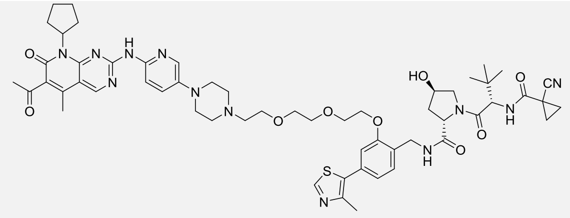
**34**	7.0	1094	3.1	234	23	4	16	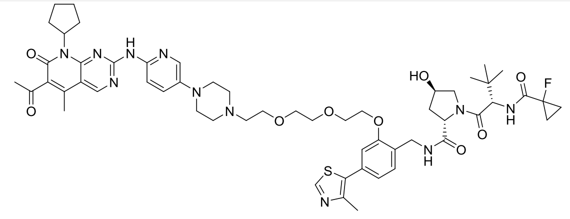

Table S2 Overview on synthesized VHL-based CDK-PROTACs. Degrader score (Deg_S),[1] topological polar surface area (TPSA), number of rotatable bonds (NRotB), hydrogen bond donors (HBD), hydrogen bond acceptors (HBA)

Cmpd	Deg_S (CDK6)	MW [g/mol]	elogD	TPSA [A^2]	NRotB	HBD	HBA	Structure
**35**	3.0	1183	4.3	231	27	4	17	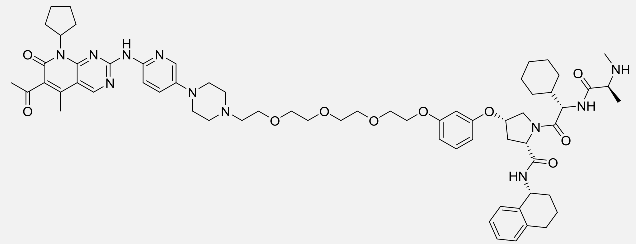
**36**	n.d.	1183	4.5	231	27	4	17	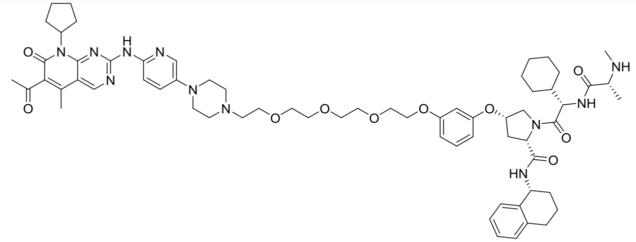

Table S3 Overview on synthesized IAP-based CDK-degraders. Degrader score (Deg_S),[1] topological polar surface area (TPSA), number of rotatable bonds (NRotB), hydrogen bond donors (HBD), hydrogen bond acceptors (HBA)

Cmpd	Deg_S (CDK6)	MW [g/mol]	elogD	TPSA [A^2]	NRotB	HBD	HBA	Structure
**37**	0.0	1221	5.8	226	25	4	16	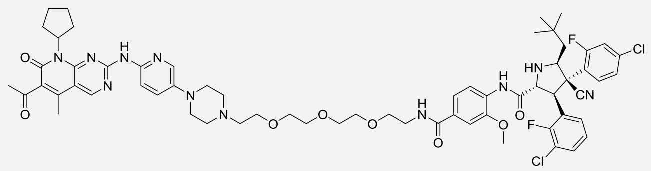
**95**	n.d.	1061	3.6[a]	244	21	5	13	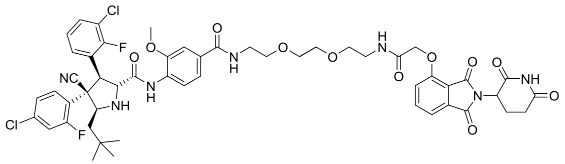

Table S4 Overview on synthesized MDM2-based PROTACs. [a]Calculated value. Degrader score (Deg_S),[1] topological polar surface area (TPSA), number of rotatable bonds (NRotB), hydrogen bond donors (HBD), hydrogen bond acceptors (HBA)

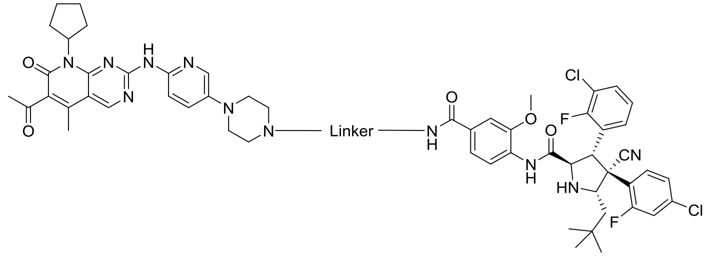

Cmpd.	Linker	elog $D_{7.4}$ [a]	D_{CDK4} [b]		D_{CDK6} [b]		$\frac{D_{CDK4}}{D_{CDK6}}$ [c]
			0.1 µM	1 µM	0.1 µM	1 µM	0.1 µM
**6** (Idasanutlin)	--	n.d.[d]	78	40	83	70	0.9
**8** (BSJ-03-123)	--	2.5	27	n.d.	5.5	2.1	4.9
**37**	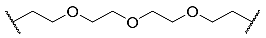	5.8	>95	>95	>95	>95	n.d.

Table S5 Activities of the MDM2-addressing CDK4/6 degrader. [a]Experimental distribution coefficient at *p*H 7.4. [b]CDK4 or CDK6 degradation indicated as remaining CDK4 or CDK6 levels after 16 h treatment of each compound at the indicated concentration. Percentage values are normalized to DMSO-treated MM.1S cells and the respective loading controls (100%). All of the data were the average of at least three independent experiments. [c]Selectivity ratio for the degradation of CDK6 over CDK4. [d]Not determined.

‘amide’ subseries	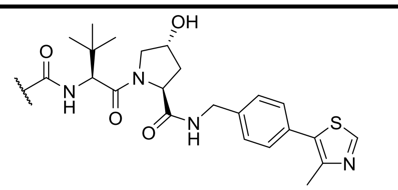<br>VHL ligand a	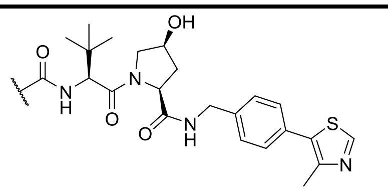<br>(–)VHL ligand a
	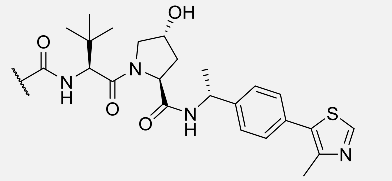<br>VHL ligand b	--
‘phenoxy’ subseries	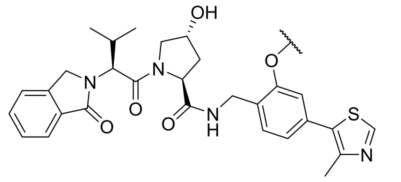<br>VHL ligand c	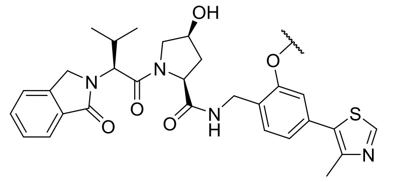<br>(–)VHL ligand c
	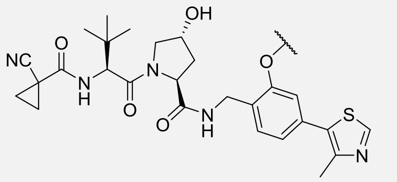<br>VHL ligand d	--
	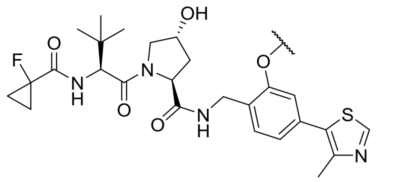<br>VHL ligand e	--

Table S6 Overview on different VHL ligands incorporated into final PROTACs (see also Table 2 and Table 3, respectively).

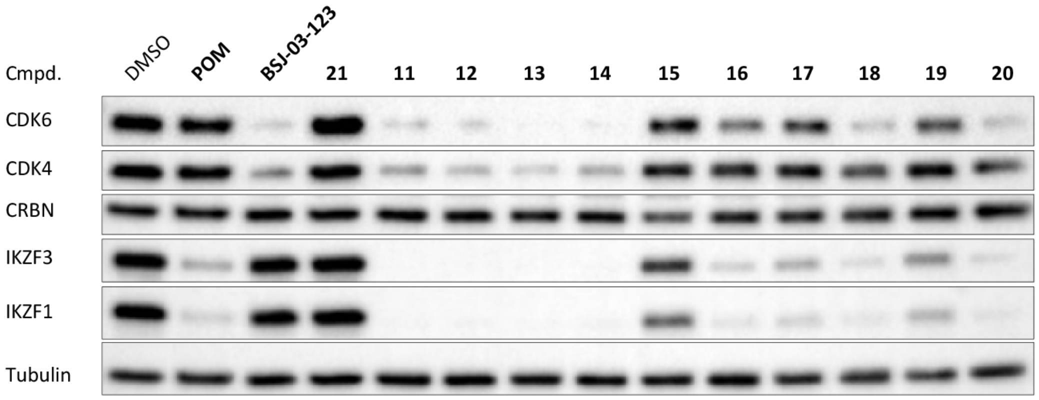

Fig. S1 Amide-connected CRBN-addressing PROTACs degrade CDK4/6. MM.1S cells were treated with 0.1 µM pomalidomide (**3**, POM), the CDK6-selective degrader BSJ-03-123 (**8**),[2] negative control **21**, or PROTACs **11**-**20** for 16 h. In addition to the target proteins CDK4/6, the expression levels of CRBN as well as the neo-substrates IKZF1 and IKZF3 were blotted.

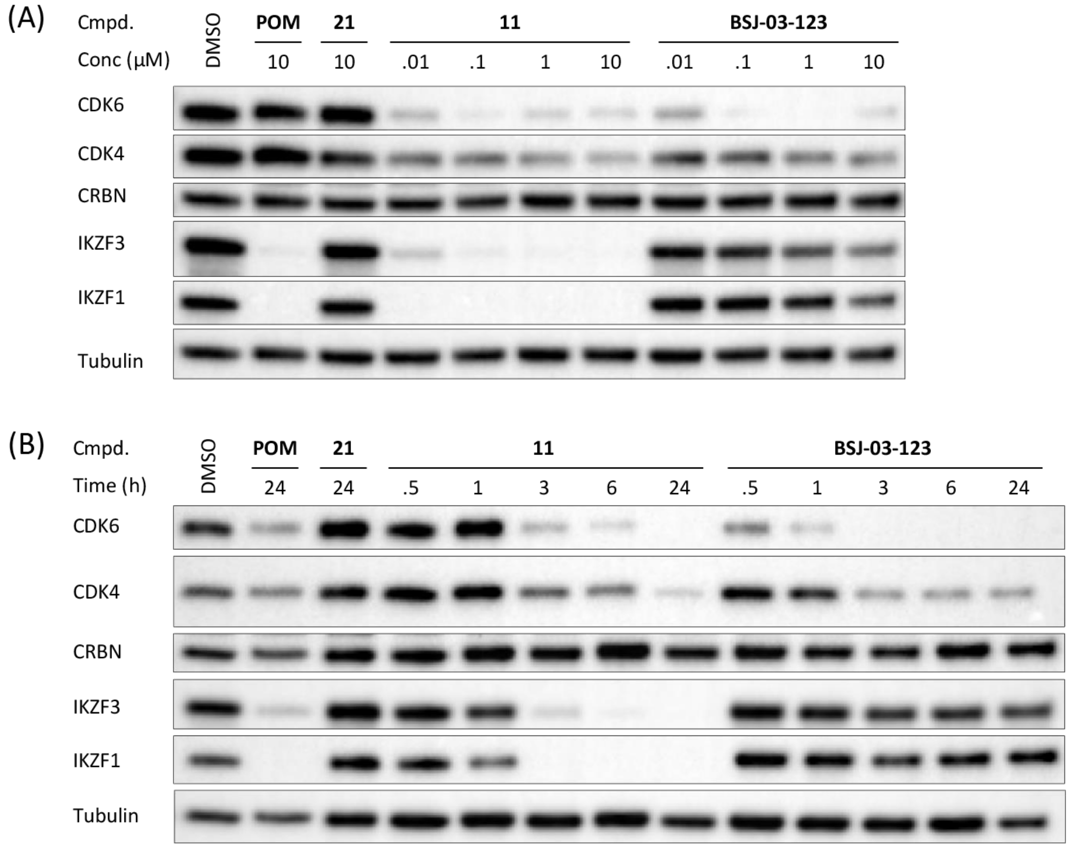

Fig. S2 (A) CRBN-based PROTAC **11** induces CDK6-selective degradation in a dose-dependent manner. MM.1S cells were treated at indicated concentrations for 16 h. The CDK6-selective degrader BSJ-03-123 (**8**) was used as a standard; (B) Time course experiment with CDK4/6d **11** versus BSJ-03-123. MM.1S cells were treated at 0.1 µM for the indicated time.

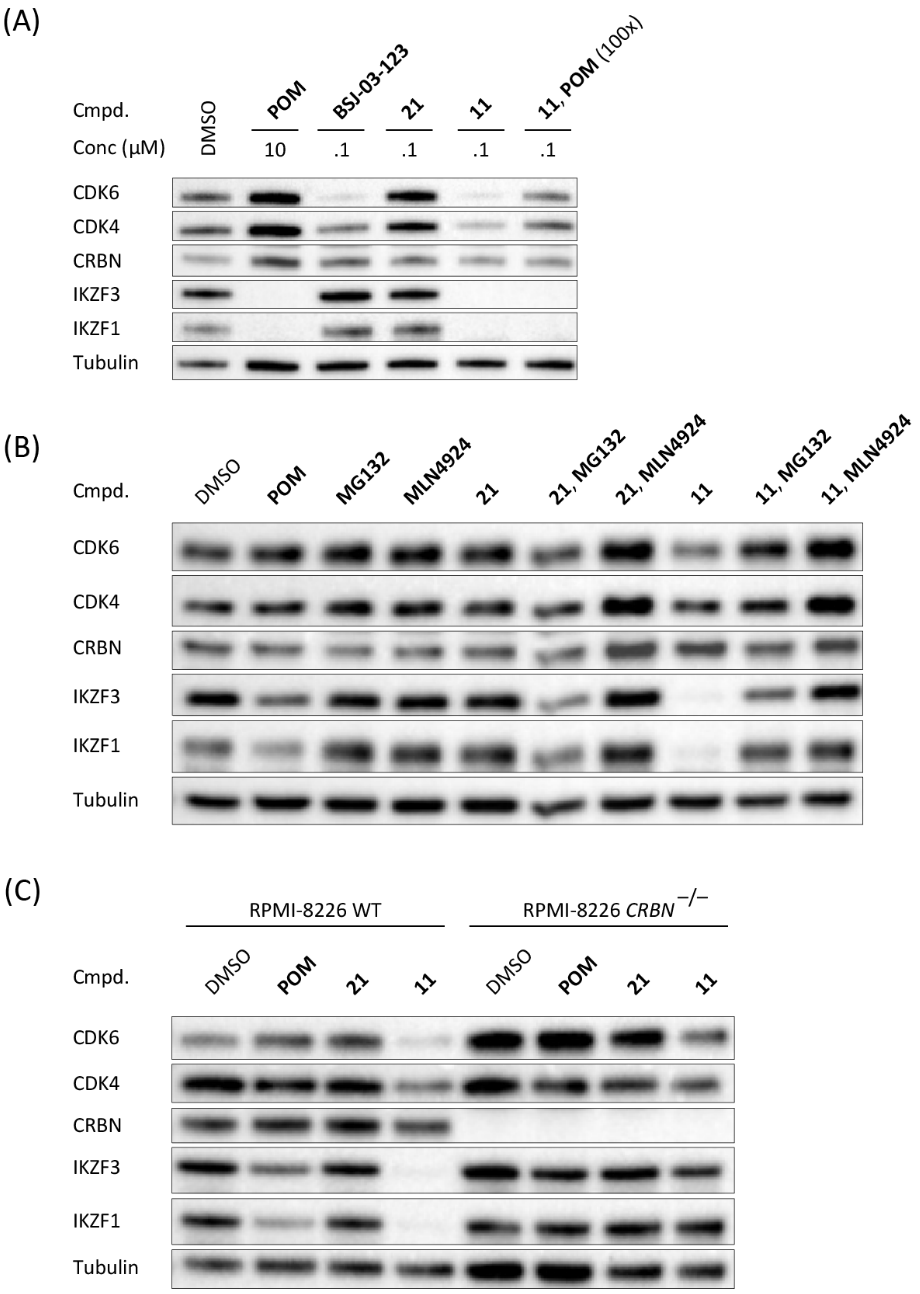

Fig. S3 CDK4/6 degradation induced by PROTAC **11** is mediated *via* the E3 ubiquitin ligase CRL4^{CRBN} and subsequent proteasomal degradation. (A) MM.1S cells were treated for 16 h with vehicle, pomalidomide (**3**, POM), the CDK6-selective degrader BSJ-03-123 (**8**), negative control **21**, or PROTAC **11** at the indicated concentrations. Co-treatment of **11** and POM (100fold excess) significantly reduced the effects on CDK4/6; (B) MM.1S cells were treated with 10 µM of the proteasome inhibitor MG132, 10 µM of the NEDD8-activating enzyme inhibitor MLN4924 or 0.1 µM PROTACs alone for 3 h or cells were pre-treated with inhibitors MG132 / MLN4924 for 1 h before addition of PROTACs for additional 3 h; (C) RPMI-8226 wild-type or RPMI-8226 *CRBN*$^{-/-}$ cells were treated with compounds at 0.1 µM for 16 h.

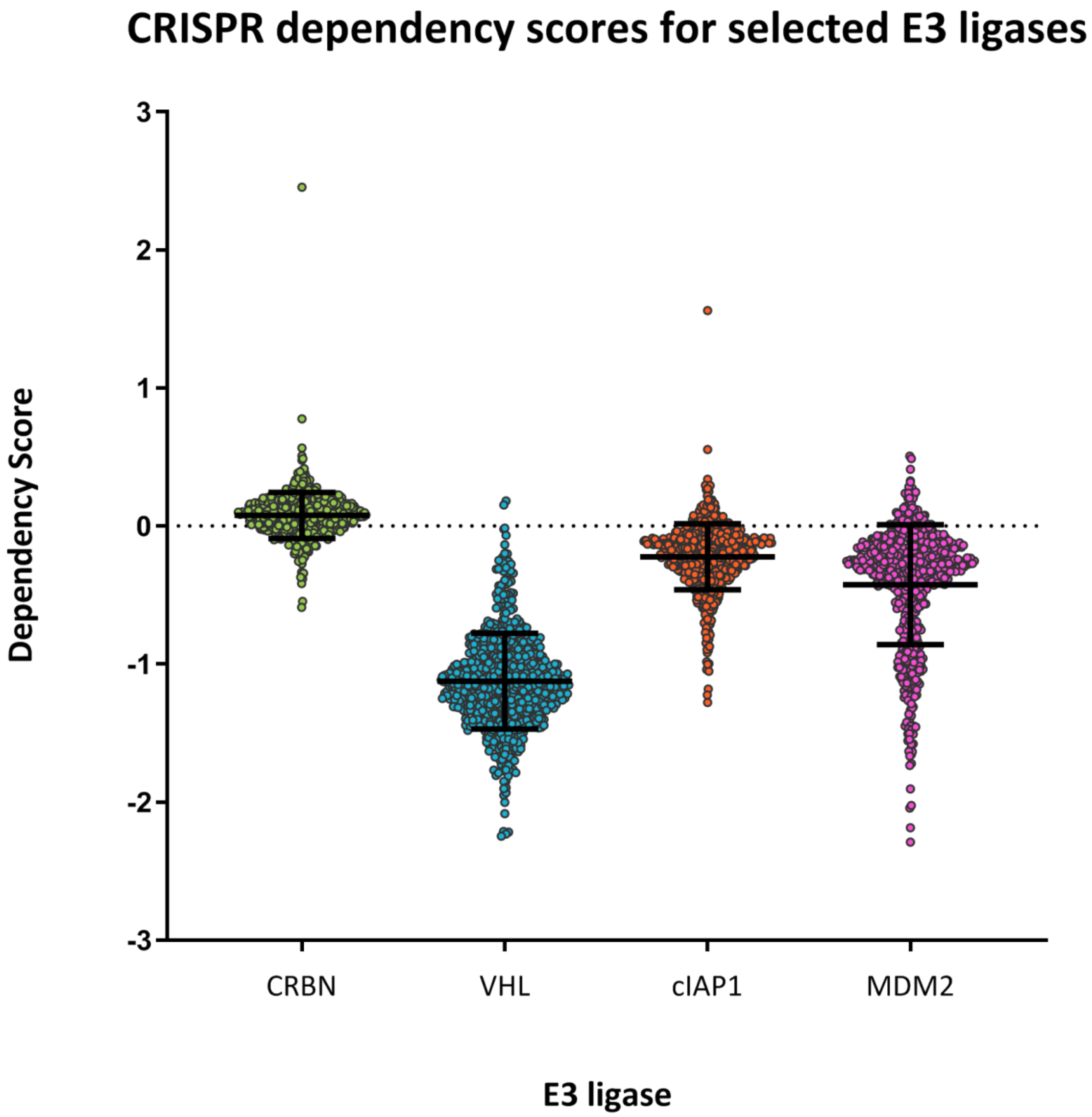

Fig. S4 CRISPR dependency scores for selected E3 ligases. CRISPR dependency scores from the DepMap database representing specific genetic vulnerabilities of cancer cells.[3–5] Lower values indicate higher relative deleteriousness for cell growth.

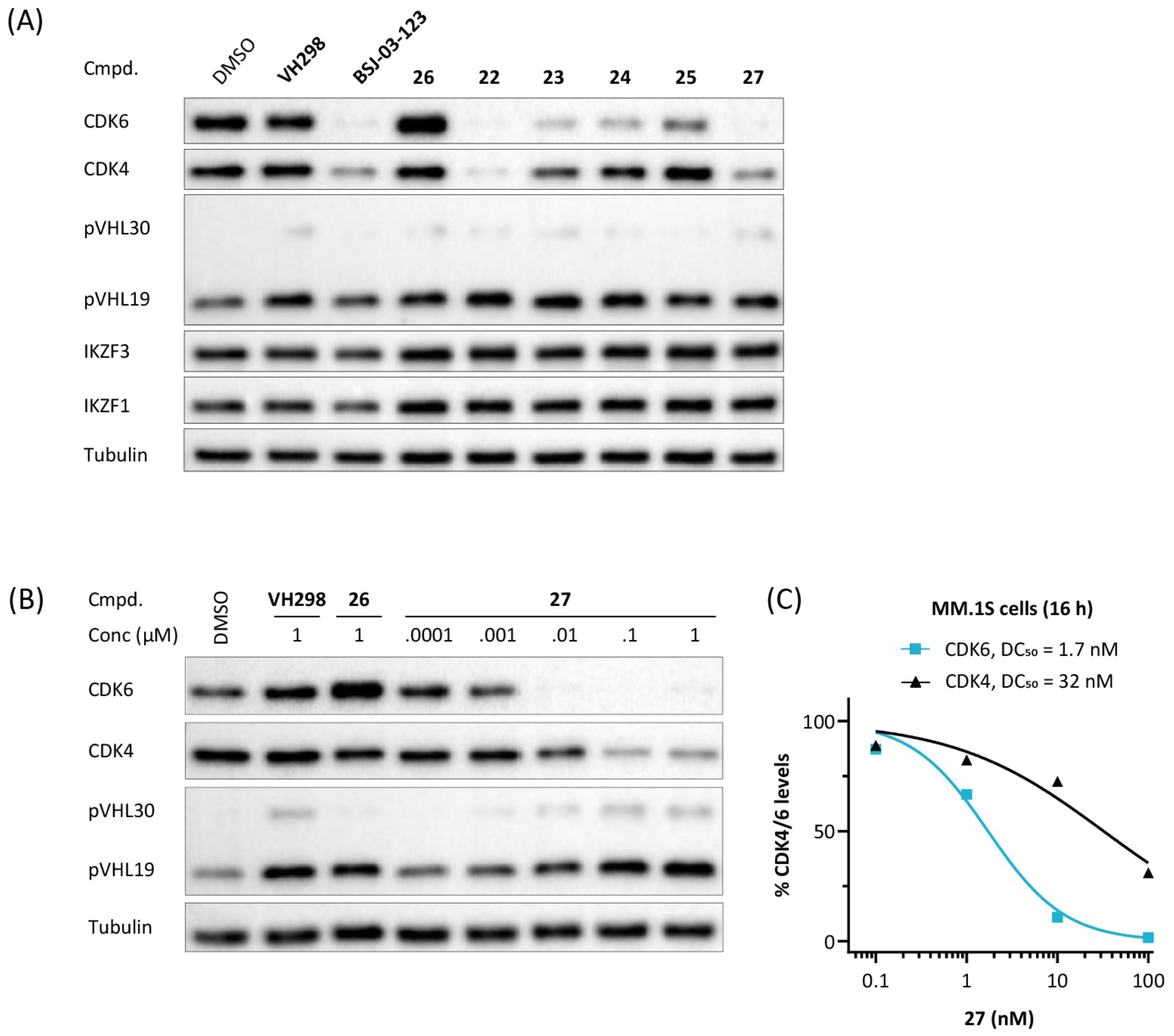

Fig. S5 VHL-based PROTACs of the ‘amide’ subseries degrade CDK4/6. (A) MM.1S cells were treated with 0.1 µM of the VHL ligand VH298 (**4**), BSJ-03-123 (**8**), negative control **26**, or PROTACs **22**-**25** and **27** for 16 h; (B) PROTAC **27** induces CDK6-selective degradation in a dose-dependent manner. MM.1S cells were treated with compounds for 16 h; (C) Quantification of (B) and calculation of the DC_{50}-value; (D) Time course experiment with the CDK6-selective, VHL-based PROTAC **27**. MM.1S cells were treated at 0.1 µM for the indicated time.

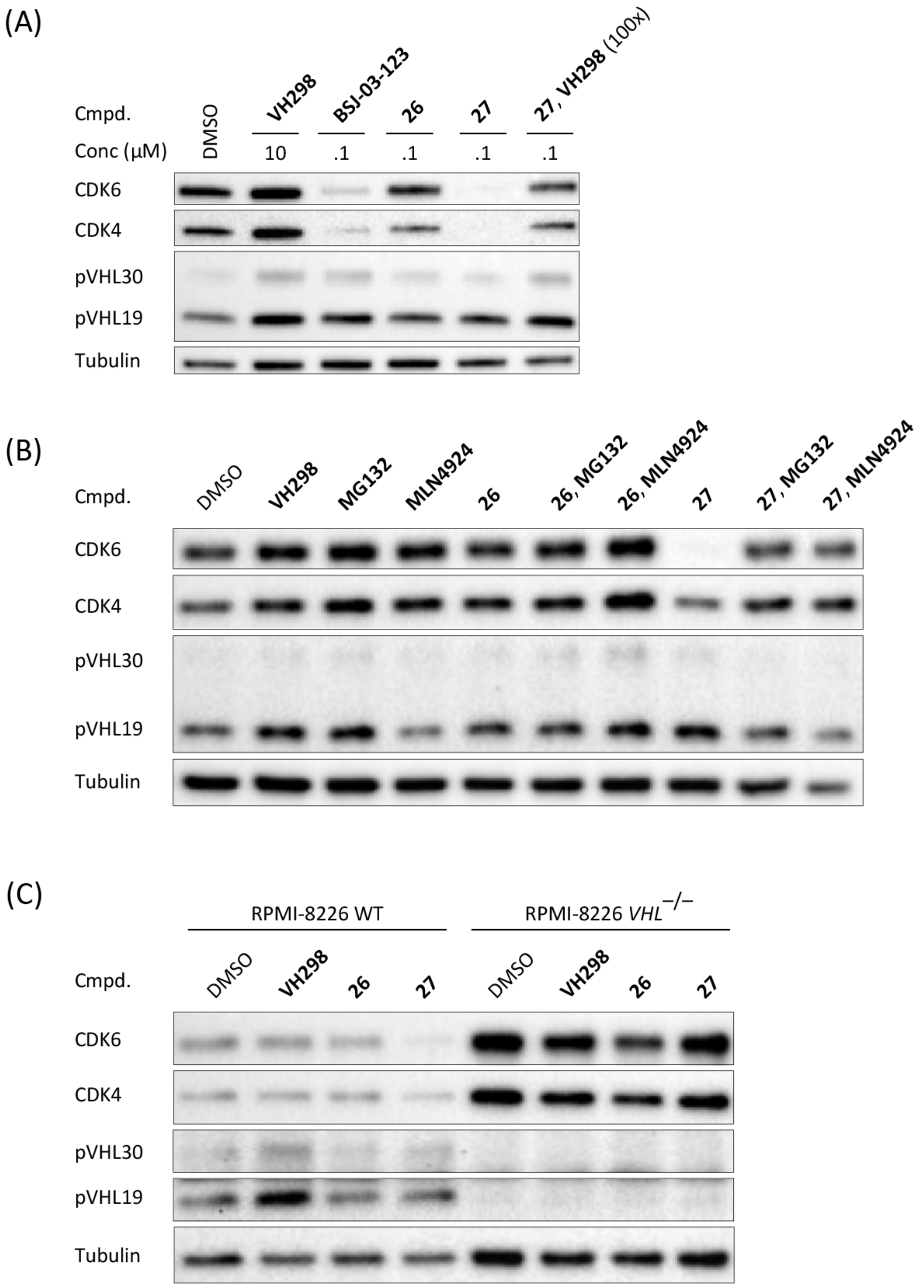

Fig. S6 CDK4/6 degradation induced by PROTAC **27** is mediated *via* the E3 ubiquitin ligase CRL2^{VHL} and subsequent proteasomal degradation. (A) MM.1S cells were treated for 16 h with vehicle, VH298 (**4**), the CDK6-selective degrader BSJ-03-123 (**8**), negative control **26**, or PROTAC **27** at the indicated concentrations. Co-treatment of **27** and VH298 (**4**, 100fold excess) significantly reduced the effects on CDK4/6; (B) MM.1S cells were treated with 10 µM of the proteasome inhibitor MG132, 10 µM of the NEDD8-activating enzyme inhibitor MLN4924 or 0.1 µM PROTACs alone for 3 h or cells were pre-treated with inhibitors MG132 / MLN4924 for 1 h before addition of PROTACs for additional 3 h; (C) RPMI-8226 wild-type or RPMI-8226 *VHL*$^{-/-}$ cells were treated with compounds at 0.1 µM for 16 h.

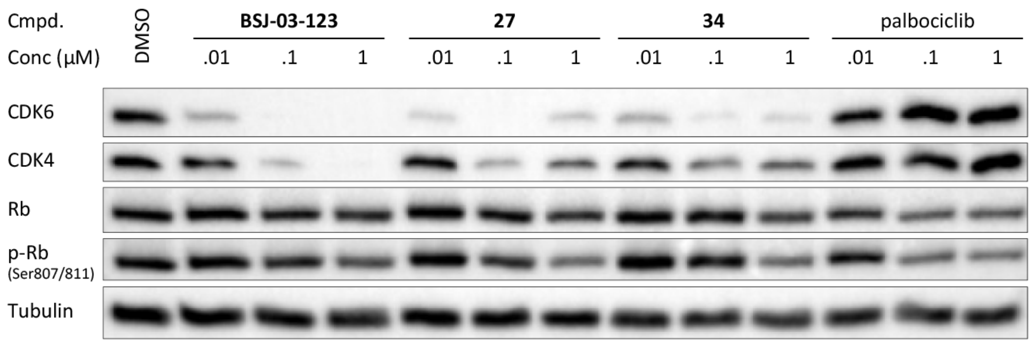

Fig. S7 CDK4/6 inhibition with palbociclib exhibits a stronger inhibitory effect of the kinase function of CDK4/6 compared to CDK4/6 degradation by CRBN- and VHL-based PROTACs. MM.1S cells were treated with BSJ-03-123 (**8**), **27**, **34**, and palbociclib for 16 h at the indicated concentrations. Target proteins CDK4 and CDK6, along with retinoblastoma (Rb) and phosphorylated retinoblastoma (p-Rb) (Ser807/811) were blotted.

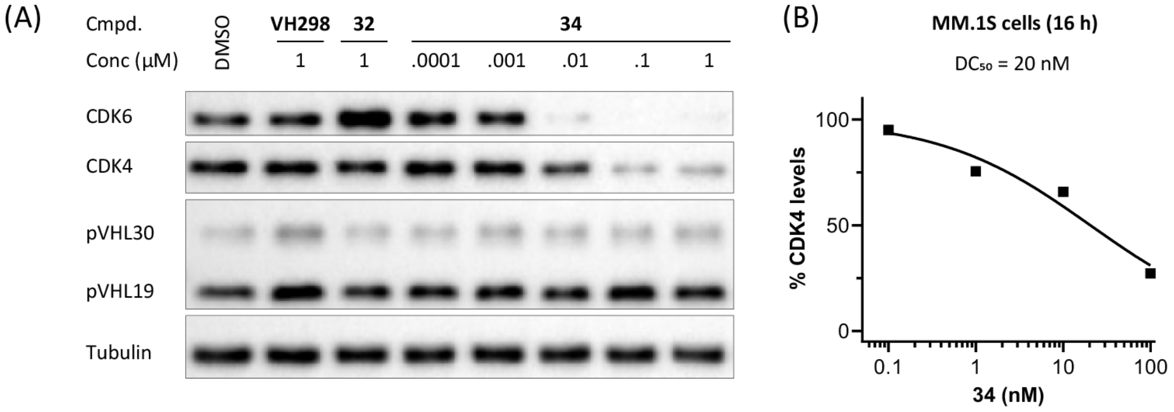

Fig. S8 VHL-based PROTAC **34** of the 'phenoxy' subseries induces CDK6-selective degradation in a dose-dependent manner. (A) MM.1S cells were treated with 1 µM of the VHL ligand VH298 (**4**), BSJ-03-123 (**8**), negative control **32**, or PROTAC **34** at the indicated concentrations for 16 h; (B) Quantification of (A) and calculation of the DC_{50}-value for CDK4. Parts of the blot (A) and the DC_{50} curve for CDK6 degradation by PROTAC **34** are presented in the main body of the manuscript.

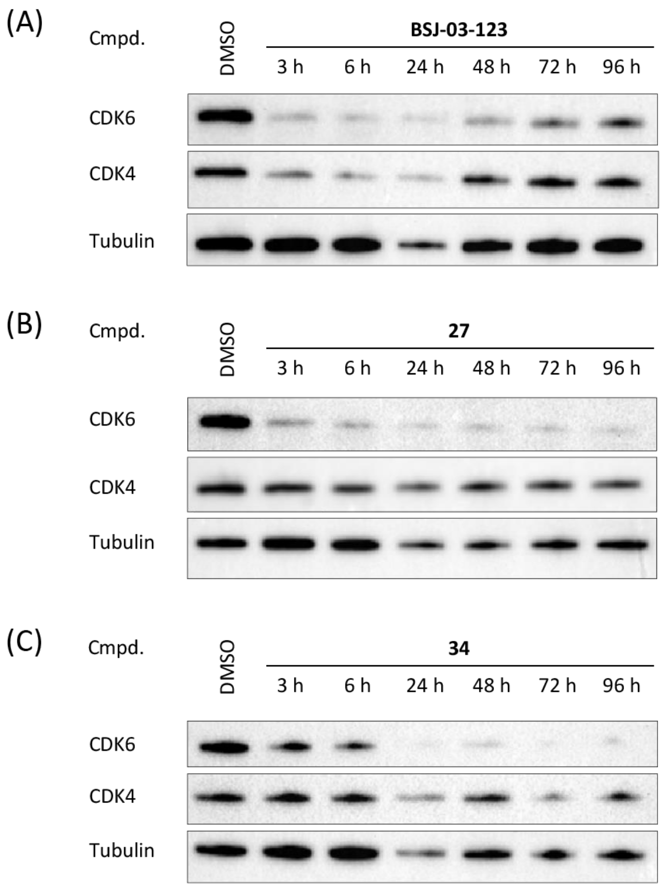

Fig. S9 Long-term treatments with (A) BSJ-03-123 (**8**) versus VHL-based PROTAC **27** of the 'amide' subseries (B) and PROTAC **34** of the 'phenoxy' subseries (C). MM.1S cells were treated with PROTACs at 0.1 µM for the indicated time without washout.

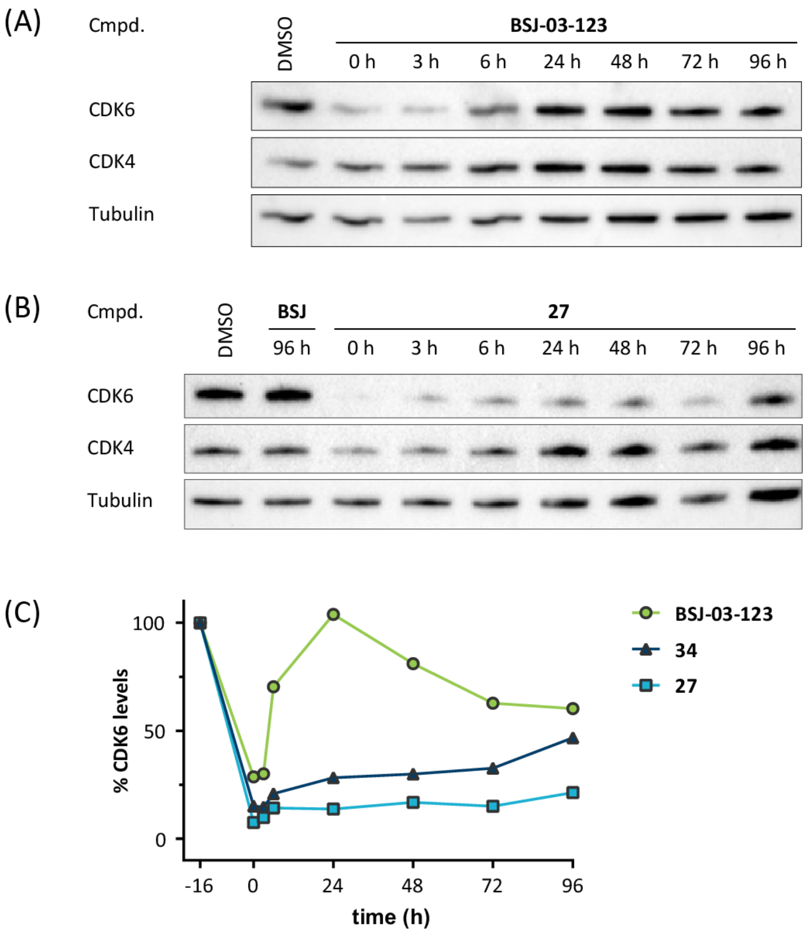

Fig. S10 Washout experiments with (A) BSJ-03-123 (**8**) versus VHL-based PROTAC **27** of the 'amide' subseries (B) and PROTAC **34** of the 'phenoxy' subseries (Fig. 3E). (C) Quantification of (A), (B), and Fig. 4E. MM.1S cells were treated with PROTACs at 0.1 µM for 16 h before washout with PBS (= 0 h), then kept in plain media until indicated time points.

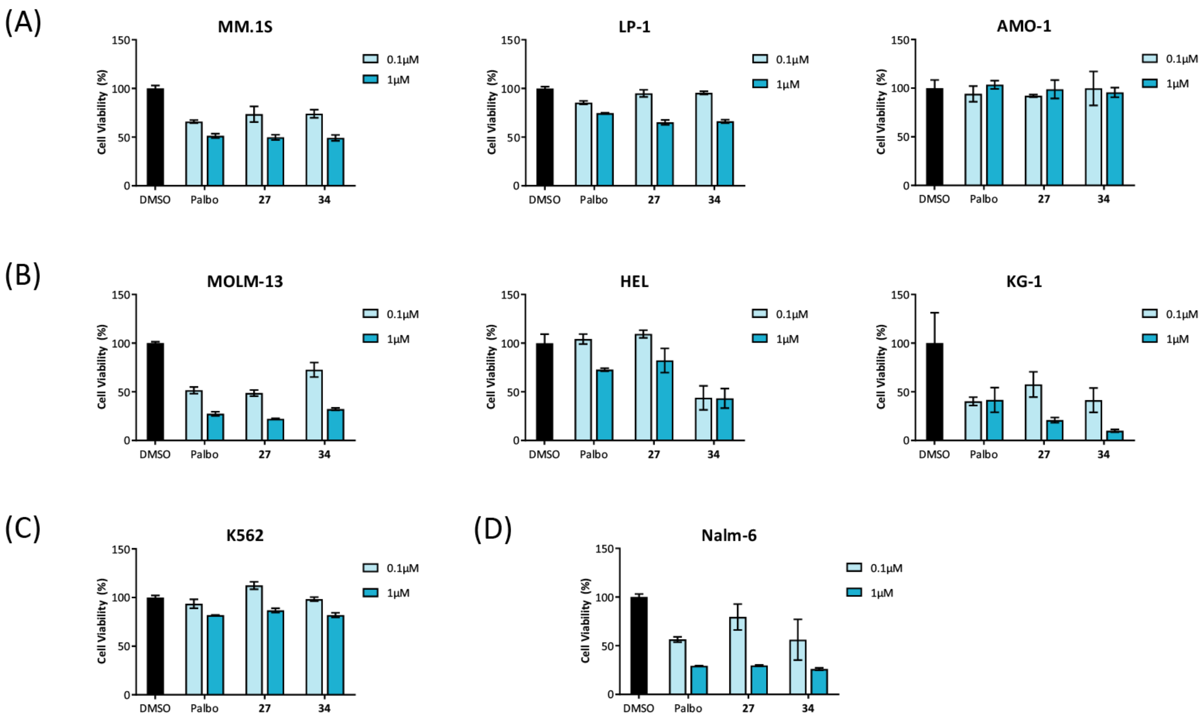

Fig. S11 VHL-based PROTACs are as potent as palbociclib (Palbo) on decreasing cell viability in various cell lines. (A) Human multiple myeloma cell lines MM.1S, LP-1, and AMO-1; (B) acute myeloid leukemia cell lines MOLM-13, HEL, KG-1; (C) chronic myelogenous leukemia cell line K562; and (D) acute lymphoblastic leukemia cell line Nalm-6 were treated with Palbo, **27**, or **34** for 96 h at 0.1 µM or 1 µM. All results were normalized to non-treated conditions and data represent mean ±SD of biological triplicates.

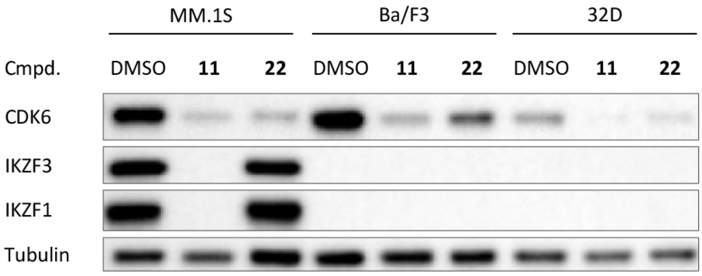

Fig. S12 CRBN- and VHL-based PROTACs are capable of inducing CDK6 degradation in human and murine cell lines. MM.1S cells, Ba/F3 or 32D cells were treated with the CRBN-based PROTAC **11** and VHL-addressing CDK4/6d **22** for 16 h at 1 µM.

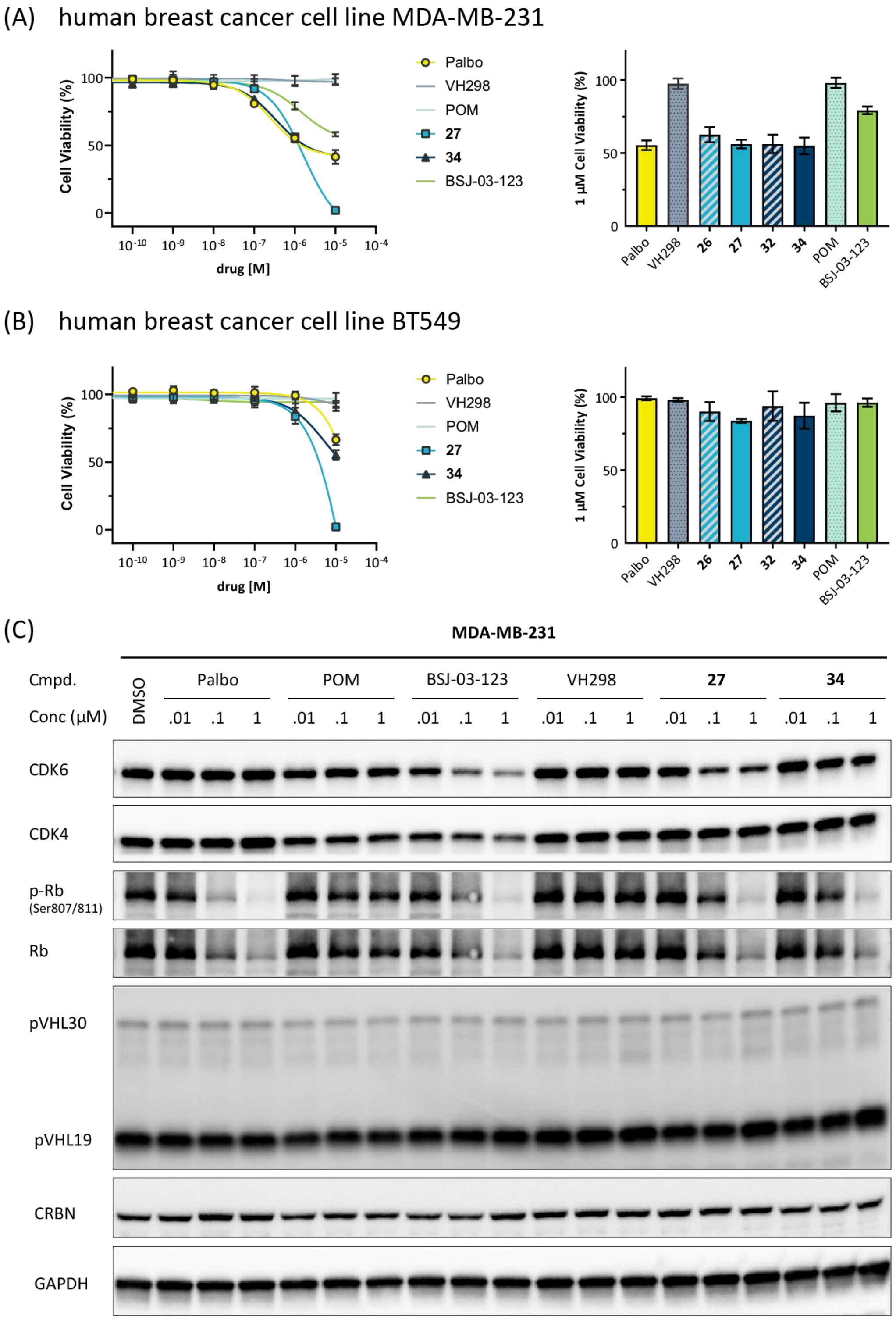

Fig. S13 Sensitivity of human cancer cell lines to CDK4/6 PROTACs. (A) Palbociclib-sensitive MDA-MB-231 cells were treated with control compounds in a dose-dependent manner for 96 h. Viability data were normalized to non-treated conditions and data represent mean ±SD of biological triplicates; (B) BT549 were treated with palbociclib, VHL-based CDK4/6 PROTACs, and CRBN-based CDK4/6 PROTACs or control compounds in a dose-dependent manner for 96 h; (C) PROTAC-mediated CDK6-selective degradation in breast cancer. MDA-MB-231 cells were treated with the compounds at the concentrations indicated for 16 h. Whole cell extracts were collected and subjected to immunoblotting using primary antibodies against the proteins as indicated.

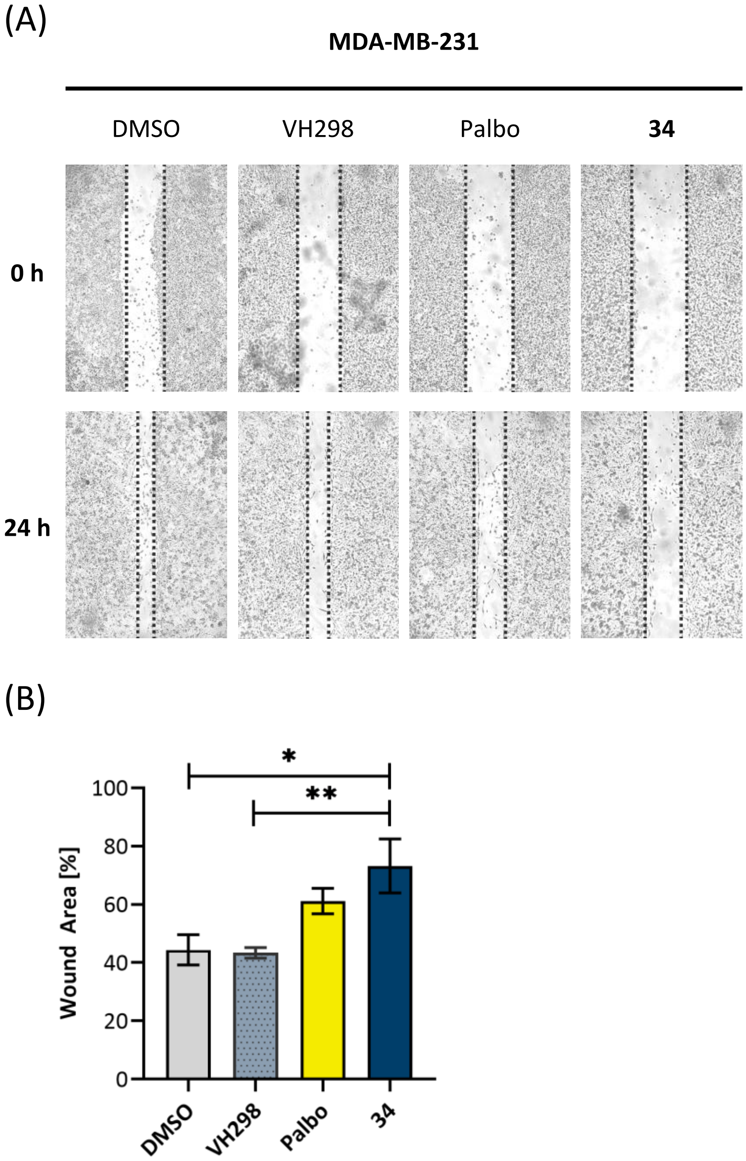

Fig. S14 Cell migration assay in MDA-MB-231 cells. (A) Wound gap pictures; (B) Column diagram showing the open wound area. Cells were pre-treated with 0.1 µM of the corresponding compound, followed by the scratch and an additional treatment with 0.1 µM. Pictures were acquired 24 h post-scratch.

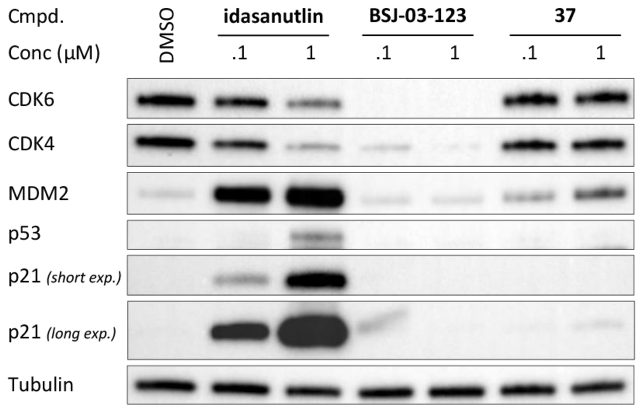

Fig. S15 Putative MDM2-based degrader **37** does not induce CDK4/6 degradation. MM.1S cells were treated with idasanutlin (**6**), the CDK6-selective degrader BSJ-03-123 (**8**), or degrader **37** at the indicated concentrations for 16 h. In addition to the target proteins CDK4/6, the expression levels of the E3 ligase MDM2 and the tumour suppressor molecule p53 as well as its downstream effector p21 were blotted.

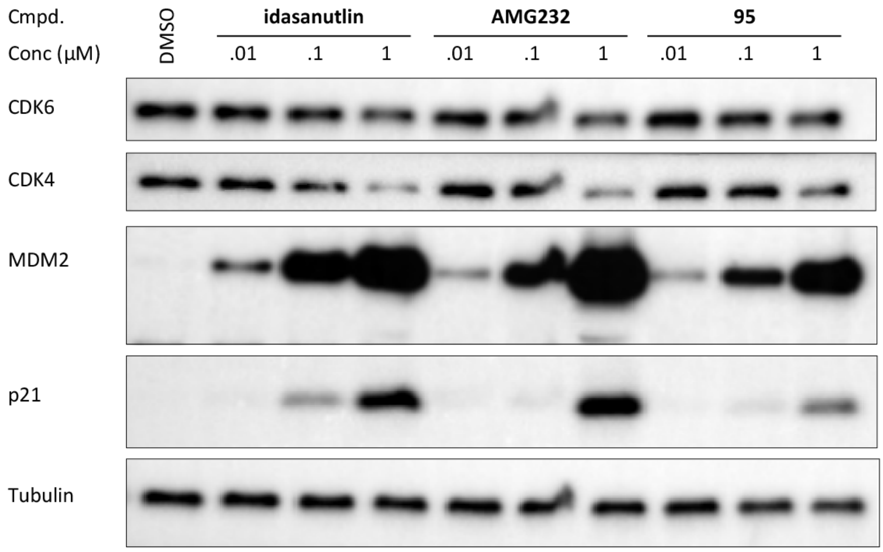

Fig. S16 MDM2i and MDM2-based PROTACs induce lead to diminished CDK4 levels in a dose-and MDM2/p21-dependent manner. MM.1S cells were treated with idasanutlin (**6**), AMG232, or bifunctional probe **95** at the indicated concentrations for 16 h.

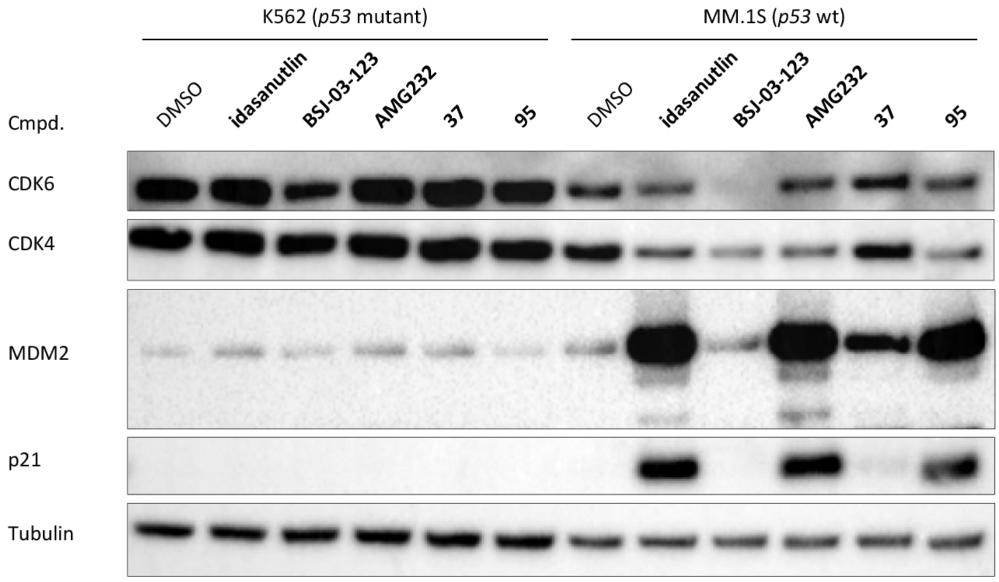

Fig. S17 Off-target CDK4/6 protein decrease and p21 stabilization resulting from treatment with MDM2i or functionalized MDM2-ligands is dependent on the p53 status. The human chronic myelogenous leukemia (CML) cell line K562 and the human multiple myeloma cell line MM.1S were treated with 1 µM of MDM2i idasanutlin (**6**), CDK6-selective degrader BSJ-03-123 (**8**), MDM2i AMG232, putative MDM2-based CDK4/6 degrader **37**, and MDM2-based bifunctional probe **95** for 16 h.

Supplementary Information: Biology

A. Cell Lines

Cell lines MM.1S, LP-1, AMO-1, MOLM13, HEL, KG-1, K562, Nalm6, RPMI-8226, 32D, Ba/F3, and HEK293T were obtained from the American Type Culture Collection (ATCC) or the Deutsche Sammlung von Mikroorganismen and Zellkulturen (DSMZ). HEK293T cells were maintained in DMEM medium (Gibco) while all other cells were maintained in RPMI-1640 medium (Biochrom). All media contained 10% fetal bovine serum (FBS) (Merck Millipore) and was supplemented with 1% penicillin/streptomycin (Thermo Fischer) and 1% L-glutamine (Merck Millipore). Cell lines 32D and Ba/F3 were cultured in the presence of murine IL-3 (Preprotech). Cells were maintained at 37 °C with 5% CO_2 in humidified atmosphere.

Human breast cancer cell lines MDA-MB-231 and BT549 were cultured in RPMI-1640 medium (Lonza) supplemented with 10% fetal bovine serum (Gibco) and were cultured at 37 °C in a humidified 5 % CO_2 incubator.

B. CRISPR-mediated Knockout of CRBN and VHL

For the generation of knock-out cell lines utilizing CRISPR/Cas9, plasmids pLKO5d.SSF.SpCas9.P2a.BSD and pLKO5.hU6.sgRNA.dTom (gifts from D. Heckl, Hannover Medical School) were used.[6] Single-guide RNAs targeting CRBN and VHL were cloned into the lentiviral vector pLKO5.hU6.sgRNA.dTom. Guide RNA sequences are as follows: CRBN: 5'-GTCCTGCTGATCTCCTTCGC-3'; VHL: 5'-GAGATGCAGGGACACACGAT-3'.

HEK293T cells were transfected with lentiviral vectors along with plasmids encoding for envelope proteins and packaging proteins. Viral supernatants were harvested 48 hours post-transfection and were used to transduce multiple myeloma cell line RPMI-8226. RPMI-8226 cells were first transduced with virus containing pLKO5d.SSF.SpCas9.P2a.BSD and were selected with blasticidin (Invivogen), followed by transduction with sgRNA-expressing lentiviral vectors. Transduction success was confirmed through FACS analysis 48 hours post-transduction with a minimum efficiency of 95% tomato fluorescence. CRBN or VHL knockout was confirmed through Western blot analysis.

C. Immunoblotting

Multiple myeloma, leukaemia, and murine cell lines:

After respective designated treatments, cells were washed with 1× PBS and lysed in IP lysis buffer (Pierce) containing HALT protease and phosphatase inhibitor cocktail (Thermo Scientific). Sodium dodecyl sulfate-polyacrylamide gel electrophoresis (SDS-PAGE) was performed with 5–20 μg of protein loaded per sample. Gel electrophoresis was carried out at a constant voltage. Proteins were transferred onto Immobilon-P transfer membranes (Millipore). Membranes were blocked with 5% milk in Tris Buffered Saline with 0.05% Tween 20 (TBS-T) followed by overnight incubation at 4 °C with shaking in primary antibodies diluted in 5% BSA in TBS-T. After incubation with respective secondary HRP-conjugated antibodies diluted in 5% milk in TBST, detection of proteins was performed using either WesternBright ECL HRP substrate or WesternBright Sirius HRP substrate (Advansta). Chemiluminescence was detected with ChemiDoc™ XRS+ System (Bio-Rad). For further detection of other proteins on the same membrane, membranes were subjected to incubation in Restore Western Blot Stripping Buffer, followed by re-blocking, and further probing of proteins was carried out. Quantification was performed using ImageJ (National Institutes of Health). Antibodies used are as follows: CDK6 (Cell Signaling, clone DCS83, 3136), CDK4 (Cell signaling, clone D9G3E, 12790), Rb (Cell Signaling, clone 4H1, 9309), phospho-Rb (Ser807/811) (Cell Signaling, 9308), CRBN (Sigma, HPA045910), IKZF1 (Cell Signaling, clone D6N9Y, 14859), IKZF3 (Cell Signaling, clone D1C1E, 15103), VHL (Cell Signaling, 68547), p53 (Cell Signaling, clone 7F5, 2527), p21 Waf1/Cip1 (Cell Signaling, clone 12D1, 2947), MDM2 (Santa Cruz, clone SMP14, sc-965), BIRC2 (BioRad, clone AB01/3B4, VMA00532), c-IAP2 (Cell Signaling, clone 58C7, 3130), XIAP (Cell Signaling, clone D2Z8W, 14334), and α-Tubulin (Sigma, clone B512, T5168).

Breast cancer cell lines:

To examine the effect of PROTAC-mediated target degradation, cells were treated with compounds at indicated concentrations for 16 hr. Whole cell extract was harvested in Laemmli sample buffer and subjected to SDS-PAGE followed by immunoblotting using primary antibodies raised against CDK4 (Cell Signaling Technology, #12790), CDK6 (Cell Signaling Technology, #3136), phospho-RB_Ser807/811 (Cell Signaling Technology, #8516), RB (Cell Signaling Technology, #9309), VHL (Cell Signaling Technology, #68547), CRBN (Sigma, #HPA045910), and GAPDH (Santa Cruz, #SC-47724). HRP-conjugated secondary antibodies used were from Jackson Immuno Research. Immunoblots were imaged using a ChemiDoc Touch Imaging System (Bio-Rad).

D. Cell Viability Assay

Multiple myeloma, leukaemia, and murine cell lines:

Cells were seeded in 96-well plates with media containing indicated concentrations of respective compounds and plates were incubated at 37 °C for 96 hours. Readout was performed with CellTiter-Glo® Luminescent Cell Viability Assay (Promega) according to the manufacturer's protocol and luminescence was measured with POLARStar Omega plate reader (BMG LabTech). All results were normalized to non-treated conditions and data represent mean ±SD of biological triplicates.

Breast cancer cell lines:

To access the compound effect on cell viability, cells were plated in 384-well tissue culture-treated plates (Greiner Bio One). Twenty-four hours later, tested compounds were added into culture to the desired concentrations. Cell viability was measured by using CellTiter-Glo® Luminescent Cell Viability Assay (Promega) at day 4 or day 7 post-treatment and normalized by non-treated controls.

E. Cell Migration Assay

In total 800.000 MDA-MB-231 breast cancer cells were seeded on uncoated 24-well plates (Starlab). After 24 h DMEM medium (Pan Biotech) was removed, wells were washed twice with PBS and incubated with 0.1 µM palbociclib, 0.1 µM **34**, 0.1 µM VH298 or DMSO in fresh FCS-free medium. After 24 h of incubation, a scratch was conducted with a 100 µL pipette tip (Starlab), medium was removed, wells were washed twice with PBS and again incubated with fresh FCS-free medium containing the compounds at the aforementioned concentrations. Wound healing was observed microscopically for 24 h with 10-fold magnification (Axiovert 100, Zeiss, Germany). Migration speed was quantified according to the reduced scratch wound area over time.

F. Statistical Analysis

Dose response curves were modeled and plotted by using GraphPad Prism software (GraphPad Software). Statistical analysis was performed using GraphPad Prism software (GraphPad Software). Variance of biological replicates is represented as the standard error of the mean (SEM) or as the standard deviation (SD).

G. General Remarks

Preparative column chromatography was performed using Merck silica gel 60 (63 – 200 mesh) or using an automated flash chromatography system CombiFlash Rf 200. Indicated compounds were purified using a reversed-phase HPLC system from Knauer (C18 column, 250 × 20 mm, Eurospher 100-10). Petroleum ether used was a mixture of alkanes boiling between 40 – 60 °C. Melting points were determined on a Büchi 510 oil bath apparatus or on a Reichelt hot-stage apparatus and were uncorrected. ^{1}H NMR and ^{13}C NMR spectra were recorded on a Bruker Avance 400 MHz NMR spectrometer, Bruker Avance 500 MHz NMR spectrometer or on a Bruker Avance III 600 MHz NMR spectrometer, respectively. NMR spectra were processed and analyzed in MestReNova. Chemical shifts are given in parts per million (ppm), coupling constants *J* are given in Hertz, and spin multiplicities are given as s (singlet), d (doublet), t (triplet), q (quartet) or m (multiplet). All multiplets related with *J*(C,F) couplings in ^{13}C NMR spectra are centred. In case of overlapping extraneous solvent peaks, multiplet analyses in ^{1}H NMR spectra were performed using qGSD (quantitative Global Spectral Deconvolution). In case of rotamers only the peaks for the major rotamer are given, Resonance assignments were made on the basis of one- and two-dimensional NMR techniques which include ^{1}H, ^{13}C, DEPT, HSQC, and HMBC experiments. HRMS was recorded on a micrOTOF-Q mass spectrometer (Bruker) with ESI-source coupled with an HPLC Dionex UltiMate 3000 (Thermo Scientific). The purity and identity of the compounds were determined by HPLC-UV obtained on an LC-MS instrument (Applied Biosystems API 2000 LC/MS/MS, HPLC Agilent 1100) or separately on an LC instrument (Acquity UPLC) and mass spectrometer (Thermo Scientific Q Exactive Plus). The purity of all the final compounds was confirmed to be ≥95% purity by LC.

H. Synthesis of CRBN-based PROTACs

General Procedure I: HATU-mediated coupling with palbociclib. The corresponding protected CRBN-linker conjugate (0.2 mmol) was dissolved in dry CH_2Cl_2 (5 mL) and TFA (5 mL) and stirred at 40 °C for 2 h. After removal of the volatiles, the oily residue was further dried under high vacuum. Subsequently, the crude acid was dissolved in dry DMF (12 mL) and DIPEA (0.14 mL, 0.8 mmol) was added, followed by the addition of HATU (0.22 mmol). After stirring for 5 minutes, palbociclib (90 mg, 0.2 mmol) was added to the mixture. It was stirred at room temperature for 16 h, after which half-saturated brine (50 mL) was added, and the product was extracted with EtOAc (3 × 25 mL). The combined organic phases were washed with brine (50 mL), dried over Na_2SO_4, filtered and concentrated *in vacuo*.

Palbociclib (1)

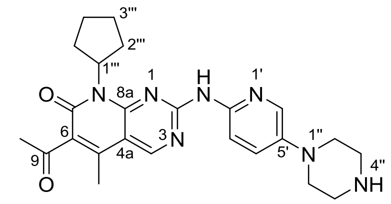

This compound was used as commercially supplied (Activate Scientific).

^{1}H NMR (600 MHz, 308 K, DMSO-d_6) δ 1.51 – 1.63 (m, 2H), 1.70 – 1.80 (m, 2H), 1.82 – 1.95 (m, 2H), 2.19 – 2.27 (m, 2H, 2'''-H, 3'''-H), 2.30 (s, 3H, CH_3), 2.41 (s, 3H, 10-H), 2.81 – 2.88 (m, 4H), 3.02 – 3.08 (m, 4H, 2''-H, 3''-H), 3.14 – 3.27 (m, 1H, 4''-H), 5.81 (p, *J* = 8.9 Hz, 1H, 1'''-H), 7.42 (dd, *J* = 3.1, 9.1 Hz, 1H, 4'-H), 7.82 (d, *J* = 9.0 Hz, 1H, 3'-H), 8.02 (d, *J* = 3.0 Hz, 1H, 6'-H), 8.93 (s, 1H, 4-H), 10.00 (s, 1H, NH); **^{13}C NMR** (151 MHz, 308 K, DMSO-d_6) δ 13.12 (CH_3), 25.17 (C-3'''), 27.65 (C-2'''), 31.39 (C-10), 45.52, 49.55 (C-2'', C-3''), 53.04 (C-1'''), 106.67 (C-4a), 115.34 (C-3'), 124.68 (C-4'), 129.32 (C-6), 135.45 (C-6'), 142.17 (C-5'), 144.22, 144.25 (C-8a, C-2'), 154.89 (C-5), 158.34 (C-4), 158.72 (C-2), 160.89 (C-7), 202.49 (C-9).

YKL-06-102 (7)

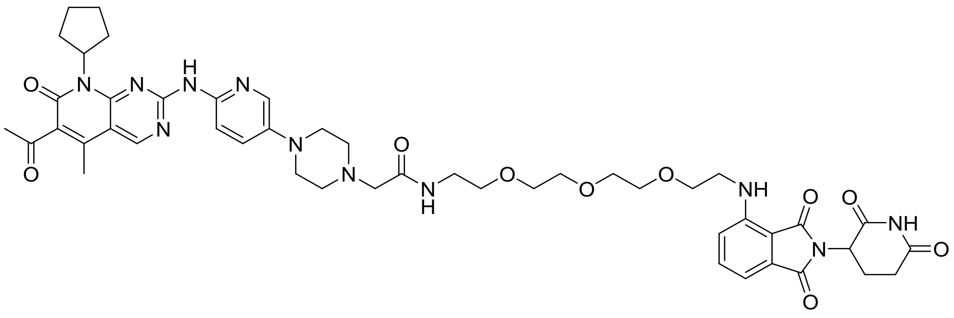

This compound was synthesized as reported previously.[2] The crude material was purified by column chromatography (CH_2Cl_2/EtOH 19:1) to yield the title compound (free amine) as a yellow solid.

Yield (70%); mp 128 – 134 °C; R_f = 0.29 (CH_2Cl_2/MeOH 19:1); **^{1}H NMR** (500 MHz, DMSO-d_6) δ 1.57 (t, *J* = 5.8 Hz, 2H), 1.72 – 1.78 (m, 2H), 1.82 – 1.92 (m, 2H), 1.95 – 2.05 (m, 1H), 2.21 – 2.24 (m, 2H), 2.29 (s, 3H), 2.41 (s, 3H), 2.59 (t, *J* = 5.0 Hz, 4H), 2.81 – 2.92 (m, 1H), 2.97 (s, 2H), 3.18 (t, *J* = 5.1 Hz, 4H), 3.22 – 3.46 (m, 8H), 3.47 – 3.54 (m, 8H), 3.57 (t, *J* = 5.4 Hz, 2H), 5.03 (dd, *J* = 5.4, 12.8 Hz, 1H), 5.79 (q, *J* = 8.7 Hz, 1H), 6.55 (t, *J* = 5.8 Hz, 1H), 7.00 (d, *J* = 7.1 Hz, 1H), 7.08 (d, *J* = 8.5 Hz, 1H), 7.44 (dd, *J* = 3.1, 8.9 Hz, 1H), 7.54 (t, *J* = 7.7 Hz, 1H), 7.70 (t, *J* = 5.8 Hz, 1H), 7.83 (d, *J* = 9.1 Hz, 1H), 8.04 (d, *J* = 3.0 Hz, 1H), 8.92 (d, *J* = 1.1 Hz, 1H), 10.00 (s, 1H), 11.05 (br s, 1H); **^{13}C NMR** (126 MHz, DMSO-d_6) δ 13.76, 22.31, 25.25, 27.71, 31.14, 31.45, 38.28, 41.87, 48.44, 48.74, 52.70, 53.10, 55.04, 61.27, 69.05, 69.14, 69.73, 69.95, 70.05, 106.76, 109.43, 110.82, 115.31, 117.55, 124.86, 129.41, 132.23, 135.52, 136.35, 142.23, 143.55, 144.49, 146.56, 154.93, 158.38, 158.72, 160.94, 167.44, 169.10, 169.27, 170.19, 172.94, 202.60; **LC-MS** (ESI) (90% H_2O to 100% MeOH in 10 min, then 100% MeOH to 20 min, DAD 220-450 nm), t_R = 11.30 min, 99% purity, *m/z* $[M + H]^+$ calcd for $C_{47}H_{57}N_{11}O_{10}$, 936.43; found, 936.7; **HRMS** (ESI) *m/z* $[M + H]^+$ calcd for $C_{47}H_{57}N_{11}O_{10}$, 936.4363; found, 936.4364.

***tert*-Butyl 6-(benzyloxycarbonylamino)hexanoate (38)**

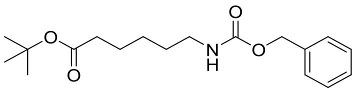

This compound was synthesized as we described previously.[7]

4-Fluoro-thalidomide (39)

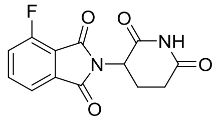

This compound was synthesized as we described previously.[8]

CRBN-6 building block (40)

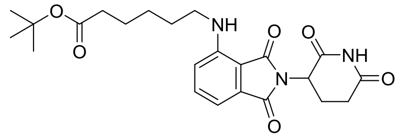

This compound was synthesized as we described previously.[7]

CRBN-based PROTAC 11

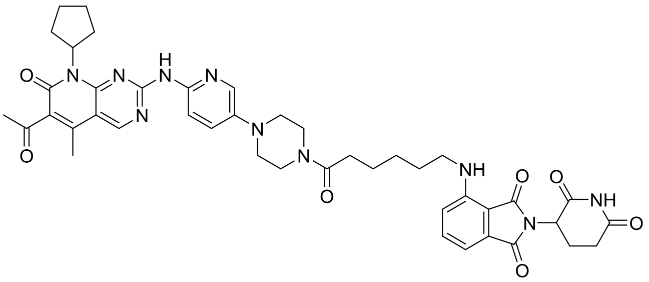

This compound was prepared using the General Procedure **I** and CRBN building block **40** (89 mg). The crude product was purified by column chromatography (gradient of CH_2Cl_2/EtOH 29:1 to 19:1) to obtain a yellow solid.

Yield (66 mg, 40%); mp 172 – 176 °C; R_f = 0.64 (EtOAc/EtOH 9:1); **^{1}H NMR** (500 MHz, DMSO-d_6) δ 1.32 – 1.44 (m, 2H), 1.50 – 1.65 (m, 6H), 1.69 – 1.81 (m, 2H), 1.83 – 1.94 (m, 2H), 1.97 – 2.07 (m, 1H), 2.18 – 2.27 (m, 2H), 2.30 (s, 3H), 2.36 (t, *J* = 7.4 Hz, 2H), 2.41 (s, 3H), 2.44 – 2.62 (m, 2H), 2.80 – 2.92 (m, 1H), 3.06 – 3.18 (m, 4H), 3.26 – 3.33 (m, 2H), 3.52 – 3.68 (m, 4H), 5.03 (dd, *J* = 5.5, 12.7 Hz, 1H), 5.81 (p, *J* = 9.0 Hz, 1H), 6.51 (t, *J* = 6.0 Hz, 1H), 7.00 (d, *J* = 7.0 Hz, 1H), 7.08 (d, *J* = 8.6 Hz, 1H), 7.47 (dd, *J* = 3.1, 9.1 Hz, 1H), 7.56 (dd, *J* = 7.0, 8.7 Hz, 1H), 7.86 (d, *J* = 9.0 Hz, 1H), 8.06 (d, *J* = 3.0 Hz, 1H), 8.94 (s, 1H), 10.06 (s, 1H), 11.05 (br s, 1H); **^{13}C NMR** (126 MHz, DMSO-d_6) δ 13.75, 22.32, 24.66, 25.25, 26.23, 27.71, 28.70, 31.13, 31.44, 32.26, 40.85, 41.94, 44.80, 48.67, 48.72, 49.10, 53.11, 106.80, 109.19, 110.53, 115.22, 117.34, 125.47, 129.46, 132.35, 136.08, 136.43, 142.20, 143.36, 144.92,

146.61, 154.92, 158.37, 158.68, 160.91, 167.44, 169.11, 170.20, 170.79, 172.92, 202.56; **LC-MS** (ESI) (90% H_2O to 100% MeCN in 10 min, then 100% MeCN to 20 min, DAD 220-400 nm), t_R = 11.64 min, 99% purity, *m/z* $[M + H]^+$ calcd for $C_{43}H_{48}N_{10}O_7$, 817.37; found, 816.9; **HRMS** (ESI) *m/z* $[M + H]^+$ calcd for $C_{43}H_{48}N_{10}O_7$, 817.3780; found, 817.3769.

CRBN-2-2-2 building block (41)

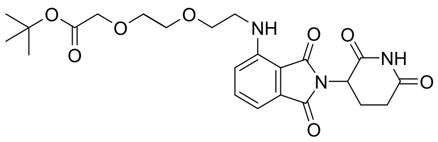

This compound was synthesized as we described previously.[8]

CRBN-based PROTAC 12

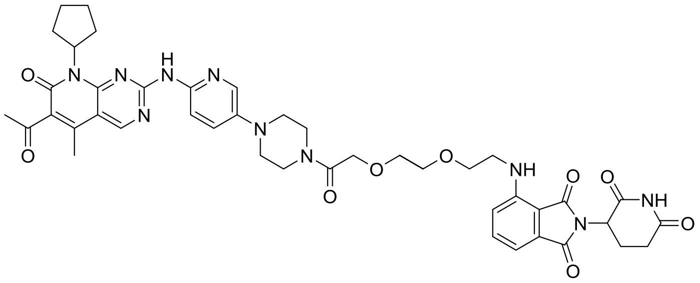

This compound was prepared using the General Procedure **I** and CRBN building block **41** (95 mg). The crude product was purified by column chromatography (gradient of EtOAc/EtOH 15:1 to 12:1) to obtain a yellow solid.

Yield (57 mg, 33%); mp 92 – 96 °C; R_f = 0.31 (CH_2Cl_2/EtOH 19:1); **^{1}H NMR** (600 MHz, DMSO-d_6) δ 1.50 – 1.62 (m, 2H), 1.70 – 1.81 (m, 2H), 1.84 – 1.92 (m, 2H), 1.96 – 2.04 (m, 1H), 2.18 – 2.28 (m, 2H), 2.30 (s, 3H), 2.41 (s, 3H), 2.46 – 2.63 (m, 2H), 2.81 – 2.91 (m, 1H), 3.03 – 3.23 (m, 4H), 3.46 (q, *J* = 5.6 Hz, 2H), 3.49 – 3.81 (m, 10H), 4.19 (s, 2H), 5.03 (dd, *J* = 5.5, 12.8 Hz, 1H), 5.81 (p, *J* = 8.9 Hz, 1H), 6.59 (t, *J* = 5.8 Hz, 1H), 7.00 (d, *J* = 7.0 Hz, 1H), 7.12 (d, *J* = 8.6 Hz, 1H), 7.44 (dd, *J* = 3.1, 9.1 Hz, 1H), 7.55 (dd, *J* = 7.1, 8.6 Hz, 1H), 7.85 (d, *J* = 9.0 Hz, 1H), 8.04 (d, *J* = 3.0 Hz, 1H), 8.94 (s, 1H), 10.06 (s, 1H), 11.07 (s, 1H); **^{13}C NMR** (151 MHz, DMSO-d_6) δ 13.76, 22.29, 25.25, 27.70, 31.13, 31.44, 40.96, 41.88, 44.18, 48.55, 48.72, 48.92, 53.09, 62.94, 68.97, 69.70, 69.73, 69.98, 69.98, 106.78, 109.41, 110.82, 115.11, 117.55, 125.40, 129.45, 132.23, 136.03, 136.37, 142.20, 143.26, 144.91, 146.54, 154.91, 158.38, 158.64, 160.90, 167.41, 167.45, 169.10, 170.20, 172.91, 202.57; **LC-MS** (ESI) (90% H_2O to 100% MeCN in 10 min, then 100% MeCN to 20 min, DAD 220-500 nm), t_R = 8.39 min, 98% purity, *m/z* $[M + H]^+$

calcd for $C_{43}H_{48}N_{10}O_9$, 849.36; found, 849.5; **HRMS** (ESI) *m/z* $[M + H]^+$ calcd for $C_{43}H_{48}N_{10}O_9$, 849.3678; found, 849.3619.

CRBN-2-2-2-2 building block (42)

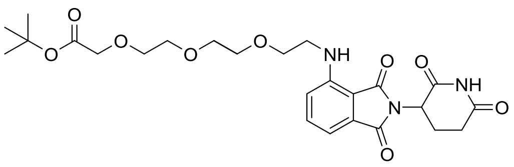

This compound was synthesized as we described previously.[8]

CRBN-based PROTAC 13

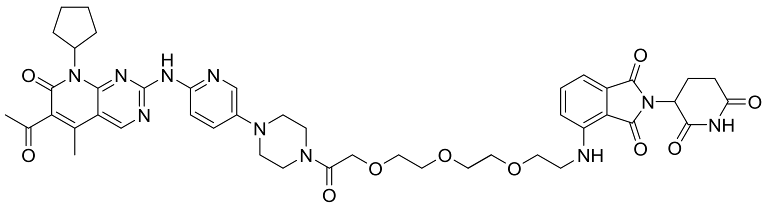

This compound was prepared using the General Procedure **I** and CRBN building block **42** (104 mg). The crude product was purified by column chromatography (gradient of CH_2Cl_2/EtOH 29:1 to 15:1) to obtain a yellow solid.

Yield (82 mg, 46%); mp 140 – 144 °C; R_f = 0.26 (CH_2Cl_2/EtOH 19:1); ^{1}**H NMR** (500 MHz, DMSO-d_6) δ 1.46 – 1.64 (m, 2H), 1.69 – 1.78 (m, 2H), 1.81 – 1.95 (m, 2H), 1.97 – 2.06 (m, 1H), 2.18 – 2.27 (m, 2H), 2.30 (s, 3H), 2.41 (s, 3H), 2.44 – 2.62 (m, 2H), 2.73 – 2.92 (m, 1H), 3.08 – 3.20 (m, 4H), 3.44 (q, *J* = 5.5 Hz, 2H), 3.50 – 3.67 (m, 14H), 4.17 (s, 2H), 5.03 (dd, *J* = 5.5, 12.7 Hz, 1H), 5.81 (p, *J* = 8.9 Hz, 1H), 6.57 (t, *J* = 5.8 Hz, 1H), 7.00 (d, *J* = 7.0 Hz, 1H), 7.10 (d, *J* = 8.7 Hz, 1H), 7.46 (dd, *J* = 3.1, 9.1 Hz, 1H), 7.54 (dd, *J* = 7.1, 8.6 Hz, 1H), 7.86 (d, *J* = 9.0 Hz, 1H), 8.05 (d, *J* = 3.0 Hz, 1H), 8.93 (s, 1H), 10.05 (s, 1H), 11.05 (br s, 1H); ^{13}**C NMR** (126 MHz, DMSO-d_6) δ 13.75, 22.29, 25.24, 27.70, 31.13, 31.43, 40.96, 41.86, 44.23, 48.58, 48.73, 48.98, 53.10, 69.05, 69.69, 69.84, 69.91, 69.97, 106.79, 109.42, 110.79, 115.16, 117.54, 125.46, 129.46, 132.22, 136.08, 136.33, 142.19, 143.31, 144.93, 146.54, 154.91, 158.36, 158.66, 160.90, 167.41, 167.46, 169.08, 170.18, 172.91, 202.55; **LC-MS** (ESI) (90% H_2O to 100% MeCN in 10 min, then 100% MeCN to 20 min, DAD 220-450 nm), t_R = 8.56 min, 99% purity, *m/z* $[M + H]^+$ calcd for $C_{45}H_{52}N_{10}O_{10}$, 893.39; found, 893.4; **HRMS** (ESI) *m/z* $[M + H]^+$ calcd for $C_{45}H_{52}N_{10}O_{10}$, 893.3941; found, 893.3923.

CRBN-2-2-2-5 building block (43)

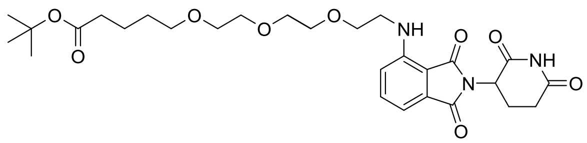

This compound was synthesized as we described previously.[8]

CRBN-based PROTAC 14

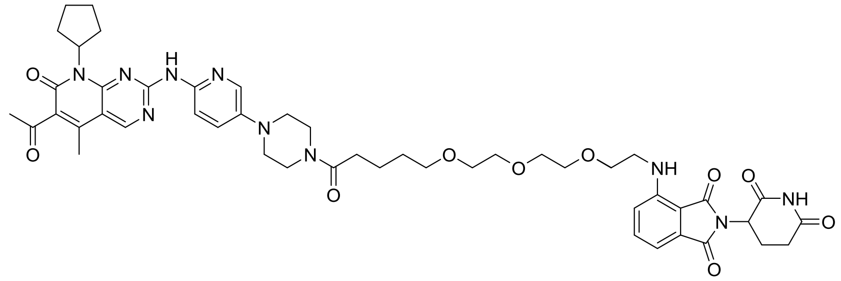

This compound was prepared using the General Procedure **I** and CRBN building block **43** (112 mg). The crude product was purified by column chromatography (gradient of CH_2Cl_2/EtOH 29:1 to 19:1) to obtain a yellow solid.

Yield (126 mg, 67%); mp 112 – 114 °C; R_f = 0.40 (CH_2Cl_2/EtOH 19:1); **^{1}H NMR** (600 MHz, DMSO-d_6) δ 1.43 – 1.65 (m, 6H), 1.70 – 1.81 (m, 2H), 1.82 – 1.94 (m, 2H), 1.97 – 2.07 (m, 1H), 2.17 – 2.27 (m, 2H), 2.29 (s, 3H), 2.34 (t, *J* = 7.1 Hz, 2H), 2.41 (s, 3H), 2.45 – 2.63 (m, 2H), 2.74 – 2.94 (m, 1H), 3.03 – 3.32 (m, 6H), 3.33 – 3.64 (m, 16H), 5.03 (dd, *J* = 5.4, 12.9 Hz, 1H), 5.80 (t, *J* = 8.9 Hz, 1H), 6.57 (t, *J* = 5.8 Hz, 1H), 7.01 (d, *J* = 7.1 Hz, 1H), 7.11 (d, *J* = 8.6 Hz, 1H), 7.47 (dd, *J* = 3.0, 9.1 Hz, 1H), 7.55 (dd, *J* = 7.1, 8.6 Hz, 1H), 7.86 (d, *J* = 9.0 Hz, 1H), 8.06 (d, *J* = 3.0 Hz, 1H), 8.93 (s, 1H), 10.07 (s, 1H), 11.07 (br s, 1H); **^{13}C NMR** (151 MHz, DMSO-d_6) δ 13.80, 21.80, 22.34, 25.30, 27.75, 28.95, 31.17, 31.48, 32.14, 40.23, 40.87, 41.91, 44.82, 48.70, 48.76, 49.13, 53.14, 69.08, 69.65, 69.99, 70.02, 70.06, 70.24, 106.83, 109.43, 110.86, 115.24, 117.62, 125.54, 129.49, 132.27, 136.12, 136.40, 142.26, 143.40, 144.96, 146.61, 154.95, 158.41, 158.71, 160.95, 167.48, 169.12, 170.23, 170.88, 172.98, 202.66; **LC-MS** (ESI) (90% H_2O to 100% MeCN in 10 min, then 100% MeCN to 20 min, DAD 220-450 nm), t_R = 8.68 min, 96% purity, *m/z* $[M + H]^+$ calcd for $C_{48}H_{58}N_{10}O_{10}$, 935.44; found, 935.5; **HRMS** (ESI) *m/z* $[M + H]^+$ calcd for $C_{48}H_{58}N_{10}O_{10}$, 935.4410; found, 935.4373.

CRBN-3-4-3-4 building block (44)

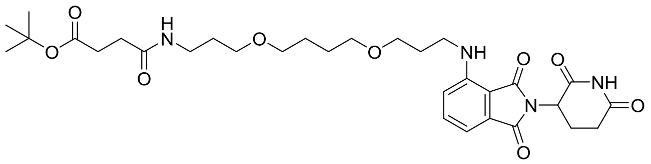

This compound was synthesized as we described previously.[7]

CRBN-based PROTAC 15

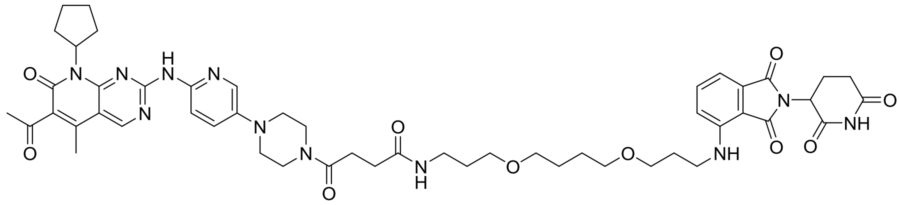

This compound was prepared using the General Procedure **I** and CRBN building block **44** (123 mg). The crude product was purified by column chromatography (gradient of EtOAc to EtOAc/EtOH 9:1) to obtain a yellow solid.

Yield (162 mg, 82%); mp 176 – 180 °C; R_f = 0.17 (CH_2Cl_2/EtOH 19:1); **^{1}H NMR** (600 MHz, DMSO-d_6) δ 1.46 – 1.64 (m, 8H), 1.71 – 1.82 (m, 4H), 1.83 – 1.94 (m, 2H), 1.97 – 2.05 (m, 1H), 2.14 – 2.28 (m, 2H), 2.30 (s, 3H), 2.32 (t, *J* = 7.0 Hz, 2H), 2.41 (s, 3H), 2.46 – 2.62 (m, 4H), 2.80 – 2.92 (m, 1H), 3.02 – 3.22 (m, 6H), 3.31 – 3.37 (m, 8H), 3.43 (t, *J* = 5.9 Hz, 2H), 3.47 – 3.77 (m, 4H), 5.03 (dd, *J* = 5.4, 12.8 Hz, 1H), 5.81 (p, *J* = 8.9 Hz, 1H), 6.63 (t, *J* = 5.9 Hz, 1H), 7.00 (d, *J* = 7.0 Hz, 1H), 7.06 (d, *J* = 8.6 Hz, 1H), 7.48 (dd, *J* = 3.0, 9.1 Hz, 1H), 7.56 (dd, *J* = 7.0, 8.6 Hz, 1H), 7.76 (t, *J* = 5.6 Hz, 1H), 7.87 (d, *J* = 9.0 Hz, 1H), 8.06 (d, *J* = 3.0 Hz, 1H), 8.94 (s, 1H), 10.08 (s, 1H), 11.06 (br s, 1H); **^{13}C NMR** (151 MHz, DMSO-d_6) δ 13.77, 22.32, 25.27, 26.14, 26.22, 27.71, 27.95, 29.05, 29.61, 30.59, 31.14, 31.45, 36.02, 40.98, 44.64, 48.60, 48.71, 48.98, 53.10, 67.83, 68.03, 70.00, 70.21, 106.79, 109.26, 110.54, 115.21, 117.19, 125.47, 129.45, 132.36, 136.07, 136.39, 142.22, 143.36, 144.93, 146.58, 154.92, 158.40, 158.68, 160.92, 167.47, 169.00, 170.16, 170.23, 171.38, 172.95, 202.59; **LC-MS** (ESI) (90% H_2O to 100% MeCN in 10 min, then 100% MeCN to 20 min, DAD 220-500 nm), t_R = 8.63 min, 98% purity, *m/z* $[M + H]^+$ calcd for $C_{51}H_{63}N_{11}O_{10}$, 990.48; found, 990.9; **HRMS** (ESI) *m/z* $[M + H]^+$ calcd for $C_{51}H_{63}N_{11}O_{10}$, 990.4832; found, 990.4852.

CRBN-6-5-5 building block (45)

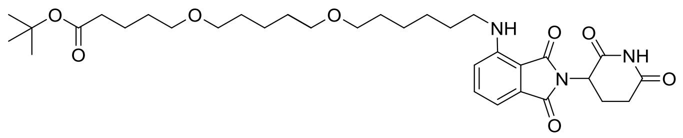

This compound was synthesized as we described previously.[8]

CRBN-based PROTAC 16

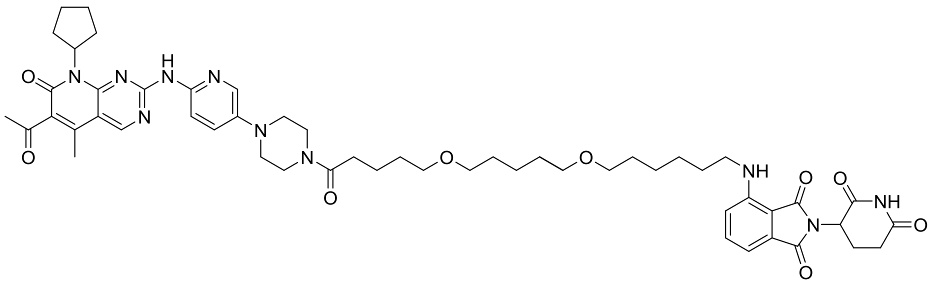

This compound was prepared using the General Procedure **I** and CRBN building block **45** (123 mg). The crude product was purified by column chromatography (gradient of EtOAc to EtOAc/EtOH 9:1) to obtain a yellow solid.

Yield (123 mg, 62%); mp 110 – 112 °C; R_f = 0.60 (EtOAc/EtOH 9:1); **^{1}H NMR** (600 MHz, DMSO-d_6) δ 1.23 – 1.38 (m, 6H), 1.41 – 1.62 (m, 14H), 1.71 – 1.81 (m, 2H), 1.82 – 1.94 (m, 2H), 1.97 – 2.05 (m, 1H), 2.17 – 2.27 (m, 2H), 2.29 (s, 3H), 2.35 (t, *J* = 7.2 Hz, 2H), 2.41 (s, 3H), 2.44 – 2.63 (m, 2H), 2.80 – 2.93 (m, 1H), 3.06 – 3.18 (m, 4H), 3.21 – 3.42 (m, 10H), 3.59 (q, *J* = 4.9, 5.5 Hz, 4H), 5.03 (dd, *J* = 5.5, 12.8 Hz, 1H), 5.81 (p, *J* = 8.9 Hz, 1H), 6.48 (t, *J* = 5.9 Hz, 1H), 6.99 (d, *J* = 7.0 Hz, 1H), 7.05 (d, *J* = 8.6 Hz, 1H), 7.48 (dd, *J* = 3.1, 9.1 Hz, 1H), 7.55 (dd, *J* = 7.1, 8.5 Hz, 1H), 7.87 (d, *J* = 9.0 Hz, 1H), 8.06 (d, *J* = 3.1 Hz, 1H), 8.93 (s, 1H), 10.07 (s, 1H), 11.06 (br s, 1H); **^{13}C NMR** (151 MHz, DMSO-d_6) δ 13.79, 21.88, 22.34, 22.73, 25.29, 25.62, 26.30, 27.73, 28.82, 28.99, 29.23, 29.26, 29.33, 31.15, 31.47, 32.18, 40.23, 40.86, 41.98, 44.82, 48.71, 48.73, 49.13, 53.13, 69.89, 70.01, 70.07, 106.81, 109.19, 110.55, 115.20, 117.32, 125.51, 129.48, 132.35, 136.11, 136.44, 142.24, 143.38, 144.97, 146.61, 154.94, 158.40, 158.69, 160.93, 167.48, 169.13, 170.24, 170.84, 172.97, 202.63; **LC-MS** (ESI) (90% H_2O to 100% MeCN in 10 min, then 100% MeCN to 20 min, DAD 220-400 nm), t_R = 10.98 min, 99% purity, *m/z* $[M + H]^+$ calcd for $C_{53}H_{68}N_{10}O_9$, 989.52; found, 989.7; **HRMS** (ESI) *m/z* $[M + H]^+$ calcd for $C_{53}H_{68}N_{10}O_9$, 989.5262; found, 989.5244.

CRBN-6-6-6 building block (46)

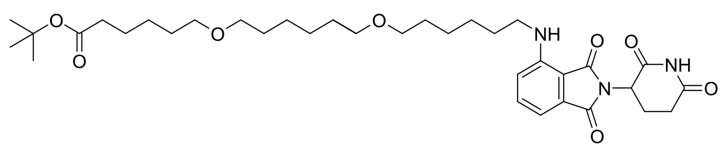

This compound was synthesized as we described previously.[8]

CRBN-based PROTAC 17

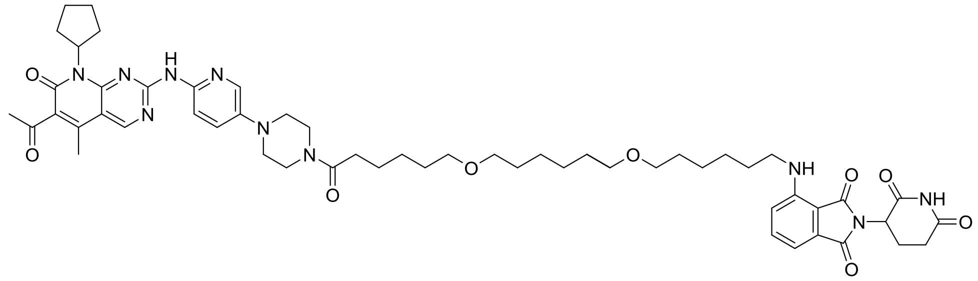

This compound was prepared using the General Procedure **I** and CRBN building block **46** (128 mg). The crude product was purified by column chromatography (gradient of EtOAc to EtOAc/EtOH 9:1) to obtain a yellow solid.

Yield (118 mg, 58%); mp 110 – 112 °C; R_f = 0.65 (CH_2Cl_2/EtOH 9:1); **^{1}H NMR** (600 MHz, DMSO-d_6) δ 1.21 – 1.37 (m, 10H), 1.39 – 1.60 (m, 14H), 1.71 – 1.81 (m, 2H), 1.82 – 1.94 (m, 2H), 2.01 (ddd, *J* = 3.1, 5.5, 10.4 Hz, 1H), 2.19 – 2.27 (m, 2H), 2.29 (s, 3H), 2.33 (t, *J* = 7.4 Hz, 2H), 2.41 (s, 3H), 2.46 – 2.63 (m, 2H), 2.80 – 2.92 (m, 1H), 3.04 – 3.20 (m, 4H), 3.21 – 3.47 (m, 10H), 3.51 – 3.70 (m, 4H), 5.03 (dd, *J* = 5.5, 12.8 Hz, 1H), 5.81 (p, *J* = 9.0 Hz, 1H), 6.48 (t, *J* = 5.9 Hz, 1H), 7.00 (d, *J* = 7.0 Hz, 1H), 7.06 (d, *J* = 8.6 Hz, 1H), 7.48 (dd, *J* = 3.0, 9.1 Hz, 1H), 7.55 (dd, *J* = 7.0, 8.6 Hz, 1H), 7.87 (d, *J* = 9.0 Hz, 1H), 8.06 (d, *J* = 3.0 Hz, 1H), 8.94 (s, 1H), 10.07 (s, 1H), 11.06 (br s, 1H); **^{13}C NMR** (151 MHz, DMSO-d_6) δ 13.79, 22.34, 24.84, 25.29, 25.63, 25.74, 26.30, 27.73, 28.83, 29.27, 29.33, 29.38, 29.40, 31.15, 31.48, 32.37, 40.23, 40.87, 41.98, 44.84, 48.70, 48.73, 49.14, 53.13, 70.00, 70.05, 70.08, 106.82, 109.20, 110.56, 115.23, 117.33, 125.50, 129.48, 132.36, 136.11, 136.45, 142.24, 143.39, 144.96, 146.62, 154.94, 158.42, 158.71, 160.94, 167.49, 169.14, 170.24, 170.87, 172.98, 202.63; **LC-MS** (ESI) (90% H_2O to 100% MeCN in 10 min, then 100% MeCN to 20 min, DAD 220-400 nm), t_R = 11.72 min, 99% purity, *m/z* $[M + H]^+$ calcd for $C_{55}H_{72}N_{10}O_9$, 1017.55; found, 1017.4; **HRMS** (ESI) *m/z* $[M + H]^+$ calcd for $C_{55}H_{72}N_{10}O_9$, 1017.5557; found, 1017.5563.

CRBN-4-4-4-6 building block (47)

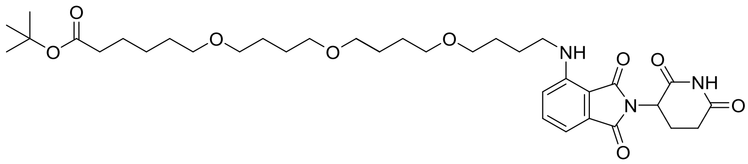

This compound was synthesized as we described previously.[8]

CRBN-based PROTAC 18

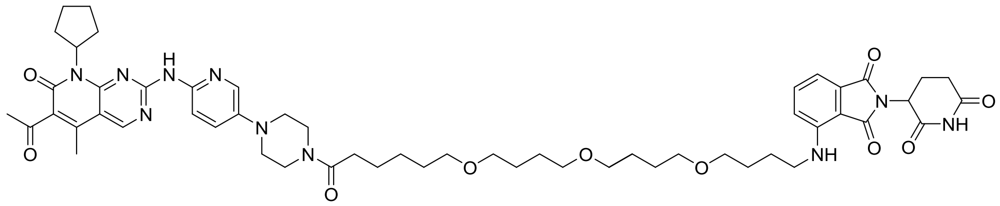

This compound was prepared using the General Procedure **I** and CRBN building block **47** (132 mg). The crude product was purified by column chromatography (gradient of EtOAc to EtOAc/EtOH 19:1) to obtain a yellow solid.

Yield (151 mg, 75%); mp 96 – 100 °C; R_f = 0.30 (CH_2Cl_2/EtOH 19:1); **^{1}H NMR** (600 MHz, DMSO-d_6) δ 1.26 – 1.38 (m, 2H), 1.41 – 1.64 (m, 18H), 1.69 – 1.79 (m, 2H), 1.82 – 1.94 (m, 2H), 1.97 – 2.05 (m, 1H), 2.19 – 2.27 (m, 2H), 2.30 (s, 3H), 2.33 (t, *J* = 7.5 Hz, 2H), 2.41 (s, 3H), 2.44 – 2.61 (m, 2H), 3.21 – 3.41 (m, 14H), 2.74 – 2.92 (m, 1H), 3.04 – 3.19 (m, 4H), 3.51 – 3.66 (m, 4H), 5.03 (dd, *J* = 5.5, 12.7 Hz, 1H), 5.81 (p, *J* = 8.8 Hz, 1H), 6.52 (t, *J* = 6.1 Hz, 1H), 6.99 (d, *J* = 7.0 Hz, 1H), 7.06 (d, *J* = 8.6 Hz, 1H), 7.47 (dd, *J* = 3.1, 9.1 Hz, 1H), 7.55 (dd, *J* = 7.0, 8.5 Hz, 1H), 7.87 (d, *J* = 9.0 Hz, 1H), 8.06 (d, *J* = 3.0 Hz, 1H), 8.94 (s, 1H), 10.06 (s, 1H), 11.05 (br s, 1H); **^{13}C NMR** (126 MHz, DMSO-d_6) δ 13.74, 22.31, 24.80, 25.24, 25.71 , 25.75, 26.23, 26.65, 27.69, 29.24, 31.12, 31.42, 32.33, 40.83, 41.78, 44.80, 48.70, 49.10, 53.11, 69.69, 69.86, 69.91, 69.94, 70.00, 106.78, 109.22, 110.50, 115.16, 117.29, 125.45, 129.45, 132.35, 136.08, 136.35, 142.19, 143.35, 144.92, 146.55, 154.91, 158.35, 158.66, 160.90, 167.43, 169.06, 170.17, 170.81, 172.90, 202.53; **LC-MS** (ESI) (90% H_2O to 100% MeCN in 10 min, then 100% MeCN to 20 min, DAD 220-500 nm), t_R = 10.72 min, 99% purity, *m/z* $[M + H]^+$ calcd for $C_{55}H_{72}N_{10}O_{10}$, 1017.55; found, 1033.4; **HRMS** (ESI) *m/z* $[M + H]^+$ calcd for $C_{55}H_{72}N_{10}O_{10}$, 1033.5513; found, 1033.5506.

CRBN-6-(4)$_3$-6 building block (48)

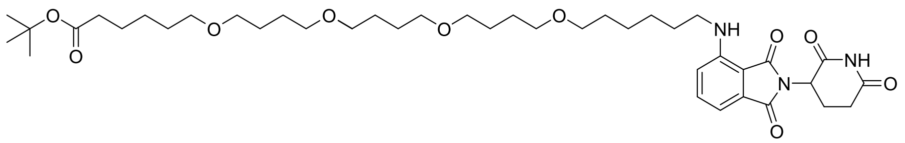

This compound was synthesized as we described previously.[8]

CRBN-based PROTAC 19

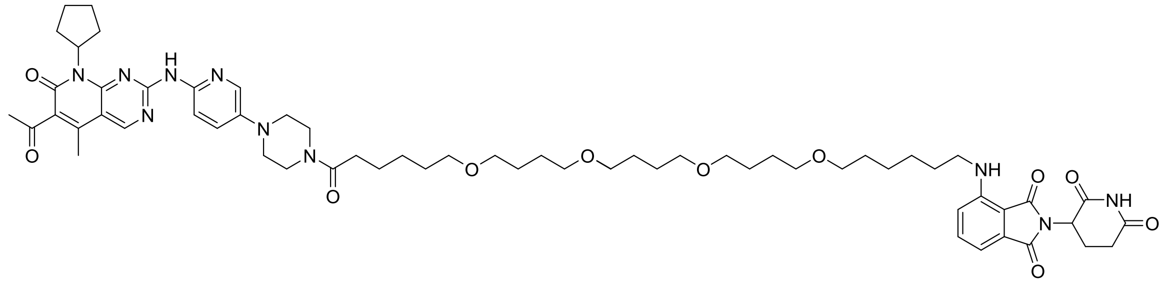

This compound was prepared using the General Procedure **I** and CRBN building block **48** (152 mg). The crude product was purified by column chromatography (gradient of EtOAc to EtOAc/EtOH 9:1) to obtain a yellow solid.

Yield (172 mg, 76%); mp 68 – 72 °C; R_f = 0.57 (EtOAc/EtOH 9:1); **^{1}H NMR** (600 MHz, DMSO-d_6) δ 1.27 – 1.33 (m, 6H), 1.41 – 1.61 (m, 22H), 1.71 – 1.81 (m, 2H), 1.83 – 1.93 (m, 2H), 1.97 – 2.05 (m, 1H), 2.19 – 2.28 (m, 2H), 2.29 (s, 3H), 2.33 (t, *J* = 7.4 Hz, 2H), 2.41 (s, 3H), 2.50 – 2.61 (m, 2H), 2.82 – 2.90 (m, 1H), 3.07 – 3.18 (m, 4H), 3.22 – 3.40 (m, 18H), 3.60 (d, *J* = 5.1 Hz, 4H), 5.03 (dd, *J* = 5.4, 12.8 Hz, 1H), 5.82 (q, *J* = 8.8 Hz, 1H), 6.49 (t, *J* = 5.9 Hz, 1H), 7.00 (d, *J* = 7.0 Hz, 1H), 7.06 (d, *J* = 8.6 Hz, 1H), 7.48 (dd, *J* = 3.1, 9.0 Hz, 1H), 7.55 (dd, *J* = 7.0, 8.6 Hz, 1H), 7.87 (d, *J* = 9.0 Hz, 1H), 8.06 (d, *J* = 3.1 Hz, 1H), 8.94 (s, 1H), 10.08 (s, 1H), 11.06 (br s, 1H); **^{13}C NMR** (151 MHz, DMSO-d_6) δ 13.77, 22.33, 24.83, 25.27, 25.63, 25.74, 26.24, 26.29, 27.72, 28.82, 29.27, 29.33, 31.14, 31.45, 32.36, 40.23, 40.85, 41.98, 44.82, 48.70, 48.72, 49.13, 53.11, 69.87, 69.90, 69.93, 69.98, 70.02, 106.80, 109.19, 110.53, 115.17, 117.30, 125.48, 129.47, 132.35, 136.10, 136.42, 142.22, 143.37, 144.96, 146.60, 154.92, 158.39, 158.68, 160.92, 167.46, 169.11, 170.21, 170.83, 172.94, 202.58; **LC-MS** (ESI) (90% H_2O to 100% MeCN in 10 min, then 100% MeCN to 20 min, DAD 220-500 nm), t_R = 11.94 min, 99% purity, *m/z* $[M + H]^+$ calcd for $C_{61}H_{84}N_{10}O_{11}$, 1017.55; found, 1133.9; **HRMS** (ESI) *m/z* $[M + H]^+$ calcd for $C_{61}H_{84}N_{10}O_{11}$, 1133.6430; found, 1133.6394.

CRBN-6-(2)$_5$-6 building block (49)

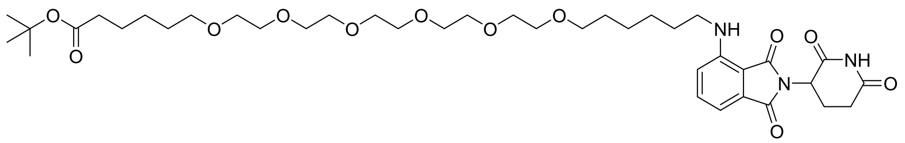

This compound was synthesized as we described previously.[8]

CRBN-based PROTAC 20

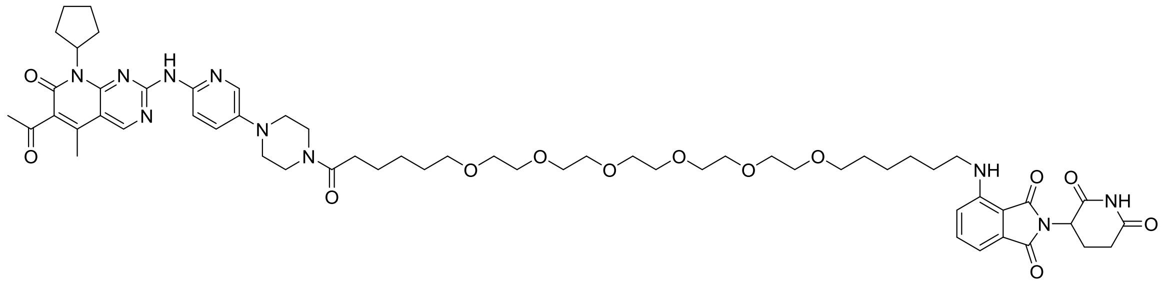

This compound was prepared using the General Procedure **I** and CRBN building block **49** (152 mg). The crude product was purified by column chromatography (gradient of EtOAc to EtOAc/EtOH 9:1) to obtain a yellow solid.

Yield (172 mg, 52%); mp 80 – 82 °C; R_f = 0.67 (EtOAc/EtOH 9:1); **^{1}H NMR** (600 MHz, DMSO-d_6) δ 1.26 – 1.40 (m, 6H), 1.40 – 1.63 (m, 10H), 1.71 – 1.81 (m, 2H), 1.81 – 1.94 (m, 2H), 1.96 – 2.06 (m, 1H), 2.18 – 2.27 (m, 2H), 2.29 (s, 3H), 2.33 (t, *J* = 7.5 Hz, 2H), 2.41 (s, 3H), 2.50 – 2.62 (m, 2H), 2.79 – 2.92 (m, 1H), 3.06 – 3.19 (m, 4H), 3.23 – 3.29 (m, 2H), 3.29 – 3.39 (m, 4H), 3.41 – 3.50 (m, 20H), 3.55 – 3.64 (m, 4H), 5.03 (dd, *J* = 5.5, 12.8 Hz, 1H), 5.81 (p, *J* = 8.9 Hz, 1H), 6.48 (t, *J* = 5.9 Hz, 1H), 6.99 (d, *J* = 7.0 Hz, 1H), 7.06 (d, *J* = 8.6 Hz, 1H), 7.48 (dd, *J* = 3.1, 9.1 Hz, 1H), 7.55 (dd, *J* = 7.1, 8.6 Hz, 1H), 7.87 (d, *J* = 9.0 Hz, 1H), 8.06 (d, *J* = 3.1 Hz, 1H), 8.93 (s, 1H), 10.07 (s, 1H), 11.06 (br s, 1H); **^{13}C NMR** (151 MHz, DMSO-d_6) δ 13.79, 22.35, 24.83, 25.29, 25.58, 25.67, 26.32, 27.74, 28.84, 29.25, 29.31, 31.16, 31.48, 32.38, 40.23, 40.88, 42.00, 44.84, 48.71, 48.74, 49.14, 53.14, 69.66, 69.68, 69.98, 70.00, 70.41, 70.45, 106.82, 109.20, 110.57, 115.22, 117.34, 125.53, 129.49, 132.37, 136.12, 136.46, 142.25, 143.40, 144.96, 146.62, 154.95, 158.42, 158.70, 160.94, 167.50, 169.14, 170.25, 170.88, 172.99, 202.64; **LC-MS** (ESI) (90% H_2O to 100% MeCN in 10 min, then 100% MeCN to 20 min, DAD 220-400 nm), t_R = 9.82 min, 99% purity, *m/z* [M + H]$^+$ calcd for $C_{59}H_{80}N_{10}O_{13}$, 1017.55; found, 1137.3; **HRMS** (ESI) *m/z* [M + H]$^+$ calcd for $C_{59}H_{80}N_{10}O_{13}$, 1137.5979; found, 1137.5838.

4-Fluoro-2-(1-methyl-2,6-dioxo-3-piperidyl)isoindoline-1,3-dione (50)

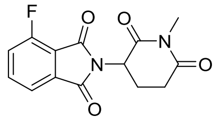

This compound was synthesized as we described previously.[8]

(–)CRBN-6 building block (51)

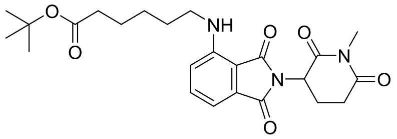

Compound **38** (161 mg, 0.5 mmol) was dissolved in dry EtOAc (10 mL) and treated with 10% Pd/C (10% m/m). The reaction mixture was stirred under H_2 (1 atm, balloon) overnight. The mixture was filtered through celite and the filtrate was concentrated. The oily residue was dissolved in dry DMSO (5 mL) and DIPEA (0.13 g, 0.17 mL, 1 mmol) as well as compound **50** (145 mg, 0.5 mmol) were added. The mixture was stirred at 90 °C for 24 h. After cooling, it was poured onto half-saturated brine (50 mL) and it was extracted with EtOAc (2 × 50 mL). The combined organic layers were washed with saturated NH_4Cl solution (50 mL) 5% LiCl solution (50 mL) and brine (50 mL), dried over Na_2SO_4, filtered and concentrated. The crude product was purified by column chromatography (petroleum ether/EtOAc 2:1) to obtain a green oil.

Yield (79 mg, 35%); R_f = 0.38 (petroleum ether/EtOAc 2:1); ^{1}**H NMR** (600 MHz, DMSO-d_6) δ 1.33 (dd, *J* = 6.5, 8.7 Hz, 2H, CH_2), 1.36 (s, 9H, CH_3), 1.48 – 1.61 (m, 4H, CH_2), 1.99 – 2.07 (m, 1H, 4’-H), 2.18 (t, *J* = 7.3 Hz, 2H, CH_2), 2.49 – 2.57 (m, 1H, 4’-H), 2.71 – 2.78 (m, 1H, 5’-H), 2.86 – 2.98 (m, 1H, 5’-H), 3.01 (s, 3H, CH_3), 3.28 (d, *J* = 6.4 Hz, 2H, $NHC\underline{H_2}$), 5.10 (dd, *J* = 5.4, 13.0 Hz, 1H, 3’-H), 6.52 (t, *J* = 6.0 Hz, 1H, $NHCH_2$), 7.01 (d, *J* = 7.0 Hz, 1H), 7.08 (d, *J* = 8.6 Hz, 1H, 5-H, 7-H), 7.57 (dd, *J* = 7.0, 8.5 Hz, 1H, 6-H); ^{13}**C NMR** (151 MHz, DMSO-d_6) δ 21.55 (C-4’), 24.51, 25.85 (CH_2), 26.74 (CH_3), 27.90 ($C(\underline{C}H_3)_3$), 28.53 (CH_2), 31.27 (C-5’), 34.84 (CH_2), 41.85 ($NHCH_2$), 49.26 (C-3’), 79.54 ($\underline{C}(CH_3)_3$), 109.17 (C-3a), 110.56 (C-7), 117.38 (C-5), 132.35 (C-7a), 136.46 (C-6), 146.62 (C-4), 167.44 (C-1), 169.07 (C-3), 169.97 (C-2’), 171.96, 172.37 (C-6’, CO); **MS** (ESI) *m/z* $[M + H]^+$ calcd for $C_{24}H_{31}N_3O_6$, 458.22; found, 458.3; **HRMS** (ESI) *m/z* $[M + H]^+$ calcd for $C_{24}H_{31}N_3O_6$, 458.2286; found, 458.2285.

CRBN-based negative control 21

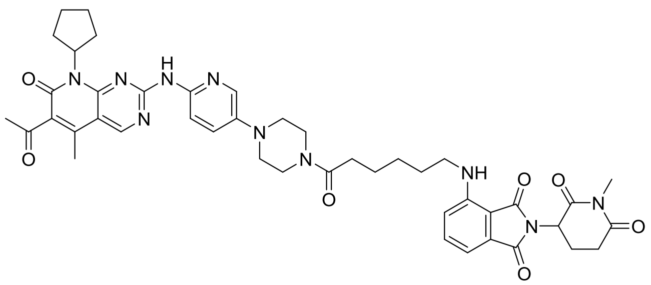

This compound was prepared using the General Procedure **I** and CRBN building block **51** (92 mg). The crude product was purified by column chromatography (CH_2Cl_2/EtOH 29:1) to obtain a yellow solid.

Yield (140 mg, 84%); mp 102 – 106 °C; R_f = 0.40 (CH_2Cl_2/EtOH 19:1); **^{1}H NMR** (600 MHz, DMSO-d_6) δ 1.31 – 1.46 (m, 2H), 1.46 – 1.66 (m, 6H), 1.66 – 1.83 (m, 2H), 1.84 – 1.94 (m, 2H), 1.99 – 2.08 (m, 1H), 2.17 – 2.28 (m, 2H), 2.30 (s, 3H), 2.36 (t, *J* = 7.4 Hz, 2H), 2.41 (s, 3H), 2.49 – 2.56 (m, 1H), 2.63 – 2.84 (m, 1H), 2.89 – 2.98 (m, 1H), 3.00 (s, 3H), 3.03 – 3.21 (m, 4H), 3.26 – 3.30 (m, 2H), 3.41 – 3.78 (m, 4H), 5.10 (dd, *J* = 5.4, 13.0 Hz, 1H), 5.81 (p, *J* = 8.9 Hz, 1H), 6.52 (t, *J* = 5.9 Hz, 1H), 7.00 (d, *J* = 7.0 Hz, 1H), 7.09 (d, *J* = 8.6 Hz, 1H), 7.47 (dd, *J* = 3.1, 9.1 Hz, 1H), 7.56 (dd, *J* = 7.0, 8.6 Hz, 1H), 7.87 (d, *J* = 9.1 Hz, 1H), 8.06 (d, *J* = 3.0 Hz, 1H), 8.94 (s, 1H), 10.08 (s, 1H); **^{13}C NMR** (151 MHz, DMSO-d_6) δ 13.76, 21.53, 24.67, 25.26, 26.23, 26.74, 27.70, 28.70, 31.27, 31.44, 32.25, 40.84, 41.93, 44.79, 48.67, 49.09, 49.26, 53.09, 106.78, 109.13, 110.54, 115.19, 117.36, 125.47, 129.45, 132.34, 136.08, 136.46, 142.21, 143.35, 144.93, 146.62, 154.91, 158.39, 158.67, 160.90, 167.41, 169.07, 169.97, 170.76, 171.94, 202.56; **LC-MS** (ESI) (90% H_2O to 100% MeCN in 10 min, then 100% MeCN to 20 min, DAD 220-500 nm), t_R = 9.67 min, 99% purity, *m/z* $[M + H]^+$ calcd for $C_{43}H_{48}N_{10}O_7$, 831.39; found, 831.5; **HRMS** (ESI) *m/z* $[M + H]^+$ calcd for $C_{43}H_{48}N_{10}O_7$, 831.3937; found, 831.3928.

J Synthesis of VHL-based PROTACs

General Procedure II: TEMPO/BAIB mediated oxidation of alcohols to carboxylic acids. The corresponding alcohol (5 mmol) was dissolved in acetonitrile (12.5 mL) and H_2O (12.5 mL). TEMPO 0.17 g, 1.1 mmol) was added followed by the portionwise addition of (diacetoxyiodo)benzene (3.54 g, 11 mmol). The orange mixture was stirred at rt for 16 h. It was neutralized by the addition of saturated $NaHCO_3$ solution (100 mL) and the aqueous layer was washed with EtOAc (2 × 100 mL). The aqueous phase was then acidified by the careful addition of 2N HCl solution until *p*H = 1. The mixture was then extracted with EtOAc (2 × 100 mL) and the combined organic layers were dried over Na_2SO_4, filtered and concentrated.

General Procedure III: HATU-mediated coupling to the VHL ligand. The corresponding VHL ligand (1 mmol) was dissolved in dry CH_2Cl_2 (5 mL) and TFA (5 mL) was added. The mixture was stirred at room temperature for 2 h. After removal of the volatiles, the oily residue was further dried under high vacuum. Subsequently, the appropriate acid (1 mmol) was dissolved in dry DMF (5 mL) and DIPEA (0.35 mL, 2 mmol) was added, followed by the addition of HATU (1.1 mmol). After stirring for 5 minutes, the deprotected amine was dissolved in dry DMF (5 mL) and DIPEA (0.70 mL, 4 mmol) and was added to the mixture. It was stirred at room temperature for 16 h, after which half-saturated brine (50 mL) was added, and the product was extracted with EtOAc (3 × 50 mL). The combined organic phases were washed with saturated NH_4Cl solution (50 mL), 5% LiCl solution (50 mL), brine (50 mL), dried over Na_2SO_4, filtered and concentrated *in vacuo*.

General Procedure IV: Finkelstein reaction and alkylation of palbociclib. The corresponding VHL-linker conjugate (0.1 mmol) was dissolved in dry acetone (5 mL) and sodium iodide (150 mg, 1 mmol) was added. After stirring for 48 h, the yellow suspension was diluted with EtOAc (50 mL) and it was washed with 10% Na_2SO_3 solution, H_2O and brine (each 25 mL), dried over Na_2SO_4, filtered and concentrated. The intermediate was then dissolved in dry DMSO (4 mL) and dry DMF (4 ml), followed by the addition of DIPEA (39 mg, 52 µL, 0.3 mmol) and palbociclib (45 mg, 0.1 mmol). The yellow suspension was stirred at 80 °C for 24 h. After cooling, the yellow solution was diluted with EtOAc (100 mL) and it was washed with saturated $NaHCO_3$ solution (50 mL), 5% LiCl solution (50 mL), and brine (50 mL). The organic layer was dried over Na_2SO_4, filtered and concentrated.

General Procedure V: Alkylation of phenolic VHL ligands. The corresponding VHL ligand (0.30 mmol) and the corresponding mesylate (0.36 mmol) were dissolved in dry DMF (6 mL) and Cs_2CO_3 (147 mg, 0.45 mmol) was added. After stirring for 18 h at rt, the beige suspension was heated at 60 °C for 3 h. After cooling, it was diluted with H_2O (50 mL) and extracted with EtOAc (3 × 25 mL). The combined organic layers were washed with 5% LiCl solution and brine (each 25 mL), dried over Na_2SO_4, filtered and concentrated.

8-Chlorooctanoic acid (52)

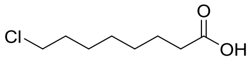

This compound was prepared using the General Procedure **II** and 8-chloro-1-octanol (0.82 g). The crude product was purified by column chromatography (gradient of CH_2Cl_2 to CH_2Cl_2/MeOH 19:1) to obtain a colorless oil.

Yield (0.33 g, 37%); R_f = 0.48 (CH_2Cl_2/MeOH 9:1); **^{1}H NMR** (500 MHz, DMSO-d_6) δ 1.20 – 1.35 (m, 4H), 1.36 (t, J = 7.3 Hz, 2H), 1.43 – 1.53 (m, 2H), 1.64 – 1.74 (m, 2H), 2.18 (t, J = 7.4 Hz, 2H), 3.61 (t, J = 6.6 Hz, 2H, CH_2), 11.86 (br s, 1H, COOH); **^{13}C NMR** (126 MHz, DMSO-d_6) δ 24.55, 26.28, 28.08, 28.54, 32.16, 33.80 (CH_2), 45.54 (CH_2Cl), 174.65 (CO); **HRMS** (ESI) m/z $[M - H]^-$ calcd for $C_8H_{15}ClO_2$, 177.0688; found, 177.0680.

VHL ligand a (53)

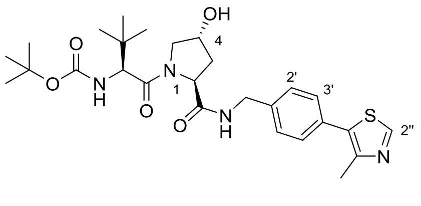

This compound was synthesized as we described previously.[8]

VHL(a)-8 building block (54)

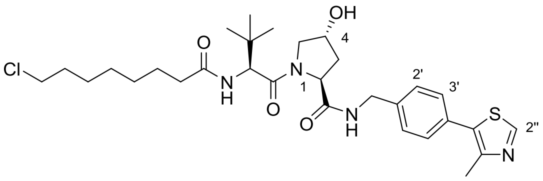

This compound was prepared using the General Procedure **III**, VHL ligand a (**53**, 0.53 g) and acid **52** (0.18 g). The crude product was purified by column chromatography (gradient of CH_2Cl_2/EtOH 29:1 to 19:1) to obtain a colorless solid.

Yield (0.46 g, 78%); mp 62 – 66 °C, R_f = 0.50 (CH_2Cl_2/MeOH 9:1); **^{1}H NMR** (600 MHz, DMSO-d_6) δ 0.92 (s, 9H, C(CH_3)$_3$), 1.18 – 1.30 (m, 4H), 1.30 – 1.38 (m, 2H), 1.39 – 1.55 (m, 2H), 1.62 – 1.72 (m, 2H, CH_2), 1.86 – 1.93 (m, 1H, 3-H), 1.98 – 2.05 (m, 1H, 3-H), 2.06 – 2.14 (m, 1H), 2.21 – 2.29 (m, 1H, CH_2), 2.43 (s, 3H, CH_3), 3.54 – 3.70 (m, 4H, 5-H, CH_2Cl), 4.21 (dd, *J* = 5.5, 15.8 Hz, 1H), 4.32 – 4.36 (m, 1H), 4.39 – 4.45 (m, 2H), 4.53 (d, *J* = 9.4 Hz, 1H, 2-H, 4-H, NHC$\underline{H}$, NHC$\underline{H}_2$), 5.09 (d, *J* = 3.6 Hz, 1H, OH), 7.35 – 7.43 (m, 4H, 2′-H, 3′-H), 7.80 (d, *J* = 9.4 Hz, 1H), 8.52 (t, *J* = 6.1 Hz, 1H, CONH), 8.97 (s, 1H, 2′′-H); **^{13}C NMR** (151 MHz, DMSO-d_6) δ 16.09 (CH_3), 25.46, 26.32 (CH_2), 26.54 (C($\underline{C}H_3$)$_3$), 28.07, 28.63, 32.17, 34.97 (CH_2), 35.37 ($\underline{C}$(CH_3)$_3$), 38.11 (C-3), 41.82 (NHCH_2), 45.54 (CH_2Cl), 56.45, 56.49, 58.85 (C-2, C-5, NHCH), 69.03 (C-4), 127.61, 128.81 (C-2′, C-3′), 129.82, 131.34 (C-1′, C-5′′), 139.68 (C-4′), 147.90 (C-4′′), 151.61 (C-2′′), 169.90, 172.11, 172.26 (CO); **MS** (ESI) *m/z* [M + H]$^+$ calcd for $C_{30}H_{43}ClN_4O_4S$, 591.27; found, 591.2; **HRMS** (ESI) *m/z* [M + H]$^+$ calcd for $C_{30}H_{43}ClN_4O_4S$, 591.2766; found, 591.2762.

VHL(a)-based PROTAC 22

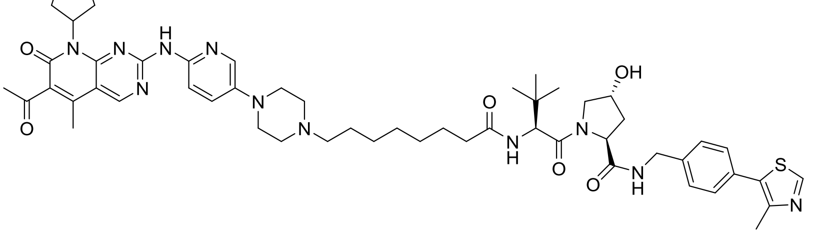

This compound was prepared using the General Procedure **IV** and precursor **54** (59 mg). The crude product was purified by column chromatography (gradient of CH_2Cl_2/MeOH+7N NH_3 29:1 to 19:1) to obtain the title compound as a yellow solid.

Yield (34 mg, 34%); mp 96 – 98 °C; R_f = 0.38 (CH_2Cl_2/MeOH+7N NH_3 9:1); **^{1}H NMR** (600 MHz, DMSO-d_6) δ; 0.92 (s, 9H), 1.16 – 1.34 (m, 7H), 1.35 – 1.65 (m, 7H), 1.72 – 1.78 (m, 2H), 1.84 – 1.92 (m, 3H), 1.98 – 2.37 (m, 12H), 2.41 (s, 3H), 2.43 (s, 3H), 3.10 – 3.16 (m, 4H), 3.58 – 3.71 (m, 2H), 4.20 (dd, *J* = 5.5, 15.9 Hz, 1H), 4.34 (p, *J* = 2.7, 3.2 Hz, 1H), 4.37 – 4.45 (m, 2H), 4.53 (d, *J* = 9.3 Hz, 1H), 5.10 (d, *J* = 3.6 Hz, 1H), 5.80 (p, *J* = 8.9 Hz, 1H), 7.35 – 7.45 (m, 5H), 7.81 (dd, *J* = 9.2, 13.1 Hz, 2H), 8.03 (d, *J* = 3.0 Hz, 1H), 8.52 (t, *J* = 6.1 Hz, 1H), 8.93 (s, 1H), 8.96 (s, 1H), 10.04 (s, 1H); **^{13}C NMR** (151 MHz, DMSO-d_6) δ 13.76, 16.08, 25.24, 25.55, 26.38, 26.54, 27.02, 27.70, 28.78, 28.82, 31.45, 35.03, 35.36, 38.10, 41.81, 48.50, 52.72, 53.06, 56.45, 56.48, 58.02, 58.85, 69.01, 106.71, 115.34, 124.77, 127.59, 128.79, 129.36, 129.80, 131.31, 135.44, 139.65, 142.24, 143.66, 144.37, 147.87, 151.58, 154.92, 158.40, 158.74, 160.92, 169.89, 172.09, 172.28, 202.60; **LC-MS** (ESI) (90% H_2O to 100% MeCN in 10 min, then 100% MeCN to 20 min, DAD 220-420 nm), t_R = 9.34 min, 99% purity, *m/z* $[M + H]^+$ calcd for $C_{54}H_{71}N_{11}O_6S$, 1002.53; found, 1002.8; **HRMS** (ESI) *m/z* $[M + H]^+$ calcd for $C_{54}H_{71}N_{11}O_6S$, 1002.5382; found, 1002.5428.

2-[2-(2-Chloroethoxy)ethoxy]acetic acid (55)

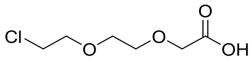

This compound was prepared using the General Procedure **II** and 2-[2-(2-chloroethoxy) ethoxy]ethanol (0.84 g). The crude product was purified by column chromatography (gradient of CH_2Cl_2 to CH_2Cl_2/MeOH 9:1) to obtain a colorless oil.

Yield (0.74 g, 81%); R_f = 0.25 (CH_2Cl_2/MeOH 9:1); **^{1}H NMR** (500 MHz, DMSO-d_6) δ 3.55 – 3.62 (m, 4H), 3.63 – 3.74 (m, 4H, CH_2Cl, OCH_2), 4.01 (s, 2H, OCH_2), 12.27 (br s, 1H, COOH); **^{13}C NMR** (126 MHz, DMSO-d_6) δ 43.68 (CH_2Cl), 67.84, 69.79, 69.95, 70.65 (OCH_2), 171.79 (CO); **MS** (ESI) *m/z* $[M + H]^+$ calcd for $C_6H_{11}ClO_4$, 183.04; found, 183.0.

VHL(a)-2-2-2 building block (56)

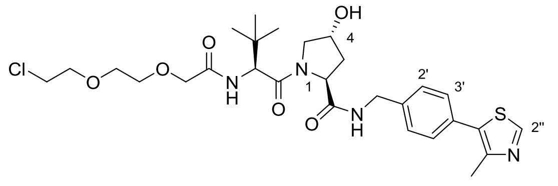

This compound was prepared using the General Procedure **III**, VHL ligand a (**53**, 0.53 g) and acid **55** (0.18 g). The crude product was purified by column chromatography (gradient of CH_2Cl_2/MeOH 29:1 to 19:1) to obtain a colorless solid.

Yield (0.35 g, 59%); mp 52 – 54 °C, R_f = 0.29 (CH_2Cl_2/MeOH 19:1); **^{1}H NMR** (500 MHz, DMSO-d_6) δ 0.93 (s, 9H, $C(CH_3)_3$), 1.86 – 1.94 (m, 1H, 3-H), 2.01 – 2.09 (m, 1H, 3-H), 2.43 (s, 3H, CH_3), 3.56 – 3.75 (m, 10H, 5-H, CH_2Cl, OCH_2), 3.90 – 4.03 (m, 2H, OCH_2), 4.23 (dd, J = 5.6, 15.8 Hz, 1H), 4.34 (d, J = 4.4 Hz, 1H), 4.36 – 4.47 (m, 2H), 4.56 (d, J = 9.6 Hz, 1H, 2-H, 4-H, $NHC\underline{H}$, $NHC\underline{H_2}$), 5.13 (d, J = 3.5 Hz, 1H, OH), 7.35 – 7.46 (m, 5H, 2‘-H, 3‘-H, CONH), 8.55 (t, J = 6.1 Hz, 1H, CONH), 8.96 (s, 1H, 2’’-H); **^{13}C NMR** (126 MHz, DMSO-d_6) δ 16.07 (CH_3), 26.36 ($C(\underline{C}H_3)_3$), 35.88 ($\underline{C}(CH_3)_3$), 38.07 (C-3), 41.88 ($NHCH_2$), 43.57 (CH_2Cl), 55.90 , 56.73, 58.92 (C-2, C-5, NHCH), 69.06 (C-4), 69.63, 69.80, 70.55, 70.80 (OCH_2), 127.64, 128.87 (C-2’, C-3’), 129.89, 131.33 (C-1’, C-5’’), 139.61 (C-4’), 147.93 (C-4’’), 151.61 (C-2’’), 168.79, 169.35, 171.96 (CO); **MS** (ESI) *m/z* $[M + H]^+$ calcd for $C_{28}H_{39}ClN_4O_6S$, 595.23; found, 595.4; **HRMS** (ESI) *m/z* $[M + H]^+$ calcd for $C_{28}H_{39}ClN_4O_6S$, 595.2352; found, 595.2354.

VHL(a)-based PROTAC 23

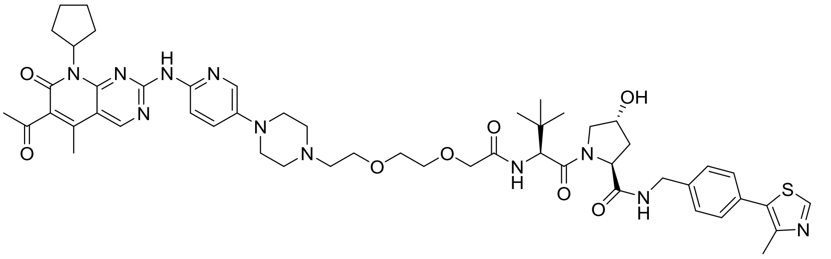

This compound was prepared using the General Procedure **IV** and precursor **56** (60 mg). The crude product was purified by column chromatography (gradient of CH_2Cl_2/MeOH+7N NH_3 29:1 to 19:1) to obtain the title compound as a yellow solid.

Yield (67 mg, 67%); mp 122 – 124 °C; R_f = 0.12 (CH_2Cl_2/MeOH+7N NH_3 19:1); **^{1}H NMR** (500 MHz, DMSO-d_6) δ 0.95 (s, 9H), 1.49 – 1.62 (m, 2H), 1.70 – 1.80 (m, 2H), 1.81 – 1.95 (m, 3H), 2.02 – 2.11 (m, 1H), 2.12 – 2.26 (m, 2H), 2.29 (s, 3H), 2.41 (s, 3H), 2.42 (s, 3H), 2.51 – 2.61 (m, 6H), 3.06 – 3.15 (m, 4H), 3.53 – 3.72 (m, 8H), 3.97 (s, 2H), 4.15 – 4.31 (m, 1H), 4.31 – 4.41 (m, 2H), 4.41 – 4.51 (m, 1H), 4.57 (d, J = 9.5 Hz, 1H), 5.13 (d, J = 3.6 Hz, 1H), 5.75 – 5.86 (m, 1H), 7.31 – 7.47 (m, 6H), 7.83 (d, J = 9.0 Hz, 1H), 8.01 (d, J = 3.2 Hz, 1H), 8.54 (t, J = 6.1 Hz, 1H), 8.92 (s, 1H), 8.94 (s, 1H), 10.02 (s, 1H); **^{13}C NMR** (126 MHz, DMSO-d_6) δ 13.73, 16.03, 25.22, 26.34, 27.68, 31.42, 35.86, 38.04, 41.84, 48.45, 53.01, 53.07, 55.83, 56.70, 57.28, 58.90, 68.60, 69.02, 69.62, 69.80, 70.64, 106.69, 115.26, 124.72, 127.58, 128.80, 129.35, 129.82, 131.26, 135.42, 139.55, 142.20, 143.60, 144.35, 147.86, 151.48, 154.90, 158.34, 158.69, 160.90, 168.72, 169.28, 171.86, 202.52; **LC-MS** (ESI) (90% H_2O to 100% MeCN in 10 min, then 100% MeCN to 20 min, DAD 220-420 nm), t_R = 8.61 min, 97% purity, m/z $[M + H]^+$ calcd for $C_{52}H_{67}N_{11}O_8S$, 1006.49; found, 1006.8; **HRMS** (ESI) m/z $[M + H]^+$ calcd for $C_{52}H_{67}N_{11}O_8S$, 1006.4968; found, 1006.5016.

2-[2-[2-(2-Chloroethoxy)ethoxy]ethoxy]ethanol (57)

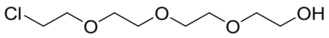

This compound was synthesized similar to a previously reported procedure.[9] In brief, tetraethylene glycol (19.42 g, 100 mmol) was dissolved in $CHCl_3$ (20 mL). Subsequently, pyridine (7.91 g, 8.07 mL, 100 mmol) was added followed by the dropwise addition of $SOCl_2$ (11.90 g, 7.26 mL, 100 mmol) while cooling with a water bath. The mixture was then stirred at 77 °C for 2 h and stirring was continued at 60 °C overnight. All volatiles (including pyridine) were removed *in vacuo* and the residue was partitioned between H_2O (100 mL) and CH_2Cl_2 (100 mL). The aqueous phase was extracted again with CH_2Cl_2 (100 mL) and the combined organic layers were washed with brine (100 mL), dried over Na_2SO_4, filtered and evaporated. The crude product was purified by column chromatography (gradient of petroleum ether/EtOAc 1:2 to EtOAc) to obtain a colourless oil.

Yield (4.45 g, 21%); R_f = 0.30 (EtOAc); **^{1}H NMR** (500 MHz, DMSO-d_6) δ 3.38 – 3.43 (m, 2H), 3.48 (q, J = 5.3 Hz, 2H), 3.48 – 3.66 (m, 8H), 3.63 – 3.73 (m, 4H, OCH_2), 4.53 (t, J = 5.5 Hz, 1H, OH); **^{13}C NMR** (126 MHz, DMSO-d_6) δ 43.72 (CH_2Cl), 60.40 (CH_2OH), 69.86 (OCH_2), 69.94 (2 × OCH_2), 70.01, 70.72, 72.50 (OCH_2); **MS** (ESI) m/z $[M + H]^+$ calcd for $C_8H_{17}ClO_4$, 213.08; found, 213.1.

2-[2-[2-(2-Chloroethoxy)ethoxy]ethoxy]acetic acid (58)

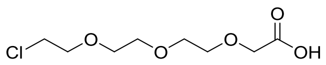

This compound was prepared using the General Procedure **II** and alcohol **57** (1.06 g). The crude product was purified by column chromatography (gradient of CH_2Cl_2 to CH_2Cl_2/MeOH 9:1) to obtain a colorless oil.

Yield (0.52 g, 46%); R_f = 0.28 (CH_2Cl_2/MeOH 9:1); **^{1}H NMR** (600 MHz, DMSO-d_6) δ 3.50 – 3.59 (m, 8H), 3.64 – 3.72 (m, 4H, OCH_2, CH_2Cl), 4.00 (s, 2H, OCH_2), 12.55 (br s, 1H, COOH); **^{13}C NMR** (151 MHz, DMSO-d_6) δ 43.74 (CH_2Cl), 67.81, 69.84, 69.89, 69.94, 70.00, 70.72 (OCH_2), 171.85 (CO); **HRMS** (ESI) *m/z* $[M – H]^-$ calcd for $C_8H_{15}ClO_5$, 225.0535; found, 225.0531.

VHL(a)-2-2-2-2 building block (59)

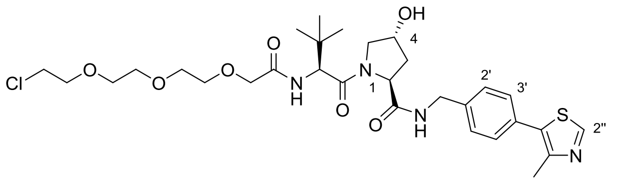

This compound was prepared using the General Procedure **III**, VHL ligand a (**53**, 0.53 g) and acid **58** (0.23 g). The crude product was purified by column chromatography (gradient of CH_2Cl_2/MeOH 29:1 to 19:1) to obtain a colorless solid.

Yield (0.45 g, 70%); mp 52 – 54 °C, R_f = 0.42 (CH_2Cl_2/MeOH 9:1); **^{1}H NMR** (500 MHz, DMSO-d_6) δ 0.94 (s, 9H, $C(CH_3)_3$), 1.86 – 1.94 (m, 1H, 3-H), 2.01 – 2.09 (m, 1H, 3-H), 2.43 (s, 3H, CH_3), 3.53 – 3.71 (m, 14H, 5-H, OCH_2, CH_2Cl), 3.96 (s, 2H, OCH_2), 4.25 (dd, *J* = 5.7, 15.8 Hz, 1H), 4.32 – 4.47 (m, 3H), 4.56 (d, *J* = 9.5 Hz, 1H, 2-H, 4-H, NHC$\underline{H}$, NHC$\underline{H}_2$), 5.12 (d, *J* = 3.7 Hz, 1H, OH), 7.35 – 7.46 (m, 5H, 2'-H, 3'-H, CONH), 8.55 (t, *J* = 6.1 Hz, 1H, CONH), 8.96 (s, 1H, 2''-H); **^{13}C NMR** (126 MHz, DMSO-d_6) δ 16.05 (CH_3), 26.33 ($C(\underline{C}H_3)_3$), 35.86 ($\underline{C}(CH_3)_3$), 38.05 (C-3), 41.86 ($NHCH_2$), 43.69 (CH_2Cl), 55.86, 56.70, 58.89 (C-2, C-5, NHCH), 69.03 (C-4), 69.78 (2 × OCH_2), 69.84, 69.99, 70.62, 70.70 (OCH_2), 127.64, 128.85 (C-2', C-3'), 129.88, 131.29 (C-1', C-5''), 139.60 (C-4'), 147.92 (C-4''), 151.58 (C-2''), 168.75, 169.32, 171.91 (CO); **MS** (ESI) *m/z* $[M + H]^+$ calcd for $C_{30}H_{43}ClN_4O_7S$, 639.26; found, 639.3; **HRMS** (ESI) *m/z* $[M + H]^+$ calcd for $C_{30}H_{43}ClN_4O_7S$, 639.2614; found, 639.2619.

VHL(a)-based PROTAC 24

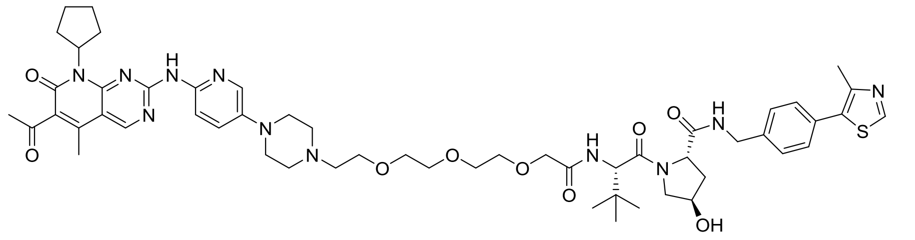

This compound was prepared using the General Procedure **IV** and precursor **59** (64 mg). The crude product was purified by column chromatography (gradient of CH_2Cl_2/MeOH+7N NH_3 29:1 to 19:1) to obtain the title compound as a yellow solid.

Yield (62 mg, 59%); mp 96 – 98 °C; R_f = 0.80 (CH_2Cl_2/MeOH+7N NH_3 9:1); **^{1}H NMR** (600 MHz, DMSO-d_6) δ 0.94 (s, 9H), 1.51 – 1.61 (m, 2H), 1.68 – 1.79 (m, 2H), 1.81 – 1.94 (m, 3H), 2.00 – 2.10 (m, 1H), 2.18 – 2.27 (m, 2H), 2.29 (s, 3H), 2.41 (s, 3H), 2.43 (s, 3H), 2.49 – 2.62 (m, 6H), 3.08 – 3.16 (m, 4H), 3.45 – 3.72 (m, 12H), 3.96 (s, 2H), 4.20 – 4.27 (m, 1H), 4.30 – 4.47 (m, 3H), 4.56 (d, *J* = 9.6 Hz, 1H), 5.13 (d, *J* = 3.6 Hz, 1H), 5.80 (p, *J* = 8.9 Hz, 1H), 7.32 – 7.48 (m, 6H), 7.82 (d, *J* = 9.0 Hz, 1H), 8.03 (d, *J* = 3.0 Hz, 1H), 8.56 (t, *J* = 6.0 Hz, 1H), 8.93 (s, 1H), 8.96 (s, 1H), 10.04 (s, 1H); **^{13}C NMR** (126 MHz, DMSO-d_6) δ 13.75, 16.05, 25.22, 26.33, 27.68, 31.43, 35.86, 38.05, 41.82, 48.43, 53.02, 55.83, 56.70, 57.31, 58.88, 68.51, 69.01, 69.76, 69.84, 69.99, 70.66, 106.69, 115.31, 124.74, 127.59, 128.81, 129.35, 129.83, 131.27, 135.44, 139.58, 142.21, 143.60, 144.37, 147.88, 151.55, 154.90, 158.38, 158.72, 160.90, 168.73, 169.27, 171.89, 202.56; **LC-MS** (ESI) (90% H_2O to 100% MeOH in 10 min, then 100% MeOH to 20 min, DAD 220-400 nm), t_R = 11.83 min, 96% purity, *m/z* $[M + H]^+$ calcd for $C_{54}H_{71}N_{11}O_9S$, 1050.52; found, 1051.0; **HRMS** (ESI) *m/z* $[M + H]^+$ calcd for $C_{54}H_{71}N_{11}O_9S$, 1050.5230; found, 1050.5260.

***tert*-Butyl 5-[5-(6-chlorohexoxy)pentoxy]pentanoate (60)**

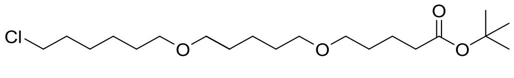

This compound was synthesized as we described previously.[8]

VHL(a)-5-5-6 building block (61)

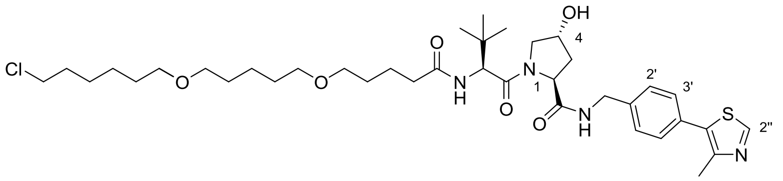

The linker building block **60** (1 mmol, 0.38 g) was dissolved in dry CH_2Cl_2 (5 mL) and TFA (5 mL) was added. The mixture was stirred at 40 °C for 2 h. After removal of the volatiles, the oily residue was further dried under high vacuum. The so obtained free acid was then subjected to the General Procedure **III** with VHL ligand a (**53**, 0.53 g). The crude product was purified by column chromatography (gradient of CH_2Cl_2/MeOH 29:1 to 19:1) to obtain a colorless oil.

Yield (0.66 g, 90%); R_f = 0.35 (CH_2Cl_2/MeOH 19:1); **^{1}H NMR** (600 MHz, DMSO-d_6) δ; 0.92 (s, 9H, $C(CH_3)_3$), 1.26 – 1.33 (m, 4H), 1.33 – 1.41 (m, 2H), 1.41 – 1.56 (m, 10H), 1.65 – 1.73 (m, 2H, CH_2), 1.86 – 1.93 (m, 1H), 2.01 (d, *J* = 9.7 Hz, 1H), 2.07 – 2.15 (m, 1H,), 2.22 – 2.30 (m, 1H, 3-H, $COCH_2$), 2.43 (s, 3H, CH_3), 3.57 – 3.64 (m, 3H), 3.66 (dd, *J* = 4.1, 10.6 Hz, 1H), 4.20 (dd, *J* = 5.6, 15.8 Hz, 1H), 4.34 (s, 1H), 4.39 – 4.45 (m, 2H, 2-H, 4-H, 5-H, NHC$\underline{H}_2$), 4.53 (d, *J* = 9.3 Hz, 1H, NHC$\underline{H}$), 5.09 (d, *J* = 3.7 Hz, 1H, OH), 7.37 (d, *J* = 8.1 Hz, 2H), 7.41 (d, *J* = 8.1 Hz, 2H, 2'-H, 3'-H), 7.81 (d, *J* = 9.4 Hz, 1H), 8.52 (t, *J* = 6.1 Hz, 1H, NH), 8.97 (s, 1H, 2''-H). The expected signal at 3.30 ppm for 4 × OCH_2 was unquantifiable (overlapping H_2O peak). **^{13}C NMR** (151 MHz, DMSO-d_6) δ 16.09 (CH_3), 22.42, 22.68, 25.14, 26.25 (CH_2), 26.54 (C($\underline{C}H_3)_3$), 28.98 (CH_2), 29.21 (2 × CH_2), 29.23, 32.18 (CH_2), 34.79, 35.37 (CH2, $\underline{C}(CH_3)_3$), 38.09 (C-3), 41.82 ($NHCH_2$), 45.51 (CH_2Cl), 56.46 , 56.49, 58.85 (C-2, C-5, NHCH), 69.02 (C-4), 69.80, 69.95 (OCH_2), 70.06 (2 × OCH_2), 127.60, 128.81 (C-2', C-3'), 129.82, 131.33 (C-5'', C-1'), 139.68 (C-4'), 147.89 (C-4''), 151.60 (C-2''), 169.88, 172.11, 172.16 (CO); **MS** (ESI) *m/z* $[M + H]^+$ calcd for $C_{38}H_{59}ClN_4O_6S$, 735.39; found, 735.3; **HRMS** (ESI) *m/z* $[M + H]^+$ calcd for $C_{38}H_{59}ClN_4O_6S$, 735.3917; found, 735.3887.

VHL(a)-based PROTAC 25

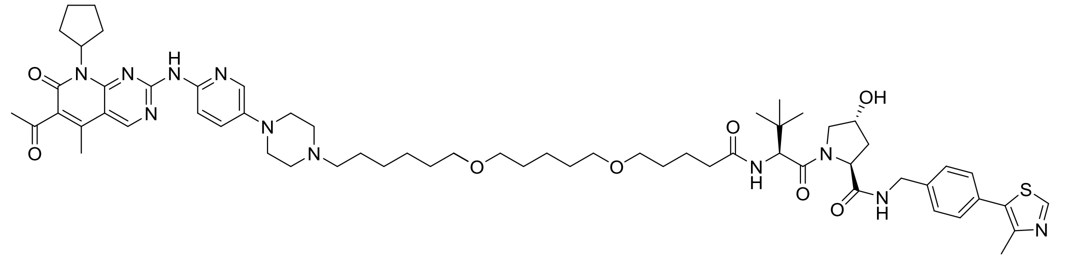

This compound was prepared using the General Procedure **IV** and precursor **61** (74 mg). The crude product was purified by column chromatography (CH_2Cl_2/MeOH+7N NH_3 19:1) followed by HPLC

purification using a gradient of 70% to 100% v/v MeOH. The title compound was obtained as a yellow solid after lyophilisation.

Yield (41 mg, 36%); mp 104 – 106 °C; R_f = 0.25 (CH_2Cl_2/MeOH+7N NH_3 19:1); **^{1}H NMR** (600 MHz, DMSO-d_6) δ 0.92 (s, 9H), 1.30 (dtd, *J* = 4.9, 7.4, 9.4 Hz, 6H), 1.37 – 1.62 (m, 16H), 1.71 – 1.80 (m, 2H), 1.82 – 1.93 (m, 3H), 1.98 – 2.06 (m, 1H), 2.06 – 2.16 (m, 1H), 2.17 – 2.33 (m, 8H), 2.41 (s, 3H), 2.43 (s, 3H), 3.13 (t, *J* = 5.0 Hz, 4H), 3.27 – 3.34 (m, 10H), 3.51 – 3.77 (m, 2H), 4.20 (dd, *J* = 5.5, 15.8 Hz, 1H), 4.29 – 4.38 (m, 1H), 4.38 – 4.46 (m, 2H), 4.53 (d, *J* = 9.4 Hz, 1H), 5.09 (d, *J* = 3.6 Hz, 1H), 5.80 (p, *J* = 8.9 Hz, 1H), 7.34 – 7.46 (m, 5H), 7.82 (dd, *J* = 9.2, 11.3 Hz, 2H), 8.03 (d, *J* = 3.0 Hz, 1H), 8.52 (t, *J* = 6.1 Hz, 1H), 8.93 (s, 1H), 8.96 (s, 1H), 10.04 (s, 1H); **^{13}C NMR** (151 MHz, DMSO-d_6) δ 13.76, 16.08, 22.43, 22.68, 25.24, 25.84, 26.41, 26.53, 26.97, 27.70, 28.98, 29.22, 29.39, 31.45, 34.79, 35.36, 38.09, 41.81, 48.49, 52.72, 53.06, 56.44, 56.49, 58.00, 58.84, 69.02, 69.80, 70.06, 106.71, 115.33, 124.76, 127.58, 128.78, 129.37, 129.80, 131.32, 135.44, 139.66, 142.24, 143.65, 144.38, 147.87, 151.57, 154.92, 158.40, 158.74, 160.92, 169.87, 172.12, 202.58; **LC-MS** (ESI) (90% H_2O to 100% MeOH in 10 min, then 100% MeOH to 20 min, DAD 220-400 nm), t_R = 12.92 min, 99% purity, *m/z* $[M + H]^+$ calcd for $C_{62}H_{87}N_{11}O_8S$, 1146.65; found, 1146.9; **HRMS** (ESI) *m/z* $[M + H]^+$ calcd for $C_{62}H_{87}N_{11}O_8S$, 1146.6533; found, 1146.6573.

(–)VHL ligand a (62)

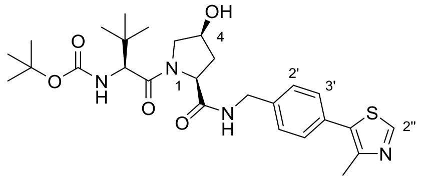

This compound was synthesized as described previously.[10]

^{1}H NMR (500 MHz, DMSO-d_6) δ 0.95 (s, 9H, $C(CH_3)_3$), 1.31 (s, 9H, $COC(CH_3)_3$), 1.92 – 2.00 (m, 1H, 3-H), 2.00 – 2.08 (m, 1H, 3-H), 2.44 (s, 3H, CH_3), 3.48 – 3.54 (m, 1H), 3.75 (dd, *J* = 5.0, 10.6 Hz, 1H), 4.10 (d, *J* = 8.2 Hz, 1H), 4.24 – 4.42 (m, 4H), 5.10 (d, *J* = 3.8 Hz, 1H, OH), 6.67 (d, *J* = 8.0 Hz, 1H, NH), 7.33 (d, *J* = 7.9 Hz, 2H, Ar-H), 7.41 (d, *J* = 8.2 Hz, 2H, Ar-H), 8.16 (t, *J* = 8.9 Hz, 1H, NH), 8.96 (s, 1H, 2"-H); **^{13}C NMR** (126 MHz, DMSO-d_6) δ 16.07 (CH_3), 26.58 ($C(\underline{C}H_3)_3$), 28.27 ($C(\underline{C}H_3)_3$), 34.81($\underline{C}(CH_3)_3$), 38.01 (C-3), 41.71 ($NHCH_2$), 55.45, 58.70, 59.04 (C-2, C-5, NHCH), 68.70 (C-4), 78.45 ($CO\underline{C}(CH_3)_3$), 127.45, 128.97 (C-2', C-3'), 129.89, 131.28 (C-5", C-1'), 139.50 (C-4'), 147.93 (C-4"), 151.55 (C-2"), 155.97 (NCO_2), 170.04 (CO), 171.88 (CO); **MS** (ESI) *m/z* $[M + H]^+$ calcd for $C_{27}H_{38}N_4O_5S$, 531.26; found, 531.4.

(–)VHL(a)-8 building block (63)

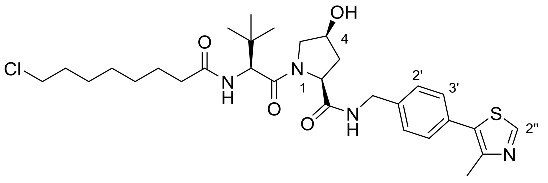

This compound was prepared using the General Procedure **III**, (–)VHL ligand a (**62**, 0.53 g) and acid **52** (0.18 g). The crude product was purified by flash chromatography on silica gel (0% to 5% MeOH in CH_2Cl_2) to yield the title compound as a colorless solid.

Yield (455 mg, 77%); mp 64 – 66 °C; R_f = 0.33 (CH_2Cl_2/MeOH 19:1); **^{1}H NMR** (600 MHz, DMSO-d_6) δ 0.96 (s, 9H, $C(CH_3)_3$), 1.09 – 1.21 (m, 4H), 1.21 – 1.43 (m, 4H), 1.56 – 1.66 (m, 2H, CH_2), 1.84 – 2.03 (m, 2H, 3-H), 2.01 – 2.11 (m, 1H), 2.12 – 2.23 (m, 1H, CH_2), 2.44 (s, 3H, CH_3), 3.51 (dd, *J* = 3.9, 10.4 Hz, 1H, 5-H'), 3.56 (t, *J* = 6.7 Hz, 2H, CH_2Cl), 3.81 (dd, *J* = 5.2, 10.5 Hz, 1H, 5-H''), 4.20 (dd, *J* = 5.6, 15.7 Hz, 1H), 4.30 – 4.50 (m, 4H, 2-H, 4-H, NHC$\underline{H}$, NHC$\underline{H}_2$), 5.11 (d, *J* = 4.0 Hz, 1H, OH), 7.31 (d, *J* = 8.0 Hz, 2H, 2'-H, 3'-H), 7.41 (d, *J* = 8.2 Hz, 2H, 2'-H, 3'-H), 7.89 (d, *J* = 7.8 Hz, 1H), 8.15 (t, *J* = 6.1 Hz, 1H, CONH), 8.97 (s, 1H, 2''-H); **^{13}C NMR** (151 MHz, DMSO-d_6) δ 16.13 (CH_3), 25.28, 26.26 (CH_2), 26.63 ($C(\underline{C}H_3)_3$), 28.06, 28.58, 32.13, 32.16, 34.33 (CH_2), 34.74 ($\underline{C}(CH_3)_3$), 38.03 (C-3), 41.71 ($NHCH_2$), 45.47 (CH_2Cl), 55.38, 57.32, 58.97 (C-2, C-5, NHCH), 68.60 (C-4), 127.45, 128.92 (C-2', C-3'), 129.86, 131.28 (C-1', C-5''), 139.51 (C-4'), 147.89 (C-4''), 151.57 (C-2''), 169.98, 171.73, 173.22 (CO); **MS** (ESI) *m/z* $[M + H]^+$ calcd for $C_{30}H_{43}ClN_4O_4S$, 591.28; found, 591.2; **HRMS** (ESI) *m/z* $[M + H]^+$ calcd for $C_{30}H_{43}ClN_4O_4S$, 591.2766; found, 591.2760.

VHL(a)-based negative control 26

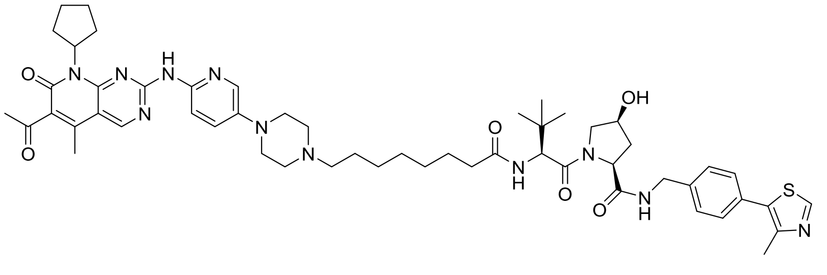

This compound was prepared using the General Procedure **IV** and precursor **63** (59 mg). The crude product was purified by column chromatography (gradient of CH_2Cl_2/MeOH+7N NH_3 29:1 to 19:1) followed by HPLC purification using a gradient of 70% to 100% v/v MeOH. The title compound was obtained as a yellow solid after lyophilisation.

Yield (61 mg, 64%); mp 126 – 130 °C; R_f = 0.18 (CH_2Cl_2/MeOH+7N NH_3 19:1); **^{1}H NMR** (600 MHz, DMSO-d_6) δ 0.97 (s, 9H), 1.09 – 1.33 (m, 7H), 1.37 (s, 5H), 1.70 – 1.80 (m, 2H), 1.81 – 1.92 (m, 2H), 1.93 – 2.27 (m, 8H), 2.30 (s, 3H), 2.41 (s, 3H), 2.41 – 2.48 (m, 7H), 2.94 – 3.27 (m, 4H), 3.51 (dd, *J* = 4.0, 10.4 Hz, 1H), 3.81 (dd, *J* = 5.2, 10.5 Hz, 1H), 4.09 – 4.27 (m, 1H), 4.27 – 4.50 (m, 4H), 5.12 (d, *J* = 4.0 Hz, 1H), 5.81 (p, *J* = 8.9 Hz, 1H), 7.23 – 7.53 (m, 5H), 7.83 (d, *J* = 9.0 Hz, 1H), 7.89 (d, *J* = 7.7 Hz, 1H), 8.02 (d, *J* = 3.0 Hz, 1H), 8.15 (t, *J* = 6.1 Hz, 1H), 8.93 (s, 1H), 8.96 (s, 1H), 10.05 (s, 1H); **^{13}C NMR** (151 MHz, DMSO-d_6) δ 13.76, 16.14, 25.24, 25.37, 26.37, 26.64, 26.97, 27.70, 28.76, 28.81, 31.44, 34.31, 34.81, 38.03, 41.73, 48.49, 52.69, 53.06, 55.37, 57.38, 58.00, 58.98, 68.60, 106.70, 115.30, 124.74, 127.45, 128.91, 129.36, 129.86, 131.29, 135.43, 139.50, 142.23, 143.64, 144.37, 147.89, 151.55, 154.92, 158.39, 158.72, 160.91, 170.01, 171.73, 173.30, 202.56; **LC-MS** (ESI) (90% H_2O to 100% MeOH in 10 min, then 100% MeOH to 20 min, DAD 220-400 nm), t_R = 12.06 min, 99% purity, *m/z* $[M + H]^+$ calcd for $C_{54}H_{71}N_{11}O_6S$, 1002.53; found, 1003.0; **HRMS** (ESI) *m/z* $[M + H]^+$ calcd for $C_{54}H_{71}N_{11}O_6S$, 1002.5382; found, 1002.5360.

VHL ligand b (64)

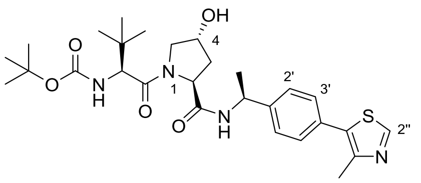

This compound was synthesized as described previously.[11,12]

^{1}H NMR (400 MHz, $CDCl_3$) δ 1.05 (s, 9H, $C(CH_3)_3$), 1.41 (s, 9H, $OC(CH_3)_3$), 1.46 (d, *J* = 7.0 Hz, 3H, $CHC\underline{H}_3$), 2.03 – 2.10 (m, 1H, 3-H), 2.53 (s, 3H, CH_3), 2.58 – 2.66 (m, 1H, 3-H), 2.77 (d, *J* = 4.0 Hz, 1H), 3.55 (dd, *J* = 3.3, 11.5 Hz, 1H), 4.15 (d, *J* = 11.5 Hz, 1H), 4.19 (d, *J* = 9.0 Hz, 1H), 4.51 (br s, 1H), 4.80 (t, *J* = 7.9 Hz, 1H), 5.07 (symm m, 1H), 5.20 (d, *J* = 8.8 Hz, 1H), 7.34 – 7.44 (m, 4H, 2’-H, 3’-H), 7.70 (d, *J* = 7.7 Hz, 1H, NHCO), 8.67 (s, 1H, 2’’-H); **^{1}H NMR** (400 MHz, DMSO-d_6) δ 0.93 (s, 9H, $C(CH_3)_3$), 1.36 (d, *J* = 7.6 Hz, 3H, $CHC\underline{H}_3$), 1.38 (s, 9H, $OC(CH_3)_3$), 1.72 – 1.81 (m, 1H, 3-H), 1.98 – 2.06 (m, 1H, 3-H), 2.45 (s, 3H, CH_3), 3.53 – 3.62 (m, 2H), 4.13 (d, *J* = 9.2 Hz, 1H), 4.28 (br s, 1H), 4.44 (t, *J* = 8.1 Hz, 1H), 4.89 (symm m, 1H), 5.12 (d, *J* = 3.3 Hz, 1H, OH), 6.42 (d, *J* = 9.0 Hz, 1H, NHBoc), 7.35 – 7.47 (m, 4H, 2’-H, 3’-H), 8.41 (d, *J* = 7.6 Hz, NHCO), 8.98 (s, 1H, 2’’-H); **^{1}H NMR** (400 MHz, CD_3OD) δ 1.02 (s, 9H, $C(CH_3)_3$), 1.44 (s, 9H, $OC(CH_3)_3$), 1.51 (d, *J* = 7.0 Hz, 3H, $CHC\underline{H}_3$), 1.90 – 2.00 (m, 1H, 3-H), 2.16 – 2.24 (m, 1H, 3-H), 2.48 (s, 3H, CH_3), 3.74 (dd, *J* = 3.8, 10.9 Hz, 1H), 3.86 (d, *J* = 11.1 Hz, 1H), 4.29 (d, *J* = 9.3 Hz, 1H), 4.44 (br s, 1H), 4.59 (t, *J* = 8.3 Hz, 1H), 4.99 (symm m, 1H), 6.41 (d, *J* = 9.4 Hz, 1H), 7.39 – 7.47 (m, 4H, 2’-H, 3’-H), 8.88 (s, 1H, 2’’-H); **^{13}C NMR** (101 MHz, $CDCl_3$) δ 16.03 (CH_3), 22.21 ($CH\underline{C}H_3$), 26.40 ($C(\underline{C}H_3)_3$), 28.29 ($OC(\underline{C}H_3)_3$), 34.97 ($\underline{C}(CH_3)_3$), 35.38 (C-3), 48.81 ($\underline{C}HCH_3$), 56.48, 58.32, 58.91 (C-2,

NHCHCO, C-5), 70.00 (C-4), 80.44 (OC(CH$_3$)$_3$), 126.40 (C-2', C-6'), 129.53 (C-3', C-5'), 130.08 (C-1'), 131.59 (C-5"), 143.18 (C-4'), 148.43 (C-4''), 150.30 (C-2''), 156.41 (NHCOO), 169.67 (NHCO), 172.65 (NCO); **HRMS** (ESI) *m/z* $[M + H]^+$ calcd for $C_{28}H_{41}N_4O_5S$, 545.2792; found, 545.2786.

VHL(b)-2-2-2-2 building block (65)

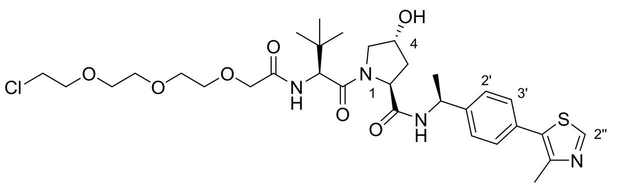

This compound was prepared using the General Procedure **III**, VHL ligand b (**64**, 0.54 g) and acid **58** (0.23 g). The crude product was purified by flash chromatography on silica gel (0% to 10% MeOH in CH_2Cl_2) to yield the title compound as a colorless solid.

Yield (392 mg, 60%); mp 58 – 60 °C; R_f = 0.34 (CH_2Cl_2/MeOH 9:1); **^{1}H NMR** (600 MHz, DMSO-d_6) δ 0.93 (s, 9H, C(CH$_3$)$_3$), 1.37 (d, *J* = 7.0 Hz, 3H, CHCH$_3$), 1.64 – 1.86 (m, 1H, 3-H'), 1.91 – 2.11 (m, 1H, 3-H''), 2.44 (s, 3H, CH$_3$), 3.48 – 3.64 (m, 10H), 3.64 – 3.76 (m, 4H), 3.95 (d, *J* = 1.3 Hz, 2H, 5-H, OCH$_2$, CH$_2$Cl), 4.24 – 4.37 (m, 1H), 4.43 (t, *J* = 8.3 Hz, 1H), 4.54 (d, *J* = 9.5 Hz, 1H), 4.90 (p, *J* = 7.3 Hz, 1H, 2-H, 4-H, 2 × NHCH), 5.10 (d, *J* = 3.6 Hz, 1H, OH), 7.24 – 7.40 (m, 3H), 7.40 – 7.52 (m, 2H, CONH, 2'-H, 3'-H), 8.40 (d, *J* = 7.8 Hz, 1H, NHCO), 8.97 (s, 1H, 2''-H); **^{13}C NMR** (151 MHz, DMSO-d_6) δ 16.12 (CH$_3$), 22.58 (CHCH$_3$), 26.37 (C(CH$_3$)$_3$), 35.89 (C(CH$_3$)$_3$), 37.86 (C-3), 40.22 (NHCH$_2$), 43.73 (CH$_2$Cl), 47.89 (CHCH$_3$),, 55.8 3, 56.65, 58.70 (C-2, NHCHCO, C-5), 68.91 (OCH$_2$), 69.77 and 69.81 (3 × OCH$_2$), 70.00 (2 × C), 70.59, 70.71 (OCH$_2$, C-4), 126.48 (C-2', C-6'), 128.97 (C-3', C-5'), 129.84 (C-1'), 131.25 (C-5"), 144.85 (C-4'), 147.90 (C-4''), 151.60 (C-2''), 168.65, 169.16, 170.59 (CO); **MS** (ESI) *m/z* $[M + H]^+$ calcd for $C_{31}H_{45}ClN_4O_7S$ 653.28; found, 653.5; **HRMS** (ESI) *m/z* $[M + H]^+$ calcd for $C_{31}H_{45}ClN_4O_7S$, 653.2770; found, 653.2759.

VHL(b)-based PROTAC 27 (CST620)

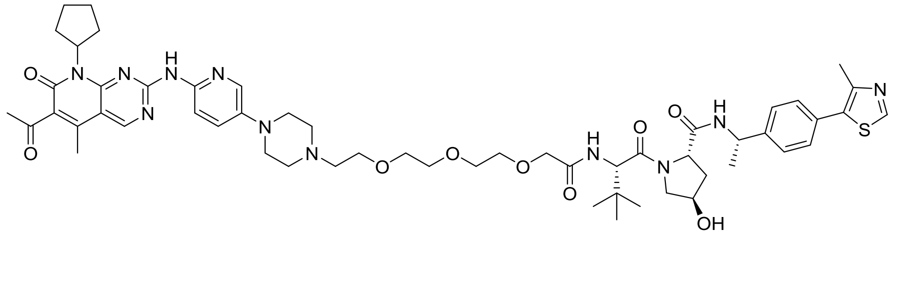

This compound was prepared using the General Procedure **IV** and precursor **65** (65 mg). The crude product was purified by column chromatography (gradient of CH_2Cl_2/MeOH+7N NH_3 29:1 to 19:1) followed by HPLC purification using a gradient of 70% to 100% v/v MeOH. The title compound was obtained as a yellow solid after lyophilisation.

Yield (61 mg, 57%); mp 116 – 120 °C; R_f = 0.13 (CH_2Cl_2/MeOH+7N NH_3 19:1); **^{1}H NMR** (600 MHz, DMSO-d_6) δ 0.94 (s, 9H), 1.36 (d, *J* = 7.0 Hz, 3H), 1.49 – 1.60 (m, 2H), 1.70 – 1.81 (m, 3H), 1.81 – 1.92 (m, 2H), 2.00 – 2.08 (m, 1H), 2.23 (q, *J* = 8.5, 9.2 Hz, 2H), 2.29 (s, 3H), 2.41 (s, 3H), 2.43 (s, 3H), 2.52 (t, *J* = 5.9 Hz, 2H), 2.57 (t, *J* = 5.0 Hz, 4H), 3.07 – 3.21 (m, 4H), 3.49 – 3.77 (m, 12H), 3.86 – 4.05 (m, 2H), 4.15 – 4.38 (m, 1H), 4.44 (t, *J* = 8.3 Hz, 1H), 4.54 (d, *J* = 9.5 Hz, 1H), 4.89 (p, *J* = 7.1 Hz, 1H), 5.11 (d, *J* = 3.6 Hz, 1H), 5.80 (p, *J* = 8.9 Hz, 1H), 7.26 – 7.57 (m, 6H), 7.83 (d, *J* = 8.9 Hz, 1H), 8.04 (d, *J* = 3.1 Hz, 1H), 8.39 (d, *J* = 7.7 Hz, 1H), 8.93 (s, 1H), 8.95 (s, 1H), 10.05 (s, 1H); **^{13}C NMR** (151 MHz, DMSO-d_6) δ 13.75, 16.11, 22.59, 25.24, 26.40, 27.69, 31.44, 35.88, 37.87, 47.89, 48.48, 53.06, 55.85, 56.66, 57.38, 58.72, 68.56, 68.93, 69.79, 69.81, 69.85, 70.02, 70.66, 106.70, 115.30, 124.75, 126.47, 128.95, 129.36, 129.84, 131.24, 135.45, 142.23, 143.63, 144.38, 144.82, 147.89, 151.56, 154.91, 158.38, 158.72, 160.91, 168.67, 169.18, 170.60, 202.56; **LC-MS** (ESI) (90% H_2O to 100% MeOH in 10 min, then 100% MeOH to 20 min, DAD 220-400 nm), t_R = 11.92 min, 99% purity, *m/z* $[M + H]^+$ calcd for $C_{55}H_{73}N_{11}O_9S$, 1064.53; found, 1064.9; **HRMS** (ESI) *m/z* $[M + H]^+$ calcd for $C_{55}H_{73}N_{11}O_9S$, 1064.5386; found, 1064.5368.

VHL ligand c (66)

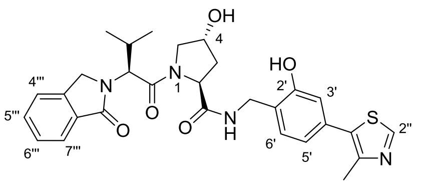

This compound was synthesized as described previously.[13]

^{1}H NMR (600 MHz, DMSO-d_6) δ 0.73 (d, *J* = 6.6 Hz, 3H), 0.97 (d, *J* = 6.6 Hz, 3H, CH(C<u>H</u>$_3$)$_2$), 1.86 – 1.94 (m, 1H), 1.97 – 2.07 (m, 1H, 3-H), 2.23 – 2.40 (m, 1H, C<u>H</u>(CH$_3$)$_2$), 2.45 (s, 3H, CH$_3$), 3.64 – 3.80 (m, 2H, 5-H), 4.16 – 4.29 (m, 2H), 4.29 – 4.35 (m, 1H), 4.36 – 4.58 (m, 3H,), 4.70 (d, *J* = 10.8 Hz, 1H, 2-H, 4-H, NCH, NHC<u>H</u>$_2$, NCH$_2$), 5.06 (d, *J* = 4.0 Hz, 1H, OH), 6.87 (dd, *J* = 1.8, 7.9 Hz, 1H, 5'-H), 6.92 (d, *J* = 1.8 Hz, 1H, 3'-H), 7.25 (d, *J* = 7.8 Hz, 1H, 6'-H), 7.49 (ddd, *J* = 2.3, 5.8, 7.8 Hz, 1H), 7.53 – 7.66 (m, 2H), 7.70 (d, *J* = 7.6 Hz, 1H, Ar'''-H), 8.37 (t, *J* = 5.9 Hz, 1H, CONH), 8.94 (s, 1H, 2''-H), 9.79 (s, 1H, Ar-OH);**^{13}C NMR** (151 MHz, DMSO-d_6) δ 16.26 (CH$_3$), 18.78, 19.06 (CH(<u>C</u>H$_3$)$_2$), 28.53 (<u>C</u>H(CH$_3$)$_2$), 37.34 (C-3), 38.27 (NHCH$_2$), 46.98 (C-2'''), 55.54 (C-5), 57.96 (C-2), 58.81 (NCH), 68.76 (C-4), 115.27 (C-3'), 119.59 (C-5'),

123.17, 123.77 (C-4''', C-7'''), 125.43 (C-1'), 128.06 (C-6'''), 128.72 (C-6'), 130.93, 131.47, 131.55 (C-4', C-5'', C-7a'''), 131.74 (C-5'''), 142.37 (C-3a'''), 147.69 (C-4''), 151.42 (C-2''), 155.04 (C-2'), 167.66, 168.25, 171.90 (CO); **MS** (ESI) *m/z* $[M + H]^+$ calcd for $C_{29}H_{32}N_4O_5S$, 549.21; found, 549.4.

8-Chlorooctyl methanesulfonate (67)

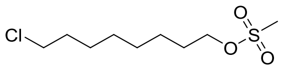

To a solution of 8-chloro-1-octanol (2.20 g, 13.12 mmol) in dry CH_2Cl_2 (20 mL), Et_3N (1.99 g, 2.74 mL, 19.68 mmol) was added under argon atmosphere and the mixture was cooled to 0 °C. Subsequently, methanesulfonyl chloride (2.25 g, 1.52 mL, 19.68 mmol) was added dropwise at 0 °C, followed by stirring of the mixture at rt for 3 h. After the reaction was complete (monitored by TLC), MeOH (20 mL) was added to the mixture carefully. The volatiles were then evaporated and the resultant residue was partitioned between EtOAc (100 mL) and H_2O (100 mL). The organic layer was further washed with brine (2 × 100 mL), dried over Na_2SO_4, filtered and evaporated. The crude product was purified by column chromatography (EtOAc/*n*-hexanes 2:1) to give a colorless oil.

Yield (2.74 g, 86%); R_f = 0.60 (EtOAc); **^{1}H NMR** (400 MHz, $CDCl_3$) δ 1.29 – 1.37 (m, 4H), 1.38 – 1.49 (m, 4H), 1.75 (symm m, 4H), 3.00 (s, 3H, CH_3), 3.53 (t, *J* = 6.7 Hz, 2H, CH_2Cl), 4.22 (t, *J* = 6.5 Hz, 2H, OCH_2); **HRMS** (ESI) *m/z* $[M + H]^+$ calcd for $C_9H_{20}O_3ClS$, 243.0816; found, 243.0815.

VHL(c)-8 building block (68)

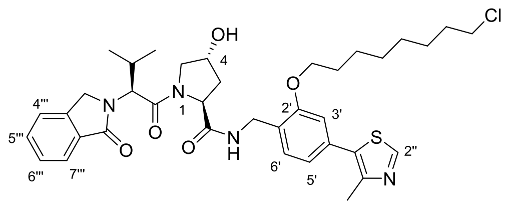

This compound was prepared using the General Procedure **V**, VHL ligand c (**66**, 165 mg) and mesylate **67** (87 mg). The crude product was purified by flash chromatography on silica gel (0% to 5% MeOH in CH_2Cl_2) to yield the title compound as a colorless solid.

Yield (142 mg, 68%); mp 80 – 84 °C; R_f = 0.22 (CH_2Cl_2/MeOH 29:1); **^{1}H NMR** (600 MHz, DMSO-d_6) δ 0.73 (d, *J* = 6.7 Hz, 3H), 0.96 (d, *J* = 6.5 Hz, 3H, $CH(C\underline{H}_3)_2$), 1.27 – 1.42 (m, 6H), 1.42 – 1.49 (m, 2H), 1.66 – 1.79 (m, 4H, CH_2), 1.88 – 1.96 (m, 1H), 1.99 – 2.07 (m, 1H, 3-H), 2.26 – 2.40 (m, 1H, $C\underline{H}(CH_3)_2$), 2.46 (s, 3H, CH_3), 3.61 (t, *J* = 6.6 Hz, 2H, CH_2Cl), 3.64 – 3.72 (m, 1H), 3.77 (dd, *J* = 4.5, 10.6 Hz, 1H, 5-H), 4.04

(t, *J* = 6.3 Hz, 2H, OCH_2), 4.18 – 4.37 (m, 3H), 4.37 – 4.59 (m, 3H), 4.71 (d, *J* = 10.8 Hz, 1H, 2-H, 4-H, NCH, NHC<u>H</u>$_2$, NCH_2), 5.06 (d, *J* = 4.1 Hz, 1H, OH), 6.96 – 7.03 (m, 2H, 3'-H, 5'-H), 7.32 (d, *J* = 7.7 Hz, 1H, 6'-H), 7.46 – 7.55 (m, 1H), 7.55 – 7.64 (m, 2H), 7.68 – 7.76 (m, 1H, Ar'''-H), 8.33 (t, *J* = 6.0 Hz, 1H, CONH), 8.97 (s, 1H, 2''-H); **^{13}C NMR** (151 MHz, DMSO-d_6) δ 16.15 (CH_3), 18.76, 19.02 (CH(<u>C</u>H_3)$_2$), 25.63, 26.37, 28.34 (CH_2), 28.52 (<u>C</u>H(CH_3)$_2$), 28.77 (2 × CH_2), 32.19 (CH_2), 37.17 (C-3), 38.22 ($NHCH_2$), 45.54 (CH_2Cl), 46.95 (C-2'''), 55.53 (C-5), 57.93 (C-2), 58.84 (NCH), 67.88 (OCH_2), 68.76 (C-4), 111.87 (C-3'), 120.90 (C-5'), 123.15, 123.75 (C-4''', C-7'''), 127.14, 127.83 (C-1', C-6'), 128.04 (C-6'''), 131.13, 131.46, 131.53 (C-4', C-5'', C-7a'''), 131.71 (C-5'''), 142.34 (C-3a'''), 148.03 (C-4''), 151.56 (C-2''), 156.11 (C-2'), 167.62, 168.26, 171.66 (CO); **MS** (ESI) *m/z* $[M + H]^+$ calcd for $C_{37}H_{47}ClN_4O_5S$, 695.30; found, 695.7; **HRMS** (ESI) *m/z* $[M + H]^+$ calcd for $C_{37}H_{47}ClN_4O_5S$, 695.3006; found, 695.3029.

VHL(c)-based PROTAC 28

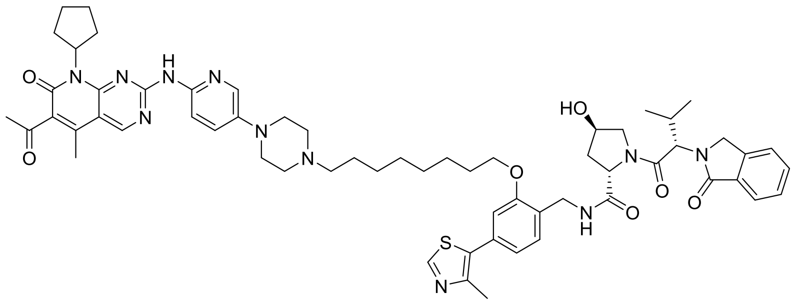

This compound was prepared using the General Procedure **IV** and precursor **68** (70 mg). The crude product was purified by column chromatography (CH_2Cl_2/MeOH+7N NH_3 29:1) followed by HPLC purification using a gradient of 70% to 100% v/v MeOH. The title compound was obtained as a yellow solid after lyophilisation.

Yield (55 mg, 50%); mp 134 – 138 °C; R_f = 0.10 (CH_2Cl_2/MeOH+7N NH_3 29:1); **^{1}H NMR** (600 MHz, DMSO-d_6) δ 0.73 (d, *J* = 6.7 Hz, 3H), 0.96 (d, *J* = 6.4 Hz, 3H), 1.25 – 1.63 (m, 12H), 1.66 – 1.98 (m, 7H), 2.00 – 2.17 (m, 1H), 2.18 – 2.37 (m, 8H), 2.41 (s, 3H), 2.43 – 2.49 (m, 7H), 3.07 – 3.26 (m, 4H), 3.64 – 3.84 (m, 2H), 4.04 (t, *J* = 6.3 Hz, 2H), 4.17 – 4.60 (m, 6H), 4.71 (d, *J* = 10.8 Hz, 1H), 5.07 (s, 1H), 5.80 (p, *J* = 8.9 Hz, 1H), 6.90 – 7.04 (m, 2H), 7.33 (d, *J* = 7.7 Hz, 1H), 7.39 – 7.53 (m, 2H), 7.55 – 7.65 (m, 2H), 7.70 (d, *J* = 7.6 Hz, 1H), 7.83 (d, *J* = 9.0 Hz, 1H), 8.03 (d, *J* = 3.1 Hz, 1H), 8.33 (t, *J* = 6.0 Hz, 1H), 8.93 (s, 1H), 8.97 (s, 1H), 10.04 (s, 1H) ; **^{13}C NMR** (151 MHz, DMSO-d_6) δ 13.75, 16.15, 18.76, 19.03, 25.24, 25.68, 26.42, 27.06, 27.69, 28.52, 28.79, 28.89, 29.07, 31.44, 37.17, 38.22, 46.95, 48.50, 52.72, 53.05, 55.54, 57.93, 58.04, 58.84, 67.88, 68.76, 106.70, 111.87, 115.30, 120.89, 123.15, 123.73, 124.73, 127.13, 127.85, 128.03, 129.35, 131.13, 131.46, 131.53, 131.70, 135.44, 142.23, 142.33, 143.64, 144.37, 148.02, 151.55, 154.91, 156.12, 158.39, 158.72, 160.91, 167.62, 168.26, 171.66, 202.56; **LC-MS** (ESI) (90% H_2O to 100% MeOH in 10 min, then 100% MeOH to 20 min, DAD 220-420 nm), t_R =

12.84 min, 99% purity, *m/z* [M + H]$^+$ calcd for $C_{61}H_{75}N_{11}O_7S$, 1106.56; found, 1106.8; **HRMS** (ESI) *m/z* [M + H]$^+$ calcd for $C_{61}H_{75}N_{11}O_7S$, 1106.5644; found, 1106.5628.

2-[2-(2-Chloroethoxy)ethoxy]ethyl methanesulfonate (69)

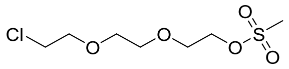

To a solution of 2-[2-(2-chloroethoxy)ethoxy]ethanol (1.18 g, 7 mmol) in dry CH_2Cl_2 (10 mL), DIPEA (1.36 g, 1.78 mL, 10.5 mmol) was added under argon atmosphere and the mixture was cooled to 0 °C. Subsequently, methanesulfonyl chloride (1.20 g, 0.81 mL, 10.5 mmol) was added dropwise at 0 °C, followed by stirring of the mixture at rt for 3 h. After the reaction was complete (monitored by TLC), MeOH (20 mL) was added to the mixture carefully. The volatiles were then evaporated and the crude product was purified by column chromatography (EtOAc) to give a colorless oil.

Yield (1.62 g, 94%); R_f = 0.50 (EtOAc); **^{1}H NMR** (400 MHz, $CDCl_3$) δ 3.07 (s, 3H, CH_3), 3.61 – 3.64 (m, 2H), 3.67 – 3.69 (m, 4H), 3.73 – 3.77 (m, 2H), 3.78 (symm m, 2H), 4.38 (symm m, 2H); **^{13}C NMR** (101 MHz, $CDCl_3$) δ 37.67 (CH_3), 42.77 (CH_2Cl), 69.03, 69.17, 70.51, 70.53, 71.28 (OCH_2); **HRMS** (ESI) *m/z* [M + H]$^+$ calcd for $C_7H_{16}O_5ClS$, 247.0402; found, 247.0399.

VHL(c)-2-2-2 building block (70)

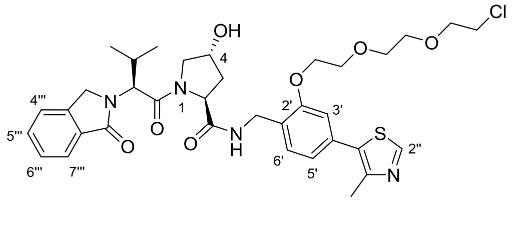

This compound was prepared using the General Procedure **V**, VHL ligand c (**66**, 165 mg) and mesylate **69** (89 mg). The crude material was purified by column chromatography (gradient of CH_2Cl_2/MeOH 29:1 to 19:1) to yield the title compound as a colorless solid.

Yield (117 mg, 56%); mp 74 – 76 °C; R_f = 0.28 (CH_2Cl_2/MeOH 15:1); **^{1}H NMR** (600 MHz, DMSO-d_6) δ 0.73 (d, *J* = 6.6 Hz, 3H), 0.96 (d, *J* = 6.5 Hz, 3H, CH(C$\underline{H}_3$)$_2$), 1.81 – 1.97 (m, 1H), 1.99 – 2.15 (m, 1H, 3-H), 2.20 – 2.41 (m, 1H, C$\underline{H}$(CH_3)$_2$), 2.46 (s, 3H, CH_3), 3.57 – 3.62 (m, 2H, CH_2Cl), 3.60 – 3.71 (m, 7H), 3.74 – 3.82 (m, 3H, 5-H, OCH_2), 4.19 (t, *J* = 4.7 Hz, 2H, OCH_2), 4.21 – 4.37 (m, 3H), 4.37 – 4.59 (m, 3H), 4.71 (d, *J* = 10.8 Hz, 1H, 2-H, 4-H, NCH, NHC$\underline{H}_2$, NCH_2), 5.06 (d, *J* = 4.1 Hz, 1H, OH), 7.00 (dd, *J* = 1.6, 7.7 Hz, 1H, 5'-H), 7.04 (d, *J* = 1.8 Hz, 1H, 3'-H), 7.33 (d, *J* = 7.8 Hz, 1H, 6'-H), 7.41 – 7.56 (m, 1H), 7.56 – 7.66

(m, 2H), 7.70 (dd, *J* = 1.1, 7.5 Hz, 1H, Ar'''-H), 8.33 (t, *J* = 6.0 Hz, 1H, CONH), 8.97 (s, 1H, 2''-H); **^{13}C NMR** (151 MHz, DMSO-d_6) δ 16.16 (CH_3), 18.77, 19.03 (CH($\underline{C}H_3)_2$), 28.54 ($\underline{C}H(CH_3)_2$), 37.23 (C-3), 38.24 ($NHCH_2$), 43.71 (CH_2Cl), 46.97 (C-2'''), 55.56 (C-5), 57.95 (C-2), 58.86 (NCH), 68.08, 68.77, 69.17, 69.88, 70.22, 70.73 (C-4, OCH_2), 112.38 (C-3'), 121.25 (C-5'), 123.16, 123.76 (C-4''', C-7'''), 127.38, 127.87 (C-1', C-6'), 128.05 (C-6'''), 131.15, 131.41, 131.54 (C-4', C-5'', C-7a'''), 131.72 (C-5'''), 142.36 (C-3a'''), 148.09 (C-4''), 151.60 (C-2''), 156.04 (C-2'), 167.64, 168.27, 171.70 (CO); **MS** (ESI) *m/z* $[M + H]^+$ calcd for $C_{35}H_{43}ClN_4O_7S$, 699.26; found, 699.7; **HRMS** (ESI) *m/z* $[M + H]^+$ calcd for $C_{35}H_{43}ClN_4O_7S$, 699.2614; found, 699.2589.

VHL(c)-based PROTAC 29

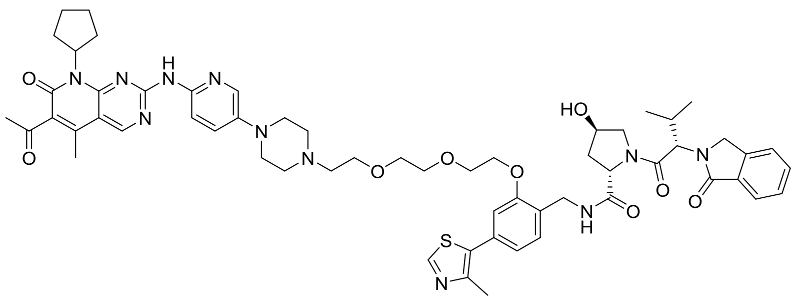

This compound was prepared using the General Procedure **IV** and precursor **70** (70 mg). The crude product was purified by column chromatography (CH_2Cl_2/MeOH+7N NH_3 29:1) followed by HPLC purification using a gradient of 70% to 100% v/v MeOH. The title compound was obtained as a yellow solid after lyophilisation.

Yield (29 mg, 26%); mp 96 – 98 °C; R_f = 0.25 (CH_2Cl_2/MeOH+7N NH_3 19:1); **^{1}H NMR** (600 MHz, DMSO-d_6) δ 0.72 (d, *J* = 6.6 Hz, 3H), 0.95 (d, *J* = 6.5 Hz, 3H), 1.52 – 1.60 (m, 2H), 1.71 – 1.80 (m, 2H), 1.80 – 1.96 (m, 3H), 2.00 – 2.07 (m, 1H), 2.17 – 2.36 (m, 6H), 2.41 (s, 3H), 2.46 (s, 3H), 2.49 – 2.61 (m, 6H), 3.11 (t, *J* = 5.0 Hz, 4H), 3.45 – 3.59 (m, 4H), 3.60 – 3.86 (m, 6H), 4.19 (t, *J* = 4.7 Hz, 2H), 4.22 – 4.60 (m, 6H), 4.71 (d, *J* = 10.8 Hz, 1H), 5.06 (d, *J* = 4.1 Hz, 1H), 5.80 (p, *J* = 8.9 Hz, 1H), 7.00 (dd, *J* = 1.6, 7.7 Hz, 1H), 7.05 (d, *J* = 1.7 Hz, 1H), 7.34 (d, *J* = 7.8 Hz, 1H), 7.41 (dd, *J* = 3.1, 9.1 Hz, 1H), 7.44 – 7.54 (m, 1H), 7.54 – 7.63 (m, 2H), 7.69 (d, *J* = 7.5 Hz, 1H), 7.82 (d, *J* = 8.9 Hz, 1H), 8.01 (d, *J* = 3.1 Hz, 1H), 8.33 (t, *J* = 6.0 Hz, 1H), 8.93 (s, 1H), 8.96 (s, 1H), 10.04 (s, 1H); **^{13}C NMR** (151 MHz, DMSO-d_6) δ 13.74, 16.16, 18.75, 19.02, 25.23, 27.68, 28.52, 31.43, 37.22, 38.23, 46.94, 48.42, 53.02, 55.54, 57.34, 57.92, 58.84, 68.14, 68.53, 68.75, 69.14, 69.90, 70.23, 106.70, 112.34, 115.28, 121.22, 123.13, 123.72, 124.73, 127.36, 127.87, 128.02, 129.35, 131.14, 131.39, 131.51, 131.69, 135.43, 142.21, 142.32, 143.59, 144.36, 148.06, 151.57, 154.90, 156.05, 158.37, 158.71, 160.90, 167.61, 168.25, 171.67, 202.56; **LC-MS** (ESI) (90% H_2O to 100% MeOH in 10 min, then 100% MeOH to 20 min, DAD 220-400 nm), t_R =

11.81 min, 99% purity, *m/z* $[M + H]^+$ calcd for $C_{59}H_{71}N_{11}O_9S$, 1110.52; found, 1111.2; **HRMS** (ESI) *m/z* $[M + H]^+$ calcd for $C_{59}H_{71}N_{11}O_9S$, 1110.5230; found, 1110.5179.

2-[2-[2-(2-Chloroethoxy)ethoxy]ethoxy]ethyl methanesulfonate (71)

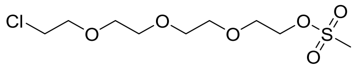

To a solution of 2-[2-[2-(2-chloroethoxy)ethoxy]ethoxy]ethanol (0.88 g, 4.14 mmol) in dry CH_2Cl_2 (10 mL), Et_3N (0.63 g, 0.86 mL, 6.21 mmol) was added under argon atmosphere and the mixture was cooled to 0 °C. Subsequently, methanesulfonyl chloride (0.71 g, 0.45 mL, 6.21 mmol) was added dropwise at 0 °C, followed by stirring of the mixture at rt for 3 h. After the reaction was complete (monitored by TLC), MeOH (20 mL) was added to the mixture carefully. The volatiles were then evaporated and the resultant residue was partitioned between EtOAc (100 mL) and H_2O (100 mL). The organic layer was further washed with brine (2 × 100 mL), dried over Na_2SO_4, filtered and evaporated. The crude product was purified by column chromatography (EtOAc/*n*-hexanes 2:1) to give a colorless oil.

Yield (1.08 g, 90%); R_f= 0.20 (EtOAc/*n*-hexanes 2:1); ^{1}**H NMR** (400 MHz, $CDCl_3$) δ 3.08 (s, 3H, CH_3), 3.61 – 3.70 (m, 10H), 3.73 – 3.79 (m, 4H), 4.38 (symm m, 2H); **HRMS** (ESI) *m/z* $[M + H]^+$ calcd for $C_9H_{20}O_6ClS$, 291.0664; found, 291.0658.

VHL(c)-2-2-2-2 building block (72)

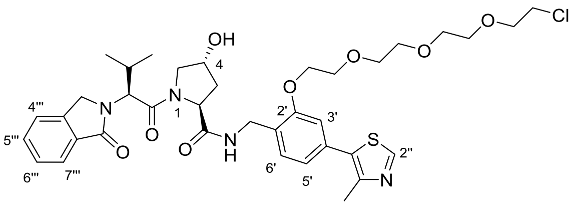

This compound was prepared using the General Procedure **V**, VHL ligand c (**66**, 165 mg) and mesylate **71** (105 mg). The crude material was purified by column chromatography (CH_2Cl_2/MeOH 29:1) to yield the title compound as a colorless solid.

Yield (120 mg, 54%); mp 56 – 58 °C; R_f = 0.39 (CH_2Cl_2/MeOH 15:1); ^{1}**H NMR** (600 MHz, DMSO-d_6) δ 0.73 (d, *J* = 6.6 Hz, 3H), 0.96 (d, *J* = 6.5 Hz, 3H, CH(C<u>H</u>$_3$)$_2$), 1.87 – 1.96 (m, 1H), 1.99 – 2.07 (m, 1H, 3-H), 2.26 – 2.37 (m, 1H, C<u>H</u>(CH_3)$_2$), 2.46 (s, 3H, CH_3), 3.46 – 3.59 (m, 6H), 3.59 – 3.71 (m, 7H), 3.73 – 3.83 (m, 3H, 5-H, CH_2Cl, OCH_2), 4.15 – 4.20 (m, 2H, OCH_2), 4.21 – 4.36 (m, 3H), 4.36 – 4.58 (m, 3H), 4.71 (d,

J = 10.8 Hz, 1H, 2-H, 4-H, NCH, NHC<u>H</u>$_2$, NCH$_2$), 5.06 (d, J = 4.1 Hz, 1H, OH), 7.00 (dd, J = 1.6, 7.7 Hz, 1H, 5′-H), 7.04 (d, J = 1.8 Hz, 1H, 3′-H), 7.33 (d, J = 7.8 Hz, 1H, 6′-H), 7.44 – 7.54 (m, 1H), 7.55 – 7.66 (m, 2H), 7.68 – 7.73 (m, 1H, Ar′′′-H), 8.33 (t, J = 6.0 Hz, 1H, CONH), 8.97 (s, 1H, 2′′-H); **^{13}C NMR** (151 MHz, DMSO-d_6) δ 16.15 (CH$_3$), 18.76, 19.02 (CH(<u>C</u>H$_3$)$_2$), 28.53 (<u>C</u>H(CH$_3$)$_2$), 37.21 (C-3), 38.23 (NHCH$_2$), 43.68 (CH$_2$Cl), 46.96 (C-2′′′), 55.54 (C-5), 57.93 (C-2), 58.85 (NCH), 68.08, 68.76, 69.14, 69.82, 69.94, 70.00, 70.22, 70.67 (C-4, OCH$_2$), 112.35 (C-3′), 121.23 (C-5′), 123.15, 123.75 (C-4′′′, C-7′′′), 127.36, 127.86 (C-1′, C-6′), 128.04 (C-6′′′), 131.13, 131.40, 131.53 (C-4′, C-5′′, C-7a′′′), 131.72 (C-5′′′), 142.34 (C-3a′′′), 148.08 (C-4′′), 151.59 (C-2′′), 156.03 (C-2′), 167.63, 168.26, 171.69 (CO); **MS** (ESI) *m/z* [M + H]$^+$ calcd for $C_{37}H_{47}ClN_4O_8S$, 743.28; found, 743.5; **HRMS** (ESI) *m/z* [M + H]$^+$ calcd for $C_{37}H_{47}ClN_4O_8S$, 743.2844; found, 743.2876.

VHL(c)-based PROTAC 30

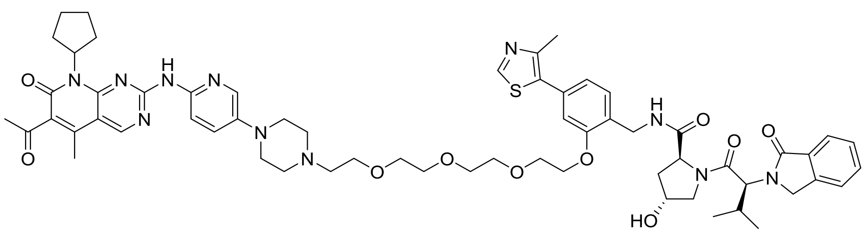

This compound was prepared using the General Procedure **IV** and precursor **72** (74 mg). The crude product was purified by column chromatography (CH_2Cl_2/MeOH+7N NH_3 19:1) to obtain the title compound as a yellow solid.

Yield (80 mg, 69%); mp 118 – 120 °C; R_f = 0.25 (CH_2Cl_2/MeOH+7N NH_3 19:1); **^{1}H NMR** (600 MHz, DMSO-d_6) δ 0.72 (d, J = 6.6 Hz, 3H), 0.96 (d, J = 6.5 Hz, 3H), 1.51 – 1.61 (m, 2H), 1.70 – 1.80 (m, 2H), 1.81 – 1.96 (m, 3H), 2.00 – 2.07 (m, 1H), 2.16 – 2.36 (m, 6H), 2.41 (s, 3H), 2.46 (s, 3H), 2.49 – 2.59 (m, 6H), 3.11 (t, J = 5.0 Hz, 4H), 3.44 – 3.60 (m, 8H), 3.60 – 3.86 (m, 6H), 4.14 – 4.21 (m, 2H), 4.21 – 4.60 (m, 6H), 4.71 (d, J = 10.8 Hz, 1H), 5.07 (d, J = 4.1 Hz, 1H), 5.74 – 5.85 (m, 1H), 7.00 (dd, J = 1.7, 7.8 Hz, 1H), 7.04 (d, J = 1.7 Hz, 1H), 7.33 (d, J = 7.8 Hz, 1H), 7.42 (dd, J = 3.2, 9.1 Hz, 1H), 7.48 (ddd, J = 2.2, 6.2, 8.1 Hz, 1H), 7.55 – 7.64 (m, 2H), 7.70 (d, J = 7.6 Hz, 1H), 7.78 – 7.86 (m, 1H), 8.02 (d, J = 3.0 Hz, 1H), 8.33 (t, J = 5.9 Hz, 1H), 8.93 (s, 1H), 8.96 (s, 1H), 10.04 (s, 1H); **^{13}C NMR** (151 MHz, DMSO-d_6) δ 13.75, 16.16, 18.76, 19.03, 25.23, 27.69, 28.53, 31.44, 37.23, 38.23, 46.95, 48.46, 53.03, 55.55, 57.36, 57.93, 58.85, 68.10, 68.51, 68.77, 69.16, 69.84, 69.97, 70.02, 70.27, 106.70, 112.35, 115.28, 121.23, 123.14, 123.73, 124.73, 127.37, 127.88, 128.03, 129.36, 131.14, 131.39, 131.53, 131.70, 135.43, 142.23, 142.33, 143.61, 144.37, 148.07, 151.55, 154.91, 156.04, 158.38, 158.71, 160.91, 167.62, 168.26, 171.68, 202.56; **LC-MS** (ESI) (90% H_2O to 100% MeOH in 10 min, then 100% MeOH to 20 min,

DAD 220-400 nm), t_R = 12.13 min, 97% purity, *m/z* $[M + H]^+$ calcd for $C_{61}H_{75}N_{11}O_{10}S$, 1154.55; found, 1154.7; **HRMS** (ESI) *m/z* $[M + H]^+$ calcd for $C_{61}H_{75}N_{11}O_{10}S$, 1154.5492; found, 1154.5452.

Linker Cl-6-5-5-OMs:

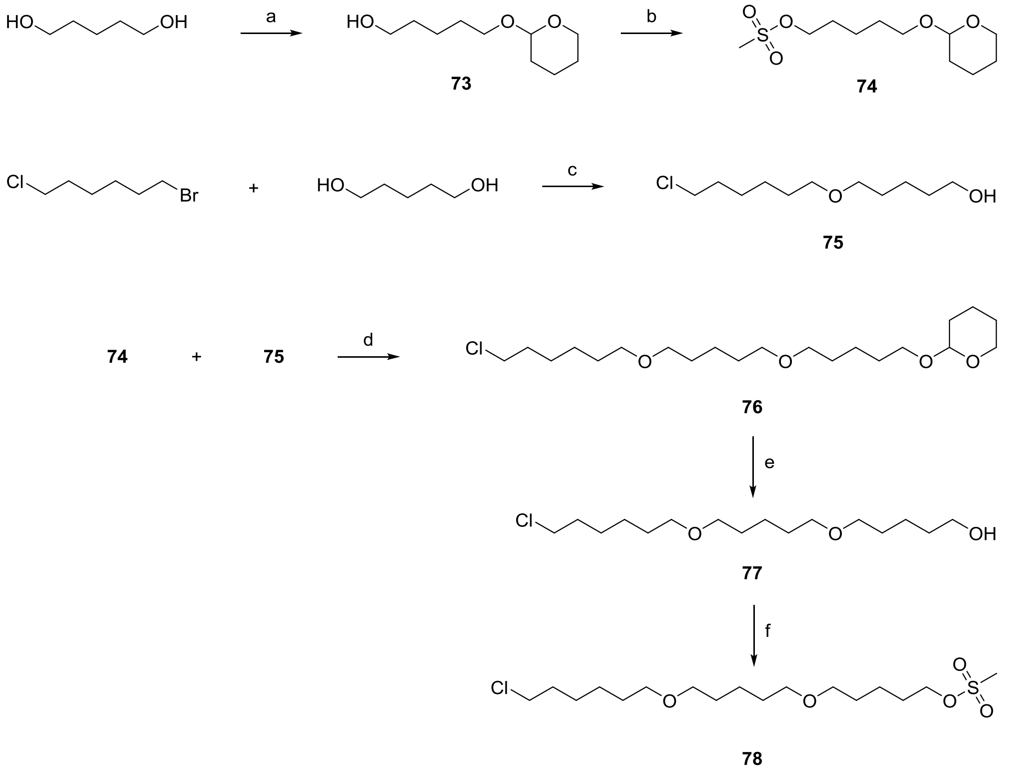

Reagents and conditions: (a) 3,4-Dihydro-2*H*-pyran, $CuSO_4·5H_2O$, MeCN, rt, 4 h; (b) MsCl, DIPEA, CH_2Cl_2, rt, 3 h; (c) 50% NaOH (aq), DMSO, rt, 20 h; (d) Bu_4NHSO_4, 50% NaOH (aq), toluene, rt, 24 h; (e) *p*-TsOH·H_2O, MeOH, rt, 20 h; (f) MsCl, DIPEA, CH_2Cl_2, rt, 3 h

5-Tetrahydropyran-2-yloxypentan-1-ol (73)

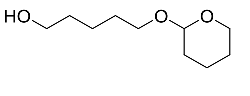

To a solution of 1,5-pentanediol (5.00 g, 47.85 mmol) in dry MeCN (25 mL), 3,4-dihydro-2*H*-pyran (4.43 g, 4.81 mL, 52.64 mmol) was added under argon atmosphere. Subsequently, $CuSO_4$·× $5H_2O$ (2.39 g, 9.57 mmol) was added, followed by stirring of the mixture at rt for 4 h. After the reaction was complete, the mixture was filtered and the filtrate was concentrated. The crude product was purified by column chromatography (EtOAc/*n*-hexanes 1:1) to give a colorless oil.

Yield (4.41 g, 49%); R_f = 0.20 (EtOAc/*n*-hexanes 1:1); ^{1}**H NMR** (400 MHz, $CDCl_3$) δ 1.39 – 1.75 (m, 12H), 1.76 – 1.87 (m, 1H), 3.39 (symm m, 1H), 3.45 – 3.53 (m, 1H), 3.64 (t, *J* = 6.6 Hz, 2H), 3.74 (symm m, 1H), 3.82 – 3.90 (m, 1H), 4.54 – 4.59 (m, 1H, CH); **HRMS** (ESI) *m/z* $[M + H]^+$ calcd for $C_{10}H_{21}O_3$, 189.1485; found, 189.1482.

5-Tetrahydropyran-2-yloxypentyl methanesulfonate (74)

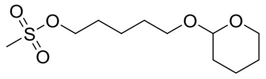

To a solution of **73** (4.94 g, 26.21 mmol) in dry CH_2Cl_2 (25 mL), DIPEA (10.16 g, 13.44 mL, 78.63 mmol) was added under argon atmosphere and the mixture was cooled to 0 °C. Subsequently, methanesulfonyl chloride (4.50 g, 3.04 mL, 39.32 mmol) was added dropwise at 0 °C, followed by stirring of the mixture at rt for 3 h. After the reaction was complete (monitored by TLC), MeOH (20 mL) was added to the mixture carefully. The volatiles were then evaporated and the crude product was purified by column chromatography (EtOAc/*n*-hexanes 1:2) to give an orange oil.

Yield (4.159 g, 60%); R_f = 0.24 (EtOAc/*n*-hexanes 1:2); ^{1}**H NMR** (400 MHz, $CDCl_3$) δ 1.47 – 1.58 (m, 6H), 1.60 – 1.72 (m, 3H), 1.75 – 1.85 (m, 3H), 3.00 (s, 3H, CH_3), 3.39 (symm m, 1H), 3.46 – 3.53 (m, 1H), 3.75 (symm m, 1H), 3.81 – 3.89 (m, 1H), 4.23 (t, *J* = 6.5 Hz, 2H), 4.54 – 4.58 (m, 1H, CH); **HRMS** (ESI) *m/z* $[M + H]^+$ calcd for $C_{11}H_{23}O_5S$, 267.1261; found, 267.1262.

5-(6-Chlorohexoxy)pentan-1-ol (75)

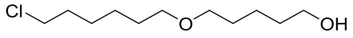

This compound was synthesized as we described previously.[8]

2-[5-[5-(6-Chlorohexoxy)pentoxy]pentoxy]tetrahydropyran (76)

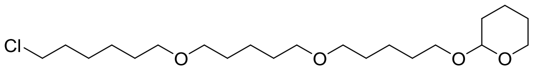

To a cooled (0 °C) solution of **75** (1.92 g, 8.637 mmol) and **74** (2.30 g, 8.637 mmol) in toluene (40 mL), Bu_4NHSO_4 (1.354 g, 9.8 mmol) and 50% NaOH (aq) (4.5 mL) were added. The reaction mixture was stirred at rt for 24 h. H_2O (70 mL) was then added and the mixture extracted with EtOAc (3 × 100 mL). The combined organic layers were further washed with H_2O and brine (each 200 mL). The organic

layers were dried over Na_2SO_4, filtered and concentrated *in vacuo*. The crude product was purified by column chromatography (EtOAc/*n*-hexanes 1:2) to give a colorless oil.

Yield (1.81 g, 53%); R_f = 0.48 (EtOAc/*n*-hexanes 1:2); **^{1}H NMR** (400 MHz, $CDCl_3$) δ 1.34 – 1.48 (m, 8H), 1.49 – 1.64 (m, 14H), 1.69 – 1.82 (m, 4H), 3.32 – 3.43 (m, 8H), 3.45 – 3.56 (m, 4H), 3.69 – 3.77 (m, 1H), 3.81 – 3.88 (m, 1H), 4.53 – 4.58 (m, 1H, CH); **^{13}C NMR** (101 MHz, $CDCl_3$) δ 19.62, 22.44, 22.50, 25.43, 25.48, 26.68, 28.48 (2 × C), 29.30 (2 × C), 29.53, 30.69, 32.51, 45.03 (CH_2Cl), 62.30, 63.91, 67.22, 67.83, 70.55, 70.70 (OCH_2), 98.82 (CH); **HRMS** (ESI) *m/z* $[M + H]^+$ calcd for $C_{21}H_{42}O_4Cl$, 393.2966; found, 393.2961.

5-[5-(6-Chlorohexoxy)pentoxy]pentan-1-ol (77)

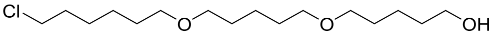

To a solution of **76** (1.80 g, 4.58 mmol) in MeOH (20 mL), *p*-TsOH ×·H_2O (0.44 g, 2.29 mmol) was added. The reaction mixture was stirred at rt for 20 h. H_2O (70 mL) was added and it was extracted with CH_2Cl_2 (2 × 150 mL). The combined organic layers were washed with brine (200 mL). The organic layers were dried over Na_2SO_4, filtered and concentrated *in vacuo*. The crude product was purified by column chromatography (EtOAc/*n*-hexanes 1:2) to give a colorless oil.

Yield (1.045 g, 74%); R_f = 0.14 (EtOAc/*n*-hexanes 1:2); **^{1}H NMR** (400 MHz, $CDCl_3$) δ 1.34 – 1.48 (m, 8H), 1.55 – 1.62 (m, 8H), 1.68 – 1.82 (m, 4H), 3.38 (td, *J* = 2.2, 6.5 Hz, 8H), 3.52 (t, *J* = 6.7 Hz, 2H), 3.63 (t, *J* = 6.4 Hz, 2H); **^{13}C NMR** (101 MHz, $CDCl_3$) δ 21.97, 22.43, 25.46, 26.66, 28.41, 28.46, 29.28, 29.50, 32.19, 32.49, 45.03 (CH_2Cl), 62.55 (CH_2OH), 67.74, 67.86, 70.54, 70.69 (OCH_2); **HRMS** (ESI) *m/z* $[M + H]^+$ calcd for $C_{16}H_{34}O_3Cl$, 309.2191; found, 309.2186.

5-[5-(6-Chlorohexoxy)pentoxy]pentyl methanesulfonate (78)

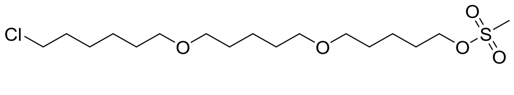

To a solution of **77** (1.05 g, 3.38 mmol) in dry CH_2Cl_2 (10 mL), DIPEA (1.31 g, 1.73 mL, 10.14 mmol) was added under argon atmosphere and the mixture was cooled to 0 °C. Subsequently, methanesulfonyl chloride (0.58 g, 0.39 mL, 5.07 mmol) was added dropwise at 0 °C, followed by stirring of the mixture at rt for 3 h. After the reaction was complete (monitored by TLC), MeOH (20 mL) was added to the mixture carefully. The volatiles were then evaporated and the crude product was purified by column chromatography (EtOAc/*n*-hexanes 1:2) to give a colorless oil.

Yield (0.750 g, 57%); R_f = 0.26 (EtOAc/*n*-hexanes 1:2); **^{1}H NMR** (400 MHz, $CDCl_3$) δ 1.32 – 1.48 (m, 8H), 1.52 – 1.62 (m, 8H), 1.72 – 1.80 (m, 4H), 2.98 (s, 3H, CH_3), 3.34 – 3.42 (m, 8H), 3.51 (t, *J* = 6.8 Hz, 2H, CH_2Cl), 4.21 (t, *J* = 6.5 Hz, 2H, OCH_2); **^{13}C NMR** (101 MHz, $CDCl_3$) δ 22.21, 22.77, 25.46, 26.65, 28.90, 29.08, 29.51 (3 × C), 32.50, 37.28 (CH_3), 45.01 (CH_2Cl), 69.95, 70.32, 70.63, 70.73, 70.82; **HRMS** (ESI) *m/z* $[M + H]^+$ calcd for $C_{17}H_{36}O_5ClS$, 387.1967; found, 387.1960.

VHL(c)-5-5-6 building block (79)

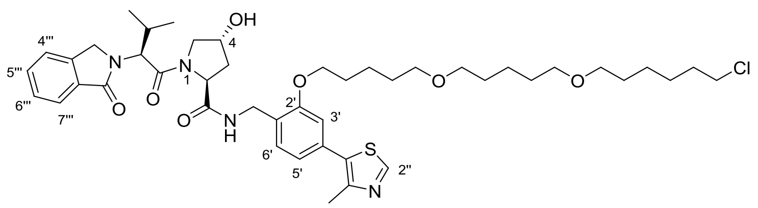

This compound was prepared using the General Procedure **V**, VHL ligand c (**66**, 165 mg) and mesylate **78** (139 mg). The crude product was purified by flash chromatography on silica gel (0% to 5% MeOH in CH_2Cl_2) to yield the title compound as a colorless resin.

Yield (123 mg, 49%); R_f = 0.35 (CH_2Cl_2/MeOH 19:1); **^{1}H NMR** (600 MHz, DMSO-*d_6*) δ 0.73 (d, *J* = 6.7 Hz, 3H), 0.95 (d, *J* = 6.5 Hz, 3H, CH(C<u>H</u>$_3$)$_2$), 1.24 – 1.40 (m, 6H), 1.41 – 1.52 (m, 8H), 1.52 – 1.64 (m, 2H), 1.65 – 1.72 (m, 2H), 1.73 – 1.83 (m, 2H, CH_2), 1.86 – 1.97 (m, 1H), 1.99 – 2.07 (m, 1H, 3-H), 2.27 – 2.39 (m, 1H, C<u>H</u>(CH$_3$)$_2$), 2.46 (s, 3H, CH_3), 3.31 – 3.39 (m, 8H), 3.59 (t, *J* = 6.6 Hz, 2H), 3.62 – 3.73 (m, 1H), 3.73 – 3.82 (m, 1H), 4.04 (t, *J* = 6.3 Hz, 2H, 5-H, OCH_2, CH_2Cl), 4.18 – 4.59 (m, 6H), 4.71 (d, *J* = 10.8 Hz, 1H, 2-H, 4-H, NCH, NHC<u>H</u>$_2$, NCH_2), 5.06 (br s, 1H, OH), 6.96 – 7.02 (m, 2H, 3'-H, 5'-H), 7.32 (d, *J* = 7.7 Hz, 1H, 6'-H), 7.45 – 7.55 (m, 1H), 7.57 – 7.64 (m, 2H), 7.70 (d, *J* = 7.6 Hz, 1H, Ar'''-H), 8.33 (t, *J* = 5.9 Hz, 1H, CONH), 8.97 (s, 1H, 2''-H); **^{13}C NMR** (151 MHz, DMSO-*d_6*) δ 16.15 (CH_3), 18.76, 19.02 (CH(<u>C</u>H$_3$)$_2$), 22.55, 22.68, 25.13, 26.24 (CH_2), 28.53 (<u>C</u>H(CH$_3$)$_2$), 28.64, 29.10, 29.18, 29.19, 29.22, 32.17 (CH_2), 37.17 (C-3), 38.21 ($NHCH_2$), 45.48 (CH_2Cl), 46.96 (C-2'''), 55.53 (C-5), 57.94 (C-2), 58.84 (NCH), 67.86, 68.77, 69.92, 70.01, 70.04, 70.08 (C-4, OCH_2), 111.86 (C-3'), 120.90 (C-5'), 123.16, 123.75 (C-4''', C-7'''), 127.14, 127.84 (C-1', C-6'), 128.04 (C-6'''), 131.13, 131.46, 131.53 (C-4', C-5'', C-7a'''), 131.72 (C-5'''), 142.35 (C-3a'''), 148.03 (C-4''), 151.56 (C-2''), 156.10 (C-2'), 167.63, 168.26, 171.66 (CO); **MS** (ESI) *m/z* $[M + H]^+$ calcd for $C_{45}H_{63}ClN_4O_7S$, 839.42; found, 839.8; **HRMS** (ESI) *m/z* $[M + H]^+$ calcd for $C_{45}H_{63}ClN_4O_7S$, 839.4179; found, 839.4160.

VHL(c)-based PROTAC 31

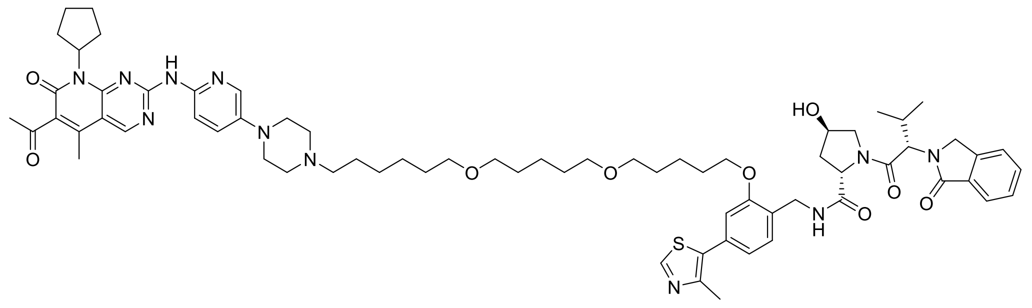

This compound was prepared using the General Procedure **IV** and precursor **79** (84 mg). The crude product was purified by column chromatography (CH_2Cl_2/MeOH+7N NH_3 29:1) followed by HPLC purification using a gradient of 80% to 100% v/v MeOH. The title compound was obtained as a yellow solid after lyophilisation.

Yield (64 mg, 51%); mp 104 – 108 °C; R_f = 0.17 (CH_2Cl_2/MeOH+7N NH_3 19:1); **^{1}H NMR** (600 MHz, DMSO-d_6) δ 0.72 (d, *J* = 6.7 Hz, 3H), 0.95 (d, *J* = 6.4 Hz, 3H), 1.23 – 1.38 (m, 6H), 1.38 – 1.51 (m, 10H), 1.52 – 1.60 (m, 4H), 1.61 – 1.80 (m, 4H), 1.80 – 1.97 (m, 3H), 1.98 – 2.08 (m, 1H), 2.20 – 2.33 (m, 7H), 2.41 (s, 3H), 2.45 (s, 3H), 2.84 – 3.22 (m, 4H), 3.29 – 3.36 (m, 10H), 3.67 (d, *J* = 10.6 Hz, 2H), 3.76 (dd, *J* = 4.5, 10.6 Hz, 2H), 4.03 (t, *J* = 6.4 Hz, 2H), 4.18 – 4.57 (m, 6H), 4.70 (d, *J* = 10.8 Hz, 1H), 5.06 (d, *J* = 4.0 Hz, 1H), 5.80 (p, *J* = 8.9 Hz, 1H), 6.94 – 7.01 (m, 2H), 7.32 (d, *J* = 7.7 Hz, 1H), 7.40 – 7.45 (m, 1H), 7.46 – 7.52 (m, 1H), 7.57 – 7.62 (m, 2H), 7.70 (d, *J* = 7.5 Hz, 1H), 7.83 (d, *J* = 9.0 Hz, 1H), 8.02 (d, *J* = 2.9 Hz, 1H), 8.33 (t, *J* = 6.0 Hz, 1H), 8.93 (s, 1H), 8.93 (d, *J* = 1.6 Hz, 1H), 8.96 (s, 1H), 10.03 (s, 1H); **^{13}C NMR** (151 MHz, DMSO-d_6) δ 13.76, 16.15, 18.76, 19.03, 22.56, 22.69, 25.25, 25.83, 26.37, 26.94, 27.70, 28.53, 28.65, 29.11, 29.20, 29.38, 31.44, 37.18, 38.22, 46.96, 48.46, 52.69, 53.07, 55.54, 57.95, 58.85, 67.86, 68.78, 70.01, 70.05, 70.09, 106.72, 111.85, 115.31, 120.91, 123.17, 123.74, 124.77, 127.15, 127.85, 128.05, 129.37, 131.13, 131.47, 131.54, 131.73, 135.45, 142.24, 142.34, 143.63, 144.41, 148.03, 151.55, 154.92, 156.11, 158.39, 158.73, 160.92, 167.65, 168.28, 171.68, 202.59; **LC-MS** (ESI) (90% H_2O to 100% MeOH in 10 min, then 100% MeOH to 20 min, DAD 220-400 nm), t_R = 13.24 min, 99% purity, *m/z* $[M + H]^+$ calcd for $C_{69}H_{91}N_{11}O_9S$, 1250.68; found, 1251.0; **HRMS** (ESI) *m/z* $[M + H]^+$ calcd for $C_{69}H_{91}N_{11}O_9S$, 1250.6795; found, 1250.6786.

(–)VHL ligand c (80)

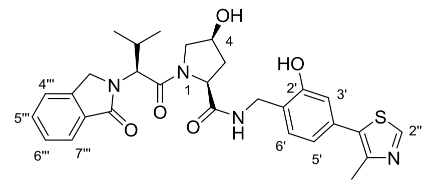

This compound was synthesized by analogy with compound **66**, but using Boc-D-Hyp-OH. The crude product was purified by column chromatography (gradient of CH_2Cl_2/MeOH 29:1 to 19:1) yielding **80** as a colorless solid.

Yield (71%); mp 106 – 108 °C; R_f = 0.30 (CH_2Cl_2/MeOH 9:1); ^{1}**H NMR** (600 MHz, DMSO-d_6) δ 0.70 (d, J = 6.6 Hz, 3H), 0.97 (d, J = 6.4 Hz, 3H, CH(C<u>H</u>$_3$)$_2$), 1.78 (ddd, J = 4.7, 7.8, 12.7 Hz, 1H), 2.04 (ddd, J = 3.0, 8.3,11.2 Hz, 1H, 3-H), 2.29 – 2.40 (m, 1H, C<u>H</u>(CH$_3$)$_2$), 2.44 (s, 3H, CH$_3$), 3.53 – 3.65 (m, 2H, 5-H), 4.13 (dd, J = 5.7, 15.9 Hz, 1H), 4.19 – 4.27 (m, 2H), 4.28 – 4.35 (m, 1H), 4.43 – 4.53 (m, 2H), 4.67 (d, J = 10.5 Hz, 1H, 2-H, 4-H, NCH, NHC<u>H</u>$_2$, NCH$_2$), 5.09 (d, J = 3.4 Hz, 1H, OH), 6.84 (dd, J = 1.8 Hz, 7.8 Hz, 1H, 5′-H), 6.90 (d, J = 1.8 Hz, 1H, 3′-H), 7.19 (d, J = 7.8 Hz, 1H, 6′-H), 7.42 – 7.52 (m, 1H), 7.52 – 7.62 (m, 2H), 7.70 (d, J = 7.7 Hz, 1H, Ar′′′-H), 8.42 (t, J = 4.7, 5.7 Hz, 1H, CONH), 8.95 (s, 1H, 2′′-H), 9.77 (s, 1H, Ar-OH); ^{13}**C NMR** (151 MHz, DMSO-d_6) δ 16.25 (CH$_3$), 18.37, 19.36 (CH(<u>C</u>H$_3$)$_2$), 27.56 (<u>C</u>H(CH$_3$)$_2$), 37.24 (C-3), 37.91 (NHCH$_2$), 46.47 (C-2′′′), 55.41 (C-5), 57.92 (C-2), 58.59 (NCH), 68.87 (C-4), 115.21 (C-3′), 119.55 (C-5′), 123.26, 123.66 (C-4′′′, C-7′′′), 125.30 (C-1′), 127.99 (C-6′′′), 128.54 (C-6′), 130.88, 131.35, 131.42 (C-4′, C-5′′, C-7a′′′), 131.71 (C-5′′′), 142.30 (C-3a′′′), 147.67 (C-4′′), 151.40 (C-2″), 154.96 (C-2′), 167.44, 167.56, 172.17 (CO); **MS** (ESI) m/z [M + H]$^+$ calcd for $C_{29}H_{32}N_4O_5S$, 549.21; found, 549.3; **HRMS** (ESI) m/z [M + H]$^+$ calcd for $C_{29}H_{32}N_4O_5S$, 549.2166; found, 549.2155.

(–)VHL(c)-2-2-2 building block (81)

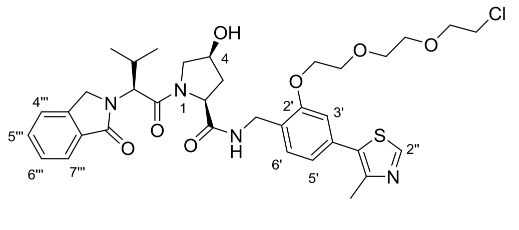

This compound was prepared using the General Procedure **V**, (–)VHL ligand c (**80**, 165 mg) and mesylate **69** (89 mg). The crude product was purified by flash chromatography on silica gel (0% to 5% MeOH in CH_2Cl_2) to yield the title compound as a colorless solid.

Yield (111 mg, 53%); mp 72 – 76 °C; R_f = 0.33 (CH_2Cl_2/MeOH 9:1); ^{1}**H NMR** (600 MHz, DMSO-d_6) δ 0.71 (d, J = 6.7 Hz, 3H), 0.98 (d, J = 6.5 Hz, 3H, CH(C<u>H</u>$_3$)$_2$), 1.67 – 1.86 (m, 1H), 2.01 – 2.12 (m, 1H, 3-H), 2.26

– 2.43 (m, 1H, $C\underline{H}(CH_3)_2$), 2.45 (s, 3H, CH_3), 3.25 – 3.71 (m, 10H), 3.78 (t, *J* = 4.7 Hz, 2H, CH_2Cl, OCH_2), 4.12 – 4.29 (m, 5H), 4.32 (d, *J* = 17.8 Hz, 1H), 4.44 – 4.54 (m, 2H), 4.68 (d, *J* = 10.5 Hz, 1H, OCH_2, 2-H, 4-H, NCH, $NHC\underline{H}_2$, NCH_2), 5.09 (d, *J* = 3.5 Hz, 1H, OH), 6.94 – 7.00 (m, 1H, 5’-H), 7.02 (d, *J* = 1.6 Hz, 1H, 3’-H), 7.27 (d, *J* = 7.8 Hz, 1H, 6’-H), 7.44 – 7.58 (m, 3H), 7.70 (d, *J* = 7.5 Hz, 1H, Ar’’’-H), 8.37 (t, *J* = 5.9 Hz, 1H, CONH), 8.97 (s, 1H, 2‘’-H); **^{13}C NMR** (151 MHz, DMSO-d_6) δ 16.17 (CH_3), 18.39, 20.04 ($CH(\underline{C}H_3)_2$), 27.59 ($\underline{C}H(CH_3)_2$), 37.20 (C-3), 37.90 ($NHCH_2$), 43.70 (CH_2Cl), 46.47 (C-2‘’’), 55.44 (C-5), 57.93 (C-2), 58.66 (NCH), 68.06, 68.89, 69.15, 69.87, 70.19, 70.71 (C-4, OCH_2), 112.37 (C-3’), 121.26 (C-5’), 123.27, 123.62 (C-4’’’, C-7’’’), 127.26, 127.76 (C-1’, C-6’), 128.00 (C-6’’’), 131.17, 131.35, 131.38 (C-4’, C-5’’, C-7a’’’), 131.70 (C-5‘’’), 142.27 (C-3a‘’’), 148.10 (C-4‘’), 151.61 (C-2‘’), 155.99 (C-2‘), 167.46, 167.63, 172.06 (CO); **MS** (ESI) *m/z* $[M + H]^+$ calcd for $C_{35}H_{43}ClN_4O_7S$, 699.26; found, 699.8; **HRMS** (ESI) *m/z* $[M + H]^+$ calcd for $C_{35}H_{43}ClN_4O_7S$, 699.2614; found, 699.2610.

VHL(c)-based negative control 32

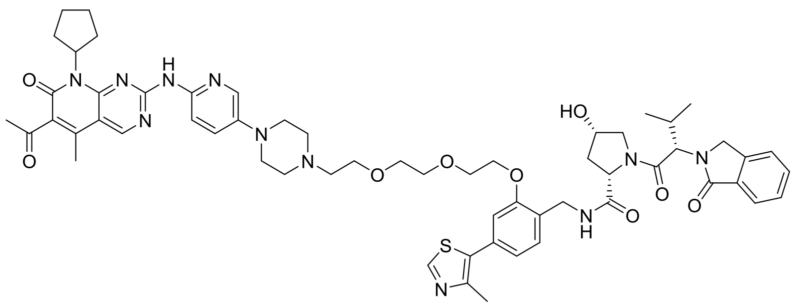

This compound was prepared using the General Procedure **IV** and precursor **81** (70 mg). The crude product was purified by column chromatography (CH_2Cl_2/MeOH+7N NH_3 19:1) followed by HPLC purification using a gradient of 80% to 100% v/v MeOH. The title compound was obtained as a yellow solid after lyophilisation.

Yield (69 mg, 62%); mp 106 – 110 °C; R_f = 0.13 (CH_2Cl_2/MeOH+7N NH_3 19:1); **^{1}H NMR** (600 MHz, DMSO-d_6) δ 0.70 (d, *J* = 6.7 Hz, 3H), 0.97 (d, *J* = 6.4 Hz, 3H), 1.50 – 1.65 (m, 2H), 1.68 – 1.83 (m, 3H), 1.83 – 1.89 (m, 2H), 2.03 – 2.09 (m, 1H), 2.19 – 2.26 (m, 2H), 2.30 (s, 3H), 2.32 – 2.39 (m, 1H), 2.41 (s, 3H), 2.45 (s, 3H), 2.49 – 2.59 (m, 6H), 3.04 – 3.12 (m, 4H), 3.38 – 3.67 (m, 8H), 3.70 – 3.83 (m, 2H), 4.13 – 4.35 (m, 6H), 4.41 – 4.54 (m, 2H), 4.67 (d, *J* = 10.5 Hz, 1H), 5.09 (d, *J* = 3.3 Hz, 1H), 5.80 (p, *J* = 8.9 Hz, 1H), 6.94 – 7.00 (m, 1H), 7.03 (d, *J* = 1.7 Hz, 1H), 7.28 (d, *J* = 7.8 Hz, 1H), 7.36 – 7.63 (m, 4H), 7.69 (d, *J* = 7.7 Hz, 1H), 7.81 (d, *J* = 9.1 Hz, 1H), 8.00 (d, *J* = 3.0 Hz, 1H), 8.38 (t, *J* = 5.9 Hz, 1H), 8.93 (s, 1H), 8.96 (s, 1H), 10.04 (s, 1H); **^{13}C NMR** (151 MHz, DMSO-d_6) δ 13.76, 16.18, 18.37, 20.04, 25.24, 27.58, 27.69, 31.45, 37.21, 37.90, 46.47, 48.45, 53.03, 55.44, 57.35, 57.92, 58.65, 68.13, 68.55, 68.90, 69.13, 69.89, 70.22, 106.70, 112.36, 115.29, 121.25, 123.26, 123.60, 124.72, 127.25, 127.78, 127.98,

129.36, 131.17, 131.34, 131.36, 131.68, 135.43, 142.22, 142.27, 143.61, 144.37, 148.08, 151.58, 154.92, 156.02, 158.39, 158.72, 160.91, 167.44, 167.61, 172.05, 202.57; **LC-MS** (ESI) (90% H_2O to 100% MeOH in 10 min, then 100% MeOH to 20 min, DAD 220-420 nm), t_R = 11.86 min, 99% purity, *m/z* $[M + H]^+$ calcd for $C_{59}H_{71}N_{11}O_9S$, 1110.52; found, 1111.1; **HRMS** (ESI) *m/z* $[M + H]^+$ calcd for $C_{59}H_{71}N_{11}O_9S$, 1110.5230; found, 1110.5213.

VHL ligand d (82)

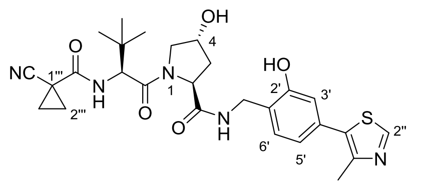

This compound was synthesized as described previously.[14]

^{1}H NMR (600 MHz, DMSO-*d*$_6$) δ 0.94 (s, 9H, $C(CH_3)_3$), 1.45 – 1.52 (m, 2H), 1.57 – 1.65 (m, 2H, 2’’’-H), 1.87 – 1.93 (m, 1H), 2.03 – 2.09 (m, 1H, 3-H), 2.43 (s, 3H, CH_3), 3.55 (d, *J* = 10.8, 1H), 3.60 – 3.65 (m, 1H, 5-H), 4.15 – 4.27 (m, 2H), 4.31 – 4.37 (m, 1H), 4.47 – 4.53 (m, 2H, 2-H, 4-H, NHC$\underline{H}$, NHC$\underline{H}_2$), 5.13 (d, *J* = 3.6 Hz, 1H, OH), 6.82 (d, *J* = 7.8 Hz, 1H), 6.87 – 6.93 (m, 1H), 7.28 – 7.36 (m, 2H, Ar-H, CONH), 8.49 (t, *J* = 6.0 Hz, 1H, CONH), 8.94 (s, 1H, 2”-H), 9.78 (s, 1H, Ar-OH); **^{13}C NMR** (151 MHz, DMSO-*d*$_6$) δ 13.90 (C-1’’’), 16.22 (CH_3), 16.77, 16.95 (C-2’’’), 26.25 (C($\underline{C}H_3)_3$), 36.39 ($\underline{C}(CH_3)_3$), 37.48 (C-3), 38.02 ($NHCH_2$), 56.78, 57.52, 58.93 (C-2, C-5, NHCH), 69.05 (C-4), 115.19 (C-3’), 119.49 (C-5’), 120.28 (CN), 125.39 (C-1’), 128.71 (C-6’), 130.83, 131.47 (C-4’, C-5’’), 147.63 (C-4”), 151.40 (C-2”), 154.97 (C-2’), 164.59, 168.87, 172.07 (CO); **MS** (ESI) *m/z* $[M + H]^+$ calcd for $C_{27}H_{33}N_5O_5S$, 540.22; found, 540.3.

VHL(d)-2-2-2 building block (83)

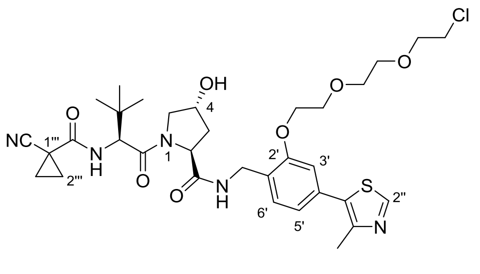

This compound was prepared using the General Procedure **V**, VHL ligand d (**82**, 162 mg) and mesylate **69** (89 mg). The crude product was purified by flash chromatography on silica gel (0% to 5% MeOH in CH_2Cl_2) to yield the title compound as a colorless solid.

Yield (108 mg, 52%); mp 67 – 70 °C; R_f = 0.30 (CH_2Cl_2/MeOH 19:1); **^{1}H NMR** (600 MHz, DMSO-d_6) δ 0.94 (s, 9H, (C(CH_3)$_3$), 1.45 – 1.55 (m, 2H), 1.57 – 1.67 (m, 2H, 2‴-H), 1.85 – 1.97 (m, 1H), 2.04 – 2.17 (m, 1H, 3-H), 2.45 (s, 3H, CH_3), 3.54 – 3.71 (m, 10H), 3.75 – 3.84 (m, 2H, 5-H, CH_2Cl, OCH_2), 4.15 – 4.39 (m, 5H), 4.48 – 4.53 (m, 2H, 2-H, 4-H, NHC$\underline{H}$, NHC$\underline{H}_2$, OCH_2), 5.14 (d, *J* = 3.7 Hz, 1H, OH), 6.95 (dd, *J* = 1.6, 7.7 Hz, 1H), 7.03 (d, *J* = 1.7 Hz, 1H), 7.37 (dd, *J* = 8.4, 21.6 Hz, 2H, Ar-H, CONH), 8.46 (t, *J* = 6.0 Hz, 1H, CONH), 8.97 (s, 1H, 2″-H); **^{13}C NMR** (151 MHz, DMSO-d_6) δ 13.88 (C-1‴), 16.13 (CH_3), 16.75, 16.94 (C-2‴), 26.23 (C($\underline{C}H_3$)$_3$), 36.37 ($\underline{C}$(CH_3)$_3$), 37.40 (C-3), 38.03 ($NHCH_2$), 43.68 (CH_2Cl), 56.79, 57.51, 58.98 (C-2, C-5, NHCH), 68.06, 69.06, 69.17, 69.88, 70.21, 70.72 (C-4, OCH_2), 112.30 (C-3′), 120.27 (CN), 121.13 (C-5′), 127.32, 127.87 (C-1′, C-6′), 131.05, 131.42 (C-4′, C-5″), 148.03 (C-4″), 151.57 (C-2″), 155.98 (C-2′), 164.58, 168.87, 171.93 (CO); **MS** (ESI) *m/z* $[M + H]^+$ calcd for $C_{33}H_{44}ClN_5O_7S$, 690.27; found, 699.6; **HRMS** (ESI) *m/z* $[M + H]^+$ calcd for $C_{33}H_{44}ClN_5O_7S$, 690.2723; found, 690.2707.

VHL(d)-based PROTAC 33

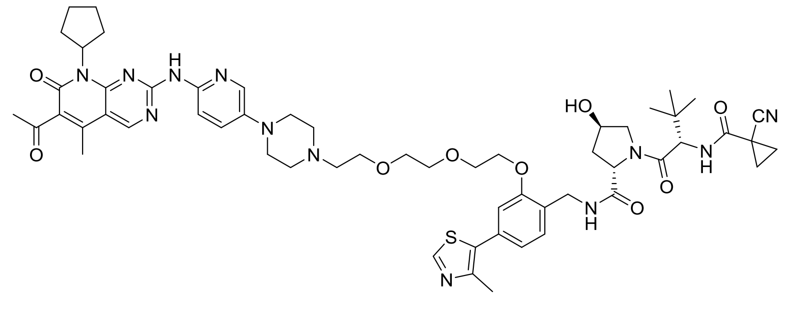

This compound was prepared using the General Procedure **IV** and precursor **83** (69 mg). The crude product was purified by column chromatography (CH_2Cl_2/MeOH+7N NH_3 19:1) to give the title product as a yellow solid.

Yield (65 mg, 59%); mp 118 – 122 °C; R_f = 0.23 (CH_2Cl_2/MeOH+7N NH_3 19:1); **^{1}H NMR** (600 MHz, DMSO-d_6) δ 0.93 (s, 9H), 1.42 – 1.65 (m, 6H), 1.66 – 1.82 (m, 2H), 1.82 – 1.97 (m, 3H), 2.03 – 2.12 (m, 1H), 2.23 (d, *J* = 3.3 Hz, 2H), 2.30 (s, 3H), 2.41 (s, 3H), 2.45 (s, 3H), 2.49 – 2.60 (m, 6H), 3.11 (t, *J* = 5.0 Hz, 4H), 3.48 – 3.68 (m, 8H), 3.70 – 3.86 (m, 2H), 4.15 – 4.39 (m, 5H), 4.46 – 4.57 (m, 2H), 5.14 (d, *J* = 3.7 Hz, 1H), 5.80 (p, *J* = 8.9 Hz, 1H), 6.95 (dd, *J* = 1.6, 7.7 Hz, 1H), 6.99 – 7.13 (m, 1H), 7.35 (d, *J* = 8.9 Hz, 1H), 7.37 – 7.51 (m, 2H), 7.82 (d, *J* = 9.0 Hz, 1H), 8.01 (d, *J* = 3.0 Hz, 1H), 8.46 (t, *J* = 6.1 Hz, 1H), 8.93 (s, 1H), 8.95 (s, 1H), 10.03 (s, 1H); **^{13}C NMR** (151 MHz, DMSO-d_6) δ 13.76, 13.88, 16.14, 16.75, 16.94, 25.24, 26.24, 27.69, 31.44, 36.38, 37.42, 38.04, 48.46, 53.05, 56.80, 57.37, 57.51, 58.98, 68.13, 68.57, 69.06, 69.15, 69.91, 70.24, 106.71, 112.29, 115.30, 120.27, 121.12, 124.73, 127.31, 127.91, 129.36, 131.07, 131.41, 135.44, 142.22, 143.62, 144.37, 148.02, 151.55, 154.92, 156.01, 158.38, 158.73, 160.91, 164.57, 168.87, 171.92, 202.57; **LC-MS** (ESI) (90% H_2O to 100% MeOH in 10 min, then

100% MeOH to 20 min, DAD 220-420 nm), t_R = 11.77 min, 99% purity, *m/z* $[M + H]^+$ calcd for $C_{57}H_{72}N_{12}O_9S$, 1101.54; found, 1102.2; **HRMS** (ESI) *m/z* $[M + H]^+$ calcd for $C_{57}H_{72}N_{12}O_9S$, 1101.5339; found, 1101.5325.

VHL ligand e (84)

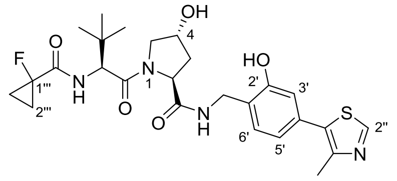

This compound was synthesized as described previously.[14]

^{1}H NMR (600 MHz, DMSO-d_6) δ 0.95 (s, 9H, $C(CH_3)_3$), 1.18 – 1.23 (m, 2H), 1.27 – 1.39 (m, 2H, 2‴-H), 1.87 – 1.95 (m, 1H), 2.02 – 2.14 (m, 1H, 3-H), 2.44 (s, 3H, CH_3), 3.56 – 3.68 (m, 2H, 5-H), 4.12 – 4.20 (m, 1H), 4.20 – 4.30 (m, 1H), 4.31 – 4.36 (m, 1H), 4.50 (t, *J* = 8.2 Hz, 1H), 4.55 – 4.62 (m, 1H, 2-H, 4-H, NHC$\underline{H}$, NHC$\underline{H}_2$), 5.14 (d, *J* = 3.7 Hz, 1H, OH), 6.83 (dd, *J* = 1.8, 7.8 Hz, 1H), 6.90 (d, *J* = 1.8 Hz, 1H), 7.25 (dd, *J* = 2.8, 9.3 Hz, 1H), 7.31 (d, *J* = 7.8 Hz, 1H, Ar-H, CONH), 8.48 (t, *J* = 6.0 Hz, 1H, CONH), 8.94 (s, 1H, 2″-H), 9.77 (s, 1H, Ar-OH); **^{13}C NMR** (151 MHz, DMSO-d_6) δ 12.95 (d, ^2J(C,F) = 40.1 Hz), 13.02 (d, ^2J(C,F) = 39.8 Hz, C-2‴), 16.21 (CH_3), 26.32 ($C(\underline{C}H_3)_3$), 36.20 ($\underline{C}(CH_3)_3$), 37.46 (C-3), 38.02 ($NHCH_2$), 56.70, 56.79, 58.90 (C-2, C-5, NHCH), 69.05 (C-4), 78.27 (d, ^1J(C,F) = 232.2 Hz, C-1‴), 115.18 (C-3′), 119.51 (C-5′), 125.40 (C-1′), 128.69 (C-6′), 130.81, 131.47 (C-4′, C-5″), 147.63 (C-4″), 151.37 (C-2″), 154.95 (C-2′), 168.21 (d, ^2J(C,F) = 19.9 Hz), 169.04, 172.09 (CO); **MS** (ESI) *m/z* $[M + H]^+$ calcd for $C_{26}H_{33}FN_4O_5S$, 533.22; found, 533.4.

VHL(e)-2-2-2 building block (85)

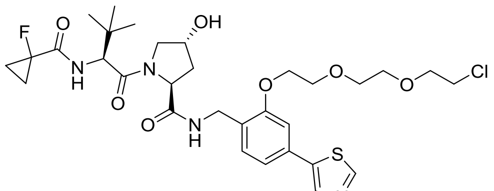

This compound was prepared using the General Procedure **V**, VHL ligand e (**84**, 160 mg) and mesylate **69** (89 mg). The crude product was purified by flash chromatography on silica gel (0% to 4% MeOH in CH_2Cl_2) to yield the title compound as a colorless solid.

Yield (107 mg, 52%); mp 58 – 60 °C; R_f = 0.21 (CH_2Cl_2/MeOH 19:1); **^{1}H NMR** (600 MHz, DMSO-d_6) δ 0.95 (s, 9H, $C(CH_3)_3$), 1.17 – 1.25 (m, 2H), 1.30 – 1.42 (m, 2H, 2‴-H), 1.86 – 1.97 (m, 1H), 2.04 – 2.12 (m, 1H, 3-H), 2.45 (s, 3H, CH_3), 3.55 – 3.71 (m, 10H), 3.76 – 3.83 (m, 2H, 5-H, CH_2Cl, OCH_2), 4.15 – 4.24 (m, 3H), 4.26 – 4.38 (m, 2H), 4.51 (t, *J* = 8.2 Hz, 1H), 4.59 (dd, *J* = 1.1, 9.1 Hz, 1H, 2-H, 4-H, NHC$\underline{H}$, NHC$\underline{H}_2$, OCH_2), 5.14 (d, *J* = 3.6 Hz, 1H, OH), 6.96 (dd, *J* = 1.6, 7.7 Hz, 1H), 7.03 (d, *J* = 1.6 Hz, 1H), 7.27 (dd, *J* = 2.8, 9.3 Hz, 1H), 7.39 (d, *J* = 7.8 Hz, 1H, Ar-H, CONH), 8.45 (t, *J* = 6.0 Hz, 1H, CONH), 8.97 (s, 1H, 2″-H); **^{13}C NMR** (151 MHz, DMSO-d_6) δ 12.95 (d, ^2J(C,F) = 39.6 Hz), 13.02 (d, ^2J(C,F) = 39.7 Hz, C-2‴), 16.13 (CH_3), 26.31 (C($\underline{C}H_3)_3$), 36.19 ($\underline{C}(CH_3)_3$), 37.39 (C-3), 38.04 ($NHCH_2$), 43.68 (CH_2Cl), 56.70, 56.81, 58.97 (C-2, C-5, NHCH), 68.05, 69.06, 69.16, 69.87, 70.21, 70.72 (C-4, OCH_2), 78.27 (d, ^1J(C,F) = 232.5 Hz, C-1‴), 112.28 (C-3′), 121.17 (C-5′), 127.33, 127.86 (C-1′, C-6′), 131.03, 131.42 (C-4′, C-5″), 148.03 (C-4″), 151.56 (C-2″), 155.96 (C-2′), 168.21 (d, ^2J(C,F) = 20.7 Hz), 169.06, 171.97 (CO); **MS** (ESI) *m/z* $[M + H]^+$ calcd for $C_{32}H_{44}ClFN_4O_7S$, 683.26; found, 683.6; **HRMS** (ESI) *m/z* $[M + H]^+$ calcd for $C_{32}H_{44}ClFN_4O_7S$, 683.2676; found, 683.2662.

VHL(e)-based PROTAC 34 (CST651)

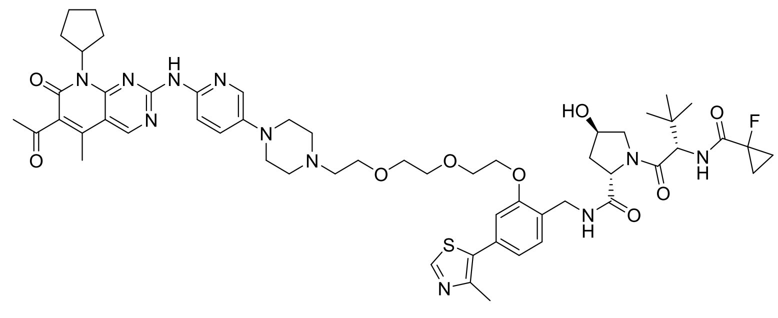

This compound was prepared using the General Procedure **IV** and precursor **85** (68 mg). The crude product was purified by column chromatography (CH_2Cl_2/MeOH+7N NH_3 29:1) followed by HPLC purification (gradient of 80% to 100% v/v MeOH) to give the title compound as a yellow solid.

Yield (38 mg, 34%); mp 120 – 124 °C; R_f = 0.25 (CH_2Cl_2/MeOH+7N NH_3 19:1); **^{1}H NMR** (600 MHz, DMSO-d_6) δ 0.95 (s, 9H), 1.18 – 1.25 (m, 2H), 1.29 – 1.40 (m, 2H), 1.51 – 1.62 (m, 2H), 1.71 – 1.79 (m, 2H), 1.83 – 1.95 (m, 3H), 2.04 – 2.11 (m, 1H), 2.18 – 2.27 (m, 2H), 2.30 (s, 3H), 2.41 (s, 3H), 2.45 (s, 3H), 2.50 – 2.59 (m, 6H), 3.07 – 3.13 (m, 4H), 3.49 – 3.70 (m, 8H), 3.74 – 3.85 (m, 2H), 4.15 – 4.37 (m, 5H), 4.51 (t, *J* = 8.2 Hz, 1H), 4.58 (d, *J* = 9.0 Hz, 1H), 5.14 (d, *J* = 3.7 Hz, 1H), 5.80 (p, *J* = 8.9 Hz, 1H), 6.96 (dd, *J* = 1.7, 7.8 Hz, 1H), 7.01 – 7.07 (m, 1H), 7.26 (dd, *J* = 2.8, 9.2 Hz, 1H), 7.40 (dd, *J* = 3.0, 8.7 Hz, 2H), 7.82 (d, *J* = 8.9 Hz, 1H), 8.01 (d, *J* = 3.0 Hz, 1H), 8.46 (s, 1H), 8.93 (s, 1H), 8.95 (s, 1H), 10.03 (s, 1H); **^{13}C NMR** (151 MHz, DMSO-d_6) δ 12.95 (d, ^2J(C,F) = 39.8 Hz), 13.02 (d, ^2J(C,F) = 40.4 Hz), 13.76, 16.14, 16.18, 25.24, 26.31, 27.69, 31.45, 36.19, 37.41, 38.04, 48.46, 53.03, 56.69, 56.82, 57.37, 58.96,

68.12, 68.57, 69.06, 69.15, 69.91, 70.24, 78.27 (d, ^1J(C,F) = 232.3 Hz), 106.70, 112.27, 115.29, 121.16, 124.72, 127.33, 127.89, 129.36, 131.04, 131.36, 131.42, 135.44, 142.22, 143.62, 144.37, 148.03, 151.55, 154.92, 155.99, 158.39, 158.72, 160.91, 168.20 (d, ^2J(C,F) = 19.9 Hz), 169.05, 171.96, 202.57; **LC-MS** (ESI) (90% H_2O to 100% MeOH in 10 min, then 100% MeOH to 20 min, DAD 220-420 nm), t_R = 11.95 min, 99% purity, *m/z* $[M + H]^+$ calcd for $C_{56}H_{72}FN_{11}O_9S$, 1094.53; found, 1095.2; **HRMS** (ESI) *m/z* $[M + H]^+$ calcd for $C_{56}H_{72}FN_{11}O_9S$, 1094.5292; found, 1094.5271.

K. Synthesis of IAP-based degraders

Monomeric IAP ligand (5)

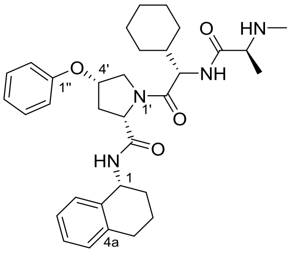

This compound was synthesized as described previously.[15]

^{1}H NMR (500 MHz, DMSO-d_6) δ 0.88 – 1.18 (m, 9H, CH_2, CH_3), 1.52 – 1.85 (m, 10H, CH_2), 2.16 (s, 3H, NHC$\underline{H}_3$), 2.04 – 2.13 (m, 1H), 2.50 – 2.59 (m, 1H), 2.62 – 2.77 (m, 2H), 2.87 – 3.00 (m, 1H), 3.64 (dd, J = 4.5, 10.8 Hz, 1H, 2-H, 3-H, 4-H, CH, CH_2), 4.23 – 4.34 (m, 1H), 4.39 (dd, J = 7.1, 8.6 Hz, 1H, 2'-H, NHC$\underline{H}$CH), 4.45 (dd, J = 5.2, 9.0 Hz, 1H, C$\underline{H}$CH$_3$), 4.87 – 4.95 (m, 1H), 5.04 (p, J = 5.4 Hz, 1H, 1-H, 4'-H), 6.88 – 6.92 (m, 2H, Ar-H), 6.95 (t, J = 7.5 Hz, 1H, Ar-H), 7.02 – 7.19 (m, 3H, Ar-H), 7.21 – 7.33 (m, 3H, Ar-H), 7.84 (d, J = 8.6 Hz, 1H), 7.89 (d, J = 8.7 Hz, 1H, CONH); **^{13}C NMR** (126 MHz, DMSO-d_6) δ 19.19 (CH$\underline{C}$H$_3$), 19.97 (C-3), 25.55, 25.75, 25.92 (CH_2), 27.91, 28.86, 29.18, 29.77 (CH_2, C-2, C-4), 34.39, 34.54 (C-3', $NHCH_3$), 46.73 (C-1), 52.15 (NH$\underline{C}$HCH), 54.45 (C-5'), 58.63 ($\underline{C}$HCH$_3$), 59.28 (NCH), 74.96 (C-4'), 115.69 (C-3''), 121.20 (C-4''), 125.86, 126.84, 128.54, 128.76 (C-5, C-6, C-7, C-8), 129.69 (C-2''), 137.14, 137.35 (C-4a, C-8a), 157.06 (C-1''), 169.98, 170.57, 174.56 (CO). The signal for NHCH$\underline{C}$H is missing (overlapping solvent peaks); **LC-MS** (ESI) (90% H_2O to 100% MeOH in 10 min, then 100% MeOH to 20 min, DAD 205-220 nm), t_R = 11.98 min, 97% purity, m/z $[M + H]^+$ calcd for $C_{33}H_{44}N_4O_4$, 561.34; found, 561.3.

IAP ligand (86)

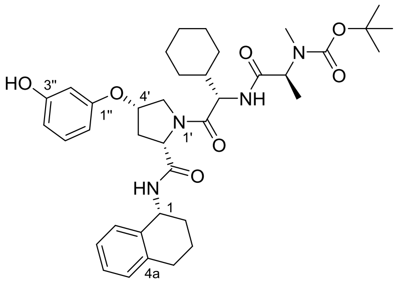

This compound was synthesized as described previously.[16]

^{1}**H NMR** (600 MHz, DMSO-d_6) δ 0.71 – 1.24 (m, 9H, CH_2, CH_3), 1.38 (s, 9H, $C(CH_3)_3$), 1.52 – 1.84 (m, 10H), 2.01 – 2.14 (m, 1H), 2.43 – 2.53 (m, 1H), 2.64 – 2.76 (m, 5H), 3.60 (dd, *J* = 4.4, 10.8 Hz, 1H, 2-H, 3-H, 4-H, CH, CH_2, NCH_3), 4.18 – 4.27 (m, 1H), 4.32 (t, *J* = 7.8 Hz, 1H, 2'-H, $NHC\underline{H}$), 4.44 (dd, *J* = 5.1, 9.0 Hz, 1H, $C\underline{H}CH_3$), 4.85 – 5.00 (m, 2H, 1-H, 4'-H), 6.23 – 6.40 (m, 3H, 2''-H, 4''-H, 6''-H), 7.00 – 7.16 (m, 4H, 5-H, 6-H, 7-H, 5''-H), 7.24 (d, *J* = 7.5 Hz, 1H, 8-H), 7.84 (d, *J* = 8.5 Hz, 1H), 8.31 (d, *J* = 8.4 Hz, 1H, CONH), 9.40 (s, 1H, OH); ^{13}**C NMR** (151 MHz, DMSO-d_6) δ 15.11 (br s, suppressed CH_3), 20.04 (C-3), 25.61, 25.78, 25.92 (CH_2), 28.17 ($C(\underline{C}H_3)_3$), 28.90, 29.07, 29.65, 29.81 (C-2, C-4, CH_2), 30.16 (NCH_3), 34.64 (C-3'), 39.52 (CH), 46.78 (C-1), 52.25 (NHCH), 53.27 (br s, suppressed $\underline{C}HCH_3$), 55.23 (C-5'), 58.65 (NCH), 74.96 (OCH), 79.17 ($\underline{C}(CH_3)_3$), 103.14 (C-2''), 106.14 (C-4''), 108.55 (C-6''), 125.89, 126.86, 128.51 (C-6, C-7, C-8), 128.79 (C-5), 130.06 (C-5''), 137.17, 137.40 (C-4a, C-8a), 155.27 (br s, suppressed CO), 158.29, 158.76 (C-1'', C-3''), 169.40, 169.97, 170.38 (CO); **MS** (ESI) *m/z* $[M + H]^+$ calcd for $C_{38}H_{52}N_4O_7$, 677.39; found, 677.6; **HRMS** (ESI) *m/z* $[M + H]^+$ calcd for $C_{38}H_{53}O_7N_4$, 677.3909; found, 677.3899.

^{1}**H NMR** (400 MHz, $CDCl_3$) δ 0.75 – 1.00 (m, 5H, CH_2), 1.30 (d, *J* = 7.3 Hz, 3H, $CHC\underline{H}_3$), 1.37 – 1.43 (m, 1H, CH_2), 1.47 (s, 9H, $C(CH_3)_3$), 1.48 – 1.59 (m, 4H, CH_2), 1.70 – 1.85 (m, 4H, 2-H, 3-H), 1.94 – 2.05 (m, 1H, CH), 2.28 (symm m, 1H, 4'-H), 2.70 – 2.76 (m, 2H, 4-H, 3'-H), 2.77 (s, 3H, NCH_3), 2.84 (d, *J* = 13.8 Hz, 1H, 4-H), 3.75 (d, *J* = 11.4 Hz, 1H, 5'-H), 4.17 (dd, *J* = 4.6, 11.4 Hz, 1H, 5'-H), 4.41 (t, *J* = 8.1 Hz, 1H, 1-H), 4.52 – 4.70 (m, 1H, 4'-H), 4.75 (dd, *J* = 2.0, 9.2 Hz, 1H, $NHC\underline{H}$), 4.86 (t, *J* = 4.3 Hz, 1H, NCH), 5.10 (q, *J* = 7.3 Hz, 1H, $C\underline{H}CH_3$), 6.27 (t, *J* = 1.8 Hz, 1H, 2''-H), 6.33 (dd, *J* = 1.8, 8.3 Hz, 1H, 6''-H), 6.47 (dd, *J* = 1.8, 8.1 Hz, 1H, 4''-H), 6.62 (d, *J* = 7.3 Hz, 1H, CONH), 6.70 (br s, 1H, CONH), 7.03 – 7.15 (m, 4H, 5-H, 6-H, 7-H, 8-H), 7.30 (d, *J* = 7.6 Hz, 1H, 5''-H). The resonance for OH is missing; ^{13}**C NMR** (101 MHz, $CDCl_3$) δ 14.45 (br s, suppressed CH_3), 19.83 (C-3), 25.44, 25.55, 25.79 (CH_2), 28.35 ($C(\underline{C}H_3)_3$), 28.45, 29.21, 29.74, 29.81 (C-2, C-4, CH_2), 30.16 (NCH_3), 33.40 (C-3'), 40.52 (CH), 47.69 (C-1), 53.40 (NHCH), 53.74 (br s, suppressed $\underline{C}HCH_3$), 55.39 (C-5'), 60.01 (NCH), 75.95 (OCH), 80.85 ($\underline{C}(CH_3)_3$), 103.41 (C-2''), 107.52 (C-4''), 109.06 (C-6''), 126.38 (C-6), 127.22 (C-7), 128.91 (C-8), 129.10 (C-5), 130.26 (C-5''), 136.39 (C-4a), 137.45 (C-8a), 156.45 (br s, suppressed CO), 157.75 (C-3''), 157.82 (C-1''), 169.67, 171.89, 172.22 (CO).

IAP-2-2-2-2 building block (87)

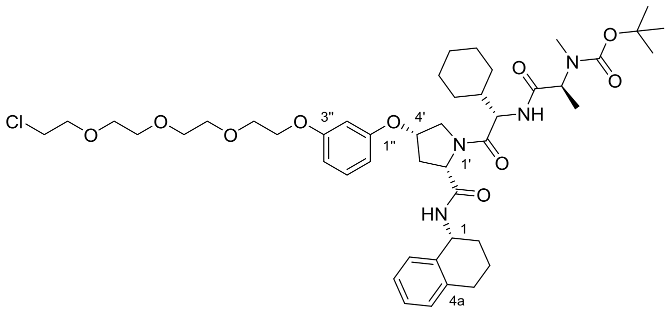

To a solution of IAP ligand **86** (0.20 g, 0.30 mmol) in dry DMF (3 mL), K_2CO_3 (62 mg, 0.45 mmol) was added under argon atmosphere. Subsequently, a solution of **71** (105 mg, 0.36 mmol) in dry DMF (5 mL) was added, followed by stirring of the mixture at 60 °C for 24 h. After the reaction was complete, the volatiles were evaporated and the crude product was purified by column chromatography (CH_2Cl_2/MeOH 50:1) to give a colorless oil.

Yield (105 mg, 40%); R_f = 0.12 (CH_2Cl_2/MeOH 20:1); **^{1}H NMR** (400 MHz, $CDCl_3$) δ 0.75 – 0.98 (m, 5H, CH_2), 1.30 (d, *J* = 7.2 Hz, 3H, CHC<u>H</u>$_3$), 1.38 – 1.42 (m, 1H, CH_2), 1.47 (s, 9H, $C(CH_3)_3$), 1.50 – 1.63 (m, 4H, CH_2), 1.75 – 1.90 (m, 4H, 2-H, 3-H), 1.98 – 2.10 (m, 1H, CH), 2.32 (symm m, 1H, 4'-H), 2.68 – 2.82 (m, 5H, 4-H, 3'-H, NCH_3), 2.89 (d, *J* = 13.7 Hz, 1H, 4-H), 3.62 (t, *J* = 5.6 Hz, 2H, CH_2), 3.66 – 3.78 (m, 11H, CH_2, 5'-H), 3.84 (t, *J* = 4.8 Hz, 2H, CH_2Cl), 4.06 (t, *J* = 5.1 Hz, 2H, OCH_2), 4.19 (dd, *J* = 4.9, 11.5 Hz, 1H, 5'-H), 4.42 (t, *J* = 7.9 Hz, 1H, 1-H), 4.50 – 4.68 (m, 1H, 4'-H), 4.76 (dd, *J* = 1.7, 9.5 Hz, 1H, NHC<u>H</u>), 4.93 (t, *J* = 4.6 Hz, 1H, NCH), 5.12 (q, *J* = 7.2 Hz, 1H, C<u>H</u>CH_3), 6.38 – 6.44 (m, 2H, 2''-H, 6''-H), 6.55 (dd, *J* = 1.7, 8.1 Hz, 1H, 4''-H), 6.59 (d, *J* = 8.1 Hz, 1H, CONH), 6.56 (br s, 1H, CONH), 7.02 – 7.20 (m, 4H, 5-H, 6-H, 7-H, 8-H), 7.29 (d, *J* = 7.2 Hz, 1H, 5''-H); **^{13}C NMR** (101 MHz, $CDCl_3$) δ 13.41 (br s, CH_3), 19.94 (C-3), 25.40, 25.51, 25.75 (CH_2), 28.29 (C(<u>C</u>H_3)$_3$), 28.38, 29.20, 29.70, 29.89 (C-2, C-4, CH_2), 30.00 (NCH_3), 33.35 (C-3'), 40.52 (CH), 42.68 (CH_2Cl), 47.57 (C-1), 53.54 (NH<u>C</u>H), 53.68 (br s, suppressed <u>C</u>HCH_3), 55.20 (C-5'), 60.05 (NCH), 67.39, 69.63, 70.57, 70.60, 70.64, 70.76, 71.29 (OCH_2), 76.13 (OCH), 80.67 <u>C</u>(CH_3)$_3$), 102.97 (C-2''), 107.63 (C-4''), 108.35 (C-6''), 126.16 (C-6), 127.09 (C-7), 128.60 (C-8), 129.04 (C-5), 130.03 (C-5''), 136.52 (C-4a), 137.33 (C-8a), 156.24 (br s, CO), 157.78 (C-3''), 160.03 (C-1''), 169.32 (CO), 171.54 (CO), 172.22 (CO); **HRMS** (ESI) *m/z* $[M + H]^+$ calcd for $C_{46}H_{67}O_{10}N_4Cl$, 871.4619; found, 871.4586.

Boc-protected intermediate 88

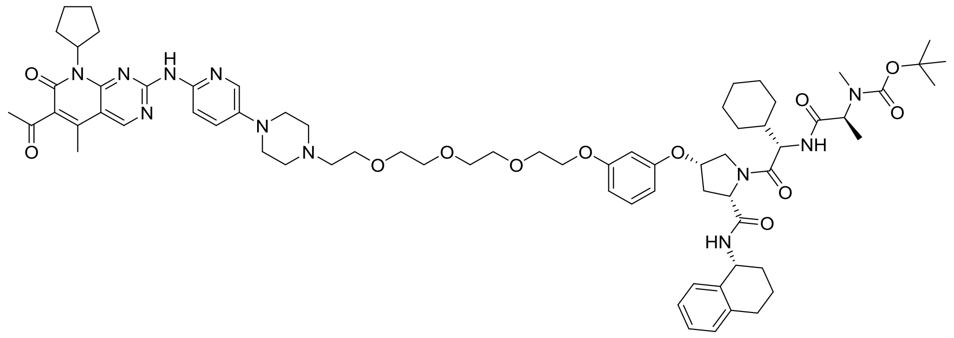

This compound was prepared using the General Procedure **IV** and precursor **87** (87 mg). The crude product was purified by column chromatography (CH_2Cl_2/MeOH+7N NH_3 29:1) followed by HPLC purification using a gradient of 80% to 100% v/v MeOH. The title compound was obtained as a yellow solid after lyophilisation and was directly used in the next step.

Yield (82 mg, 62%); mp 120 – 122 °C; R_f = 0.15 (CH_2Cl_2/MeOH+7N NH_3 29:1); **LC-MS** (ESI) (90% H_2O to 100% MeOH in 10 min, then 100% MeOH to 20 min, DAD 220-400 nm), t_R = 13.21 min, 99% purity, *m/z* $[M + H]^+$ calcd for $C_{70}H_{95}N_{11}O_{12}$, 1220.50; found, 1282.9; **HRMS** (ESI) *m/z* $[M + H]^+$ calcd for $C_{70}H_{95}N_{11}O_{12}$, 1282.7234; found, 1282.7221.

IAP-based degrader 35

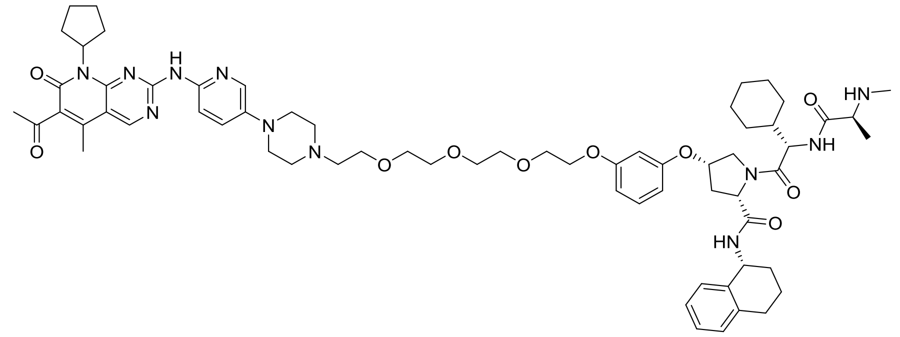

PROTAC precursor **88** (47 µmol, 61 mg) was dissolved in 1M HCl in EtOAc (3 mL) and stirred at rt for 4 h. The yellow precipitate was filtered off, washed with dry Et_2O (3 × 5 mL) and dried *in vacuo*. The title compound (HCl salt) was then neutralized by eluting it with CH_2Cl_2/MeOH+7N NH_3 19:1 on a small silica gel column. After evaporation of the solvent, a yellow solid was obtained.

Yield (44 mg, 79%); mp 94 – 96 °C; R_f = 0.30 (CH_2Cl_2/MeOH+7N NH_3 19:1); **^{1}H NMR** (600 MHz, DMSO-d_6) δ 0.86 – 1.13 (m, 8H), 1.50 – 1.83 (m, 15H), 1.83 – 1.92 (m, 2H), 2.02 – 2.12 (m, 1H), 2.17 (s, 3H), 2.19 – 2.27 (m, 3H), 2.29 (s, 3H), 2.41 (s, 3H), 2.48 – 2.55 (m, 2H), 2.56 (t, *J* = 5.0 Hz, 4H), 2.62 – 2.85 (m, 2H), 2.97 (q, *J* = 6.9 Hz, 1H), 3.12 (t, *J* = 5.0 Hz, 4H), 3.48 – 3.65 (m, 11H), 3.69 – 3.76 (m, 2H), 3.95

– 4.15 (m, 2H), 4.28 (dd, *J* = 6.1, 10.9 Hz, 1H), 4.38 (t, *J* = 7.9 Hz, 1H), 4.43 (dd, *J* = 5.4, 9.0 Hz, 1H), 4.91 (q, *J* = 7.5, 8.0 Hz, 1H), 5.03 (p, *J* = 5.3 Hz, 1H), 5.80 (p, *J* = 8.9 Hz, 1H), 6.45 – 6.59 (m, 3H), 6.93 – 7.19 (m, 4H), 7.23 (d, *J* = 7.5 Hz, 1H), 7.43 (dd, *J* = 3.1, 9.1 Hz, 1H), 7.80 – 7.88 (m, 2H), 7.93 (d, *J* = 8.6 Hz, 1H), 8.02 (d, *J* = 3.0 Hz, 1H), 8.93 (s, 1H), 10.04 (s, 1H); **^{13}C NMR** (151 MHz, DMSO-d_6) δ 13.76, 19.09, 20.04, 25.24, 25.58, 25.76, 25.94, 27.69, 27.94, 28.89, 29.17, 29.82, 31.44, 34.28, 34.56, 40.23, 46.75, 48.47, 52.13, 53.04, 54.51, 57.36, 58.61, 59.16, 67.32, 68.53, 69.07, 69.84, 69.96, 69.99, 70.11, 74.99, 102.42, 106.71, 107.45, 108.05, 115.32, 124.74, 125.88, 126.86, 128.52, 128.78, 129.36, 130.21, 135.44, 137.15, 137.38, 142.23, 143.63, 144.38, 154.92, 158.27, 158.40, 158.73, 159.88, 160.92, 170.02, 170.55, 174.37, 202.58; **LC-MS** (ESI) (90% H_2O to 100% MeOH in 10 min, then 100% MeOH to 20 min, DAD 220-400 nm), t_R = 13.00 min, 99% purity, *m/z* $[M + H]^+$ calcd for $C_{70}H_{95}N_{11}O_{12}$, 1182.67; found, 1183.3; **HRMS** (ESI) *m/z* $[M + H]^+$ calcd for $C_{65}H_{87}N_{11}O_{10}$, 1182.6710; found, 1182.6700.

(–)IAP ligand (89)

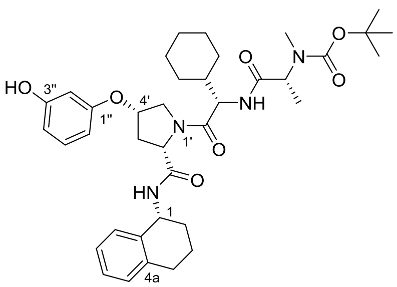

This compound was synthesized as described previously.[16]

^{1}H NMR (600 MHz, DMSO-d_6) δ 0.82 – 1.28 (m, 9H, CH_2, CH_3), 1.35 (s, 9H, $C(CH_3)_3$), 1.53 – 1.82 (m, 10H), 2.01 – 2.21 (m, 1H), 2.27 – 2.49 (m, 1H), 2.62 – 2.71 (m, 2H), 2.74 (s, 3H), 3.62 (br s, 1H, 2-H, 3-H, 4-H, CH, CH_2, NCH_3), 4.31 (p, *J* = 13.5, 15.6 Hz, 2H, 2'-H, NHC<u>H</u>), 4.44 (dd, *J* = 4.8, 9.0 Hz, 1H, C<u>H</u>CH_3), 4.88 – 4.96 (m, 2H, 4'-H), 6.19 – 6.45 (m, 3H, 2''-H, 4''-H, 6''-H), 6.93 – 7.19 (m, 4H, 5-H, 6-H, 7-H, 5''-H), 7.25 (d, *J* = 7.5 Hz, 1H, 8-H), 7.86 (br s, 1H), 7.91 (br s, 1H, CONH), 9.39 (s, 1H, OH); **^{13}C NMR** (151 MHz, DMSO-d_6) δ 15.73 (br s, suppressed CH_3), 19.94 (C-3), 25.59, 25.73, 25.98 (CH_2), 28.15 (<u>C</u>$(CH_3)_3$), 28.88, 29.04, 29.65, 29.75 (C-2, C-4, CH_2), 30.19 (NCH_3), 34.77 (C-3'), 46.79 (C-1), 52.27 (NHCH), 52.84 (br s, suppressed <u>C</u>HCH_3), 54.93 (C-5'), 58.57 (NCH), 74.96 (OCH_2), 79.23 (<u>C</u>$(CH_3)_3$),, 103.14 (C-2''), 106.09 (C-4''), 108.55 (C-6''), 125.88, 126.87, 128.59 (C-6, C-7, C-8), 128.79 (C-5), 130.06 (C-5''), 137.17, 137.36 (C-4a, C-8a), 155.19 (br s, CO), 158.24, 158.75, (C-1'', C-3''), 169.41, 170.47, 170.99 (CO); **MS** (ESI) *m/z* $[M + H]^+$ calcd for $C_{38}H_{52}N_4O_7$, 677.39; found, 677.7; **HRMS** (ESI) *m/z* $[M + H]^+$ calcd for $C_{38}H_{53}O_7N_4$, 677.3909; found, 677.3896.

(–)IAP-2-2-2-2 building block (90)

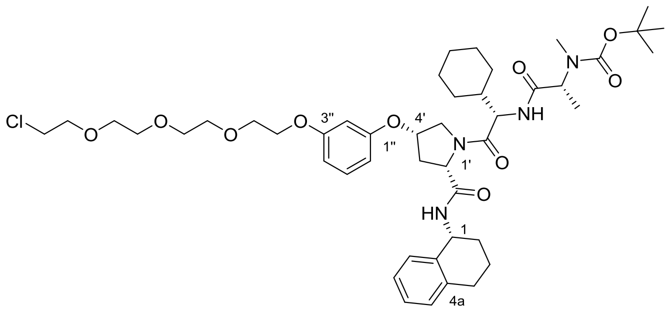

To a solution of (–)IAP ligand **89** (0.20 g, 0.30 mmol) in dry DMF (3 mL), K_2CO_3 (62 mg, 0.45 mmol) was added under argon atmosphere. Subsequently, a solution of **71** (105 mg, 0.36 mmol) in dry DMF (5 mL) was added, followed by stirring of the mixture at 60 °C for 24 h. After the reaction was complete, the volatiles were evaporated and the crude product was purified by column chromatography (CH_2Cl_2/MeOH 20:1) to give a colorless oil.

Yield (120 mg, 46%); R_f = 0.12 (CH_2Cl_2/MeOH 20:1); ^{1}**H NMR** (400 MHz, $CDCl_3$) δ 0.80 – 1.00 (m, 5H, CH_2), 1.32 (d, *J* = 6.8 Hz, 3H, CHC$\underline{H}_3$), 1.40 – 1.44 (m, 1H, CH_2), 1.47 (s, 9H, $C(CH_3)_3$), 1.50 – 1.62 (m, 4H, CH_2), 1.75 – 1.91 (m, 4H, 2-H, 3-H), 2.00 – 2.08 (m, 1H, CH), 2.33 (symm m, 1H, 4'-H), 2.64 – 2.82 (m, 5H, 4-H, 3'-H, NCH_3), 2.87 (d, *J* = 13.9 Hz, 1H, 4-H), 3.62 (t, *J* = 5.9 Hz, 2H, CH_2), 3.65 – 3.78 (m, 11H, CH_2, 5'-H), 3.84 (t, *J* = 4.5 Hz, 2H, CH_2Cl), 4.06 (t, *J* = 5.0 Hz, 2H OCH_2), 4.16 (dd, *J* = 4.8, 11.4 Hz, 1H, 5'-H), 4.42 (t, *J* = 6.5 Hz, 1H, 1-H), 4.55 – 4.80 (m, 2H, 4'-H, NHC$\underline{H}$), 4.94 (t, *J* = 4.5 Hz, 1H, NCH), 5.12 (q, *J* = 6.8 Hz, 1H, C$\underline{H}$$CH_3$), 6.36 – 6.44 (m, 2H, 2''-H, 6''-H), 6.51 – 6.57 (m, 2H, 4''-H, CONH), 6.60 (br s, 1H, CONH), 7.02 – 7.19 (m, 4H, 5-H, 6-H, 7-H, 8-H), 7.29 (d, *J* = 7.6 Hz, 1H, 5''-H); ^{13}**C NMR** (101 MHz, $CDCl_3$) δ 13.47 (br s, CH_3), 19.97 (C-3), 25.40, 25.58, 25.71 (CH_2), 28.27 (C($\underline{C}H_3$)$_3$), 28.27, 29.20, 29.74, 29.92 (C-2, C-4, CH_2), 30.10 (NCH_3), 33.41 (C-3'), 40.52 (CH), 42.68 (CH_2Cl), 47.56 (C-1), 53.49 (NH$\underline{C}$H), 53.75 (br s, suppressed $\underline{C}$HCH_3), 55.25 (C-5'), 60.03 (NCH), 67.39, 69.62, 70.57, 70.60, 70.64, 70.76, 71.29 (OCH_2), 76.24 (OCH), 80.66 $\underline{C}$(CH_3)$_3$), 102.97 (C-2''), 107.63 (C-4''), 108.35 (C-6''), 126.16 (C-6), 127.09 (C-7), 128.60 (C-8), 129.04 (C-5), 130.03 (C-5''), 136.52 (C-4a), 137.33 (C-8a), 156.24 (br s, CO), 157.78 (C-3''), 160.03 (C-1''), 169.32 (CO), 171.54 (CO), 172.22 (CO); **HRMS** (ESI) *m/z* calcd for $C_{46}H_{67}O_{10}N_4Cl$, 871.4619; found, 871.5594.

Boc-protected intermediate 91

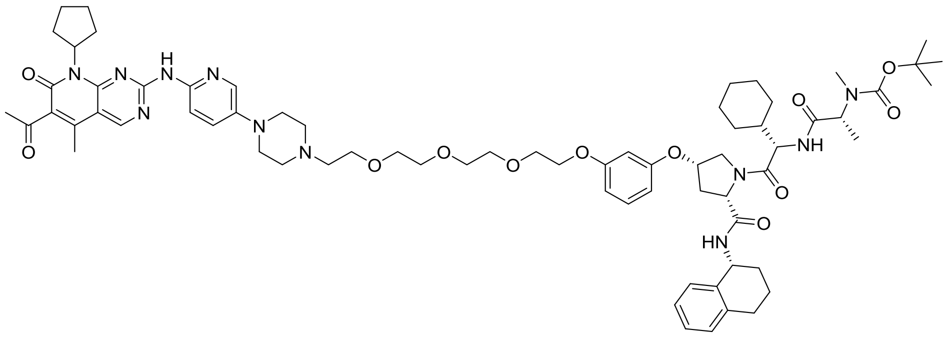

This compound was prepared using the General Procedure **IV** and precursor **90** (87 mg). The crude product was purified by column chromatography (CH_2Cl_2/MeOH+7N NH_3 29:1) followed by HPLC purification using a gradient of 80% to 100% v/v MeOH. The title compound was obtained as a yellow solid after lyophilisation and was directly used in the next step.

Yield (87 mg, 68%); mp 110 – 114 °C; R_f = 0.30 (CH_2Cl_2/MeOH+7N NH_3 19:1); **LC-MS** (ESI) (90% H_2O to 100% MeOH in 10 min, then 100% MeOH to 20 min, DAD 220-420 nm), t_R = 13.19 min, 99% purity, *m/z* $[M + H]^+$ calcd for $C_{70}H_{95}N_{11}O_{12}$, 1220.50; found, 1282.9; **HRMS** (ESI) *m/z* $[M + H]^+$ calcd for $C_{70}H_{95}N_{11}O_{12}$, 1282.7234; found, 1282.7221.

IAP-based negative control 36

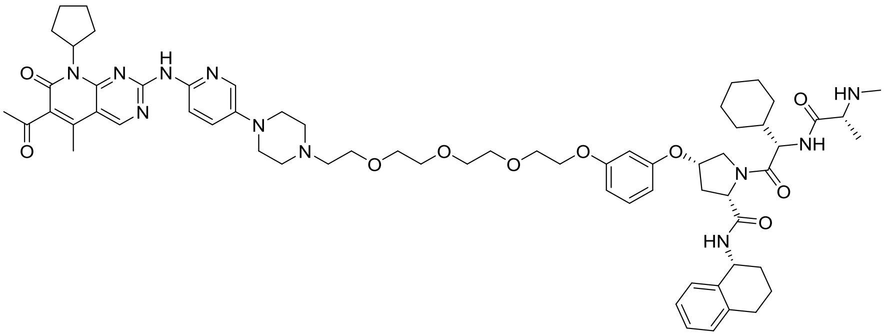

PROTAC precursor **91** (51 µmol, 65 mg) was dissolved in 1M HCl in EtOAc (5 mL) and stirred at rt for 4 h. The yellow precipitate was filtered off, washed with dry Et_2O (3 × 5 mL) and dried *in vacuo*. The title compound (HCl salt) was then neutralized by eluting it with CH_2Cl_2/MeOH+7N NH_3 19:1 on a small silica gel column. After evaporation of the solvent, a yellow solid was obtained.

Yield (52 mg, 86%); mp 100 – 104 °C; R_f = 0.28 (CH_2Cl_2/MeOH+7N NH_3 19:1); **^{1}H NMR** (600 MHz, DMSO-d_6) δ 0.70 – 1.29 (m, 9H), 1.41 – 1.82 (m, 14H), 1.82 – 1.90 (m, 2H), 1.99 – 2.14 (m, 1H), 2.18 (s, 3H), 2.19 – 2.27 (m, 3H), 2.29 (s, 3H), 2.41 (s, 3H), 2.48 – 2.54 (m, 2H), 2.54 – 2.60 (m, 4H), 2.60 – 2.76 (m, 2H), 2.87 – 3.04 (m, 1H), 3.12 (t, *J* = 5.0 Hz, 4H), 3.48 – 3.65 (m, 11H), 3.67 – 3.78 (m, 2H), 4.04 (dd, *J* = 3.7, 6.0 Hz, 2H), 4.30 (dd, *J* = 6.0, 10.7 Hz, 1H), 4.33 – 4.40 (m, 1H), 4.43 (dd, *J* = 5.4, 8.9 Hz, 1H), 4.74 – 4.94 (m, 1H), 5.02 (p, *J* = 5.3 Hz, 1H), 5.80 (p, *J* = 8.9 Hz, 1H), 6.45 – 6.58 (m, 3H), 6.94 – 7.20 (m, 4H), 7.20 – 7.32 (m, 1H), 7.43 (dd, *J* = 3.1, 9.1 Hz, 1H), 7.83 (dd, *J* = 8.7, 10.6 Hz, 2H), 7.95 (d, *J* = 8.5 Hz, 1H), 8.02 (d, *J* = 3.0 Hz, 1H), 8.93 (s, 1H), 10.04 (s, 1H); **^{13}C NMR** (151 MHz, DMSO-d_6) δ 13.75, 19.16, 20.05, 25.23, 25.58, 25.76, 25.96, 27.69, 27.98, 28.88, 29.24, 29.82, 31.44, 34.51, 34.57, 40.23, 46.75, 48.47, 52.11, 53.04, 54.65, 57.36, 58.61, 59.01, 67.32, 68.53, 69.07, 69.84, 69.96, 69.98, 70.10, 75.00, 102.41, 106.70, 107.44, 108.04, 115.31, 124.73, 125.87, 126.85, 128.49, 128.78, 129.35, 130.20, 135.43, 137.14, 137.39, 142.22, 143.62, 144.38, 154.91, 158.28, 158.39, 158.73, 159.88, 160.91, 170.01, 170.49, 174.53, 202.56; **HPLC** (95% H_2O (with 0.1% TFA) to 95% MeCN in 10 min, then 95% MeCN for 4 min), t_R = 5.60 min, 98.48% purity, detection at 254 nm; **MS** (ESI) *m/z* $[M + H]^+$ calcd for $C_{65}H_{87}N_{11}O_{10}$, 1182.67; found, 1183.4; **HRMS** (ESI) *m/z* $[M + H]^+$ calcd for $C_{65}H_{87}N_{11}O_{10}$, 1182.6710; found, 1182.6694.

L. Synthesis of MDM2-based PROTACs

Idasanutlin (6)

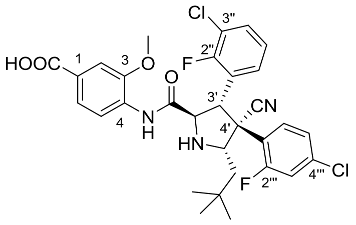

This compound was used as commercially supplied (MedChemExpress).

^{1}H NMR (600 MHz, DMSO-d_6) δ 0.96 (s, 9H (C(CH_3)$_3$), 1.26 (d, *J* = 14.1 Hz, 1H), 1.63 (dd, *J* = 9.8, 14.2 Hz, 1H, CH_2), 3.90 – 3.98 (m, 4H, OCH_3, 5'-H), 4.33 – 4.41 (m, 1H, NH), 4.56 – 4.62 (m, 2H, 2'-H, 3'-H), 7.31 – 7.41 (m, 3H), 7.49 – 7.59 (m, 4H), 7.72 (t, *J* = 7.1 Hz, 1H, Ar-H), 8.36 (d, *J* = 8.3 Hz, 1H, 2-H), 10.46 (s, 1H, CONH), 12.84 (s, 1H, COOH).

^{13}C NMR (151 MHz, DMSO-d_6) δ 29.67 (C($\underline{C}H_3$)$_3$), 30.26 ($\underline{C}$(CH_3)$_3$), 44.10 (CH_2), 50.35 (C-3'), 55.97 (OCH_3), 63.43, 63.56 (C-4', C-5'), 64.82 (C-2'), 111.22 (C-2), 117.40, 117.48 (C-1, C-3'''), 117.65, 117.83, 119.35 (d, ^{2}J(C,F) = 9.6 Hz, C-3''), 119.65 (d, ^{2}J(C,F) = 18.7 Hz, C-1'''), 122.92, 125.47 (d, ^{3}J(C,F) = 4.5 Hz), 125.80 (d, ^{3}J(C,F) = 2.7 Hz, Ar-CH), 126.07 (Ar-CH), 126.15 (d, ^{2}J(C,F) = 13.3 Hz, C-1''), 128.78, 130.22, 131.01, 131.13 (d, ^{3}J(C,F) = 4.4 Hz, Ar-CH), 134.94 (d, ^{3}J(C,F) = 11.1 Hz, C-4'''), 147.72 (C-3), 155.76 (d, ^{1}J(C,F) = 246.6 Hz, C-2''), 159.70 (d, ^{1}J(C,F) = 251.0 Hz, C-2'''), 167.03 (COOH), 171.41 (CONH).

2-[2-[2-[2-(*tert*-Butoxycarbonylamino)ethoxy]ethoxy]ethoxy]ethyl methanesulfonate (92)

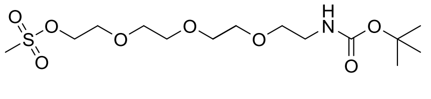

This compound was synthesized as we described previously.[7]

Palbociclib-2-2-2-2 conjugate (93)[2]

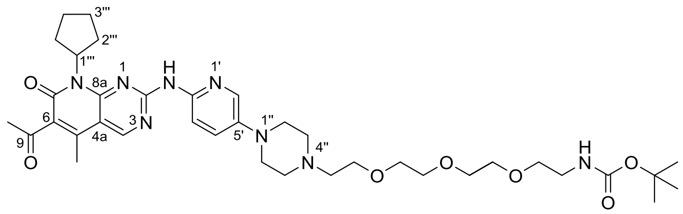

A mixture of palbociclib (0.18 mmol, 81 mg) and methanesulfonate **92** (0.36 mmol. 134 mg) in dry DMSO (5 mL) and DIPEA (0.54 mmol, 94 µL) was stirred at 80 °C for 24 h. After cooling, it was partitioned between saturated $NaHCO_3$ solution (50 mL) and EtOAc (100 mL), and the organic layer was washed with 5% LiCl solution and brine (each 50 mL), dried over Na_2SO_4, filtered and evaporated. The crude product was purified by column chromatography (CH_2Cl_2/MeOH+7N NH_3 49:1) to obtain a yellow solid.

Yield (99 mg, 76%); mp 78 – 82 °C; R_f = 0.54 (CH_2Cl_2/MeOH+7N NH_3 19:1); **^{1}H NMR** (600 MHz, DMSO-d_6) δ 1.36 (s, 9H, ($C(CH_3)_3$), 1.50 – 1.63 (m, 2H), 1.63 – 1.80 (m, 2H), 1.80 – 1.91 (m, 2H), 2.19 – 2.27 (m, 2H, 2‴-H, 3‴-H), 2.30 (s, 3H, CH_3), 2.41 (s, 3H, 10-H), 2.52 (t, *J* = 5.9 Hz, 2H), 2.53 – 2.62 (m, 4H), 3.05 (d, *J* = 6.0 Hz, 2H), 3.14 (t, *J* = 5.0 Hz, 4H), 3.37 (t, *J* = 6.1 Hz, 2H), 3.43 – 3.60 (m, 10H, 2″-H, 3″-H, NCH_2, OCH_2, NHC<u>H</u>$_2$), 5.80 (p, *J* = 8.9 Hz, 1H, 1‴-H), 6.72 (t, *J* = 5.9 Hz, 1H, N<u>H</u>CH_2), 7.44 (dd, *J* = 3.1, 9.1 Hz, 1H, 4′-H), 7.83 (d, *J* = 9.0 Hz, 1H, 3′-H), 8.03 (d, *J* = 3.0 Hz, 1H, 6′-H), 8.93 (s, 1H, 4-H), 10.05 (s, 1H, NH); **^{13}C NMR** (151 MHz, DMSO-d_6) δ 13.74 (CH_3), 25.23 (C-3‴), 27.68 (C-2‴), 28.37 (C(<u>C</u>H_3)$_3$), 31.43 (C-10), 48.46 (2C, C-2″), 53.04 (3C, C-3″, C-1‴), 57.35 (NCH_2), 68.53, 69.32, 69.68, 69.83, 69.90, 69.94 (OCH_2), 77.71 (<u>C</u>(CH_3)$_3$), 106.70 (C-4a), 115.29 (C-3′), 124.75 (C-4′), 129.35 (C-6), 135.44 (C-6′), 142.22 (C-5′), 143.61, 144.38 (C-8a, C-2′), 154.91 (C-5), 155.72 (CO), 158.38 (C-4), 158.71 (C-2), 160.90 (C-7), 202.55 (C-9). The signal for NH<u>C</u>H_2 is missing (overlapping solvent peaks); **MS** (ESI) *m/z* $[M + H]^+$ calcd for $C_{37}H_{54}N_8O_7$, 723.41; found, 723.7; **HRMS** (ESI) *m/z* $[M + H]^+$ calcd for $C_{37}H_{54}N_8O_7$, 723.4149; found, 723.4179.

MDM2-based PROTAC 37

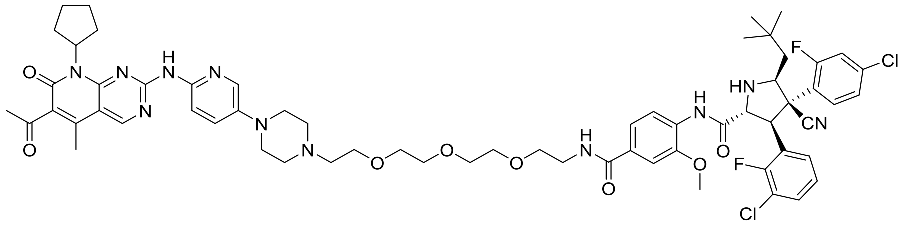

Palbociclib-2-2-2-2 conjugate **93** (82 µmol, 66 mg) was dissolved in dry CH_2Cl_2 (4 mL) and TFA (4 mL) and stirred at rt for 2 h. After removal of the volatiles, the oily residue was further dried under high vacuum. Subsequently, it was dissolved in dry DMF (4 mL) and DIPEA (60 µL, 0.34 mmol) and idasanutlin (68 µmol, 42 mg) were added. After stirring for 5 minutes, HATU (75 µmol, 28 mg) was added to the mixture and it was stirred at room temperature for 16 h. The reaction mixture was quenched with saturated $NaHCO_3$ solution (100 mL) and the product was extracted with EtOAc (3 × 50 mL). The combined organic phases were washed with 5% LiCl solution and brine (each 50 mL), dried over Na_2SO_4, filtered and concentrated *in vacuo*. The crude product was purified by column chromatography (CH_2Cl_2/MeOH+7N NH_3 35:1) followed by HPLC purification using a gradient of 60% to 90% v/v MeCN + 0.05% TFA. The title compound (TFA salt) was obtained as a light yellow solid after lyophilisation. The pure product was then neutralized by eluting it with CH_2Cl_2/MeOH+7N NH_3 19:1 on a small silica gel column. After evaporation of the solvent, a light yellow solid was obtained.

Yield (44 mg, 53%); mp 142 – 146 °C; R_f = 0.16 (CH_2Cl_2/MeOH+7N NH_3 29:1); **^{1}H NMR** (600 MHz, DMSO-d_6) δ 0.95 (s, 9H), 1.25 (d, *J* = 13.8 Hz, 1H), 1.51 – 1.67 (m, 3H), 1.69 – 1.79 (m, 2H), 1.81 – 1.92 (m, 2H), 2.17 – 2.27 (m, 2H), 2.29 (s, 3H), 2.41 (s, 3H), 2.49 – 2.65 (m, 6H), 2.93 – 3.23 (m, 4H), 3.41 (q, *J* = 5.9 Hz, 2H), 3.44 – 3.64 (m, 12H), 3.90 (s, 3H), 3.91 – 4.01 (m, 1H), 4.16 – 4.48 (m, 1H), 4.52 – 4.64 (m, 2H), 5.80 (p, *J* = 8.9 Hz, 1H), 7.30 – 7.40 (m, 3H), 7.43 (dd, *J* = 3.1, 9.2 Hz, 1H), 7.45 – 7.59 (m, 4H), 7.63 – 7.75 (m, 1H), 7.82 (d, *J* = 9.0 Hz, 1H), 8.03 (d, *J* = 3.0 Hz, 1H), 8.30 (d, *J* = 8.4 Hz, 1H), 8.45 (t, *J* = 5.7 Hz, 1H), 8.93 (s, 1H), 10.04 (s, 1H), 10.38 (s, 1H); **^{13}C NMR** (151 MHz, DMSO-d_6) δ 13.75, 25.23, 27.69, 29.63, 30.21, 31.44, 44.04, 48.45, 50.32, 53.00, 53.05, 55.95, 57.33, 63.38, 63.58, 64.78, 68.51, 69.14, 69.83, 69.91, 69.93, 106.71, 109.76, 115.30, 117.17, 117.45, 117.61, 117.79, 119.32, 119.61, 120.18, 124.75, 125.41, 125.77, 126.17, 128.74, 129.40, 129.77, 130.15, 131.11, 134.90, 135.45, 142.21, 143.60, 144.40, 147.65, 154.91, 155.72, 158.38, 158.72, 159.65, 160.91, 165.68, 171.16, 202.56; **LC-MS** (ESI) (90% H_2O to 100% MeOH in 10 min, then 100% MeOH to 20 min, DAD 220-420 nm), t_R = 13.33 min, 99% purity, *m/z* $[M + H]^+$ calcd for $C_{63}H_{73}Cl_2F_2N_{11}O_8$, 1220.50; found, 1220.9; **HRMS** (ESI) *m/z* $[M + H]^+$ calcd for $C_{63}H_{73}Cl_2F_2N_{11}O_8$, 1220.5062; found, 1220.5050.

Pomalidomide conjugate 94

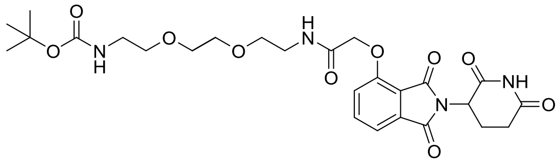

This compound was synthesized as reported previously.[17]

^{1}H NMR (600 MHz, DMSO-d_6) δ 1.35 (s, 9H, CH_3), 1.92 – 2.15 (m, 1H, 4'-H), 2.50 – 2.71 (m, 2H, 4'-H, 5'-H), 2.76 – 2.95 (m, 1H, 5'-H), 3.04 (q, *J* = 6.0 Hz, 2H), 3.26 – 3.33 (m, 2H), 3.36 (t, *J* = 6.1 Hz, 2H), 3.45 (t, *J* = 5.7 Hz, 2H), 3.49 (h, *J* = 2.2 Hz, 4H, NHC$\underline{H}_2$, OCH_2), 4.78 (s, 2H, OCH_2), 5.10 (dd, *J* = 5.5, 12.9 Hz, 1H, 3'-H), 6.71 (t, *J* = 5.8 Hz, 1H, N$\underline{H}$$CH_2$), 7.39 (d, *J* = 8.5 Hz, 1H, 5-H), 7.49 (d, *J* = 7.2 Hz, 1H, 7-H), 7.80 (dd, *J* = 7.3, 8.5 Hz, 1H, 6-H), 7.98 (t, *J* = 5.7 Hz, 1H, N$\underline{H}$$CH_2$), 11.09 (br s, 1H, NH); **^{13}C NMR** (151 MHz, DMSO-d_6) δ 22.14 (C-4'), 28.36 (C($\underline{C}H_3)_3$), 31.08 (C-5'), 38.55 ($NHCH_2$), 48.95 (C-3'), 67.68, 68.96, 69.31, 69.60, 69.71 (OCH_2), 77.73 ($\underline{C}(CH_3)_3$), 116.18 , 116.93 (C-3a, C-5), 120.52 (C-7), 133.19 (C-6), 137.06 (C-7a), 155.14 (C-4), 155.72, 165.58 (CO), 166.87, 167.03 (C-1, C-3), 169.98 (C-2'), 172.88 (C-6'); one signal for NH$\underline{C}H_2$ is missing (overlapping residual solvent peaks). **MS** (ESI) *m/z* $[M + H]^+$ calcd for $C_{26}H_{34}N_4O_{10}$, 563.23; found, 563.5.

MDM2-based degrader 95

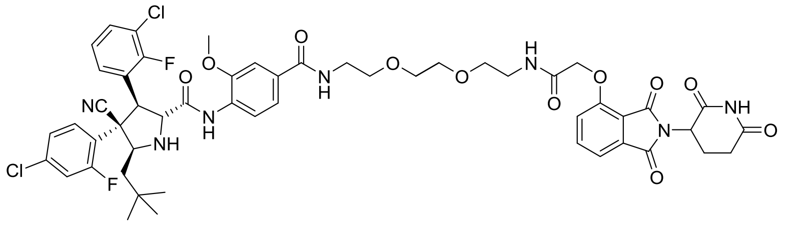

Pomalidomide conjugate **94** (63 µmol, 36 mg) was dissolved in dry CH_2Cl_2 (4 mL) and TFA (4 mL) and stirred at rt for 2 h. After removal of the volatiles, the oily residue was further dried under high vacuum. Subsequently, it was dissolved in dry DMF (4 mL) and DIPEA (55 µL, 0.32 mmol) and idasanutlin (63 µmol, 39 mg) were added. After stirring for 5 minutes, HATU (70 µmol, 27 mg) was added to the mixture and it was stirred at room temperature for 16 h. The reaction mixture was quenched with saturated $NaHCO_3$ solution (100 mL) and the product was extracted with EtOAc (3 × 50 mL). The combined organic phases were washed with 5% LiCl solution and brine (each 50 mL), dried over Na_2SO_4, filtered and concentrated *in vacuo*. The crude product was purified by column chromatography (CH_2Cl_2/MeOH+7N NH_3 29:1) followed by HPLC purification using 80% v/v MeCN + 0.05% TFA. The title compound (TFA salt) was obtained as a colorless solid after lyophilisation. The pure product was then neutralized by eluting it with CH_2Cl_2/MeOH+7N NH_3 19:1 on a small silica gel column. After evaporation of the solvent, a colorless solid was obtained.

Yield (53 mg, 72%); mp 116 – 120 °C; R_f = 0.32 (CH_2Cl_2/MeOH+7N NH_3 19:1); ^{1}**H NMR** (600 MHz, DMSO-d_6) δ 0.98 (s, 9H), 1.28 (d, *J* = 13.9 Hz, 1H), 1.65 (dd, *J* = 9.8, 14.2 Hz, 1H), 2.00 – 2.09 (m, 1H), 2.51 – 2.73 (m, 2H), 2.77 – 3.02 (m, 1H), 3.27 – 3.36 (m, 2H), 3.41 (q, *J* = 6.0 Hz, 2H), 3.47 (t, *J* = 5.7 Hz, 2H), 3.51 – 3.67 (m, 6H), 3.92 (s, 3H), 3.93 – 4.01 (m, 1H), 4.07 – 4.56 (m, 1H), 4.56 – 4.72 (m, 2H), 4.78 (s, 2H), 5.12 (dd, *J* = 5.5, 12.9 Hz, 1H), 7.26 – 7.44 (m, 4H), 7.44 – 7.51 (m, 2H), 7.51 – 7.57 (m, 2H), 7.58 (dd, *J* = 2.1, 12.6 Hz, 1H), 7.65 – 7.78 (m, 1H), 7.80 (dd, *J* = 7.2, 8.5 Hz, 1H), 7.99 (t, *J* = 5.7 Hz, 1H), 8.31 (d, *J* = 8.3 Hz, 1H), 8.46 (t, *J* = 5.7 Hz, 1H), 10.40 (s, 1H), 11.10 (br s, 1H); ^{13}**C NMR** (151 MHz, DMSO) δ 22.14, 29.65, 30.23, 31.09, 38.55, 40.23, 44.05, 48.97, 50.33, 55.95, 63.38, 63.55, 64.77, 67.68, 68.97, 69.16, 69.74, 109.75, 116.18, 116.93, 117.17, 117.46, 117.62, 117.80, 119.33, 119.62, 120.18, 120.50, 125.43, 125.77, 126.17, 128.75, 129.44, 129.74, 130.16, 131.11, 133.18, 134.89, 137.04, 147.64, 155.11, 155.73, 159.67, 165.58, 165.71, 166.86, 167.04, 169.99, 171.17, 172.89; **LC-MS** (ESI) (90% H_2O to 100% MeOH in 10 min, then 100% MeOH to 20 min, DAD 220-400 nm), t_R = 12.67 min, 99% purity, *m/z* $[M + H]^+$ calcd for $C_{52}H_{53}Cl_2F_2N_7O_{11}$, 1060.32; found, 1060.6; **HRMS** (ESI) *m/z* $[M + Na]^+$ calcd for $C_{52}H_{53}Cl_2F_2N_7O_{11}$, 1082.3040; found, 1082.2934.

M. Selected Analytical Spectra

Liquid chromatography analysis of **34** (CST651).

Chromatogram and Results

Injection Details

Injection Name:	**CST 651**	*Run Time (min):*	**14,50**
Vial Number:	**RB7**	*Injection Volume:*	**2,00**
Injection Type:	**Unknown**	*Channel:*	**UV_VIS_3**
Calibration Level:		*Wavelength:*	**254**
Instrument Method:	**Purity_PROTAC**	*Bandwidth:*	**16**
Processing Method:	**Quantitative**	*Dilution Factor:*	**1,0000**
Injection Date/Time:	**28.okt..19 19:23**	*Sample Weight:*	**1,0000**

Chromatogram

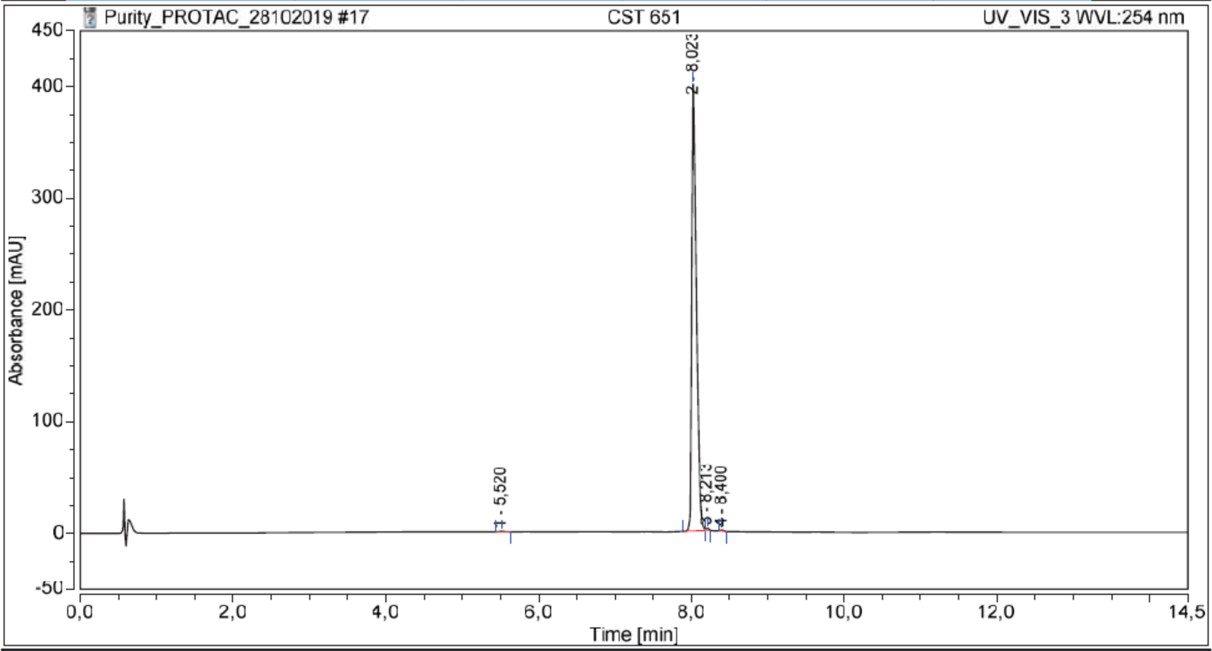

Integration Results

No.	Peak Name	Retention Time min	Area mAU*min	Height mAU	Relative Area %	Relative Height %	Amount n.a.
1		5,520	0,031	0,549	0,11	0,14	n.a.
2		8,023	27,948	400,075	99,49	99,20	n.a.
3		8,213	0,071	1,777	0,25	0,44	n.a.
4		8,400	0,041	0,886	0,15	0,22	n.a.
**Total:**			**28,092**	**403,287**	**100,00**	**100,00**	

High resolution mass spectrometry analysis of **34** (CST651).

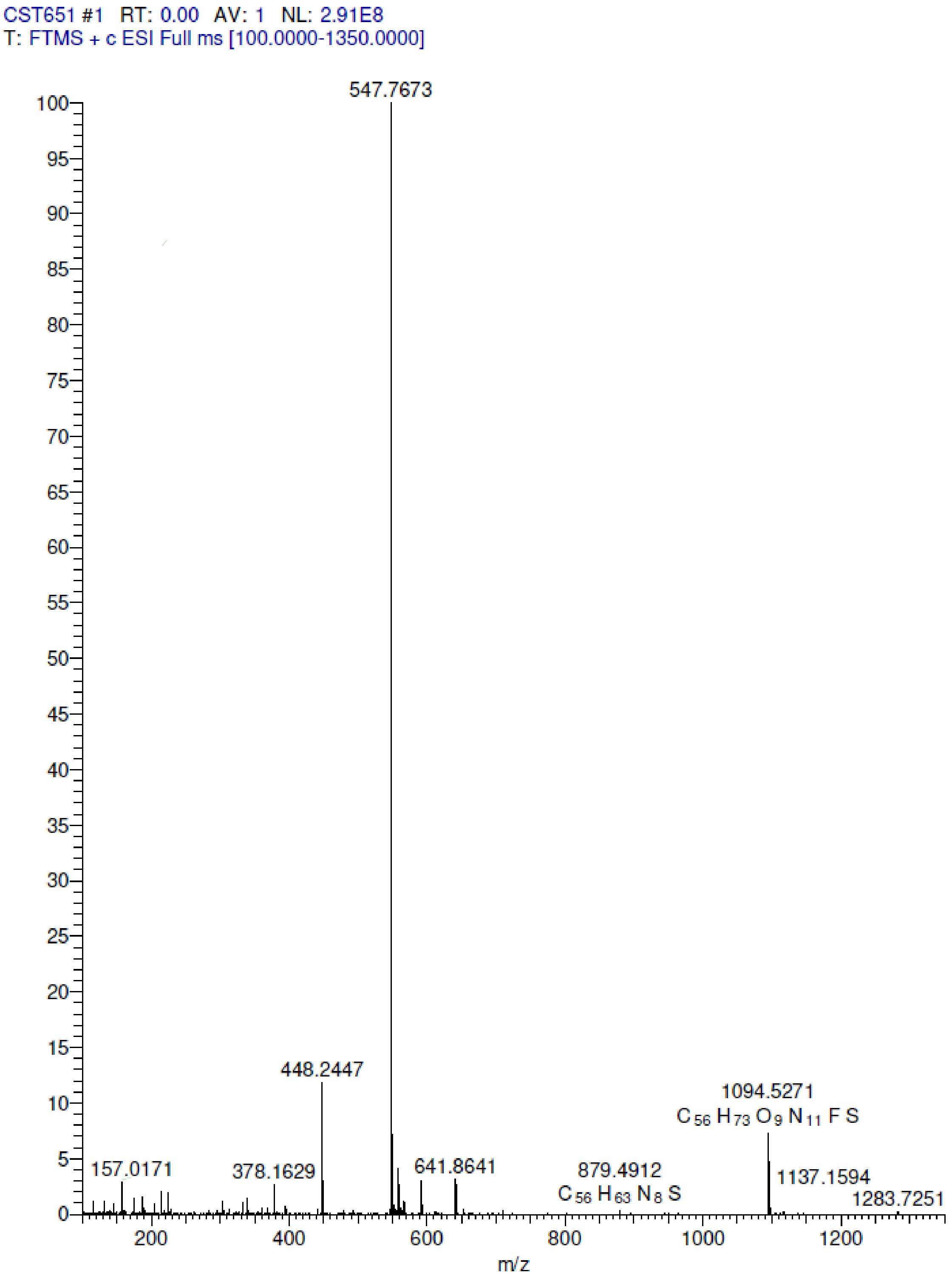

Elemental composition search on mass 1094.5271

m/z= 1089.5271-1099.5271

m/z	Theo. Mass	Delta (ppm)	RDB equiv.	Composition
1094.52710	1094.52920	-1.92	25.5	$C_{56}H_{73}O_{9}N_{11}FS$

^{1}H and ^{13}C NMR spectrum of compound **34** (CST651).

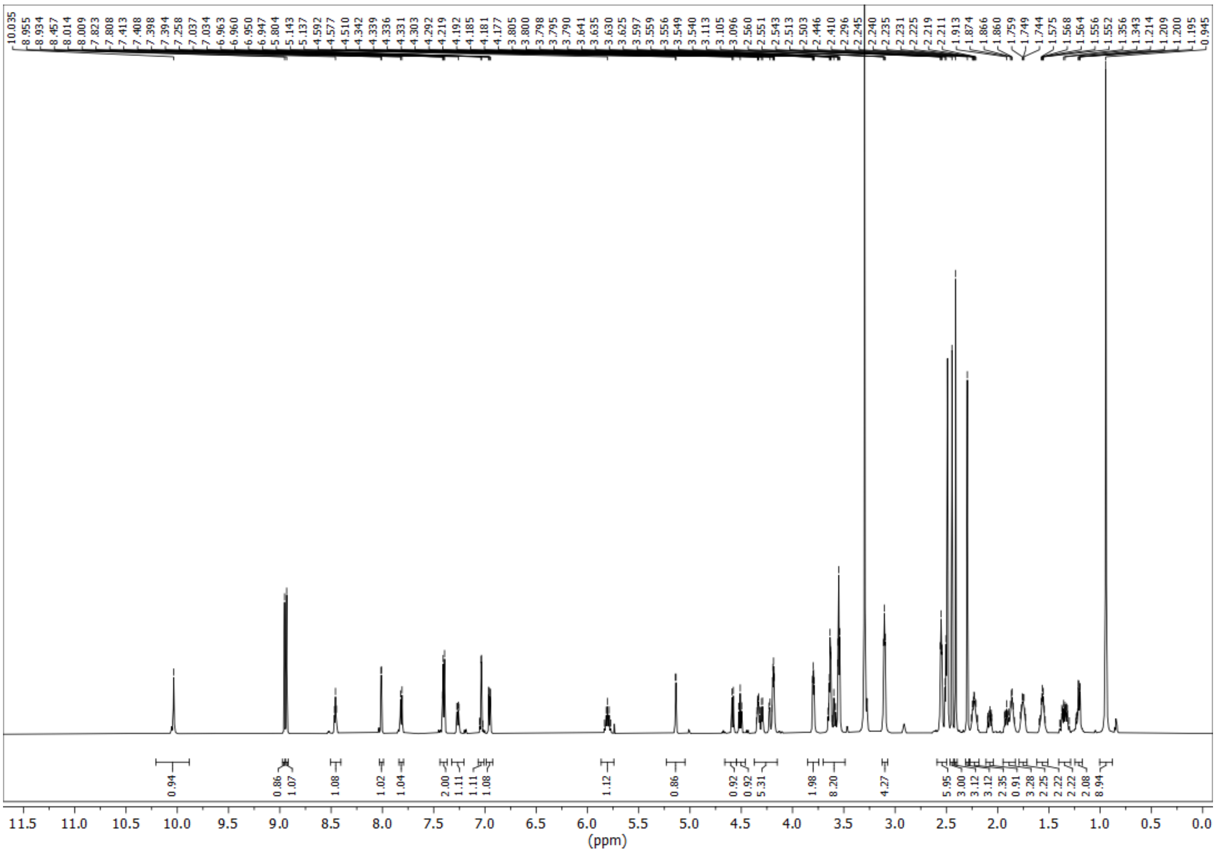

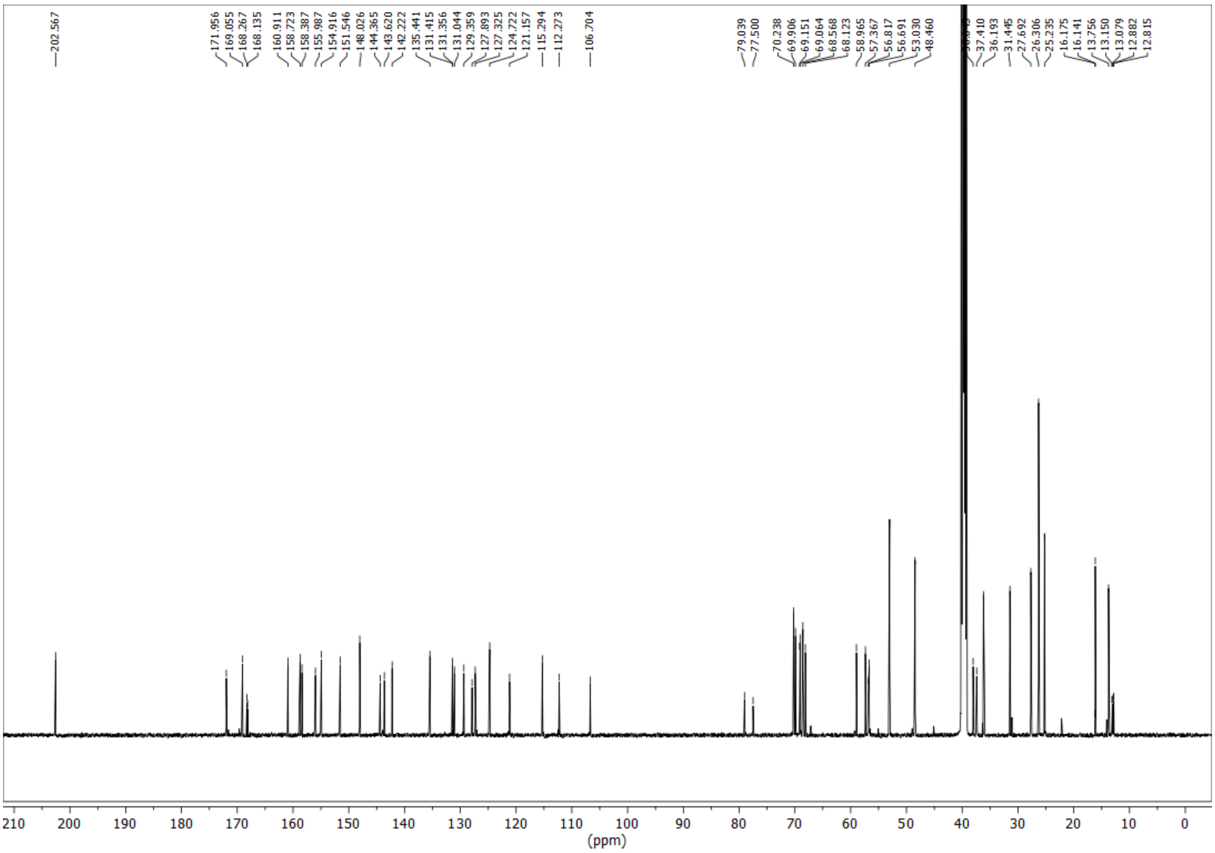

N. Molecular Descriptor Calculation

Predicted values for the topological polar surface area (TPSA), the number of rotatable bonds (NRotB), as well as hydrogen bond donors (HBD) and acceptors (HBA) were calculated using MarvinSketch 17.28.0 (ChemAxon). The degrader score (Deg_S)[1] as an overall measure of PROTAC efficacy was calculated based on our western blotting data (see Table 1–5).

O. Physicochemical Measurements

The determination of the elog $D_{7.4}$ values was performed by a chromatographic method according to the literature.[18] HPLC analysis was conducted on a Waters 2696 Separation Module coupled with a Waters 996 Photodiode Array Detector. The column was a Polaris C18-A column (2 mm I.D., 50 mm length, 3 µm particle size) from Agilent Technologies. A linear mobile phase gradient was applied with 20 µM TRIS buffer adjusted to *p*H 7.4 and MeCN. Therefore, the gradient was run from 5% MeCN to 95% within 5 min, maintained for 1.5 min to be followed by an equilibration time of 5.5 min between the runs with a constant flow-rate of 0.8 ml/min and a temperature of 25 °C. The output wavelength of the detector was set to 269.5 nm. In order to calibrate the log $D_{7.4}$ to the retention time, 6 different drugs with known log $D_{7.4}$ were dissolved in DMSO and 10 µl were injected (Table S7). By plotting the log $D_{7.4}$ versus the retention times a linear calibration line (R^2 = 0.996) could be obtained (Figure S11). Approximately 1 mg of the samples was dissolved in DMSO and 5 µl were injected. Analyses were conducted in duplicates. Subsequently, the mean retention times of the analytes were taken to calculate their log $D_{7.4}$ values with aid of the calibration line.

Compound	Concentration [mg/ml]	Mean retention time [min]	Lit. log $D_{7.4}$ [18,19]
Atenolol	1.5	1.630	–1.38
Metoprolol	4.0	2.456	–0.06
Labetalol	0.12	2.866	1.07
Diltiazem	0.075	3.614	2.70
Triphenylene	0.01	5.093	5.49
Permethrin	0.1	5.740	6.50

Table S7: Determined retention times of the calibration compounds and log $D_{7.4}$ values.

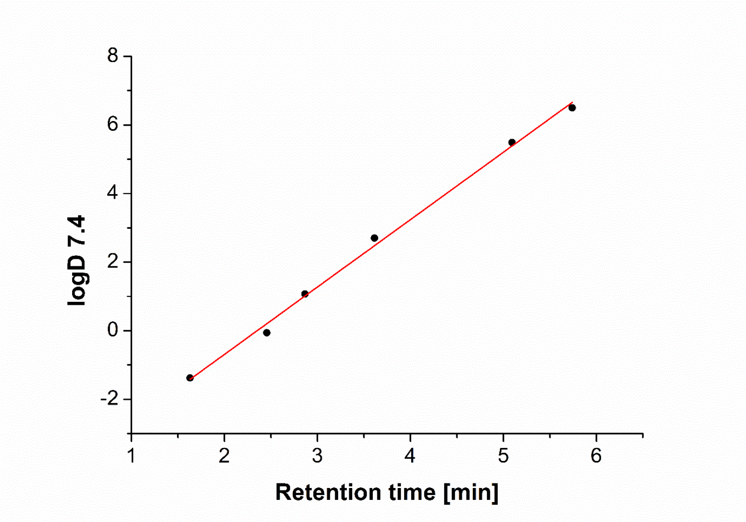

Fig. S18 Calibration line – log $D_{7.4}$ versus retention time.

References

1 H. J. Maple, N. Clayden, A. Baron, C. Stacey and R. Felix, *Med. Chem. Commun.*, 2019, **10**, 1755–1764.

2 M. Brand, B. Jiang, S. Bauer, K. A. Donovan, Y. Liang, E. S. Wang, R. P. Nowak, J. C. Yuan, T. Zhang, N. Kwiatkowski, A. C. Müller, E. S. Fischer, N. S. Gray and G. E. Winter, *Cell Chem. Biol.*, 2019, **26**, 300-306.e9.

3 R. M. Meyers, J. G. Bryan, J. M. McFarland, B. A. Weir, A. E. Sizemore, H. Xu, N. V. Dharia, P. G. Montgomery, G. S. Cowley, S. Pantel, A. Goodale, Y. Lee, L. D. Ali, G. Jiang, R. Lubonja, W. F. Harrington, M. Strickland, T. Wu, D. C. Hawes, V. A. Zhivich, M. R. Wyatt, Z. Kalani, J. J. Chang, M. Okamoto, K. Stegmaier, T. R. Golub, J. S. Boehm, F. Vazquez, D. E. Root, W. C. Hahn and A. Tsherniak, *Nat. Genet.*, 2017, **49**, 1779–1784.

4 Broad DepMap, 2019.

5 J. M. Dempster, J. Rossen, M. Kazachkova, J. Pan, G. Kugener, D. E. Root and A. Tsherniak, *Extracting Biological Insights from the Project Achilles Genome-Scale CRISPR Screens in Cancer Cell Lines*, biorxiv, 2019.

6 D. Heckl, M. S. Kowalczyk, D. Yudovich, R. Belizaire, R. V. Puram, M. E. McConkey, A. Thielke, J. C. Aster, A. Regev and B. L. Ebert, *Nat. Biotechnol.*, 2014, **32**, 941–946.

7 C. Steinebach, I. Sosič, S. Lindner, A. Bricelj, F. Kohl, Y. L. D. Ng, M. Monschke, K. G. Wagner, J. Krönke and M. Gütschow, *Med. Chem. Commun.*, 2019, **10**, 1037–1041.

8 C. Steinebach, H. Kehm, S. Lindner, L. P. Vu, S. Köpff, Á. López Mármol, C. Weiler, K. G. Wagner, M. Reichenzeller, J. Krönke and M. Gütschow, *Chem. Commun.*, 2019, **55**, 1821–1824.

9 N. D. Bogdan, M. Matache, V. M. Meier, C. Dobrotă, I. Dumitru, G. D. Roiban and D. P. Funeriu, *Chem. - Eur. J.*, 2010, **16**, 2170–2180.

10 M. Zengerle, K.-H. Chan and A. Ciulli, *ACS Chem. Biol.*, 2015, **10**, 1770–1777.

11 K. Raina, J. Lu, Y. Qian, M. Altieri, D. Gordon, A. M. K. Rossi, J. Wang, X. Chen, H. Dong, K. Siu, J. D. Winkler, A. P. Crew, C. M. Crews and K. G. Coleman, *Proc. Natl. Acad. Sci.*, 2016, **113**, 7124–7129.

12 J. Hu, B. Hu, M. Wang, F. Xu, B. Miao, C.-Y. Yang, M. Wang, Z. Liu, D. F. Hayes, K. Chinnaswamy, J. Delproposto, J. Stuckey and S. Wang, *J. Med. Chem.*, 2019, **62**, 1420–1442.

13 D. L. Buckley, K. Raina, N. Darricarrere, J. Hines, J. L. Gustafson, I. E. Smith, A. H. Miah, J. D. Harling and C. M. Crews, *ACS Chem. Biol.*, 2015, **10**, 1831–1837.

14 V. Zoppi, S. J. Hughes, C. Maniaci, A. Testa, T. Gmaschitz, C. Wieshofer, M. Koegl, K. M. Riching, D. L. Daniels, A. Spallarossa and A. Ciulli, *J. Med. Chem.*, 2019, **62**, 699–726.

15 T. K. Oost, C. Sun, R. C. Armstrong, A.-S. Al-Assaad, S. F. Betz, T. L. Deckwerth, H. Ding, S. W. Elmore, R. P. Meadows, E. T. Olejniczak, A. Oleksijew, T. Oltersdorf, S. H. Rosenberg, A. R. Shoemaker, K. J. Tomaselli, H. Zou and S. W. Fesik, *J. Med. Chem.*, 2004, **47**, 4417–4426.

16 N. Ohoka, Y. Morita, K. Nagai, K. Shimokawa, O. Ujikawa, I. Fujimori, M. Ito, Y. Hayase, K. Okuhira, N. Shibata, T. Hattori, T. Sameshima, O. Sano, R. Koyama, Y. Imaeda, H. Nara, N. Cho and M. Naito, *J. Biol. Chem.*, 2018, **293**, 6776–6790.

17 J. Lohbeck and A. K. Miller, *Bioorg. Med. Chem. Lett.*, 2016, **26**, 5260–5262.

18 E. H. Kerns, L. Di, S. Petusky, T. Kleintop, D. Huryn, O. McConnell and G. Carter, *J. Chromatogr. B*, 2003, **791**, 381–388.

19 A. Finizio, M. Vighi and D. Sandroni, *Chemosphere*, 1997, **34**, 131–161.
